# Late‐Stage Heteroarylation of Hetero(aryl)sulfonium Salts Activated by α‐Amino Alkyl Radicals

**DOI:** 10.1002/anie.202103085

**Published:** 2021-05-05

**Authors:** Eva Maria Alvarez, Teresa Karl, Florian Berger, Luca Torkowski, Tobias Ritter

**Affiliations:** ^1^ Max-Planck-Institut für Kohlenforschung Kaiser-Wilhelm-Platz 1 45470 Mülheim an der Ruhr Germany

**Keywords:** heteroarylation, late-stage functionalization, radical reaction, sulfonium salts, α-amino alkyl radical

## Abstract

We report a late‐stage heteroarylation of aryl sulfonium salts through activation with α‐amino alkyl radicals in a mechanistically distinct approach from previously reported halogen‐atom transfer (XAT). The new mode of activation of aryl sulfonium salts proceeds in the absence of light and photoredox catalysts, engaging a wide range of hetarenes. Furthermore, we demonstrate the applicability of this methodology in synthetically useful cross‐coupling transformations.

## Graphical abstract

A new mode of activation of sulfonium salts by α‐amino alkyl radicals is reported. The C−S bond of the aryl sulfonium salt is cleaved by the action of α‐amino alkyl radicals in the absence of light and photoredox catalysis. The methodology enables a late‐stage heteroarylation of hetero(aryl) sulfonium salts.

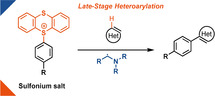

Aryl sulfonium salts are reactive reagents, which participate in synthetically useful reactions such as palladium‐catalyzed cross‐coupling reactions,^[1]^ photoredox‐catalyzed reactions,^[2]^ sulfur(IV) reductive elimination,^[3]^ and cine‐substitution.^[4]^ The accessibility, high stability, and high redox potential make aryl sulfonium salts useful precursors for aryl radicals. Known methods for generating aryl radicals from sulfonium salts require a photocatalyst, in most cases a transition metal complex, and irradiation.^[5]^ Recently, progress was made in the use of α‐amino alkyl radicals as initiators in photoredox catalysis to generate aryl radicals from aryl halides via halogen‐atom transfer.^[6]^ However, little is known about the role of α‐amino alkyl radicals as strong reductants to transfer electrons to organic molecules and ensuing bond‐forming reactions.^[7]^ Herein, we report a mechanistically different approach for activating sulfonium salts with α‐amino alkyl radicals, which allows for an operationally simple protocol that proceeds without the need for a catalyst, irradiation, or inert atmosphere. The methodology reported here generates a strong reductant (α‐amino alkyl radicals) from a weak reductant (amine) through oxidation and deprotonation, a process known as reductant upconversion.^[8]^ We have applied this new mode of C−S bond activation for the development of a late‐stage heteroarylation of aryl sulfonium salts. The synergistic cooperation of sodium persulfate and tributylamine allows for the generation of in situ formed α‐amino alkyl radicals and enables access to C(*sp* ^*2*^)–C(*sp* ^*2*^) cross‐coupling of aryl and hetaryl sulfonium salts with hetarenes. We anticipate that our method will find application in medicinal chemistry and material science to prepare heterobiaryls, which are hard to access otherwise.

C−H arylation of arenes was first reported by Gomberg and Bachmann^[9]^ in 1924, when diazonium salts were treated with benzene. Although aryl radicals can easily be generated from aryl diazonium salts^[10]^ (E(PhN_2_^+^/PhN_2_^.^)=−0.16 V vs. SCE),^[11]^ the preparation of the often unstable diazonium salts requires several steps when starting from an unfunctionalized arene. More readily accessible aryl halides can be used to generate aryl radicals in presence of KO*t*Bu at elevated temperature, however such harsh conditions prevent the use of functionally more complex small molecules, and only a few examples were reported so far.^[12]^ A more functional‐group‐tolerant method was developed by König et al.^[13]^ by developing a photoredox catalyst that becomes sufficiently reducing through double excitation to reduce aryl halides (E(MeOC_6_H_4_I/ MeOC_6_H_4_I^−.^)=−2.17 V vs. SCE). Alternatively, the aryl halide can be activated by halogen atom abstraction using silyl‐,^[14]^ stannyl‐,^[15]^ or α‐amino alkyl radicals,^[6]^ however none of those atom‐abstraction processes are amenable to late‐stage functionalization nor known to enable C−H arylation of electron‐poor hetarenes. Conventional cross‐coupling reactions can be a more direct approach to form the biaryl structures but require the prefunctionalization of both reaction partners, and hetarene halides can be difficult to access.^[16]^ Aryl sulfonium salts can be more reactive than aryl halides, and can be activated in multiple types of reactions (Scheme 1 A), yet they are more thermally stable and hence safer than diazonium salts, as well as more accessible for complex small molecules. In addition, they can be accessed directly in a single step by C−H functionalization. The generation of aryl radicals from thianthrenium (TT) salts (*E*(PhTT^+^/PhTT^.^)=−1.5 V vs. SCE) has been accomplished by using photoredox catalysts, enabling a variety of cross coupling reactions.[^2a^, ^2b^, ^2c^, ^2d^, ^2e^, ^5b^] Notably, the use of α‐amino alkyl radicals for the activation of sulfonium salts has not yet been reported before into the multifaceted reactivity of the sulfonium salts. In combination with the exceptional site‐selectivity of thianthrenium salts, the new activation mode of sulfonium salts enables the installation of a greater diversity of hetarenes in a late‐stage process, otherwise not readily accessible. This in turn allows to assemble challenging linked heteroaryl–(hetero)aryl products. As part of exploiting the versatility of this new mode of activation of aryl sulfonium salts, we also expanded the reactivity to other synthetic transformations such as borylation,[^17b^, ^17c^, ^17d^] iodination^[17c]^ and allylation^[17a]^ beyond their addition to heterocycles.

**Scheme 1:**
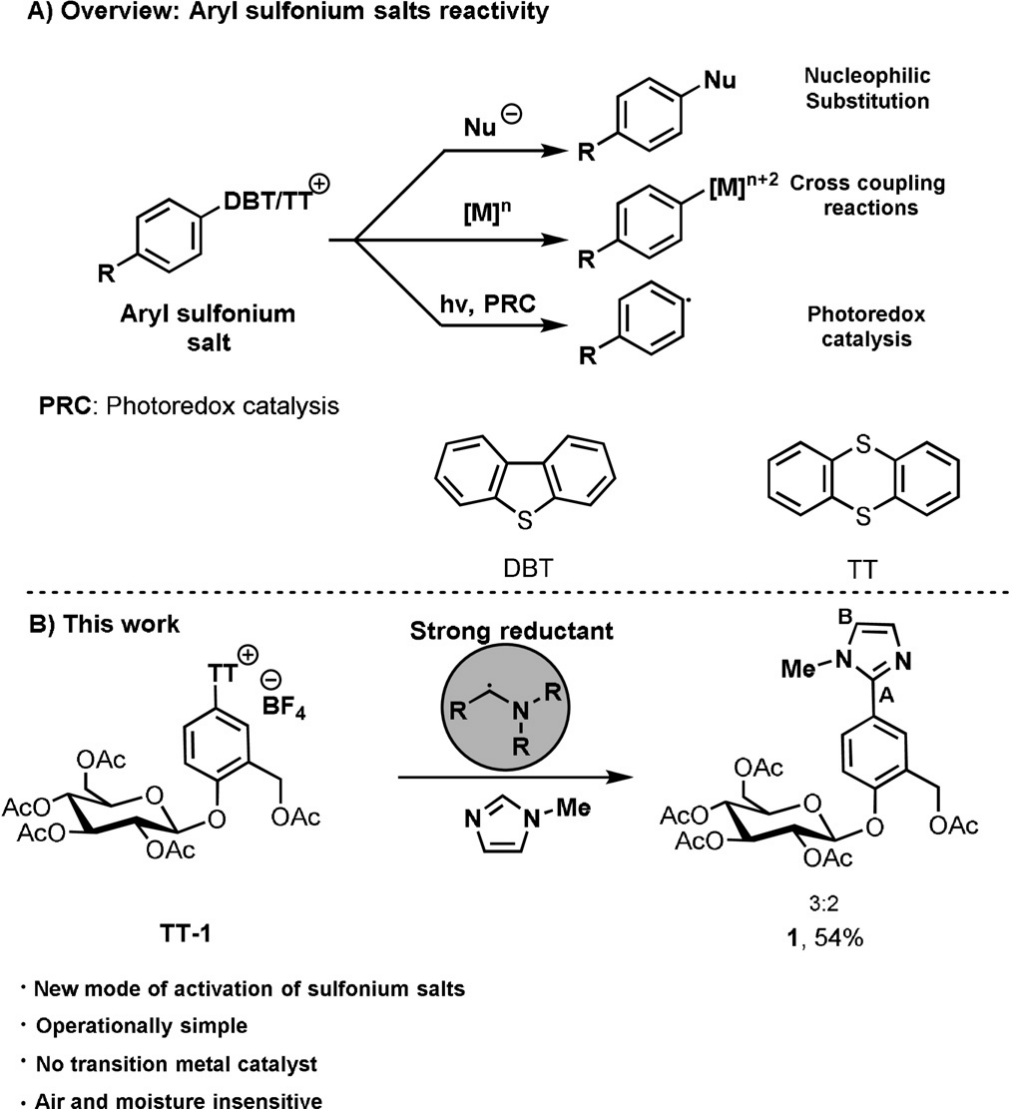
Methods available for the activation of sulfonium salts.

In the process of developing a general *cine*‐subtitution^[4]^ by using the unique reactivity of sulfonium salts as pseudo‐Michael acceptors, we questioned whether nucleophilic radicals could attack on the electron‐poor sulfonium salt. In line with this rational design, we explored alkyl halides as alkyl radical precursors through activation with α‐amino alkyl radicals as described recently by Leonori et al.^[6a]^ However, instead, we discovered a homolytic C−S bond activation of the thianthrenium salt resulting in thianthrene, aryl halide and hydrodefunctionalized product. Based on these observations, we envisioned the use of α‐amino alkyl radicals to afford selective aryl radical generation and enable heteroarylation of various (hetero)aryl sulfonium salts that has been challenging to access with conventional modes of reactivity of sulfonium salts.^[5c]^ The α‐amino alkyl radicals are generated in situ by oxidation of an amine with persulfate (*E*(S_2_O_8_^2−^/SO_4_^2−^)=+2.01 V vs. SCE), making the reaction independent of light and photoredox catalyst. Despite the success of aryl sulfonium salts as aryl radical precursors,[^5b^, ^5c^] the previous scope with respect to hetarenes has been small. Now we show how, for example, *N*‐methyl imidazole can be installed at a late‐stage in salicin pentaacetate (**1**) via the selectively accessible TT salt (Scheme 1 B).

We optimized thermal conditions for generation of α‐amino alkyl radicals and found that *n*‐Bu_3_N in combination with Na_2_S_2_O_8_ in DMSO and water are well suited. Control experiments show that no desired product is observed in the absence of Na_2_S_2_O_8_ or *n*‐Bu_3_N (see the Supporting Information, Table S1). Although aryl radicals are good H‐abstractors and amines are good H‐atom donors, excess amounts of *n*‐Bu_3_N are needed for the reaction. The *n*‐Bu_3_N is not miscible with the DMSO/ water mixture explaining why it resulted in smaller amounts of hydrodefunctionalized products compared to other amines. The biphasic reaction mixture enables the reaction in the simultaneous presence of both oxidant and reductant.^[18]^ The new mode of activation of sulfonium salts is operationally simple, and does not require inert conditions. The reaction works on a broad scope of different coupling partners (Table 1). The sulfonium salts can be derived from electron‐neutral (e.g. **3**, **13**, **15**, **17**), electron‐rich (e.g. **4**, **5**, **12**, **14**) and electron‐poor arene derivatives (e.g. **7**, **11**, **16**), polycyclic hydrocarbons (e.g. **2**, **6**), or heterocycles (e.g. **20**–**26**). The sulfonium salt can be a thianthrenium (TT) as well as a dibenzothiophenium (DBT) salt, and may carry substituents in the *para* (e.g. **3**), *meta* (e.g. **5**), and *ortho* (e.g. **11**, **16**) positions. The hetarene coupling partners can be 5‐ (e.g. **12**, **13**, **14**, **17**) or 6‐membered heterocycles (e.g. **2**, **4**, **6**). The reaction can be used to cross‐couple hetarene sulfonium salts to hetarenes to afford compounds such as **20**–**26**, which are challenging to access otherwise.^[5c]^ Although the reaction uses persulfate as a stoichiometric reagent, the functional group tolerance even towards oxidation‐sensitive substrates is high, presumably due to the presence of excess amine, so that oxidation‐sensitive *N*‐methyl pyrrole derivatives could be prepared. To highlight the applicability of our method to complex small molecules, we performed late‐stage heteroarylation of biologically relevant molecules such as boscalid (**5**, **9**), pyriproxyfen (**8**), bifonazole (**10**), indomethacin (**14**), and famoxadone (**18**).

**Table 1:** Substrate scope of (hetero)arylation of sulfonium salts.

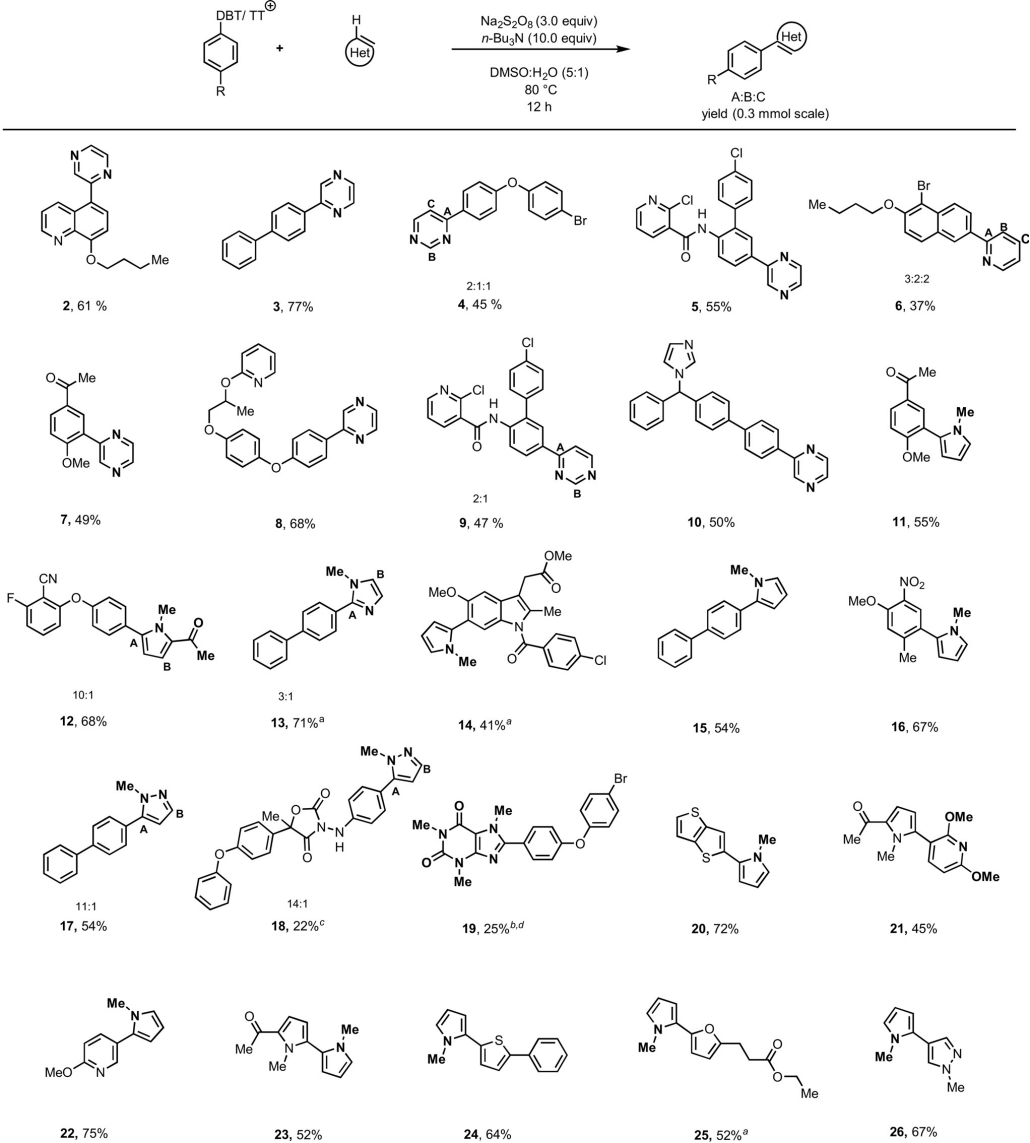

General conditions except where otherwise noted: aryl sulfonium salt (0.2–0.3 mmol), sodium persulfate (3.0 equiv), *n*‐Bu_3_N (10 equiv), hetarene (50.0 equiv), DMSO:H_2_O (0.1 M), 80 °C. [a] Sodium persulfate (5.0 equiv) and DIPEA (10.0 equiv) were used. [b] 20 mol % AgNO_3_ was added. [c] 0.12 mmol scale. [d] The reaction was run with 3.0 equiv of the hetarene. DMSO=dimethyl sulfoxide.

A proposed mechanism of the arylation reaction is shown in Scheme 2. Single‐electron oxidation of an amine produces an amine radical cation (step I), deprotonation of which yields an α‐aminoalkyl radical (step II), which is a strong reductant (*E*=−1.12 V vs. SCE).^[19]^ Generation of strong reductants from weak reductants is known as *reductant upconversion*,^[8]^ which has been used for example for the one‐electron reduction of enones.^[20]^ Remarkably, the strongly reducing α‐amino alkyl radical reacts with the thianthrenium salt (*E*(ArTT+/ ArTT^.^)=−1.5 V vs. SCE), rather than the persulfate (*E*(S_2_O_8_^2−^/SO_4_^2−^ + SO_4_^−.^)=+1.4 V vs. SCE) despite the much higher reduction potential of the persulfate. The difference in reaction rate might be a result of the large π‐system of the thianthrenium salt accelerating the rate of its reduction, as well as the structural reorganization required for the reduction of persulfate. In addition, the biphasic reaction mixture as well as the fact that the persulfate oxidant is not fully dissolved may also rationalize the chemoselective reduction of the thianthrenium salt in preference to the persulfate oxidant. Although the reduction of arylthianthrenium salts with α‐aminoalkyl radicals would be slightly uphill (+0.4 eV), the resulting arylthianthrenyl radical is unstable toward fast dissociation into thianthrene and aryl radicals (*t*_1/2_<10^−10^ s).^[2d]^ The subsequent addition of aryl radicals to (het)arenes (step IV) and the generation of (hetero)biaryls (step V) have been described in the literature.^[21]^ The C−S bond cleavage of thianthrenium salts with α‐aminoalkyl radicals could also proceed through group abstraction, analogously to the activation of arylhalides with α‐aminoalkyl radicals reported by Leonori et al.^[6]^ However, no reaction of silyl radicals with thianthrenium salts was observed (see the supporting information, Table S3), although silyl radicals are well known to abstract halogen atoms^[22]^ as well as sulfur‐containing functional groups (e.g. Barton–McCombie deoxygenation).^[23]^ The observation that the here reported arylation reaction is, like the photoredox arylation,^[5b]^ halted by the presence of a weak oxidant like nitrobenzene (*E*=−1.1 V vs. SCE),^[24]^ which is only slightly more oxidizing than the thianthrenium salt, is in line with a mechanism proceeding via single electron reduction (see the Supporting Information, Table S2). Light is not required for the transformation as it also proceeds analogously in the dark.

**Scheme 2:**
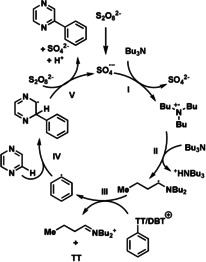
Mechanism proposal.

To demonstrate the synthetic potential of this methodology, we performed a scale‐up synthesis of compound **27** (Scheme 3), resulting in 5.41 g (55 %) of two constitutional isomers that were separated chromatographically. The transformation affords the highest yields when the heteroarene is used as a co‐solvent (20 to 50 equivalents with respect to substrate), which is appropriate for late‐stage functionalization reactions with simple yet useful heterocycles such as those shown in Table 1. The reactions typically proceed best with 50 equivalents of heterocycle but we have observed that in some cases, 20 equiv of the heteroarene can be used. It is noteworthy to point out the high chemoselectivity in the activation of sulfonium salts given that generation of α‐amino alkyl radicals can activate aryl bromides in a halogen‐atom‐transfer mechanism.^[6]^

**Scheme 3:**
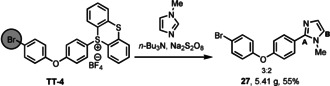
Gram‐scale synthesis. Reaction conditions: aryl sulfonium salt (30 mmol), sodium persulfate (3.0 equiv), *n*‐Bu_3_N (10 equiv), hetarene (36.0 equiv), DMSO:H_2_O (0.1 M), 80 °C, 16 h. DMSO=dimethyl sulfoxide.

Besides arylation of hetarenes, the α‐amino alkyl radical activation of sulfonium salts can also be applied to several other transformations, such as borylation, iodination, and allylation. The addition of aryl radicals to allylchloride can be used to prepare allylarenes from arylsulfonium salts (Scheme 4). The allylation of aryl sulfonium salts further explores the reactivity of the sulfonium salts in a synthetic transformation not reported before.^[25]^ Furthermore, this process does not require prior functionalization of the allylic coupling partner into organometallic reagents to engage in the conventional transition‐metal‐catalyzed cross‐coupling reactions.^[26]^

**Scheme 4:**
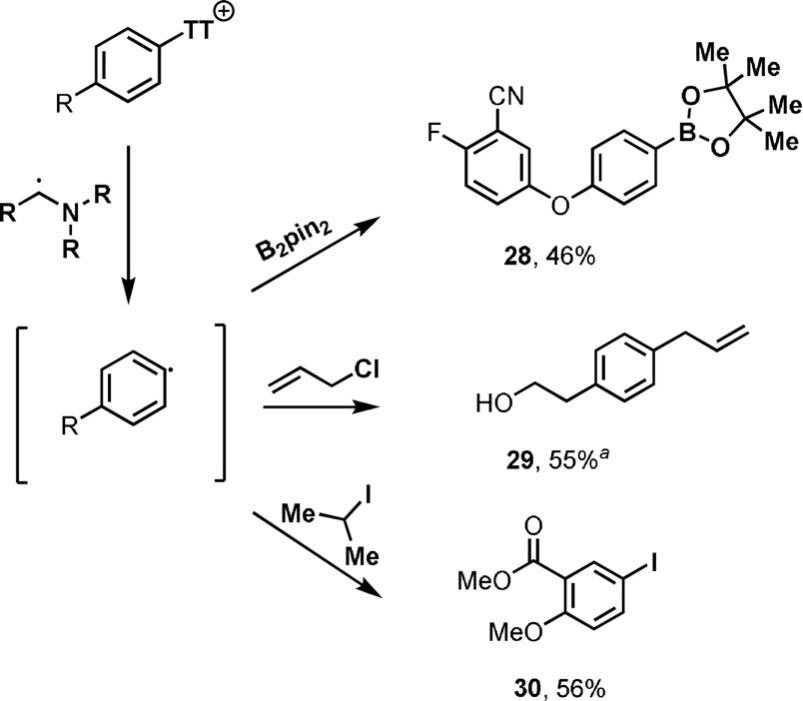
Functional group diversification with α‐amino alkyl radicals. For detailed experimental procedures, see the Supporting Information. [a] Starting from the O‐acetyl protected phenylethanol derived TT salt.

In conclusion, we have demonstrated the coupling of aryl sulfonium salts with electron‐poor and electron‐rich hetarenes through a new mode of activation of sulfonium salts which relies on a challenging coupling in presence of a strong reductant, α‐amino alkyl radical, which is known to be not compatible with oxidative conditions. The hetarene coupling partners are broad in scope, allowing the synthesis of compounds that are difficult to prepare otherwise, as for example compound **26**. The execution of the reaction is simple as it does not need any additives, light, catalysts, or transition metals, and is tolerant of air and moisture. We believe that this new mode of activation of sulfonium salts will expand the range of transformations for the late‐stage functionalization of structurally complex compounds under milder conditions.

## Conflict of interest

A patent application (number EP18204755.5, Germany), dealing with the use of thianthrene and its derivatives for C−H functionalization has been filed and F.B. and T.R. may benefit from royalty payments.

## Supporting information

As a service to our authors and readers, this journal provides supporting information supplied by the authors. Such materials are peer reviewed and may be re‐organized for online delivery, but are not copy‐edited or typeset. Technical support issues arising from supporting information (other than missing files) should be addressed to the authors.

Supplementary
