# Supplementary material for: Late‐Stage Heteroarylation of Hetero(aryl)sulfonium Salts Activated by α‐Amino Alkyl Radicals
Source: Angew Chem Int Ed Engl. 2021 May 5;60(24):13609–13. doi: 10.1002/anie.202103085 (PMC8251951; doi:10.1002/anie.202103085)
Supplement: Supplementary file 1 — Supplementary [file ANIE-60-13609-s001.pdf]

Angewandte Chemie
Eine Zeitschrift der Gesellschaft Deutscher Chemiker

Supporting Information

Late-Stage Heteroarylation of Hetero(aryl)sulfonium Salts Activated by α-Amino Alkyl Radicals

*Eva Maria Alvarez, Teresa Karl, Florian Berger, Luca Torkowski, and Tobias Ritter**

anie_202103085_sm_miscellaneous_information.pdf

TABLE OF CONTENTS

TABLE OF CONTENTS 1

MATERIALS AND METHODS 6

EXPERIMENTAL DATA 7

General procedure for thianthrenation of arenes 7

General procedure for heteroarylation of (hetero)aryl sulfonium salts 7

Reaction condition optimization 8

Evaluation of oxidant 8

Control experiments 9

Sulfonium salts synthesis 12

1-(4-Methoxyphenyl)ethan-1-one-derived thianthrenium tetrafluoroborate (**TT-7**) 12

5-Methyl-2-nitroanisole-derived thianthrenium tetrafluoroborate (**TT-16**) 13

2-Acetyl-1-methylpyrrole dibenzothiophenium tetrafluoroborate (**TT-21**) 14

Ethyl 3-(furan-2-yl)propanoate dibenzothiophenium trifluoroacetate (**TT-25**) 14

1-Methyl pyrazole-derived dibenzothiophenium trifluoroacetate (**TT-26**) 15

Heteroarylation of (hetero)aryl sulfonium salts 16

Salicin pentaacetate *N*-methyl imidazole derivative **1a, 1b** 16

8-Butoxy-5-(pyrazin-2-yl)quinoline (**2**) 17

2-([1,1'-Biphenyl]-4-yl)pyrazine (**3**) 18

1-Bromo-4-phenoxybenzene pyrimidine derivative **4a, 4b, 4c** 19

Boscalid pyrazine derivative (**5**) 20

1-Bromo-2-butoxynaphthalene pyridine derivative **6a, 6b, 6c** 21

1-(4-Methoxy-3-(pyrazin-2-yl)phenyl)ethan-1-one (**7**) 23

Pyriproxyfen pyrazine derivative (**8**) 23

Boscalid pyrimidine derivative **9a, 9b** 24

Bifonazole pyrazine derivative (**10**) 25

1-(4-Methoxy-3-(1-methyl-1H-pyrrol-2-yl)phenyl)ethan-1-one (**11**) 26

5-(4-(5-Acetyl-1-methyl-1H-pyrrol-2-yl)phenoxy)-2-fluorobenzonitrile (**12**) 27

Biphenyl imidazole derivative **13a, 13b** 27

Indomethacin-*N*-methyl pyrrole derivative (**14)** 28

2-([1,1'-Biphenyl]-4-yl)-1-methyl-1H-pyrrole (**15**) 29

2-(4-Methoxy-2-methyl-5-nitrophenyl)-1-methyl-1H-pyrrole (**16**) 30

5-([1,1'-Biphenyl]-4-yl)-1-methyl-1H-pyrazole (**17**) 31

Famoxadone *N*-methyl pyrazole derivative (**18**) 32

8-(4-(4-Bromophenoxy)phenyl)-1,3,7-trimethyl-3,7-dihydro-1H-purine-2,6-dione (**19**)........................32
1-Methyl-2-(thieno[3,2-b]thiophen-2-yl)-1H-pyrrole (**20**) 33
1-(5-(2,6-Dimethoxypyridin-3-yl)-1-methyl-1H-pyrrol-2-yl)ethan-1-one (**21**) 34
2-Methoxy-5-(1-methyl-1H-pyrrol-2-yl)pyridine (**22**)..35
1-(1,1'-Dimethyl-1H,1'H-[2,2'-bipyrrol]-5-yl)ethan-1-one (**23**)..35
1-Methyl-2-(5-phenylthiophen-2-yl)-1H-pyrrole (**24**) 36
Ethyl 3-(5-(1-methyl-1H-pyrrol-2-yl)furan-2-yl)propanoate (**25**) 37
1-Methyl-4-(1-methyl-1H-pyrrol-2-yl)-1H-pyrazole (**26**)...38

Gram-Scale Synthesis...39

1-Bromo-4-phenoxybenzene imidazole derivative **27a, 27b** 39

Diversification 40

2-Fluoro-6-(4-(4,4,5,5-tetramethyl-1,3,2-dioxaborolan-2-yl)phenoxy)benzonitrile (**28**) 40
2-(4-Allylphenyl)ethan-1-ol (**29**)..41
Methyl 5-iodo-2-methoxybenzoate (**30**) 41

SPECTROSCOPIC DATA...43

^{1}H NMR of 1-(4-methoxyphenyl)ethan-1-one-derived thianthrenium tetrafluoroborate (**TT-7**)..................43

^{13}C NMR of 1-(4-methoxyphenyl)ethan-1-one-derived thianthrenium tetrafluoroborate (**TT-7**).................44

^{19}F NMR of 1-(4-methoxyphenyl)ethan-1-one-derived thianthrenium tetrafluoroborate (**TT-7**) 45

^{1}H NMR of 5-methyl-2-nitroanisole-derived thianthrenium tetrafluoroborate (**TT-16**)................................46

^{13}C NMR of 5-methyl-2-nitroanisole-derived thianthrenium tetrafluoroborate (**TT-16**)..............................47

^{19}F NMR of 5-methyl-2-nitroanisole-derived thianthrenium tetrafluoroborate (**TT-16**) 48

HSQC of 5-methyl-2-nitroanisole-derived thianthrenium tetrafluoroborate (**TT-16**)..................................49

HMBC of 5-methyl-2-nitroanisole-derived thianthrenium tetrafluoroborate (**TT-16**) 50

^{1}H NMR of 2-acetyl-1-methylpyrrole dibenzothiophenium tetrafluoroborate (**TT-21**) 51

^{13}C NMR of 2-acetyl-1-methylpyrrole dibenzothiophenium tetrafluoroborate (**TT-21**) 52

^{19}F NMR of 2-acetyl-1-methylpyrrole dibenzothiophenium tetrafluoroborate (**TT-21**)................................53

^{1}H NMR of ethyl 3-(furan-2-yl)propanoate dibenzothiophenium trifluoroacetate (**TT-25**) 54

^{13}C NMR of ethyl 3-(furan-2-yl)propanoate dibenzothiophenium trifluoroacetate (**TT-25**)..........................55

^{19}F NMR of ethyl 3-(furan-2-yl)propanoate dibenzothiophenium trifluoroacetate (**TT-25**) 56

^{1}H NMR of 1-methyl pyrazole-derived dibenzothiophenium salt (**TT-26**)...57

^{13}C NMR of 1-methyl pyrazole-derived dibenzothiophenium salt (**TT-26**) 58

^{19}F NMR of 1-methyl pyrazole-derived dibenzothiophenium salt (**TT-26**) ... 59

^{1}H NMR of salicin pentaacetate *N*-methyl imidazole derivative (**1a**) ... 60

^{13}C NMR of salicin pentaacetate *N*-methyl imidazole derivative (**1a**) ... 61

^{1}H NMR of salicin pentaacetate *N*-methyl imidazole derivative (**1b**) ... 62

^{13}C NMR of salicin pentaacetate *N*-methyl imidazole derivative (**1b**) ... 63

^{1}H NMR of 8-butoxy-5-(pyrazin-2-yl)quinoline (**2**) ... 64

^{13}C NMR of 8-butoxy-5-(pyrazin-2-yl)quinoline (**2**) ... 65

^{1}H NMR of 2-([1,1'-biphenyl]-4-yl)pyrazine (**3**) ... 66

^{13}C NMR of 2-([1,1'-biphenyl]-4-yl)pyrazine (**3**) ... 67

^{1}H NMR of 2-(4-(4-bromophenoxy)phenyl)pyrimidine (**4a**) ... 68

^{13}C NMR of 2-(4-(4-bromophenoxy)phenyl)pyrimidine (**4a**) ... 69

^{1}H NMR of 5-(4-(4-bromophenoxy)phenyl)pyrimidine (**4b**) ... 70

^{13}C NMR of 5-(4-(4-bromophenoxy)phenyl)pyrimidine (**4b**) ... 71

^{1}H NMR of 4-(4-(4-bromophenoxy)phenyl)pyrimidine (**4c**) ... 72

^{13}C NMR of 4-(4-(4-bromophenoxy)phenyl)pyrimidine (**4c**) ... 73

^{1}H NMR of boscalid pyrazine derivative (**5**) ... 74

^{13}C NMR of boscalid pyrazine derivative (**5**) ... 75

^{1}H NMR of 2-(5-bromo-6-butoxynaphthalen-2-yl)pyridine (**6a**) ... 76

^{13}C NMR of 2-(5-bromo-6-butoxynaphthalen-2-yl)pyridine (**6a**) ... 77

^{1}H NMR of 3-(5-bromo-6-butoxynaphthalen-2-yl)pyridine (**6b**) ... 78

^{13}C NMR of 3-(5-bromo-6-butoxynaphthalen-2-yl)pyridine (**6b**) ... 79

^{1}H NMR of 4-(5-bromo-6-butoxynaphthalen-2-yl)pyridine (**6c**) ... 80

^{13}C NMR of 4-(5-bromo-6-butoxynaphthalen-2-yl)pyridine (**6c**) ... 81

^{1}H NMR of 1-(4-methoxy-3-(pyrazin-2-yl)phenyl)ethan-1-one (**7**) ... 82

^{13}C NMR of 1-(4-methoxy-3-(pyrazin-2-yl)phenyl)ethan-1-one (**7**) ... 83

^{1}H NMR of pyriproxyfen pyrazine derivative (**8**) ... 84

^{13}C NMR of pyriproxyfen pyrazine derivative (**8**) ... 85

^{1}H NMR of boscalid pyrimidine derivative (**9a**) ... 86

^{13}C NMR of boscalid pyrimidine derivative (**9a**) ... 87

^{1}H NMR of boscalid pyrimidine derivative (**9b**) 88

^{13}C NMR of of boscalid pyrimidine derivative (**9b**) 89

^{1}H NMR of bifonazole pyrazine derivative (**10**) 90

^{13}C NMR of bifonazole pyrazine derivative (**10**) 91

^{1}H NMR of 1-(4-methoxy-3-(1-methyl-1H-pyrrol-2-yl)phenyl)ethan-1-one (**11**) 92

^{13}C NMR of 1-(4-methoxy-3-(1-methyl-1H-pyrrol-2-yl)phenyl)ethan-1-one (**11**) 93

^{1}H NMR of 5-(4-(5-acetyl-1-methyl-1H-pyrrol-2-yl)phenoxy)-2-fluorobenzonitrile (**12**) 94

^{13}C NMR of 5-(4-(5-acetyl-1-methyl-1H-pyrrol-2-yl)phenoxy)-2-fluorobenzonitrile (**12**) 95

^{19}F NMR of 5-(4-(5-acetyl-1-methyl-1H-pyrrol-2-yl)phenoxy)-2-fluorobenzonitrile (**12**) 96

^{1}H NMR of 5-([1,1'-biphenyl]-4-yl)-1-methyl-1H-imidazole (**13a**) 97

^{13}C NMR of 5-([1,1'-biphenyl]-4-yl)-1-methyl-1H-imidazole (**13a**) 98

^{1}H NMR of 5-([1,1'-biphenyl]-4-yl)-1-methyl-1H-imidazole (**13b**) 99

^{13}C NMR of 5-([1,1'-biphenyl]-4-yl)-1-methyl-1H-imidazole (**13b**) 100

^{1}H NMR of indometacin-*N*-methyl pyrrole derivative (**14**) 101

^{13}C NMR of indometacin-N-methyl pyrrole derivative (**14**) 102

^{1}H NMR of 2-([1,1'-biphenyl]-4-yl)-1-methyl-1H-pyrrole (**15**) 103

^{13}C NMR of 2-([1,1'-biphenyl]-4-yl)-1-methyl-1H-pyrrole (**15**) 104

^{1}H NMR 2-(4-methoxy-2-methyl-5-nitrophenyl)-1-methyl-1H-pyrrole (**16**) 105

^{13}C NMR of 2-(4-methoxy-2-methyl-5-nitrophenyl)-1-methyl-1H-pyrrole (**16**) 106

HSQC of 2-(4-methoxy-2-methyl-5-nitrophenyl)-1-methyl-1H-pyrrole (**16**) 107

HMBC of 2-(4-methoxy-2-methyl-5-nitrophenyl)-1-methyl-1H-pyrrole (**16**) 108

^{1}H NMR of 5-([1,1'-biphenyl]-4-yl)-1-methyl-1H-pyrazole (**17**) 109

^{13}C NMR of 5-([1,1'-biphenyl]-4-yl)-1-methyl-1H-pyrazole (**17**) 110

^{1}H NMR of famoxadone *N*-methyl pyrazole derivative (**18**) 111

^{13}C NMR of famoxadone N-methyl pyrazole derivative (**18**) 112

^{1}H NMR of 8-(4-(4-bromophenoxy)phenyl)-1,3,7-trimethyl-3,7-dihydro-1H-purine-2,6-dione (**19**) 113

^{13}C NMR of 8-(4-(4-bromophenoxy)phenyl)-1,3,7-trimethyl-3,7-dihydro-1H-purine-2,6-dione (**19**) 114

^{1}H NMR of 1-methyl-2-(thieno[3,2-b]thiophen-2-yl)-1H-pyrrole (**20**) 115

^{13}C NMR of 1-methyl-2-(thieno[3,2-b]thiophen-2-yl)-1H-pyrrole (**20**) 116

^{1}H NMR of 1-(5-(2,6-dimethoxypyridin-3-yl)-1-methyl-1H-pyrrol-2-yl)ethan-1-one (**21**)........................... 117

^{13}C NMR of 1-(5-(2,6-dimethoxypyridin-3-yl)-1-methyl-1H-pyrrol-2-yl)ethan-1-one (**21**) 118

^{1}H NMR of 2-methoxy-5-(1-methyl-1H-pyrrol-2-yl)pyridine (**22**).. 119

^{13}C NMR of 2-methoxy-5-(1-methyl-1H-pyrrol-2-yl)pyridine (**22**) 120

^{1}H NMR of 1-(1,1'-dimethyl-1H,1'H-[2,2'-bipyrrol]-5-yl)ethan-1-one (**23**) 121

^{13}C NMR of 1-(1,1'-dimethyl-1H,1'H-[2,2'-bipyrrol]-5-yl)ethan-1-one (**23**)... 122

^{1}H NMR of 1-methyl-2-(5-phenylthiophen-2-yl)-1H-pyrrole (**24**) 123

^{13}C NMR of 1-methyl-2-(5-phenylthiophen-2-yl)-1H-pyrrole (**24**) 124

^{1}H NMR of ethyl 3-(5-(1-methyl-1H-pyrrol-2-yl)furan-2-yl)propanoate (**25**) 125

^{13}C NMR of ethyl 3-(5-(1-methyl-1H-pyrrol-2-yl)furan-2-yl)propanoate (**25**) 126

^{1}H NMR of 1-methyl-4-(1-methyl-1H-pyrrol-2-yl)-1H-pyrazole (**26**) 127

^{13}C NMR of 1-methyl-4-(1-methyl-1H-pyrrol-2-yl)-1H-pyrazole (**26**) 128

^{1}H NMR of 2-(4-(4-bromophenoxy)phenyl)-1-methyl-1H-imidazole (**27a**) 129

^{13}C NMR of 2-(4-(4-bromophenoxy)phenyl)-1-methyl-1H-imidazole (**27a**) 130

^{1}H NMR of 5-(4-(4-bromophenoxy)phenyl)-1-methyl-1H-imidazole (**27b**) 131

^{13}C NMR of 5-(4-(4-bromophenoxy)phenyl)-1-methyl-1H-imidazole (**27b**)... 132

^{1}H NMR of 2-fluoro-6-(4-(4,4,5,5-tetramethyl-1,3,2-dioxaborolan-2-yl)phenoxy)benzonitrile (**28**)........... 133

^{13}C NMR of 2-fluoro-6-(4-(4,4,5,5-tetramethyl-1,3,2-dioxaborolan-2-yl)phenoxy)benzonitrile (**28**) 134

^{19}F NMR of 2-fluoro-6-(4-(4,4,5,5-tetramethyl-1,3,2-dioxaborolan-2-yl)phenoxy)benzonitrile (**28**)........... 135

^{1}H NMR of 2-(4-allylphenyl)ethan-1-ol (**29**) 136

^{13}C NMR of 2-(4-allylphenyl)ethan-1-ol (**29**)... 137

^{1}H NMR of methyl 5-iodo-2-methoxybenzoate (**30**) 138

^{13}C NMR of methyl 5-iodo-2-methoxybenzoate (**30**) 139

REFERENCES.. 140

MATERIALS AND METHODS

Unless otherwise noted, all reactions were carried out under ambient atmosphere and reaction progress was monitored by thin-layer chromatography (TLC). Concentration under reduced pressure was performed by rotary evaporation at 25–40 °C at an appropriate pressure. Purified compounds were further dried under high vacuum (0.010–0.005 mBar). Yields refer to spectroscopically pure compounds. High-resolution mass spectra were obtained using *Q Exactive Plus* from *Thermo*.

Solvents

DMSO was purchased from Fischer Scientific GmbH. All deuterated solvents were purchased from Eur*iso*-Top.

Chromatography

Thin layer chromatography (TLC) was performed using EMD TLC plates pre-coated with 250 μm thickness silica gel 60 F_{254} plates. Compound visualization was achieved by fluorescence quenching under 254 nm UV light. Flash chromatography was performed using silica gel (40–63 μm particle size) purchased from Geduran.

Spectroscopy and instruments

NMR spectra were recorded on a Bruker *Ascend*^{TM} 500 spectrometer operating at 500 MHz, 471 MHz, and 126 MHz, for ^{1}H, ^{19}F, and ^{13}C acquisitions, respectively; or on a Varian Unity/Inova 600 spectrometer operating at 600 MHz and 151 MHz for ^{1}H and ^{13}C acquisitions, respectively; or on a Bruker AV300 spectrometer operating at 300 MHz and 75 MHz for ^{1}H and ^{13}C acquisitions, respectively. Chemical shifts are reported in ppm with the residual solvent signal as the internal standard. For ^{1}H NMR: $CDCl_3$, δ 7.26; CD_3CN, δ 1.94; DMSO-d_6, δ 2.50. For ^{13}C NMR: $CDCl_3$, δ 77.16; CD_3CN, δ 1.32, 118.26; DMSO-d_6, δ 39.52.[1] Data is reported as follows: s = singlet, d = doublet, t = triplet, q = quartet, m = multiplet, br = broad; coupling constants are reported in Hz.

Starting materials

All reagents were used as received from commercial suppliers unless otherwise stated. Arylthianthrenium salts **TT-1, TT-2, TT-3, TT-4, TT-5, TT-6, TT-8, TT-9, TT-10, TT-11, TT-12, TT-14, TT-18, TT-20, TT-22, TT-24, TT-29, TT-30,** thianthrene-*S*-oxide (**S1**), and dibenzothiophene-*S*-oxide **(S2)** were prepared according to the literature.[2-8]

EXPERIMENTAL DATA

General procedure for thianthrenation of arenes

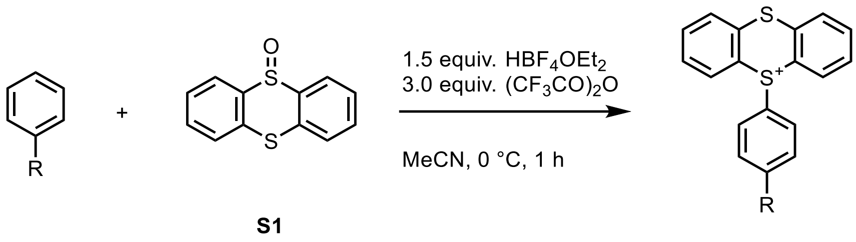

Under ambient atmosphere, a 20 mL glass-vial was charged with arene (0.50 mmol, 1.0 equiv.) and dry MeCN (c = 0.25 M). After cooling to 0 °C, $HBF_4·OEt_2$ (1.2 equiv. + 1.0 equiv. per basic functional group) was added to the vial while stirring the reaction mixture. Other acids may be used instead of $HBF_4·OEt_2$ like triflic acid (TfOH). For acid sensitive substrates $BF_3·OEt_2$ or trimethylsilyltriflate (TMSOTf) can be used. After all solids were dissolved, thianthrene-S-oxide (**S1**) (0.50 mmol, 1.0 equiv.) was added in one portion to the solution at 0 °C, leading to a suspension. Subsequently, trifluoroacetic anhydride (0.21 mL, 0.32 g, 1.5 mmol, 3.0 equiv.) was added in one portion at 0 °C, resulting in a color change to deep purple. The vial was sealed with a screw-cap. The mixture was stirred at 0 °C for 1 h, subsequently, the reaction mixture was warmed to 25 °C and stirred until all solid dissolved and the intensity of the purple color decreased. The solution was diluted with 5 mL DCM and poured onto a mixture of 30 mL DCM, 20 mL saturated aqueous Na_2CO_3 solution, and 10 ml water. After stirring for 5 min at 25 °C, the mixture was poured into a separatory funnel, and the layers were separated. The DCM layer was washed with aqueous $NaBF_4$ solution (2 × ca. 20 ml, 5% (w/w)) and with water (2 × ca. 20 mL). Washing with $NaBF_4$ solution is only required if it is of interest that the product contains only one type of counterion, solutions containing other ions, like triflate or hexafluorophosphate may be used as well. The DCM layer was dried over Na_2SO_4, filtered, and the solvent was removed under reduced pressure. In order to obtain analytically pure samples of thianthrenium salts, the residue was purified by chromatography on silica gel eluting with DCM/*i*-PrOH, subsequently, the product was dissolved in 2 mL DCM and precipitated with 20 mL Et_2O. The solid was dried in vacuo to afford the thianthrenium salt.[2]

General procedure for heteroarylation of (hetero)aryl sulfonium salts

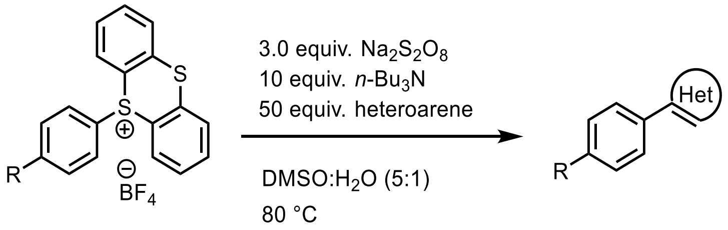

An oven-dried 25 mL round bottom flask equipped with a magnetic stir bar was charged with an aryl thianthrenium salt (0.30 mmol, 1.0 equiv.), $Na_2S_2O_8$ (0.90 mmol, 3.0 equiv.), and the heteroarene (15 mmol, 50 equiv.). Subsequently, DMSO (2.5 mL), and H_2O (0.50 mL) were added, followed by *n*-Bu_3N (3.0 mmol, 10

equiv.). The round bottom flask was capped with a septum, and the reaction mixture was placed in a preheated oil bath at 80 °C overnight. After cooling to 25 °C, EtOAc (15 mL) and H_2O (15 mL) were added to the reaction mixture. The resulting mixture was transferred to a separatory funnel and extracted with EtOAc (3 ×15 mL). The organic phase was dried over $MgSO_4$, and the solvent was removed under reduced pressure. The crude product was purified by chromatography on silica gel to afford the desired product.

Reaction condition optimization

General Procedure for optimization of reaction conditions:

In a 4 mL vial equipped with a magnetic stir bar were added biphenyl ether thianthrenium salt (**TT-9**) (20 mg, 0.04 mmol, 1.0 equiv.), $Na_2S_2O_8$ (50 mg, 0.21 mmol, 5.0 equiv.), and pyrazine (0.17 g, 2.0 mmol, 50 equiv.). Subsequently, DMSO (0.28 mL), and H_2O (0.14 mL) were added, followed by DIPEA (74 µL, 0.40 mmol, 10 equiv.). The vial was capped, and the reaction mixture was placed in a heating block at 80 °C overnight. After cooling to 25 °C, EtOAc (1.5 mL), and H_2O (1.5 mL) were added to the reaction mixture. The resulting mixture was transferred to a separatory funnel and extracted with EtOAc (3 ×5 mL). The organic phase was collected and the solvent was evaporated under reduced pressure. To the residue was added mesitylene (5.0 µL, 4.3 mg, 0.04 mmol) as an internal standard. The ^{1}H-NMR resonances of the pyrazine protons of the product between 8 and 9 ppm was integrated relative to the ^{1}H-NMR resonances of the aromatic protons of mesitylene (δ = 6.70 ppm).

Evaluation of oxidant

Table S1: Evaluation of the oxidant.

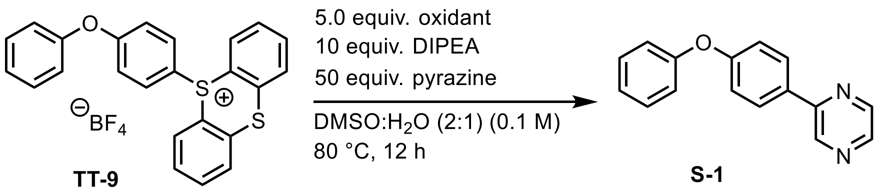

Entry	Oxidant	Yield(%)
1	$Na_2S_2O_8$	64%
2	$K_2S_2O_8$	58%
3	$(NH_4)_2S_2O_8$	60%
4	t-BuOOH	40%

Control Experiments

5	No oxidant	–
6	No amine	–

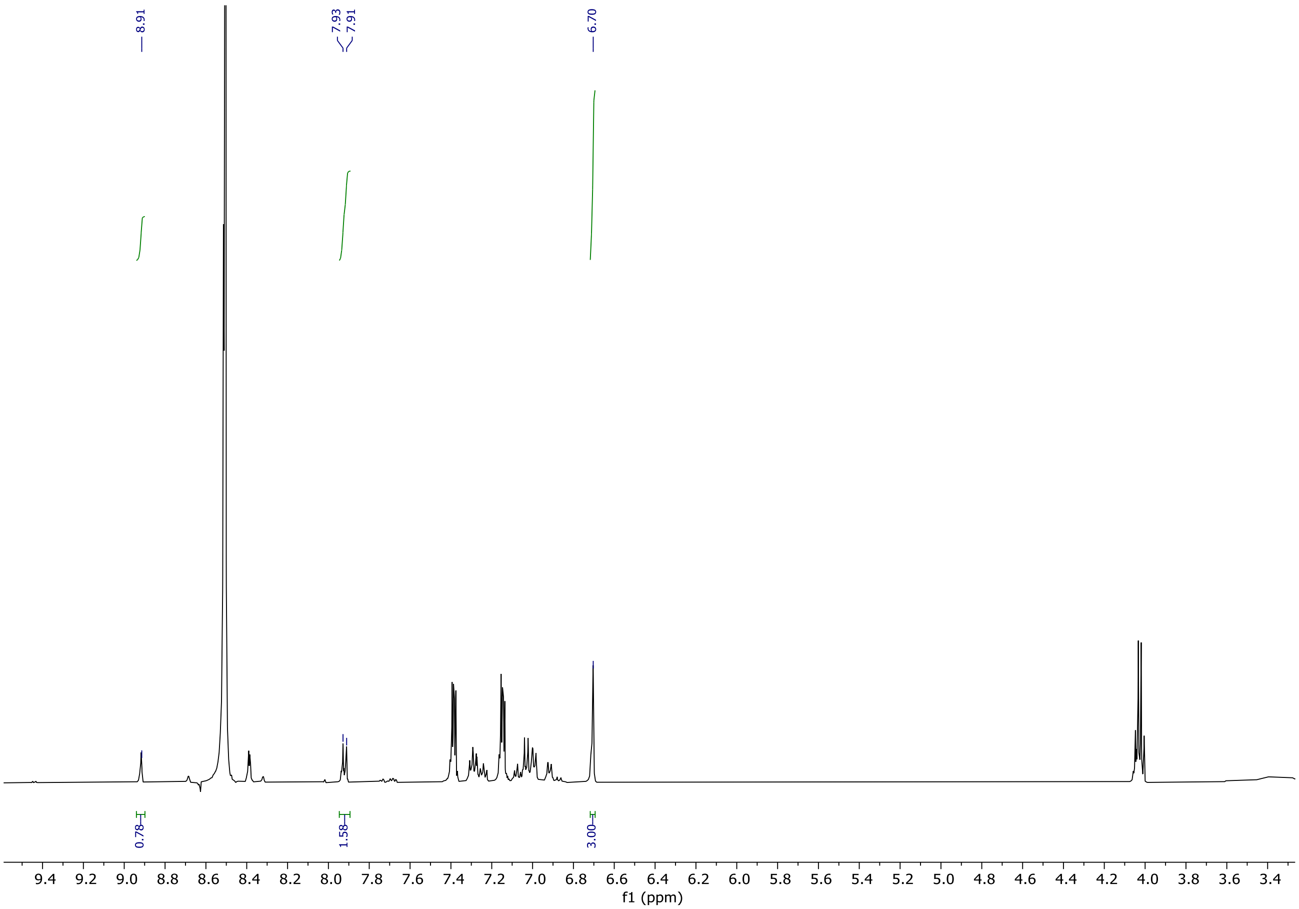

Figure S1. ^{1}H NMR of the reaction mixture after aqueous work-up with mesitylene as internal standard**.**

Control experiments

A) α-Aminoalkyl radical procedure:

Under an ambient atmosphere, a 4 ml glass vial equipped with a magnetic stir bar was charged with biphenyl thianthrenium salt (**TT-3**) (46 mg, 0.10 mmol, 1.0 equiv.), $Na_2S_2O_8$ (71 mg, 0.30 mmol, 3.0 equiv.), and pyrazine (0.40 g, 5.0 mmol, 50 equiv.). Subsequently, DMSO (0.88 mL), and H_2O (0.18 mL) were added. *n*-Bu_3N (0.24 mL, 0.19 g, 1.0 mmol, 10 equiv.) was added followed by nitrobenzene (0 mL (experiment A) or 0.05 mL, 60 mg, 0.49 mmol, 4.9 equiv. (experiment B)). The vial was sealed without degassing, and subsequently placed in an aluminium heating block preheated to 80 °C for 1 day.

B) Photoredox- catalysis procedure[2]:

Under an ambient atmosphere, a 4 ml glass vial equipped with a magnetic stir bar was charged with biphenyl thianthrenium salt (**TT-3**) (46 mg, 0.12 mmol, 1.0 equiv.), Ir[dF(CF_3)ppy]$_2$(dtbpy)PF_6 (1 mg, 1 µmol, 1 mol%), K_2CO_3 (11 mg, 0.08 mmol, 0.70 equiv.), and pyrazine (0.49 g, 6.0 mmol, 50 equiv.). Subsequently, DMSO (0.88 mL), and H_2O (0.18 mL) were added. Nitrobenzene (0 mL (experiment C) or 0.05 mL, 60 mg, 0.49 mmol, 4.9 equiv. (experiment D)) was added. The vial was sealed after purging with argon, and subsequently irradiated with blue LEDs (60 W, 450 nm, approx. 20 °C) for 2 h.

Workup and analysis: (all reactions)

The reaction mixtures were diluted with 3 mL EtOAc each, and washed each with 4 mL H_2O. The EtOAc layers were concentrated under reduced presssure, and dried in vacuo. The resulting residues were dissolved each in 1 mL $CDCl_3$, and 20 µL of CH_2Br_2 was added to each sample. The product was quantified by ^1H NMR.

Results:

a: α-Aminoalkyl radicals without $PhNO_2$: 61% product + 91% Thianthrene.

b: α-Aminoalkyl radicals with $PhNO_2$: < 1% product + Thianthrene not determined.

c: Photocatalysis without $PhNO_2$: 62% product + 89% Thianthrene.

d: Photocatalysis with $PhNO_2$: 10% product + 18% Thianthrene.

Table S2: Control Experiment.

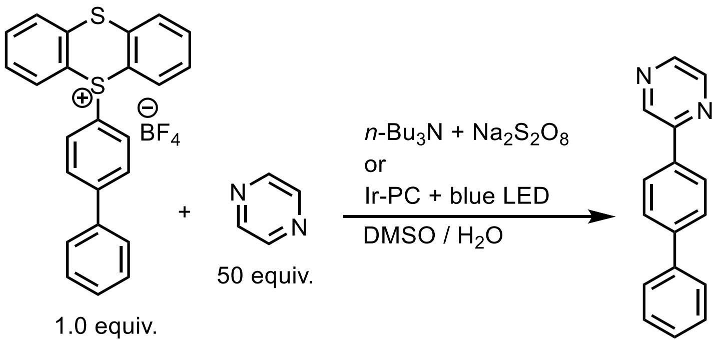

NMR yield	no additive	in presence of 3 equiv. $PhNO_2$
Photoredox	62%	10%
Amino alkyl radical	61%	<1%

C) Absence of light

A 25 mL round bottom flask equipped with a magnetic stir bar was charged with **TT-3** (130 mg, 0.285 mmol, 1.00 equiv.), $Na_2S_2O_8$ (0.203 g, 0.855 mmol, 3.00 equiv.), and pyrazine (1.14 g, 14.3 mmol, 50.0 equiv.). The round bottom flask was wrapped with aluminium foil. Subsequently, DMSO (2.50 mL), and H_2O (0.50 mL) were added, followed by *n*-Bu_3N (0.680 mL, 2.85 mmol, 10.0 equiv.). The round bottom flask was capped with a septum, and placed in a preheated oil bath at 80 °C for 18 h. After cooling to 25 °C, H_2O (15 mL) was added to the reaction mixture. The resulting mixture was transferred to a separatory funnel and extracted with EtOAc (3 × 15 mL). The combined organic layers were dried over $MgSO_4$, and concentrated under reduced pressure. The residue was purified by chromatography on silica gel eluting with with Et_3N/hexanes/EtOAc (3:100:0–3:100:20 (v/v/v)). The product-containing fractions were collected and concentrated under reduced pressure. The residue was further dried in vacuo to afford 45.4 mg (70%) of **3** as a light yellow solid.

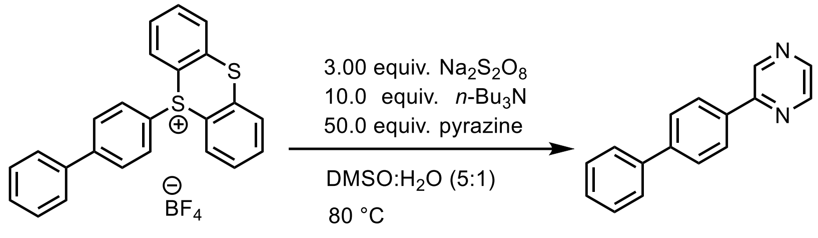

TT-3 **3**, 70%

D) Silyl radical-mediated halogen abstraction:

Under an ambient atmosphere, a 4 mL glass vial equipped with a magnetic stir bar was charged with 4-iodobiphenyl derived thianthrenium salt (**TT-11**) (20 mg, 0.03 mmol, 1.0 equiv.), $Na_2S_2O_8$ (20 mg, 0.08 mmol, 2.8 equiv.), pyrazine (0.13 g, 1.5 mmol, 50 equiv.), and tris-(trimethylsilyl)silanol (42 µL, 0.12 mmol, 4.5 equiv.). Subsequently, DMSO (0.06 mL), and H_2O (0.3 mL) were added. The vial was sealed without degassing, and subsequently placed in an aluminium heating block preheated to 80 °C for 12 h.

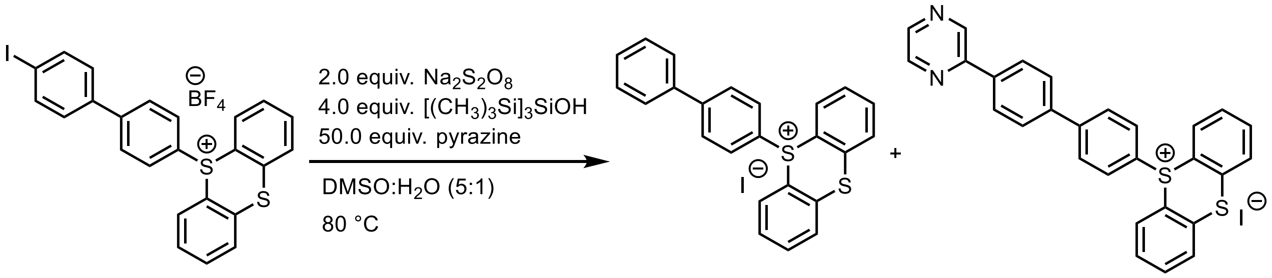

TT-11 **S-2** **S-3**

S-2

HRMS-ESI (m/z) calc'd for $C_{24}H_{17}S_2^+$ [M- I]$^+$, 369.0768; found, 369.0766; deviation: – 0.35 ppm.

S-3

HRMS-ESI (m/z) calc'd for $C_{28}H_{19}N_2S_2^+$ [M- I]$^+$, 447.0983; found, 447.0984; deviation: + 0.31 ppm.

Table S3: Investigation of halogen-atom transfer (XAT) process by using silanes.

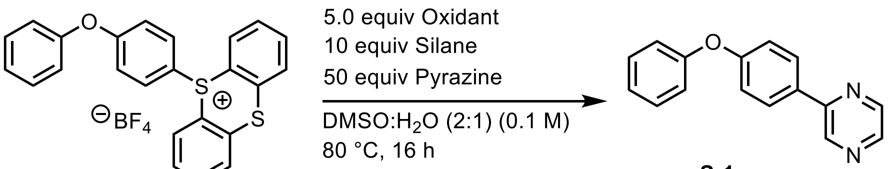

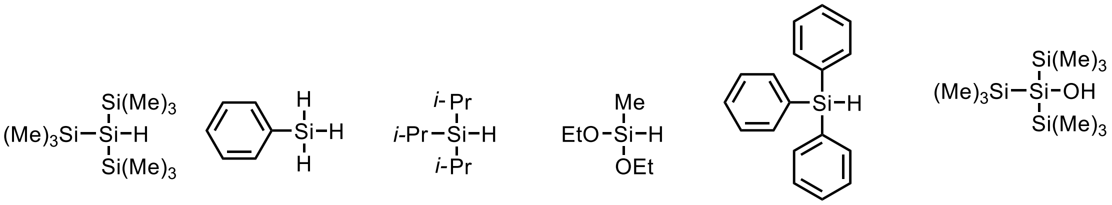

1 2 3 4 5 6

Entry	Silane	Yield(%)	
1	$[(CH_3)_3Si]_3SiH$	–	No C-S bond cleavage
2	$[(C_6H_5)]SiH_3$	–	
3	$[(CH_3)_2CH]_3SiH$	–	
4	$[(CH_3CH_2)O]SiCH_3$	–	
5	$[(C_6H_5)_3]SiH$	–	
6	$[(CH_3)_3Si]_3SiOH$	–	

Sulfonium salts synthesis

1-(4-Methoxyphenyl)ethan-1-one-derived thianthrenium tetrafluoroborate (TT-7)

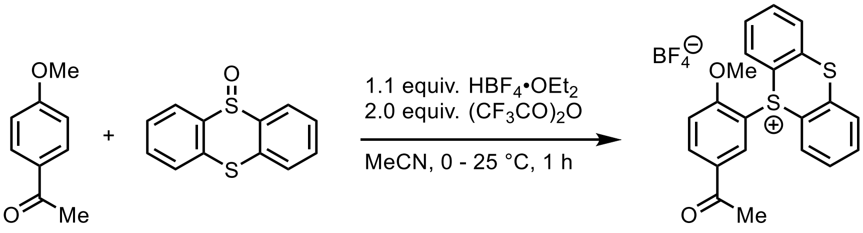

S1 **TT-7**, 87%

Under ambient atmosphere, a 20 mL glass-vial was charged with acetyl anisole (1.5 g, 10 mmol, 1.0 equiv.), thianthrene-*S*-oxide (**S1**) (2.3 g, 10 mmol, 1.0 equiv.), and MeCN (8.0 mL, c = 1.2 M). After cooling to 0 °C, trifluoroacetic anhydride (2.8 mL, 4.2 g, 20 mmol, 2.0 equiv.) was added, followed by dropwise addition of $HBF_4{\cdot}OEt_2$ (1.5 mL, 1.8 g, 11 mmol, 1.1 equiv.). The vial was sealed with a screw-cap, and the mixture was stirred at 0 °C for 5 min, followed by stirring at 25 °C over 1 h. The reaction mixture was concentrated under

reduced pressure, and subsequently diluted with DCM (20 mL) and washed with concentrated $Na_2S_2O_3$ solution (50 mL). The DCM layer was washed with aqueous $NaBF_4$ solution (2 × ca. 40 ml, 5% (w/w)). The organic phase was dried over $MgSO_4$, filtered and the solvent was removed under reduced pressure. The residue was purified by chromatography on silica gel eluting with DCM/MeOH (1:0–4:1 (v/v)) to afford 3.9 g (87%) of **TT-7** as a white solid.

R_f = 0.22 (DCM/*i*-PrOH, 9:1 (v/v)).

NMR Spectroscopy:

^{1}H NMR (500 MHz, DMSO-*d*6, 298 K, δ): 8.45 (dd, *J* = 8.0, 1.4 Hz, 2H), 8.29 (dd, *J* = 8.7, 2.0 Hz, 1H), 8.09 (dd, *J* = 8.0, 1.3 Hz, 2H), 7.92 (td, *J* = 7.7, 1.4 Hz, 2H), 7.84 (td, *J* = 7.7, 1.4 Hz, 2H), 7.45 (d, *J* = 8.8 Hz, 1H), 7.09 (d, *J* = 2.0 Hz, 1H), 4.05 (s, 3H), 2.44 (s, 3H).

^{13}C {^{1}H} NMR (126 MHz, DMSO-*d*6, 298 K, δ): 195.1, 160.3, 136.3, 136.0, 135.8, 134.7, 130.1, 129.9, 129.6, 128.2, 117.1, 114.2, 111.2, 57.7, 26.3.

^{19}F NMR (471 MHz, DMSO-*d*6, 298 K, δ): –148.19 (bs), –148.24 (bs).

HRMS-ESI(m/z) calc'd for $C_{21}H_{17}O_2S_2^+$ [M-BF4]+, 365.0666; found, 365.0664; deviation: – 0.41 ppm.

5-Methyl-2-nitroanisole-derived thianthrenium tetrafluoroborate (TT-16)

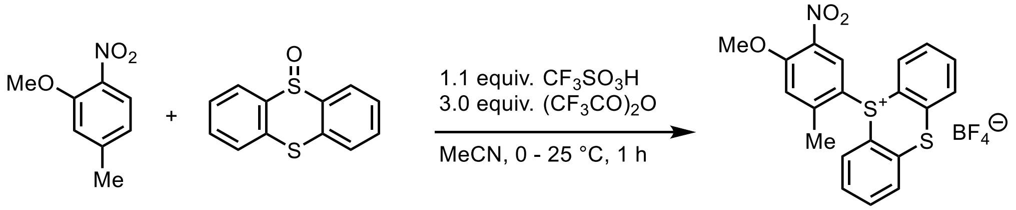

S1 **TT-16**, 80%

Under ambient atmosphere, a 20 mL glass-vial was charged with 5-methyl-2-nitroanisole (0.5 g, 3.0 mmol, 1.0 equiv.), thianthrene-*S*-oxide (**S1**) (0.7 g, 3.0 mmol, 1.0 equiv.), and MeCN (5.0 mL, c = 0.6 M). After cooling to 0 °C, trifluoroacetic anhydride (1.3 mL, 1.9 g, 9.0 mmol, 3.0 equiv.) was added, followed by dropwise addition of triflic acid (0.3 mL, 0.7 g, 3.3 mmol, 1.1 equiv.). The vial was sealed with a screw-cap, and the mixture was stirred at 0 °C for 5 min, followed by stirring at 25 °C over 1 h. The reaction mixture was concentrated under reduced pressure, and subsequently diluted with DCM (10 mL) and washed with saturated aqueous Na_2CO_3 solution (20 mL). The DCM layer was washed with aqueous $NaBF_4$ solution (2 × ca. 20 ml, 5% (w/w)). The organic phase was dried over $MgSO_4$, filtered and the solvent was removed under reduced pressure. The residue was purified by chromatography on silica gel eluting with DCM/MeOH (1:0–4:1 (v/v)) to afford 1.1 g (80%) of **TT-16** as a white solid.

R_f = 0.16 (DCM/*i*-PrOH, 9:1 (v/v)).

NMR Spectroscopy:

^{1}H NMR (500 MHz, DMSO-*d*6, 298 K, δ): 8.44 (dd, J = 7.9, 1.4 Hz, 2H), 8.13 (dd, J = 8.0, 1.3 Hz, 2H), 7.92

(td, J = 7.7, 1.4 Hz, 2H), 7.83 (td, J = 7.8, 1.4 Hz, 2H), 7.60 (s, 1H), 7.51 (s, 1H), 3.97 (s, 3H), 2.70 (s, 3H).

^{13}C {^{1}H} NMR (126 MHz, DMSO-*d*$_6$, 298 K, δ): 155.6, 147.0, 137.0, 135.8, 134.9, 134.7, 130.8, 129.9, 126.7, 119.2, 118.5, 112.8, 57.7, 20.5.

^{19}F NMR (471 MHz, DMSO-*d*$_6$, 298 K, δ): –148.20 (bs), –148.25 (bs).

HRMS-ESI(m/z) calc'd for $C_{20}H_{16}NO_3S_2^+$ [M-BF$_4$]$^+$, 382.0568; found, 382.0566; deviation: – 0.38 ppm.

2-Acetyl-1-methylpyrrole dibenzothiophenium tetrafluoroborate (TT-21)

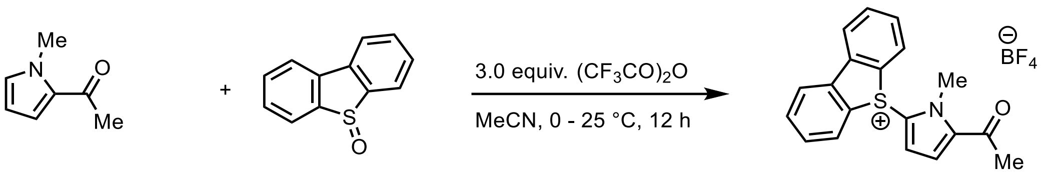

S2 **TT-21**, 23%

Under ambient atmosphere, a 20 mL glass vial equipped with a magnetic stir bar was charged with 2-acetyl-1-methylpyrrole (74 mg, 0.07 mL, 0.60 mmol, 1.0 equiv.), dibenzothiophene-*S*-oxide (**S2**) (0.12 g, 0.60 mmol, 1.0 equiv.) and MeCN (3.0 mL, c = 0.20 M). After all solid was dissolved, the mixture was cooled to –78 °C using a dry ice/acetone bath. Trifluoroacetic anhydride (0.25 mL, 0.38 g, 1.8 mmol, 3.0 equiv.) was added in one portion to the frozen reaction mixture. The reaction was allowed to warm at 25 °C overnight. The reaction mixture was diluted with DCM (6.0 mL) and washed with water (2 × 10 mL). The DCM layer was washed with aqueous NaBF$_4$ solution (2 × ca. 10 ml, 5% (w/w)). The organic phase was dried over MgSO$_4$, filtered and the solvent was removed under reduced pressure. The residue was purified by chromatography on silica gel eluting with EtOAc/DCM/*i*-PrOH (1:0:0–0:9:1 (v/v/v)) to afford 59 mg (23%) of **TT-21** as a green solid.

NMR Spectroscopy:

^{1}H NMR (500 MHz, CDCl$_3$, 298 K, δ): 8.27 (s, 1H), 8.14 (d, *J* = 9.9 Hz, 4H), 7.86 (t, *J* = 7.7 Hz, 2H), 7.67 (t, *J* = 7.2 Hz, 2H), 6.40 (s, 1H), 4.00 (s, 3H), 2.23 (s, 3H).

^{13}C NMR (126 MHz, CD$_3$CN, 298 K, δ): 189.6, 139.8, 138.2, 135.2, 135.1, 134.1, 132.5, 128.4, 125.4, 118.9, 100.9, 39.2, 27.7.

^{19}F NMR (471 MHz, CDCl$_3$, 298 K, δ): –150.17 (bs), –150.22 (bs).

HRMS-ESI (m/z) calc'd for $C_{19}H_{16}NOS^+$ [M-BF$_4$]$^+$, 306.0945; found, 306.0947; deviation: + 0.56 ppm.

Ethyl 3-(furan-2-yl)propanoate dibenzothiophenium trifluoroacetate (TT-25)

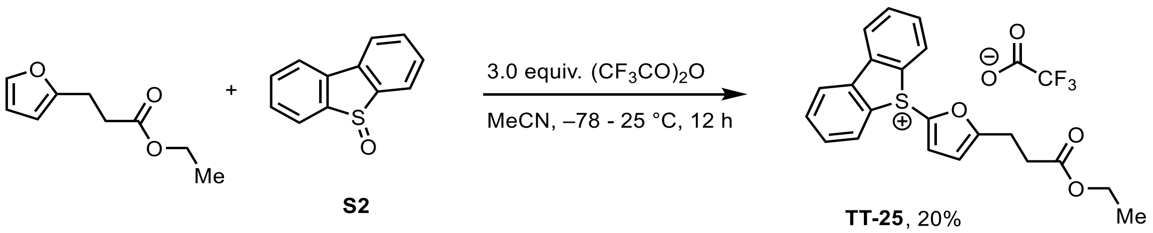

S2 **TT-25**, 20%

Under ambient atmosphere, a 50 mL round bottom flask equipped with a magnetic stir bar was charged with ethyl-3-(furan-2-yl)-propionate (0.96 mL, 1.0 g, 6.0 mmol, 1.0 equiv.), dibenzothiophene-*S*-oxide (**S2**) (1.2 g, 6.0 mmol, 1.0 equiv.) and MeCN (10 mL, c = 0.6 M). After all solid was dissolved, the mixture was cooled to –78 °C using a dry ice/acetone bath. Trifluoroacetic anhydride (2.5 mL, 3.8 g, 18 mmol, 3.0 equiv.) was added in one portion to the frozen reaction mixture. The reaction was allowed to warm at 25 °C and the reaction completion was monitored by TLC. The reaction mixture was diluted with DCM (20 mL) and washed with water (2 × 30 mL). The organic phase was dried over $MgSO_4$, filtered and the solvent was removed under reduced pressure. The residue was purified by chromatography on silica gel eluting with EtOAc/DCM/*i*-PrOH (1:0:0–0:9:1 (v/v/v)) to afford 0.54 g (20%) of **TT-25** as an off-white solid.

NMR Spectroscopy:

^{1}H NMR (500 MHz, $CDCl_3$, 298 K, δ): 8.36 – 8.29 (m, 3H), 8.10 (d, *J* = 7.9 Hz, 2H), 7.83 (t, *J* = 7.6 Hz, 2H), 7.65 (t, *J* = 7.7 Hz, 2H), 6.31 (d, *J* = 3.7 Hz, 1H), 4.01 (q, *J* = 7.1 Hz, 2H), 2.75 (t, *J* = 7.5 Hz, 2H), 2.36 (t, *J* = 7.5 Hz, 2H), 1.16 (t, *J* = 7.2 Hz, 3H).

^{13}C NMR (126 MHz, CD_3CN, 298 K, δ): 289.2, 284.3, 257.3, 252.5, 249.4, 248.6, 247.7, 245.8, 243.5, 242.3, 228.2, 178.3, 148.8, 141.2, 131.4.

^{19}F NMR (471 MHz, $CDCl_3$, 298 K, δ): –75.1 (s).

HRMS-ESI (m/z) calc'd for $C_{21}H_{19}O_3S^+$ [M- CO_2CF_3]$^+$, 351.1048; found, 351.1049; deviation: + 0.49 ppm.

1-Methyl pyrazole-derived dibenzothiophenium trifluoroacetate (TT-26)

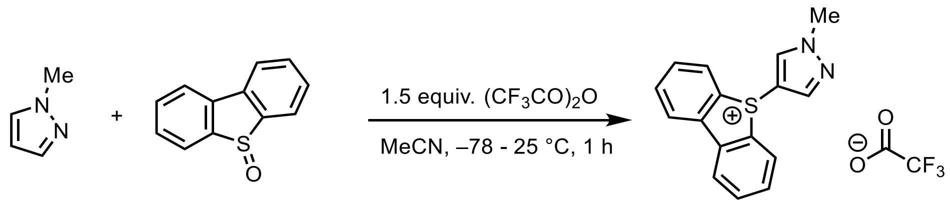

S2

TT-26, 23%

Under ambient atmosphere, a 50 mL round bottom flask equipped with a magnetic stir bar was charged with 1-methyl pyrazole (0.90 g, 11 mmol, 1.0 equiv.), dibenzothiophene-*S*-oxide (**S2**) (2.4 g, 10 mmol, 1.0 equiv.) and MeCN (15 mL, c = 0.67 M). After all solid was dissolved, the mixture was cooled to –78 °C using a dry ice/acetone bath. Subsequently, trifluoroacetic anhydride (2.1 mL, 3.2 g, 15 mmol, 1.5 equiv.) was added in one portion to the frozen reaction mixture. The dry ice/acetone bath was removed, and the reaction was allowed to warm at 25 °C over 1 h. The reaction mixture was diluted with DCM (30 mL) and washed with water (2 × 90 mL). The organic phase was dried over $MgSO_4$, filtered and the solvent was removed under reduced pressure. The residue was purified by chromatography on silica gel eluting with DCM/MeOH (1:0–4:1 (v/v)) to afford 0.87 g (23%) of **TT-26** as a colorless solid.

NMR Spectroscopy:

^{1}H NMR (500 MHz, $CDCl_3$, 298 K, δ): 9.47 (s, 1H), 8.29 (d, *J* = 7.9 Hz, 2H), 8.10 (d, *J* = 9.2 Hz, 2H), 7.83

(t, J = 8.1 Hz, 2H), 7.70 – 7.61 (m, 2H), 6.90 (s, 1H), 3.98 (s, 3H).

^{13}C NMR (126 MHz, $CDCl_3$, 298 K, δ): 140.3, 138.3, 137.9, 134.0, 133.4, 131.7, 128.8, 123.6, 100.7, 40.4.

^{19}F NMR (471 MHz, CD_3CN, 298 K, δ): –75.4 (s).

HRMS-ESI (m/z) calc'd for $C_{16}H_{13}N_2S^+$ [M-CO_2CF_3]$^+$, 265.0794; found, 265.0794; deviation: – 0.06 ppm.

Heteroarylation of (hetero)aryl sulfonium salts

Salicin pentaacetate *N*-methyl imidazole derivative 1a, 1b

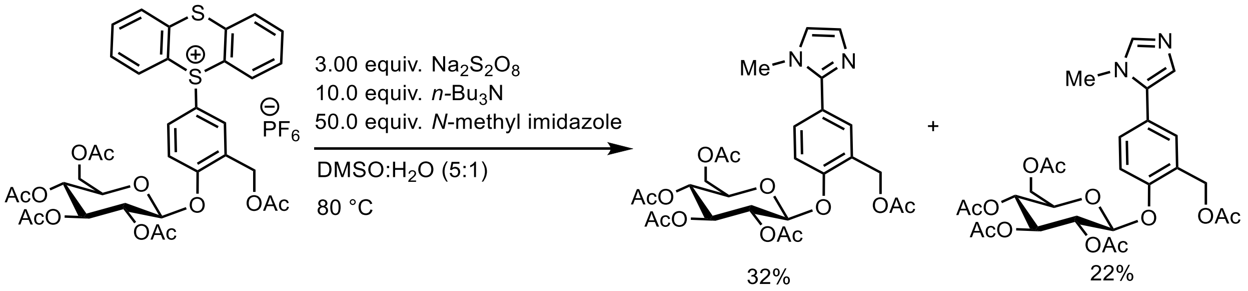

TT-1 **1a** **1b**

A 25 mL round bottom flask equipped with a magnetic stir bar was charged with **TT-1** (257 mg, 0.300 mmol, 1.00 equiv.), $Na_2S_2O_8$ (0.21 g, 0.90 mmol, 3.0 equiv.), and *N*-methyl imidazole (1.20 mL, 15.0 mmol, 50.0 equiv.). Subsequently, DMSO (2.5 mL), and H_2O (0.5 mL) were added, followed by *n*-Bu_3N (0.72 mL, 3.0 mmol, 10 equiv.). The round bottom flask was capped with a septum, and placed in a preheated oil bath at 80 °C for 12 h. After cooling to 25 °C, H_2O (15 mL) was added to the reaction mixture. The resulting mixture was transferred to a separatory funnel and extracted with EtOAc (3 × 15 mL). The combined organic layers were dried over $MgSO_4$, and concentrated under reduced pressure. The residue was purified by chromatography on silica gel eluting with Et_3N/EtOAc (3:100 (v/v)). The product-containing fractions were collected and concentrated under reduced pressure. The residue was further dried in vacuo to afford 55.6 mg (32%) of **1a** as a white solid and 38.6 mg (22%) of **1b** as a colorless solid.

Data for 1a:

R_f = 0.40 (Et_3N/EtOAc, 3:100 (v/v)).

NMR Spectroscopy:

^{1}H NMR (500 MHz, $CDCl_3$, 298 K, δ): 7.59 (d, J = 2.0 Hz, 1H), 7.53 (dd, J = 8.5, 2.0 Hz, 1H), 7.13 (d, J = 8.7 Hz, 1H), 7.07 (dq, J = 2.8, 1.3 Hz, 1H), 6.94 (q, J = 1.6 Hz, 1H), 5.33 – 5.26 (m, 2H), 5.20 – 5.13 (m, 1H), 5.12 (d, J = 7.2 Hz, 2H), 5.06 (d, J = 13.1 Hz, 1H), 4.27 (dd, J = 12.4, 5.3 Hz, 1H), 4.18 (dd, J = 12.2, 2.0 Hz, 1H), 3.93 – 3.86 (m, 1H), 3.69 (s, 3H), 2.11 – 2.00 (m, 15H).

^{13}C NMR (126 MHz, $CDCl_3$, 298 K, δ): 170.8, 170.6, 170.3, 169.5, 169.4, 154.9, 146.8, 130.1, 127.9, 126.5, 125.4, 122.5, 115.7, 99.1, 72.6, 72.2, 71.0, 68.3, 62.0, 60.8, 34.6, 21.0, 20.8, 20.7.

HRMS-ESI (m/z) calc'd for $C_{27}H_{32}N_2O_{12}Na^+$ [M+Na]$^+$, 599.1849; found, 599.1848; deviation: – 0.32 ppm.

Data for 1b:

R*f* = 0.25 (MeOH/EtOAc, 5:100 (v/v)).

NMR Spectroscopy:

^{1}H NMR (500 MHz, $CDCl_3$, 298 K, δ): 7.85 (s, 1H), 7.35 (d, *J* = 2.2 Hz, 1H), 7.29 (dd, *J* = 8.4, 2.3 Hz, 1H), 7.14 (d, *J* = 8.4 Hz, 1H), 7.08 (s, 1H), 5.35 – 5.26 (m, 2H), 5.22 – 5.10 (m, 3H), 5.06 (d, *J* = 13.1 Hz, 1H), 4.28 (dd, *J* = 12.4, 5.2 Hz, 1H), 4.20 (dd, *J* = 12.4, 2.6 Hz, 1H), 3.89 (ddd, *J* = 10.1, 5.2, 2.5 Hz, 1H), 3.67 (s, 3H), 2.15 – 1.97 (m, 15H).

^{13}C NMR (126 MHz, $CDCl_3$, 298 K, δ): 170.7, 170.6, 170.3, 169.5, 169.4, 154.5, 132.9, 129.9, 129.9, 127.0, 126.6, 124.4, 116.0, 99.2, 72.6, 72.3, 71.0, 68.3, 62.0, 60.8, 32.9, 21.0, 20.8, 20.7.

HRMS-ESI (m/z) calc'd for $C_{27}H_{33}N_2O_{12}^+$ $[M+H]^+$, 577.2031; found, 577.2028; deviation: – 0.53 ppm.

8-Butoxy-5-(pyrazin-2-yl)quinoline (2)

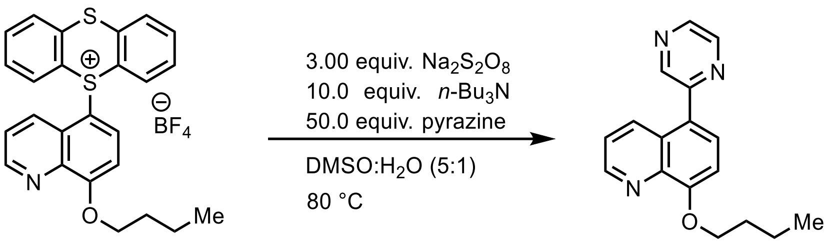

A 25 mL round bottom flask equipped with a magnetic stir bar was charged with **TT-2** (151 mg, 0.300 mmol, 1.00 equiv.), $Na_2S_2O_8$ (0.21 g, 0.90 mmol, 3.0 equiv.), and pyrazine (1.20 g, 15.0 mmol, 50.0 equiv.). Subsequently, DMSO (2.5 mL), and H_2O (0.5 mL) were added, followed by *n*-Bu_3N (0.72 mL, 3.0 mmol, 10 equiv.). The round bottom flask was capped, and placed in a preheated oil bath at 80 °C for 12 h. After cooling to 25 °C, an aqueous solution of tartaric acid (15% (w/w), 15 mL) was added to the reaction mixture. The resulting mixture was transferred to a separatory funnel and extracted with EtOAc (3 × 15 mL). The combined organic layers were dried over $MgSO_4$, and concentrated under reduced pressure. The residue was purified by chromatography on silica gel eluting with hexanes/EtOAc (1:0–5:1 (v/v)). The product-containing fractions were collected and concentrated under reduced pressure. The residue was further dried in vacuo to afford 51.5 mg (61%) of **2** as a light yellow solid.

R*f* = 0.15 (hexanes/EtOAc, 1:1 (v/v)).

NMR Spectroscopy:

^{1}H NMR (500 MHz, $CDCl_3$, 298 K, δ): 9.01 (d, *J* = 4.1 Hz, 1H), 8.87 (d, *J* = 1.7 Hz, 1H), 8.74 – 8.70 (m, 1H), 8.63 (d, *J* = 8.5 Hz, 1H), 8.59 (d, *J* = 2.6 Hz, 1H), 7.67 (d, *J* = 8.1 Hz, 1H), 7.48 (dd, *J* = 8.6, 4.2 Hz, 1H), 7.17 (d, *J* = 8.1 Hz, 1H), 4.31 (t, *J* = 7.0 Hz, 2H), 2.05 (p, *J* = 7.1 Hz, 2H), 1.59 (q, *J* = 7.5 Hz, 2H), 1.02 (t, *J* = 7.4 Hz, 3H).

^{13}C NMR (126 MHz, $CDCl_3$, 298 K, δ): 156.2, 154.2, 149.5, 145.7, 143.9, 142.7, 140.5, 134.0, 129.2,

127.5, 126.3, 122.3, 108.2, 69.1, 31.0, 19.4, 14.0.

HRMS-EI (m/z) calc'd for $C_{17}H_{17}N_3O^+$ [M]$^+$, 279.1376; found, 279.1372; deviation: – 1.65 ppm.

2-([1,1'-Biphenyl]-4-yl)pyrazine (3)

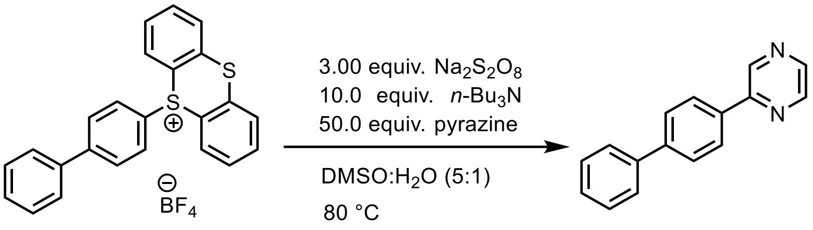

TT-3 **3**, 77%

A 25 mL round bottom flask equipped with a magnetic stir bar was charged with **TT-3** (130 mg, 0.285 mmol, 1.00 equiv.), $Na_2S_2O_8$ (0.203 g, 0.855 mmol, 3.00 equiv.), and pyrazine (1.14 g, 14.3 mmol, 50.0 equiv.). Subsequently, DMSO (2.50 mL), and H_2O (0.50 mL) were added, followed by *n*-Bu_3N (0.680 mL, 2.85 mmol, 10.0 equiv.). The round bottom flask was capped with a septum, and placed in a preheated oil bath at 80 °C for 12 h. After cooling to 25 °C, an aqueous solution of tartaric acid (10% (w/w), 15 mL) was added to the reaction mixture. The resulting mixture was transferred to a separatory funnel and extracted with EtOAc (3 × 15 mL). The combined organic layers were dried over $MgSO_4$, and concentrated under reduced pressure. The residue was purified by chromatography on silica gel eluting with hexanes/EtOAc (1:0–5:1 (v/v)). The product-containing fractions were collected and concentrated under reduced pressure. The residue was further dried in vacuo to afford 50.7 mg (77%) of **3** as a light yellow solid.

R_f = 0.50 (hexanes/EtOAc, 1:1 (v/v)).

NMR Spectroscopy:

^{1}H NMR (500 MHz, $CDCl_3$, 298 K, δ): 9.09 (s, 1H), 8.66 (d, *J* = 2.1 Hz, 1H), 8.52 (s, 1H), 8.11 (d, *J* = 8.5 Hz, 2H), 7.76 (d, *J* = 8.5 Hz, 2H), 7.69 – 7.64 (m, 2H), 7.48 (dd, *J* = 8.4, 6.9 Hz, 2H), 7.42 – 7.35 (m, 1H).

^{13}C NMR (126 MHz, $CDCl_3$, 298 K, δ): 152.6, 144.4, 143.0, 142.9, 142.3, 140.4, 135.3, 129.0, 127.9, 127.9, 127.5, 127.3.

HRMS-EI (m/z) calc'd for $C_{16}H_{12}N_2$ [M]$^+$, 232.0996; found, 232.0995; deviation: – 0.44 ppm.

1-Bromo-4-phenoxybenzene pyrimidine derivative 4a, 4b, 4c

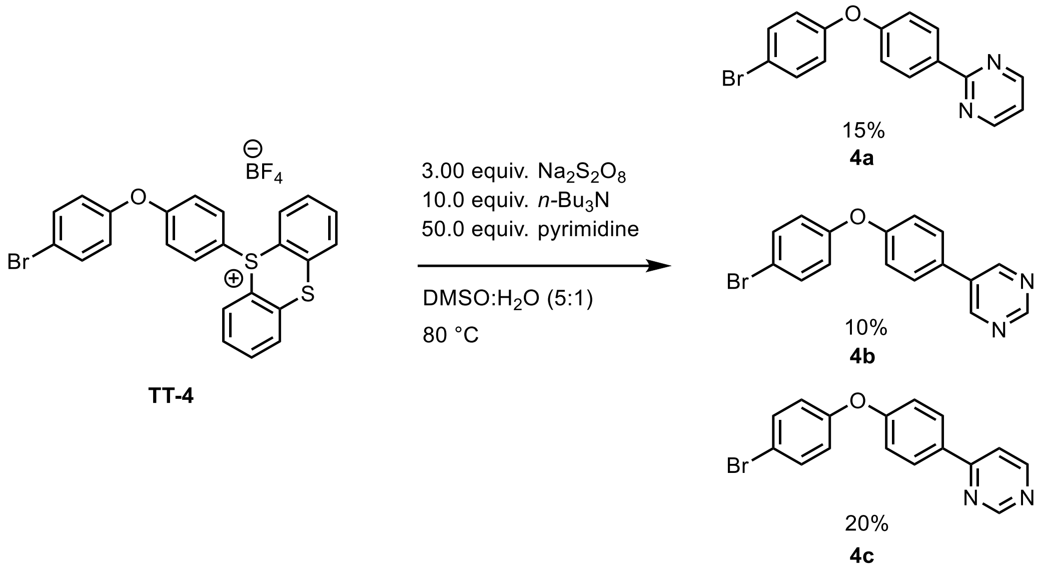

A 25 mL round bottom flask equipped with a magnetic stir bar was charged with **TT-4** (165 mg, 0.300 mmol, 1.00 equiv.), $Na_2S_2O_8$ (0.21 g, 0.90 mmol, 3.0 equiv.), and pyrimidine (1.2 mL, 15 mmol, 50 equiv.). Subsequently, DMSO (2.5 mL), and H_2O (0.50 mL) were added, followed by *n*-Bu_3N (0.71 mL, 3.0 mmol, 10 equiv.). The round bottom flask was capped with a ground glass stopper and placed in an oil bath at 80 °C for 12 h. After cooling to 25 °C, H_2O (15 mL) was added to the reaction mixture. The resulting mixture was transferred to a separatory funnel and extracted with EtOAc (2 × 20 mL). The combined organic layers were dried over $MgSO_4$, and concentrated under reduced pressure. The residue was purified by chromatography on silica gel eluting with Et_3N/EtOAc/hexanes (3:0:100–3:18:100 (v/v/v)) to afford 15 mg (15%) of **4a** as a colorless solid, 10 mg (10%) of **4b** as a colorless solid and 20 mg (20%) of **4c** as a light yellow solid. A sample of the C2-isomer was further purified by column chromatography on silica gel eluting with DCM, which was used for the acquisition of the ^{1}H NMR spectrum.

Data for 4a:

R_f = 0.64 (EtOAc/DCM, 1:5 (v/v)).

NMR Spectroscopy: 4a (15%)

^{1}H NMR (500 MHz, $CDCl_3$, 298 K, δ): 8.78 (d, *J* = 4.9 Hz, 2H), 8.43 (d, *J* = 9.0 Hz, 2H), 7.47 (d, *J* = 9.0 Hz, 2H), 7.17 (t, *J* = 4.8 Hz, 1H), 7.08 (d, *J* = 9.0 Hz, 2H), 6.96 (d, *J* = 8.9 Hz, 2H).

^{13}C NMR (151 MHz, $CDCl_3$, 298 K, δ): 164.2, 159.5, 157.4, 155.9, 133.0, 133.0, 130.2, 121.3, 119.0, 118.6, 116.5.

HRMS-ESI (m/z) calc'd for $C_{16}H_{12}BrN_2O^+$ $[M+H]^+$, 327.0128; found, 327.0128; deviation: – 0.02 ppm.

Data for 4b:

R_f = 0.52 (EtOAc/hexanes, 1:5 (v/v)).

NMR Spectroscopy: 4b (10%)

^{1}H NMR (600 MHz, $CDCl_3$, 298 K, δ): 9.19 (s, 1H), 8.93 (s, 2H), 7.55 (d, *J* = 8.7 Hz, 2H), 7.48 (d, *J* = 9.0 Hz, 2H), 7.13 (d, *J* = 8.7 Hz, 2H), 6.95 (d, *J* = 9.0 Hz, 2H).

^{13}C NMR (151 MHz, $CDCl_3$, 298 K, δ): 158.2, 157.5, 155.8, 154.8, 133.8, 133.1, 129.5, 128.7, 121.2, 119.6, 116.7.

HRMS-ESI (m/z) calc'd for $C_{16}H_{12}BrN_2O^+$ [M+H]$^+$, 327.0127; found, 327.0128; deviation: + 0.16 ppm.

Data for 4c:

R_f = 0.58 (EtOAc/hexanes, 1:1 (v/v)).

NMR Spectroscopy: 4c (20%)

^{1}H NMR (500 MHz, $CDCl_3$, 298 K, δ): 9.23 (s, 1H), 8.74 (d, *J* = 5.3 Hz, 1H), 8.09 (d, *J* = 8.9 Hz, 2H), 7.67 (d, *J* = 5.3 Hz, 1H), 7.48 (d, *J* = 9.0 Hz, 2H), 7.09 (d, *J* = 8.9 Hz, 2H), 6.95 (d, *J* = 8.9 Hz, 2H).

^{13}C NMR (126 MHz, $CDCl_3$, 298 K, δ): 163.1, 159.9, 159.2, 157.5, 155.5, 133.1, 131.7, 129.1, 121.4, 118.8, 116.9, 116.6.

HRMS-ESI (m/z) calc'd for $C_{16}H_{12}BrN_2O^+$ [M+H]$^+$, 327.0126; found, 327.0128; deviation: + 0.47 ppm.

Boscalid pyrazine derivative (5)

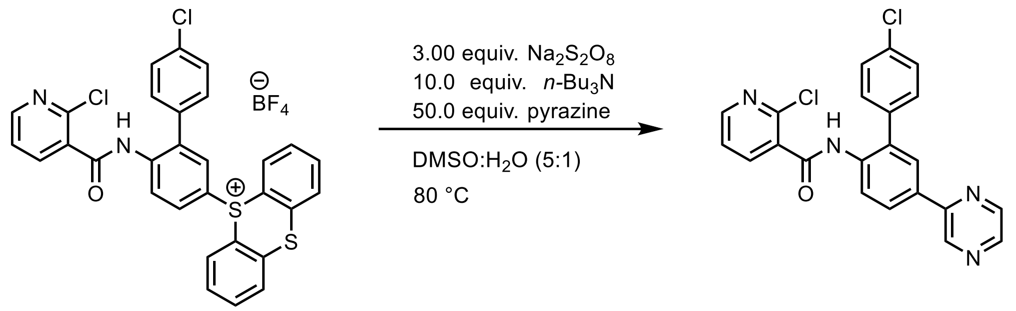

TT-5 **5,** 55%

A 25 mL round bottom flask equipped with a magnetic stir bar was charged with **TT-5** (193 mg, 0.300 mmol, 1.00 equiv.), $Na_2S_2O_8$ (0.21 g, 0.87 mmol, 3.0 equiv.), and pyrazine (1.20 g, 15.0 mmol, 50.0 equiv.). Subsequently, DMSO (2.5 mL), and H_2O (0.5 mL) were added, followed by *n*-Bu_3N (0.72 mL, 3.0 mmol, 10 equiv.). The round bottom flask was capped, and placed in a preheated oil bath at 80 °C for 12 h. After cooling to 25 °C, an aqueous solution of tartaric acid (15% (w/w), 15 mL) was added to the reaction mixture. The resulting mixture was transferred to a separatory funnel and extracted with EtOAc (3 × 15 mL). The combined organic layers were dried over $MgSO_4$, and concentrated under reduced pressure. The residue was purified by chromatography on silica gel eluting with hexanes/EtOAc (20:3–20:15 (v/v)). The product-containing fractions were collected and concentrated under reduced pressure. The residue was further dried in vacuo to afford 68.8 mg (55%) of **5** as a light yellow solid.

R_f = 0.12 (hexanes/EtOAc, 1:1 (v/v)).

NMR Spectroscopy:

^{1}H NMR (500 MHz, $CDCl_3$, 298 K, δ): 9.06 (d, *J* = 1.7 Hz, 1H), 8.69 (d, *J* = 8.7 Hz, 1H), 8.63 (dd, *J* = 2.5, 1.6 Hz, 1H), 8.52 (d, *J* = 2.4 Hz, 1H), 8.48 (dd, *J* = 4.7, 2.0 Hz, 1H), 8.36 (s, 1H), 8.21 (dd, *J* = 7.7, 2.1 Hz, 1H), 8.11 (dd, *J* = 8.7, 2.3 Hz, 1H), 7.98 (d, *J* = 2.1 Hz, 1H), 7.48 (d, *J* = 8.5 Hz, 2H), 7.40 (d, *J* = 8.5 Hz, 2H), 7.39 – 7.36 (m, 1H).

^{13}C NMR (151 MHz, $CDCl_3$, 298 K, δ): 162.6, 151.9, 151.7, 146.7, 144.4, 143.1, 142.1, 140.7, 136.3, 135.7, 135.1, 132.9, 132.6, 131.0, 130.9, 129.7, 128.9, 127.4, 123.2, 122.0.

HRMS-EI (m/z) calc'd for $C_{22}H_{14}Cl_2ON_4$ $[M]^+$, 420.0549; found, 420.0545; deviation: – 0.91 ppm.

1-Bromo-2-butoxynaphthalene pyridine derivative 6a, 6b, 6c

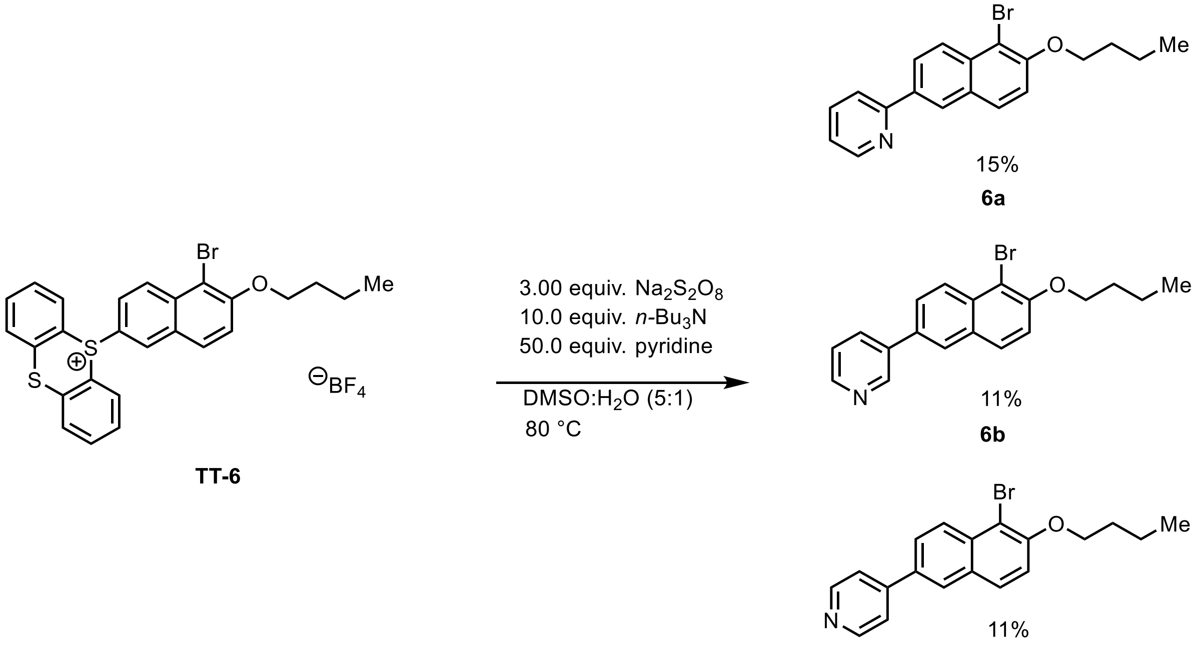

TT-6 6a 6b 6c

A 25 mL round bottom flask equipped with a magnetic stir bar was charged with **TT-6** (186 mg, 0.320 mmol, 1.00 equiv.), $Na_2S_2O_8$ (0.23 g, 0.96 mmol, 3.0 equiv.), and pyridine (1.28 mL, 16.0 mmol, 50.0 equiv.). Subsequently, DMSO (2.5 mL), and H_2O (0.50 mL) were added, followed by *n*-Bu_3N (0.76 mL, 3.2 mmol, 10 equiv.). The round bottom flask was capped with a ground glass stopper and placed in an oil bath at 80 °C for 12 h. After cooling to 25 °C, H_2O (15 mL) was added to the reaction mixture. The resulting mixture was transferred to a separatory funnel and extracted with EtOAc (2 × 20 mL). The combined organic layers were dried over $MgSO_4$, and concentrated under reduced pressure. The residue was purified by chromatography on silica gel eluting with Et_3N/EtOAc/hexanes (3:0:100–3:30:100 (v/v/v)) to afford 16.6 mg (15%) of **6a** as a yellow solid, 12.9 mg (11%) of **6b** as a colorless solid and 12.1 mg (11%) of **6c** as a colorless solid.

Data for 6a:

R_f = 0.31 (EtOAc/hexanes, 20:100 (v/v)). The TLC plate was treated with a solution of 3% Et_3N in EtOAc and

subsequently dried at ambient conditions.

NMR Spectroscopy:

^{1}H NMR (300 MHz, $CDCl_3$, 298 K, δ): 8.71 (ddd, *J* = 4.9, 1.8, 1.0 Hz, 1H), 8.43 (d, *J* = 1.9 Hz, 1H), 8.27 (d, *J* = 8.9 Hz, 1H), 8.14 (dd, *J* = 8.9, 1.8 Hz, 1H), 7.89 – 7.80 (m, 2H), 7.76 (ddd, *J* = 8.0, 7.2, 1.8 Hz, 1H), 7.25 – 7.20 (m, 2H), 4.17 (t, *J* = 6.4 Hz, 2H), 1.91 – 1.76 (m, 2H), 1.65 – 1.50 (m, 2H), 0.99 (t, *J* = 7.4 Hz, 3H).

^{13}C NMR (126 MHz, $CDCl_3$, 298 K, δ): 157.0, 154.1, 149.9, 137.1, 135.1, 133.7, 130.0, 129.8, 127.0, 126.6, 126.3, 122.3, 120.8, 115.6, 109.4, 70.0, 31.6, 19.4, 14.0.

HRMS-ESI (m/z) calc'd for $C_{19}H_{19}BrNO^+$ $[M+H]^+$, 356.0648; found, 356.0645; deviation: – 0.86 ppm.

Data for 6b:

R_f = 0.36 (EtOAc/hexanes, 50:100 (v/v)). The TLC plate was treated with a solution of 3% Et_3N in EtOAc and subsequently dried at ambient conditions.

NMR Spectroscopy:

^{1}H NMR (300 MHz, $CDCl_3$, 298 K, δ): 8.99 (s, 1H), 8.63 (s, 1H), 8.33 (d, *J* = 8.8 Hz, 1H), 8.03 (d, *J* = 7.9 Hz, 1H), 7.98 (d, *J* = 1.8 Hz, 1H), 7.87 (d, *J* = 9.0 Hz, 1H), 7.78 (dd, *J* = 8.9, 1.9 Hz, 1H), 7.50 – 7.40 (m, 1H), 7.31 (d, *J* = 9.0 Hz, 1H), 4.21 (t, *J* = 6.4 Hz, 2H), 1.96 – 1.82 (m, 2H), 1.69 – 1.54 (m, 2H), 1.02 (t, *J* = 7.4 Hz, 3H).

^{13}C NMR (126 MHz, $CDCl_3$, 298 K, δ): 154.1, 148.1, 148.1, 136.5, 135.0, 133.4, 133.0, 130.0, 129.3, 127.5, 126.7, 126.3, 124.0, 115.9, 109.4, 70.1, 31.6, 19.4, 14.0.

HRMS-ESI (m/z) calc'd for $C_{19}H_{19}BrNO^+$ $[M+H]^+$, 356.0647; found, 356.0645; deviation: – 0.66 ppm.

Data for 6c:

R_f = 0.19 (EtOAc/hexanes, 1:1 (v/v)).

NMR Spectroscopy:

^{1}H NMR (600 MHz, $CDCl_3$, 298 K, δ): 8.72 (s, 2H), 8.34 (d, *J* = 8.9 Hz, 1H), 8.08 (d, *J* = 1.9 Hz, 1H), 7.89 (d, *J* = 8.8 Hz, 1H), 7.83 (dd, *J* = 8.8, 1.9 Hz, 1H), 7.70 (d, *J* = 6.2 Hz, 2H), 7.33 (d, *J* = 9.0 Hz, 1H), 4.22 (t, *J* = 6.5 Hz, 2H), 1.97 – 1.84 (m, 2H), 1.61 (dq, *J* = 14.9, 7.4 Hz, 2H), 1.02 (t, *J* = 7.4 Hz, 3H).

^{13}C NMR (151 MHz, $CDCl_3$, 298 K, δ): 154.5, 149.4, 149.0, 133.7, 133.3, 129.8, 129.6, 127.7, 126.9, 126.2, 122.1, 115.9, 109.3, 70.0, 31.6, 19.4, 14.0.

HRMS-ESI (m/z) calc'd for $C_{19}H_{19}BrNO^+$ $[M+H]^+$, 356.0644; found, 356.0645; deviation: + 0.18 ppm.

1-(4-Methoxy-3-(pyrazin-2-yl)phenyl)ethan-1-one (7)

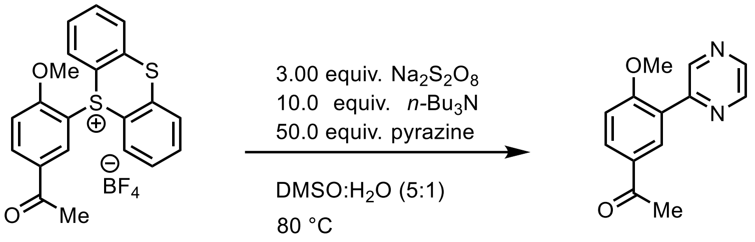

TT-7 **7**, 49%

A 25 mL round bottom flask equipped with a magnetic stir bar was charged with **TT-7** (136 mg, 0.300 mmol, 1.00 equiv.), $Na_2S_2O_8$ (2.1 g, 0.90 mmol, 3.0 equiv.), and pyrazine (1.20 g, 15.0 mmol, 50.0 equiv.). Subsequently, DMSO (2.5 mL), and H_2O (0.5 mL) were added, followed by *n*-Bu_3N (0.71 mL, 3.0 mmol, 10 equiv.). The round bottom flask was capped with a septum, and placed in an oil bath at 80 °C for 20 h. After cooling to 25 °C, an aqueous solution of citric acid (15% (w/w), 15 mL) was added to the reaction mixture. The resulting mixture was transferred to a separatory funnel and extracted with EtOAc (3 × 15 mL). The combined organic layers were dried over $MgSO_4$, and concentrated under reduced pressure. The residue was purified by chromatography on silica gel eluting with hexanes/EtOAc (1:0–3:1 (v/v)). The product-containing fractions were collected and concentrated under reduced pressure. The residue was further dried in vacuo to afford 33.3 mg (49%) of **7** as a light yellow solid.

R_f = 0.21 (hexanes/EtOAc, 1:1 (v/v)).

NMR Spectroscopy:

^{1}H NMR (500 MHz, $CDCl_3$, 298 K, δ): 9.15 (s, 1H), 8.68 (dd, *J* = 2.5, 1.6 Hz, 1H), 8.51 (d, *J* = 2.5 Hz, 1H), 8.46 (d, *J* = 2.3 Hz, 1H), 8.09 (dd, *J* = 8.7, 2.3 Hz, 1H), 7.09 (d, *J* = 8.8 Hz, 1H), 3.98 (s, 3H), 2.62 (s, 3H).

^{13}C NMR (75 MHz, $CDCl_3$, 298 K, δ): 196.7, 160.9, 151.1, 146.4, 144.4, 142.8, 132.4, 131.7, 131.0, 125.8, 111.4, 56.1, 26.6.

HRMS-EI (m/z) calc'd for $C_{13}H_{12}N_2O_2Na^+$ $[M+Na]^+$, 251.0793; found, 251.0797; deviation: + 1.38 ppm.

Pyriproxyfen pyrazine derivative (8)

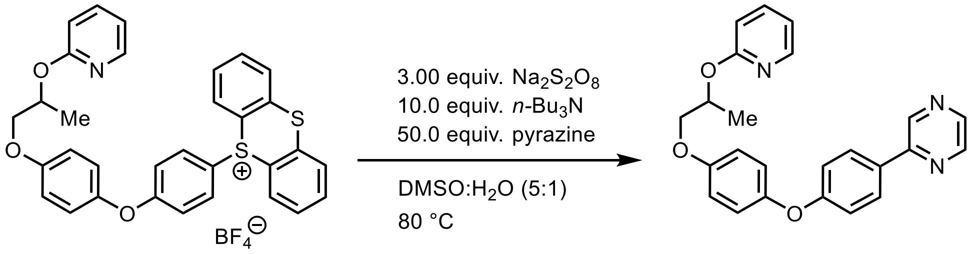

TT-8 **8**, 68%

A 25 mL round bottom flask equipped with a magnetic stir bar was charged with **TT-8** (187 mg, 0.300 mmol, 1.00 equiv.), $Na_2S_2O_8$ (0.21 g, 0.90 mmol, 3.0 equiv.), pyrazine (1.20 g, 15.0 mmol, 50.0 equiv.). Subsequently, DMSO (2.5 mL), and H_2O (0.5 mL) were added, followed by *n*-Bu_3N (0.71 mL, 3.0 mmol, 10 equiv.). The round

bottom flask was capped with a septum, and placed in an oil bath at 80 °C for 20 h. After cooling to 25 °C, aqueous solution of tartaric acid (15% (w/w), 15 mL) was added to the reaction mixture. The resulting mixture was transferred to a separatory funnel and extracted with EtOAc (3 × 15 mL). The combined organic layers were dried over $MgSO_4$, and concentrated under reduced pressure. The residue was purified by chromatography on silica gel eluting with hexanes/EtOAc (1:0–5:1 (v/v)) to afford 81 mg (68%) of **8** as a light yellow solid.

R_f = 0.52 (hexanes/EtOAc, 1:1 (v/v)).

NMR Spectroscopy:

^{1}H NMR (500 MHz, $CDCl_3$, 298 K, δ): 8.98 (d, *J* = 1.5 Hz, 1H), 8.59 (dd, *J* = 2.6, 1.5 Hz, 1H), 8.46 (d, *J* = 2.4 Hz, 1H), 8.16 (ddd, *J* = 5.2, 2.0, 0.9 Hz, 1H), 7.96 (d, *J* = 8.9 Hz, 2H), 7.58 (tdd, *J* = 7.1, 2.1, 1.2 Hz, 1H), 7.05 (d, *J* = 8.9 Hz, 2H), 7.02 (d, *J* = 9.2 Hz, 2H), 6.96 (d, *J* = 9.2 Hz, 2H), 6.87 (dddd, *J* = 6.1, 5.0, 2.3, 1.2 Hz, 1H), 6.78 – 6.74 (m, 1H), 5.65 – 5.56 (m, 1H), 4.21 (dd, *J* = 9.9, 5.3 Hz, 1H), 4.10 (dd, *J* = 9.8, 4.8 Hz, 1H), 1.50 (d, *J* = 6.4 Hz, 3H).

^{13}C NMR (126 MHz, $CDCl_3$, 298 K, δ): 163.2, 160.6, 155.8, 152.5, 149.6, 146.8, 144.2, 142.5, 141.9, 138.9, 130.5, 128.6, 121.4, 117.8, 116.9, 116.1, 111.9, 71.2, 69.5, 17.1.

HRMS-ESI (m/z) calc'd for $C_{24}H_{22}N_3O_3^+$ $[M+H]^+$, 400.1656; found, 471.1656; deviation: – 0.16 ppm.

Boscalid pyrimidine derivative 9a, 9b

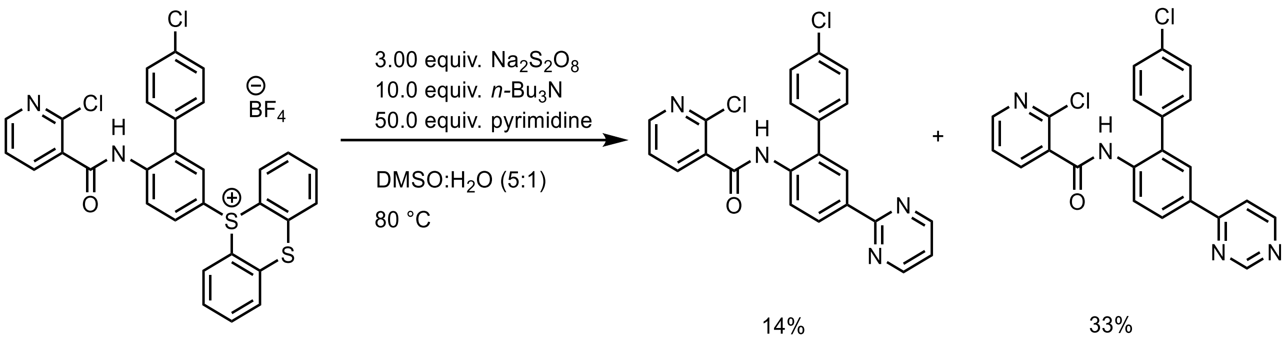

TT-5 **9a** **9b**

A 25 mL round bottom flask equipped with a magnetic stir bar was charged with **TT-5** (129 mg, 0.200 mmol, 1.00 equiv.), $Na_2S_2O_8$ (0.14 g, 0.60 mmol, 3.0 equiv.), and pyrimidine (0.790 mL, 10.0 mmol, 50.0 equiv.). Subsequently, DMSO (1.65 mL), and H_2O (0.33 mL) were added, followed by *n*-Bu_3N (0.48 mL, 2.0 mmol, 10 equiv.). The round bottom flask was capped with a septum, and placed in an oil bath at 80 °C for 20 h. After cooling to 25 °C, H_2O (15 mL) was added to the reaction mixture. The resulting mixture was transferred to a separatory funnel and extracted with EtOAc (3 × 15 mL). The combined organic layers were dried over $MgSO_4$, and concentrated under reduced pressure. The residue was purified by chromatography on silica gel eluting with Et_3N/EtOAc/hexanes (3:0:100–3:70:100 (v/v/v)) to afford 12.2 mg (14%) of **9a** as a colorless solid and 27.5 mg (33%) of **9b** as a light yellow solid.

Data for 9a:

R_f = 0.39 (hexanes/EtOAc, 2:3 (v/v)).

NMR Spectroscopy:

^{1}H NMR (500 MHz, $CDCl_3$, 298 K, δ): 8.80 (d, *J* = 4.7 Hz, 2H), 8.68 (d, *J* = 8.7 Hz, 1H), 8.55 (dd, *J* = 8.7 Hz, 2.1 Hz, 1H), 8.47 (dd, *J* = 4.7, 2.0 Hz, 1H), 8.38 (d, *J* = 2.1 Hz, 1H), 8.36 (s, 1H), 8.21 (d, *J* = 7.8 Hz, 1H), 7.47 (d, *J* = 8.2 Hz, 2H), 7.41 (d, *J* = 8.2 Hz, 2H), 7.38 (dd, *J* = 7.6, 4.7 Hz, 1H), 7.20 (t, *J* = 4.9 Hz, 1H).

^{13}C NMR (151 MHz, $CDCl_3$, 298 K, δ): 163.8, 162.5, 157.5, 151.7, 146.8, 140.6, 137.1, 136.0, 134.9, 133.9, 131.9, 131.1, 131.0, 130.2, 129.6, 129.1, 123.2, 121.4, 119.3.

HRMS-ESI (m/z) calc'd for $C_{22}H_{13}N_4OCl_2^+$ $[M-H]^+$, 419.0475; found, 419.0472; deviation: – 0.76 ppm.

Data for 9b:

R_f = 0.12 (DCM/EtOAc, 4:1 (v/v)).

NMR Spectroscopy:

^{1}H NMR (500 MHz, $CDCl_3$, 298 K, δ): 9.26 (s, 1H), 8.78 (d, *J* = 5.4 Hz, 1H), 8.73 (d, *J* = 8.7 Hz, 1H), 8.48 (dd, *J* = 4.7, 2.0 Hz, 1H), 8.42 (s, 1H), 8.22 (dd, *J* = 7.7, 2.1 Hz, 1H), 8.17 (dd, *J* = 8.7, 2.3 Hz, 1H), 8.09 (d, *J* = 2.3 Hz, 1H), 7.77 (d, *J* = 5.5 Hz, 1H), 7.49 (d, *J* = 8.4 Hz, 2H), 7.40 (d, *J* = 8.2, 2H), 7.37 (m, 1H).

^{13}C NMR (151 MHz, $CDCl_3$, 298 K, δ): 163.5, 162.6, 158.4, 156.7, 151.8, 146.7, 140.8, 137.8, 135.4, 135.3, 132.4, 132.4, 131.0, 130.7, 129.8, 129.4, 128.1, 123.2, 121.7, 116.9.

HRMS-ESI (m/z) calc'd for $C_{22}H_{13}N_4OCl_2^+$ $[M-H]^+$, 419.0476; found, 419.0472; deviation: – 0.93 ppm.

Bifonazole pyrazine derivative (10)

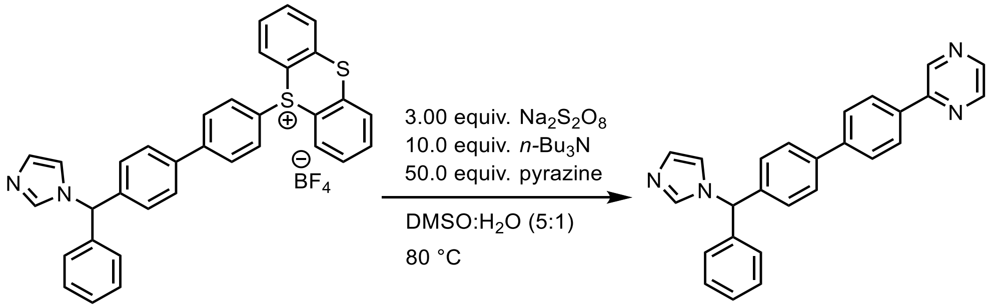

TT-10 **10**, 50%

A 25 mL round bottom flask equipped with a magnetic stir bar was charged with **TT-10** (120 mg, 0.196 mmol, 1.00 equiv.), $Na_2S_2O_8$ (0.14 g, 0.59 mmol, 3.0 equiv.), and pyrazine (0.784 g, 9.80 mmol, 50.0 equiv.). Subsequently, DMSO (1.6 mL), and H_2O (0.32 mL) were added, followed by *n*-Bu_3N (0.460 mL, 1.96 mmol, 10.0 equiv.). The round bottom flask was capped with a septum, and placed in an oil bath at 80 °C for 12 h. After cooling to 25 °C, H_2O (15 mL) was added to the reaction mixture. The resulting mixture was transferred to a separatory funnel and extracted with EtOAc (3 × 15 mL). The combined organic layers were dried over $MgSO_4$, and concentrated under reduced pressure. The residue was purified by chromatography on silica gel

eluting with Et_3N/EtOAc/hexanes (3:0:100–3:80:100 (v/v/v)) to afford 38 mg (50%) of **10** as a light yellow solid.

R_f = 0.81 (DCM/MeOH, 10:1 (v/v)).

NMR Spectroscopy:

^{1}H NMR (500 MHz, $CDCl_3$, 298 K, δ): 9.07 (d, *J* = 1.6 Hz, 1H), 8.65 (s, 1H), 8.52 (d, *J* = 2.4 Hz, 1H), 8.11 (d, *J* = 8.4 Hz, 2H), 7.73 (d, *J* = 8.4 Hz, 2H), 7.69 – 7.60 (m, 3H), 7.42 – 7.36 (m, 3H), 7.21 (d, *J* = 8.2 Hz, 2H), 7.19 – 7.14 (m, 3H), 6.93 (s, 1H), 6.63 (s, 1H).

^{13}C NMR (600 MHz, $CDCl_3$, 298 K, δ): 152.4, 144.4, 143.2, 142.3, 141.5, 141.3, 137.4, 137.0, 136.2, 135.9, 129.5, 129.4, 128.8, 128.2, 128.1, 127.9, 127.6, 125.1, 120.3, 66.2.

HRMS-ESI (m/z) calc'd for $C_{26}H_{21}N_4^+$ $[M+H]^+$, 389.1765; found, 389.1761; deviation: – 1.05 ppm.

1-(4-Methoxy-3-(1-methyl-1H-pyrrol-2-yl)phenyl)ethan-1-one (11)

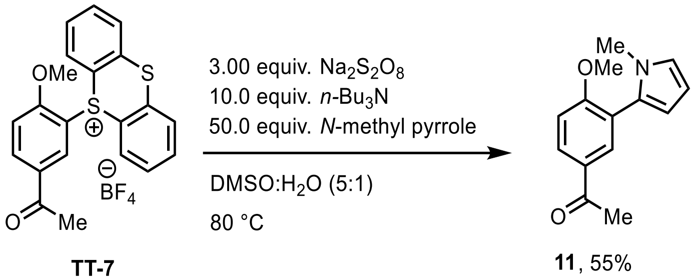

A 25 mL round bottom flask equipped with a magnetic stir bar was charged with **TT-7** (136 mg, 0.300 mmol, 1.00 equiv.), $Na_2S_2O_8$ (0.21 g, 0.90 mmol, 3.0 equiv.), and *N*-methyl pyrrole (1.33 mL, 15.0 mmol, 50.0 equiv.). Subsequently, DMSO (2.5 mL), and H_2O (0.5 mL) were added, followed by *n*-Bu_3N (0.71 mL, 3.0 mmol, 10 equiv.). The round bottom flask was capped with a septum, and placed in an oil bath at 80 °C for 20 h. After cooling to 25 °C, H_2O (15 mL) was added to the reaction mixture. The resulting mixture was transferred to a separatory funnel and extracted with EtOAc (3 × 15 mL). The combined organic layers were dried over $MgSO_4$, and concentrated under reduced pressure. The residue was purified by chromatography on silica gel eluting with hexanes/EtOAc (1:0–3:1 (v/v)) to afford 38 mg (55%) **11** as a light yellow solid.

R_f = 0.17 (hexanes/EtOAc, 5:1 (v/v)).

NMR Spectroscopy:

^{1}H NMR (500 MHz, $CDCl_3$, 298 K, δ): 8.01 (dd, *J* = 8.7, 2.3 Hz, 1H), 7.90 (d, *J* = 2.3 Hz, 1H), 7.00 (d, *J* = 8.7 Hz, 1H), 6.75 (dd, *J* = 2.7, 1.8 Hz, 1H), 6.24 (dd, *J* = 3.6, 2.7 Hz, 1H), 6.18 (dd, *J* = 3.5, 1.8 Hz, 1H), 3.89 (s, 3H), 3.48 (s, 3H), 2.57 (s, 3H).

^{13}C NMR (75 MHz, $CDCl_3$, 298 K, δ): 196.9, 161.4, 133.2, 130.3, 130.2, 130.1, 123.1, 122.7, 110.5, 109.6, 107.9, 55.9, 34.7, 26.5.

HRMS-GC-CI (m/z) calc'd for $C_{14}H_{15}NO_2{}^+$ $[M]^+$, 229.1097; found, 229.1097; deviation: – 0.05 ppm.

5-(4-(5-Acetyl-1-methyl-1H-pyrrol-2-yl)phenoxy)-2-fluorobenzonitrile (12)

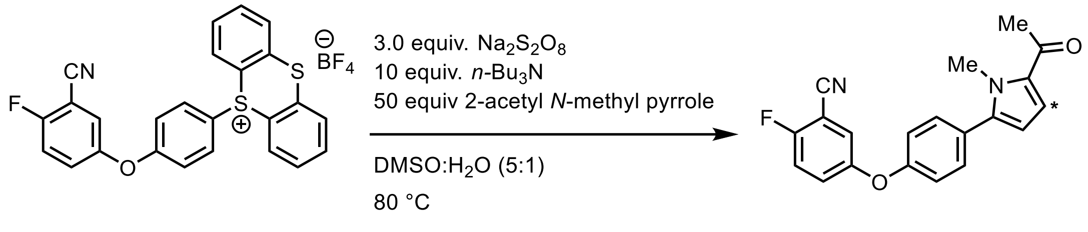

TT-12 **12**, 68%,10:1

A 25 mL round bottom flask equipped with a magnetic stir bar was charged with **TT-12** (155 mg, 0.300 mmol, 1.00 equiv.), $Na_2S_2O_8$ (0.21 g, 0.90 mmol, 3.0 equiv.), 2-acetyl *N*-methyl pyrrole (1.70 mL, 14.4 mmol, 48.0 equiv.). Subsequently, DMSO (2.5 mL), and H_2O (0.5 mL) were added followed by *n*-Bu_3N (0.71 mL, 3.0 mmol, 10 equiv.). The round bottom flask was capped with a ground glass stopper and placed in an oil bath at 80 °C for 12 h. After cooling to 25 °C, H_2O (15 mL) was added to the reaction mixture. The resulting mixture was transferred to a separatory funnel and extracted with EtOAc (2 × 20 mL). The combined organic layers were dried over $MgSO_4$, and concentrated under reduced pressure. The residue was purified by chromatography on silica gel eluting with Et_3N/EtOAc/hexanes (3:0:100–3:15:100 (v/v/v)) to afford 68 mg (68%) of **12** as a light yellow solid.

Data for major isomer 12:

$\mathbf{R_f}$ **=** 0.55 (EtOAc/hexanes, 1:1 (v/v)).

NMR Spectroscopy:

^{1}H NMR (500 MHz, $CDCl_3$, 298 K, δ): 7.54 – 7.42 (m, 3H), 7.18 (d, *J* = 8.8 Hz, 2H), 7.04 (d, *J* = 4.1 Hz, 1H), 6.94 (td, *J* = 8.4, 0.9 Hz, 1H), 6.74 (d, *J* = 8.5 Hz, 1H), 6.23 (d, *J* = 4.1 Hz, 1H), 3.90 (s, 3H), 2.48 (s, 3H).

^{13}C NMR (75 MHz, $CDCl_3$, 298 K, δ): 188.8, 164.3 (d, *J* = 260.1 Hz), 160.6 (d, *J* = 3.9 Hz), 154.9, 141.7, 135.1 (d, *J* = 10.4 Hz), 132.3, 131.4, 129.3, 120.3, 119.8, 112.6 (d, *J* = 3.6 Hz), 111.0, 110.4 (d, *J* = 19.8 Hz), 109.6, 94.5 (d, *J* = 18.0 Hz), 35.3, 27.5.

^{19}F NMR (471 MHz, CD_3CN, 298 K, δ): –107.7 (s).

HRMS-ESI (m/z) calc'd for $C_{20}H_{15}N_2O_2FNa^+$ $[M+Na]^+$, 357.1010; found, 357.1010; deviation: – 0.13 ppm.

Biphenyl imidazole derivative 13a, 13b

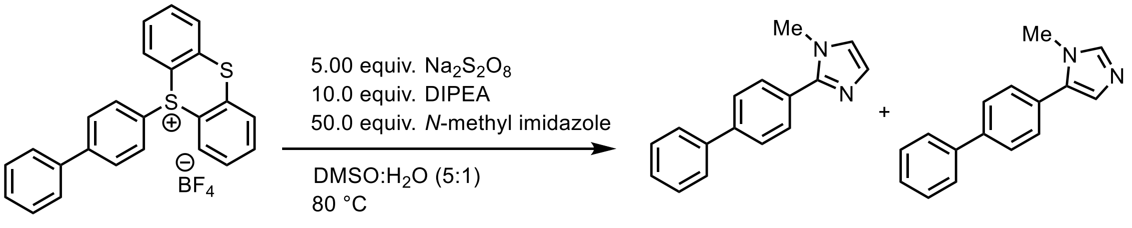

TT-3 **13a**, 54% **13b**, 17%

A 25 mL round bottom flask equipped with a magnetic stir bar was charged with **TT-3** (137 mg, 0.300 mmol, 1.00 equiv.), $Na_2S_2O_8$ (0.36 g, 1.5 mmol, 3.0 equiv.), and *N*-methyl imidazole (1.2 mL, 15 mmol, 50 equiv.). Subsequently, DMSO (2.0 mL), and H_2O (1.0 mL) were added, followed by DIPEA (0.52 mL, 3.0 mmol, 10 equiv.). The round bottom flask was capped with a septum, and placed in an oil bath at 80 °C for 20 h. After cooling to 25 °C, EtOAc (15 mL) and H_2O (15 mL) were added to the reaction mixture. The resulting mixture was transferred to a separatory funnel and extracted with EtOAc (3 × 15 mL). The combined organic layers were dried over $MgSO_4$, and concentrated under reduced pressure. The residue was purified by chromatography on silica gel eluting with hexanes/EtOAc (5:1–5:3 (v/v)) to afford 38 mg (54%) of **13a** as a white solid and 12 mg (17%) of **13b** as a pale yellow solid.

Data for 13a:

R_f = 0.08 (hexanes/EtOAc, 3:10 (v/v)).

NMR Spectroscopy:

^{1}H NMR (500 MHz, $CDCl_3$, 298 K, δ): 7.70 (q, *J* = 8.4 Hz, 4H), 7.64 – 7.60 (m, 2H), 7.45 (t, *J* = 7.6 Hz, 2H), 7.40 – 7.33 (m, 1H), 7.18 (s, 1H), 7.01 (s, 1H), 3.81 (s, 3H).

^{13}C NMR (126 MHz, $CDCl_3$, 298 K, δ): 147.5, 141.7, 140.4, 129.2, 129.0, 128.1, 127.8, 127.4, 127.2, 124.2, 122.6, 34.8.

HRMS-ESI (m/z) calc'd for $C_{16}H_{15}N_2^+$ $[M+H]^+$, 235.1229; found, 235.1230; deviation: + 0.35 ppm.

Data for 13b:

R_f = 0.10 (DCM/EtOAc, 4:1 (v/v)).

NMR Spectroscopy:

^{1}H NMR (600 MHz, $CDCl_3$, 298 K, δ): 7.72 (s, 1H), 7.68 (d, *J* = 8.5 Hz, 2H), 7.63 (d, *J* = 8.3 Hz, 2H), 7.49 – 7.45 (m, 4H), 7.38 (t, *J* = 7.4 Hz, 1H), 7.18 (s, 1H), 3.74 (s, 3H).

^{13}C NMR (151 MHz, $CDCl_3$, 298 K, δ): 141.2, 140.4, 139.1, 133.5, 129.0, 129.0, 128.3, 127.8, 127.6, 127.2, 127.2, 33.0.

HRMS-EI (m/z) calc'd for $C_{16}H_{14}N_2^+$ $[M]^+$, 234.1154; found, 234.1152; deviation: – 1.08 ppm.

Indomethacin-*N*-methyl pyrrole derivative (14)

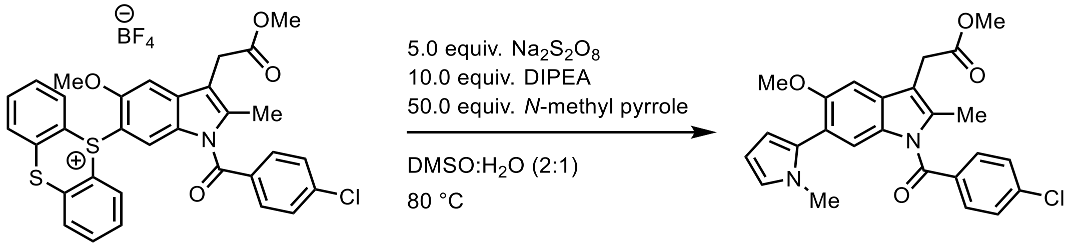

TT-14 **14**, 41%

A 25 mL round bottom flask equipped with a magnetic stir bar was charged with **TT-14** (202 mg, 0.300 mmol, 1.00 equiv.), $Na_2S_2O_8$ (3.6 g, 1.5 mmol, 5.0 equiv.), and *N*-methyl pyrrole (1.33 mL, 15.0 mmol, 50.0 equiv.). Subsequently, DMSO (2.0 mL), and H_2O (1.0 mL) were added, followed by *n*-Bu_3N (0.52 mL, 3.0 mmol, 10 equiv.). The round bottom flask was capped with a septum, and placed in an oil bath at 80 °C for 20 h. After cooling to 25 °C, H_2O (15 mL) was added to the reaction mixture. The resulting mixture was transferred to a separatory funnel and extracted with EtOAc (3 × 15 mL). The combined organic layers were dried over $MgSO_4$, and concentrated under reduced pressure. The residue was purified by chromatography on silica gel eluting with hexanes/EtOAc (1:0–3:1 (v/v)). The product-containing fractions were collected and concentrated under reduced pressure. The residue was further dried in vacuo to afford 54.9 mg (41%) of **14** as a yellow liquid.

R_f = 0.52 (hexanes/EtOAc, 1:1 (v/v)).

NMR Spectroscopy:

^{1}H NMR (500 MHz, $CDCl_3$, 298 K, δ): 7.69 (d, *J* = 7.0 Hz, 2H), 7.46 (d, *J* = 7.0 Hz, 2H), 7.00 (s, 1H), 6.95 (s, 1H), 6.67 (s, 1H), 6.15 (s, 1H), 5.94 (d, *J* = 3.7 Hz, 1H), 3.85 (s, 3H), 3.74 (s, 3H), 3.72 (s, 2H), 3.40 (s, 3H), 2.40 (s, 3H).

^{13}C NMR (126 MHz, $CDCl_3$, 298 K, δ):171.5, 168.3, 154.3, 139.6, 136.2, 133.7, 131.5, 130.5, 130.2, 129.2, 122.5, 118.9, 117.8, 112.4, 109.3, 107.5, 99.5, 55.9, 52.3, 34.6, 30.3, 13.4.

HRMS-ESI (m/z) calc'd for $C_{25}H_{23}ClN_2O_4Na^+$ $[M+Na]^+$,472.1239; found, 473.1239; deviation: - 0.16 ppm.

2-([1,1'-Biphenyl]-4-yl)-1-methyl-1H-pyrrole (15)

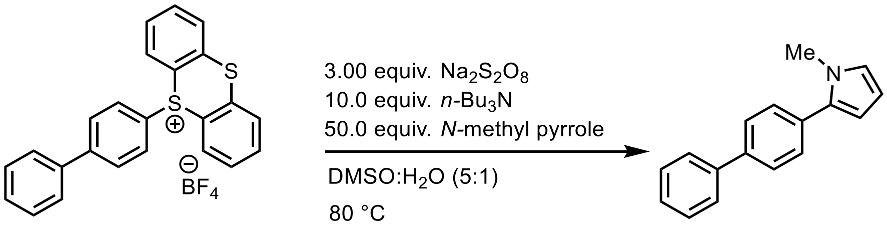

TT-3 **15**, 54%

A 25 mL round bottom flask equipped with a magnetic stir bar was charged with **TT-3** (137 mg, 0.300 mmol, 1.00 equiv.), $Na_2S_2O_8$ (0.21 g, 0.90 mmol, 3.0 equiv.), and *N*-methyl pyrrole (1.33 mL, 15.0 mmol, 50.0 equiv.). Subsequently, DMSO (2.5 mL), and H_2O (0.5 mL) were added, followed by *n*-Bu_3N (0.71 mL, 3.0 mmol, 10 equiv.). The round bottom flask was capped with a septum, and placed in an oil bath at 80 °C for 20 h. After cooling to 25 °C, H_2O (15 mL) was added to the reaction mixture. The resulting mixture was transferred to a separatory funnel and extracted with EtOAc (3 × 15 mL). The combined organic layers were dried over $MgSO_4$, and concentrated under reduced pressure. The residue was purified by chromatography on silica gel eluting with hexanes/EtOAc (1:0–50:1 (v/v)) to afford 38 mg (54%) of **15** as a colorless solid.

R_f = 0.84 (hexanes/EtOAc, 1:1 (v/v)).

NMR Spectroscopy:

^{1}H NMR (500 MHz, $CDCl_3$, 298 K, δ): 7.65 – 7.62 (m, 4H), 7.50 – 7.43 (m, 4H), 7.36 (t, J = 7.3 Hz, 1H), 6.76 – 6.73 (m, 1H), 6.28 (dd, J = 3.7, 1.8 Hz, 1H), 6.23 (dd, J = 3.6, 2.7 Hz, 1H), 3.72 (s, 3H).

^{13}C NMR (126 MHz, $CDCl_3$, 298 K, δ): 140.8, 139.6, 134.4, 132.5, 129.0, 129.0, 127.4, 127.2, 127.1, 124.0, 109.0, 108.0, 35.3.

HRMS-ESI (m/z) calc'd for $C_{17}H_{16}N^+$ [M-H]$^+$, 234.1279; found, 234.1277; deviation: – 0.75 ppm.

2-(4-Methoxy-2-methyl-5-nitrophenyl)-1-methyl-1H-pyrrole (16)

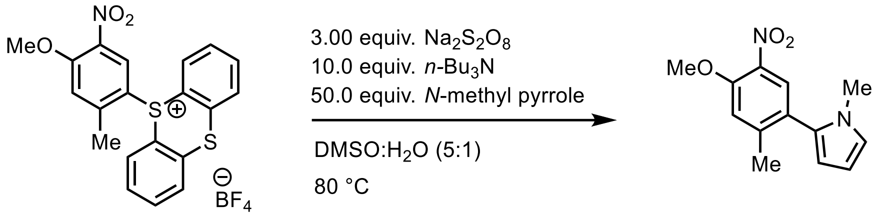

TT-16 **16**, 67%

A 25 mL round bottom flask equipped with a magnetic stir bar was charged with **TT-16** (188 mg, 0.400 mmol, 1.00 equiv.), $Na_2S_2O_8$ (0.28 g, 1.2 mmol, 3.0 equiv.), and *N*-methyl pyrrole (1.8 mL, 20 mmol, 50 equiv.). Subsequently, DMSO (3.35 mL), and H_2O (0.67 mL) were added, followed by *n*-Bu_3N (0.95 mL, 4.0 mmol, 10 equiv.). The round bottom flask was capped with a ground glass stopper and placed in an oil bath at 80 °C for 12 h. After cooling to 25 °C, H_2O (15 mL) was added to the reaction mixture. The resulting mixture was transferred to a separatory funnel and extracted with EtOAc (3 × 15 mL). The combined organic layers were dried over $MgSO_4$, and concentrated under reduced pressure. The residue was purified by chromatography on silica gel eluting with hexanes/EtOAc (1:0–10:1 (v/v)) to afford 65.8 mg (67%) of **16** as a yellow solid.

R_f = 0.16 (EtOAc/hexanes, 1:10 (v/v)).

NMR Spectroscopy:

^{1}H NMR (500 MHz, $CDCl_3$, 298 K, δ): 7.80 (s, 1H), 6.98 (s, 1H), 6.72 (dd, J = 2.7, 1.7 Hz, 1H), 6.20 (dd, J = 3.6, 2.7 Hz, 1H), 6.07 (dd, J = 3.5, 1.7 Hz, 1H), 3.99 (s, 3H), 3.41 (s, 3H), 2.26 (s, 3H).

^{13}C NMR (126 MHz, $CDCl_3$, 298 K, δ): 152.7, 146.4, 137.1, 130.3, 128.5, 125.7, 122.6, 114.9, 109.6, 107.7, 56.7, 34.3, 21.0.

HRMS-EI (m/z) calc'd for $C_{13}H_{14}N_2O_3^+$ [M]$^+$, 246.1002; found, 246.0999; deviation: – 1.09 ppm.

20 equivalents hetarene

A 10 mL round bottom flask equipped with a magnetic stir bar was charged with **TT-16** (117 mg, 0.250 mmol, 1.00 equiv.), $Na_2S_2O_8$ (0.18 g, 0.75 mmol, 3.0 equiv.), and *N*-methyl pyrrole (0.45 mL, 5.0 mmol, 20 equiv.). Subsequently, DMSO (1.0 mL), and H_2O (0.2 mL) were added, followed by *n*-Bu_3N (0.59 mL, 2.5 mmol, 10 equiv.). The round bottom flask was capped with a ground glass stopper and placed in an oil bath at 80 °C for 18 h. After cooling to 25 °C, H_2O (15 mL) was added to the reaction mixture. The resulting mixture was transferred to a separatory funnel and extracted with EtOAc (3 × 15 mL). The combined organic layers were

dried over $MgSO_4$, and concentrated under reduced pressure. The residue was purified by chromatography on silica gel eluting with Et_3N/hexanes/EtOAc (3:100:0–3:100:10 (v/v/v)) to afford 45.6 mg (74%) of **16** as a yellow solid.

5-([1,1'-Biphenyl]-4-yl)-1-methyl-1H-pyrazole (17)

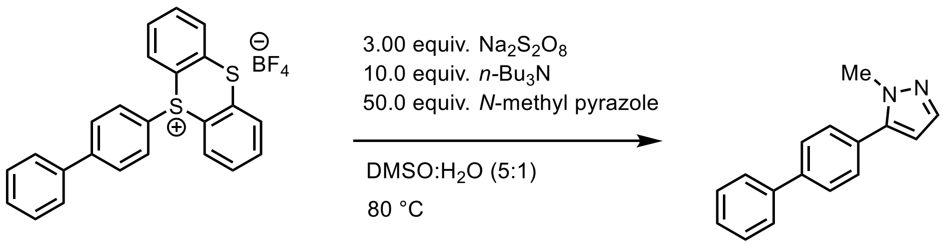

TT-3 **17**, 54%

A 25 mL round bottom flask equipped with a magnetic stir bar was charged with **TT-3** (182 mg, 0.400 mmol, 1.00 equiv.), $Na_2S_2O_8$ (0.286 g, 1.20 mmol, 3.00 equiv.), and *N*-methyl pyrazole (1.67 mL, 20.0 mmol, 50.0 equiv.). Subsequently, DMSO (3.5 mL), and H_2O (0.70 mL) were added, followed by *n*-Bu_3N (0.95 mL, 4.0 mmol, 10 equiv.). The round bottom flask was capped with a ground glass stopper and placed in an oil bath at 80 °C for 12 h. After cooling to 25 °C, H_2O (15 mL) was added to the reaction mixture. The resulting mixture was transferred to a separatory funnel and extracted with EtOAc (2 × 20 mL). The combined organic layers were dried over $MgSO_4$, and concentrated under reduced pressure. The residue was purified by chromatography on silica gel eluting with Et_3N/EtOAc/hexanes (3:0:100–3:20:100 (v/v)) to afford 50.5 mg (54%) of **17** as a yellow solid.

R_f = 0.22 (EtOAc/hexanes, 1:1 (v/v)). The TLC plate was treated with a solution of 3% Et_3N in EtOAc and subsequently dried at ambient conditions.

NMR Spectroscopy:

^{1}H NMR (500 MHz, $CDCl_3$, 298 K, δ): 7.69 (d, J = 8.5 Hz, 2H), 7.65 – 7.62 (m, 2H), 7.54 (d, J = 1.9 Hz, 1H), 7.52 – 7.45 (m, 4H), 7.39 (t, J = 7.4 Hz, 1H), 6.36 (d, J = 1.8 Hz, 1H), 3.95 (s, 3H).

^{13}C NMR (126 MHz, $CDCl_3$, 298 K, δ): 143.4, 141.4, 140.4, 138.7, 129.7, 129.2, 129.1, 127.8, 127.5, 127.2, 106.2, 37.7.

HRMS-EI (m/z) calc'd for $C_{16}H_{14}N_2^+$ $[M]^+$, 234.1154; found, 234.1152; deviation: – 1.12 ppm.

Famoxadone *N*-methyl pyrazole derivative (18)

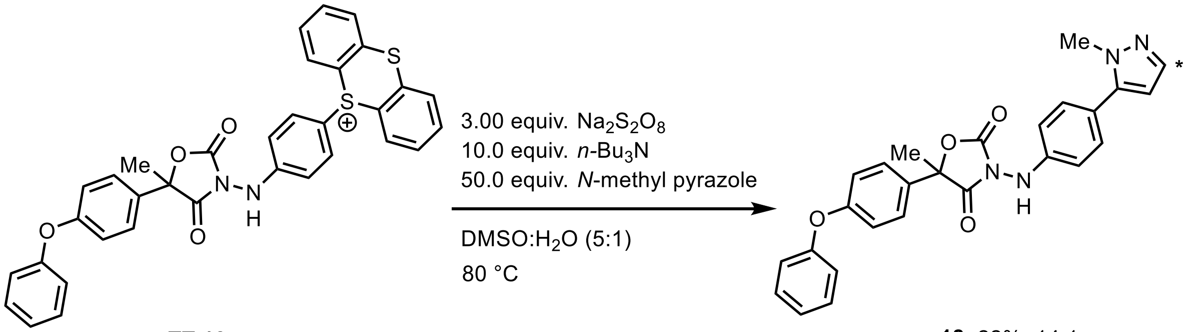

TT-18 **18**, 22%, 14:1

A 25 mL round bottom flask equipped with a magnetic stir bar was charged with **TT-18** (90 mg, 0.12 mmol, 1.0 equiv.), $Na_2S_2O_8$ (89 mg, 0.36 mmol, 3.0 equiv.), and *N*-methyl pyrazole (0.52 mL, 6.0 mmol, 50 equiv.). Subsequently, DMSO (1.0 mL), and H_2O (0.20 mL) were added followed by *n*-Bu_3N (0.30 mL, 1.2 mmol, 10 equiv.). The round bottom flask was capped with a ground glass stopper and placed in an oil bath at 80 °C for 12 h. After cooling to 25 °C, H_2O (15 mL) was added to the reaction mixture. The resulting mixture was transferred to a separatory funnel and extracted with EtOAc (2 × 20 mL). The combined organic layers were dried over $MgSO_4$, and concentrated under reduced pressure. The residue was purified by chromatography on silica gel eluting with Et_3N/EtOAc/hexanes (3:10:100–3:50:100 (v/v/v)) to afford 12 mg (22%) of **18** as colorless crystals.

Data for major isomer 18:

R_f = 0.20 (EtOAc/hexanes, 1:1 (v/v)).

NMR Spectroscopy:

^{1}H NMR (500 MHz, $CDCl_3$, 298 K, δ): 7.55 (d, *J* = 8.7 Hz, 2H), 7.48 (d, *J* = 1.8 Hz, 1H), 7.39 – 7.34 (m, 2H), 7.29 (d, *J* = 6.9 Hz, 2H), 7.16 (td, *J* = 7.5, 1.1 Hz, 1H), 7.06 – 7.01 (m, 4H), 6.79 (d, *J* = 8.7 Hz, 2H), 6.39 (s, 1H), 6.23 (s, 1H), 3.84 (s, 3H), 2.01 (s, 3H).

^{13}C NMR (151 MHz, $CDCl_3$, 298 K, δ): 172.1, 158.8, 156.3, 152.8, 144.6, 143.1, 138.6, 130.2, 130.2, 130.1, 126.2, 125.5, 124.3, 119.7, 118.8, 114.1, 106.0, 85.4, 37.5, 25.7.

HRMS-ESI (m/z) calc'd for $C_{26}H_{23}N_4O_4^+$ $[M+H]^+$, 455.1717; found, 455.1714; deviation: – 0.59 ppm.

8-(4-(4-Bromophenoxy)phenyl)-1,3,7-trimethyl-3,7-dihydro-1H-purine-2,6-dione (19)

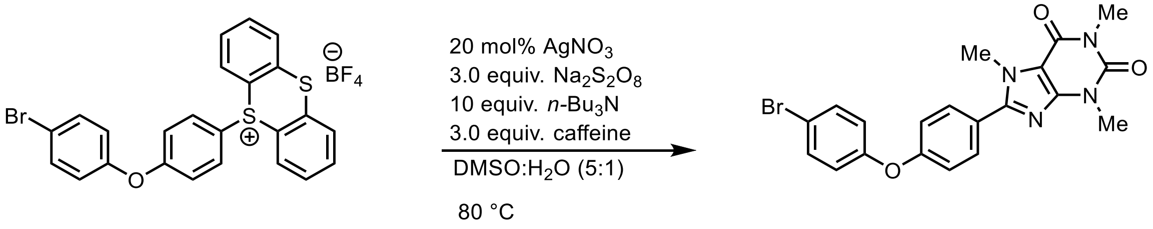

TT-4 **19**, 25%

A 25 mL round bottom flask equipped with a magnetic stir bar was charged with **TT-4** (193 mg, 0.350 mmol, 1.00 equiv.), $Na_2S_2O_8$ (0.250 g, 1.05 mmol, 3.00 equiv.), $AgNO_3$ (12 mg, 0.07 mmol, 20 mol%), and caffeine (0.204 g, 1.05 mmol, 3.00 equiv.). Subsequently, DMSO (3.0 mL), and H_2O (0.6 mL) were added followed by *n*-Bu_3N (0.83 mL, 3.5 mmol, 10 equiv.). The round bottom flask was capped with a ground glass stopper and placed in an oil bath at 80 °C for 12 h. After cooling to 25 °C, H_2O (15 mL) was added to the reaction mixture. The resulting mixture was transferred to a separatory funnel and extracted with EtOAc (2 × 20 mL). The combined organic layers were dried over $MgSO_4$, and concentrated under reduced pressure. The residue was purified by chromatography on silica gel eluting with Et_3N/EtOAc/hexanes (3:20:100–3:50:100 (v/v/v)) to afford 38 mg (25%) of **19** as a light yellow solid.

R_f = 0.35 (EtOAc/hexanes, 1:1 (v/v)). The TLC plate was treated with a solution of 3% Et_3N in EtOAc and subsequently dried at ambient conditions.

NMR Spectroscopy:

^{1}H NMR (500 MHz, $CDCl_3$, 298 K, δ): 7.67 (d, *J* = 8.9 Hz, 2H), 7.49 (d, *J* = 9.0, 2H), 7.11 (d, *J* = 8.9 Hz, 2H), 6.95 (d, *J* = 9.0 Hz, 2H), 4.06 (s, 3H), 3.63 (s, 3H), 3.43 (s, 3H).

^{13}C NMR (126 MHz, $CDCl_3$, 298 K, δ): 159.1, 155.7, 155.4, 151.8, 151.7, 148.4, 133.1, 131.1, 123.4, 121.4, 118.7, 117.0, 108.6, 34.0, 29.9, 28.1.

HRMS-ESI (m/z) calc'd for $C_{20}H_{18}BrN_4O_3^+$ $[M+H]^+$, 441.0556; found, 441.0557; deviation: + 0.11 ppm.

1-Methyl-2-(thieno[3,2-b]thiophen-2-yl)-1H-pyrrole (20)

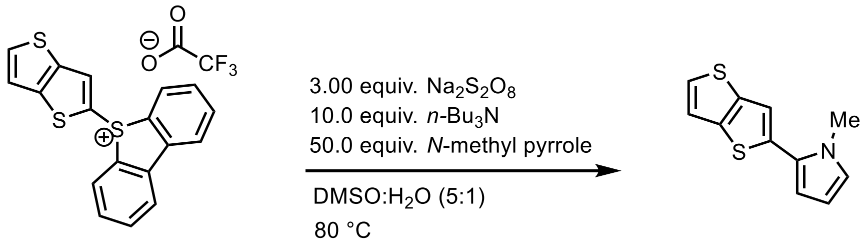

TT-20 **20**, 72%

A 25 mL round bottom flask equipped with a magnetic stir bar was charged with **TT-20** (175 mg, 0.400 mmol, 1.00 equiv.), $Na_2S_2O_8$ (0.29 g, 1.2 mmol, 3.0 equiv.), and *N*-methyl pyrrole (1.8 mL, 20 mmol, 50 equiv.). Subsequently, DMSO (3.5 mL), and H_2O (0.70 mL) were added, followed by *n*-Bu_3N (0.95 mL, 4.0 mmol, 10 equiv.). The round bottom flask was capped with a ground glass stopper and placed in an oil bath at 80 °C for 12 h. After cooling to 25 °C, H_2O (15 mL) was added to the reaction mixture. The resulting mixture was transferred to a separatory funnel and extracted with EtOAc (2 × 20 mL). The combined organic layers were dried over $MgSO_4$, and concentrated under reduced pressure. The residue was purified by chromatography on silica gel eluting with Et_3N/EtOAc/hexanes (3:0:100–3:2:100 (v/v/v)) to afford 62.8 mg (72%) of **20** as a colorless oil.

R_f = 0.59 (EtOAc/hexanes, 20:100 (v/v)). The TLC plate was treated with a solution of 3% Et_3N in EtOAc and

subsequently dried at ambient conditions.

NMR Spectroscopy:

^{1}H NMR (300 MHz, CD_3CN , 298 K, δ): 7.45 (d, *J* = 5.3 Hz, 1H), 7.32 – 7.28 (m, 2H), 6.78 (dd, *J* = 2.7, 1.8 Hz, 1H), 6.32 (dd, *J* = 3.7, 1.8 Hz, 1H), 6.11 (dd, *J* = 3.7, 2.7 Hz, 1H), 3.74 (s, 3H).

^{13}C NMR (75 MHz, CD_3CN, 298 K, δ): 140.7, 138.9, 138.1, 128.0, 127.9, 126.0, 120.6, 117.6, 111.0, 108.8, 35.8.

HRMS-EI (m/z) calc'd for $C_{11}H_9NS_2^+$ [M]$^+$, 219.0172; found, 219.0171; deviation: – 0.53 ppm.

1-(5-(2,6-Dimethoxypyridin-3-yl)-1-methyl-1H-pyrrol-2-yl)ethan-1-one (21)

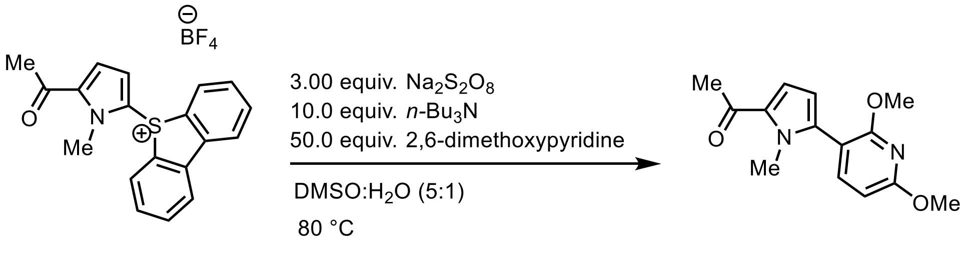

TT-21 **21**, 45%

A 25 mL round bottom flask equipped with a magnetic stir bar was charged with **TT-21** (182 mg, 0.460 mmol, 1.00 equiv.), $Na_2S_2O_8$ (0.330 g, 1.39 mmol, 3.00 equiv.), and 2,6-dimethoxypyridine (3.0 mL, 23 mmol, 50 equiv.). Subsequently, DMSO (4.0 mL), and H_2O (0.80 mL) were added, followed by *n*-Bu_3N (1.1 mL, 5.0 mmol, 10 equiv.). The round bottom flask was capped with a ground glass stopper and placed in an oil bath at 80 °C for 12 h. After cooling to 25 °C, H_2O (15 mL) was added to the reaction mixture. The resulting mixture was transferred to a separatory funnel and extracted with EtOAc (2 × 20 mL). The combined organic layers were dried over $MgSO_4$, and concentrated under reduced pressure. The residue was purified by chromatography on silica gel eluting with Et_3N/EtOAc/hexanes (3:0:100–3:5:100 (v/v/v)) to afford 54 mg (45%) of **21** as a light yellow solid.

R_f = 0.70 (EtOAc/hexanes, 1:1 (v/v)).

NMR Spectroscopy:

^{1}H NMR (300 MHz, CD_3CN, 298 K, δ): 7.81 (dd, *J* = 8.2, 0.7 Hz, 1H), 7.42 – 7.30 (m, 2H), 6.36 (dd, *J* = 8.1, 0.6 Hz, 1H), 4.01 (s, 3H), 3.90 (s, 3H), 3.89 (s, 3H), 2.40 (s, 3H).

^{13}C NMR (126 MHz, CD_3CN, 298 K, δ): 189.3, 161.9, 159.4, 139.2, 131.5, 131.0, 118.8, 118.3, 109.8, 101.9, 54.1, 53.9, 37.9, 27.4.

HRMS-EI (m/z) calc'd for $C_{14}H_{16}N_2O_3^+$ [M]$^+$, 260.1159; found, 260.1155; deviation: – 1.45 ppm.

2-Methoxy-5-(1-methyl-1H-pyrrol-2-yl)pyridine (22)

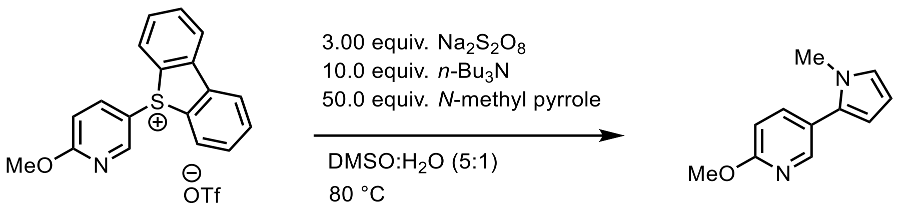

TT-22 **22**, 75%

A 25 mL round bottom flask equipped with a magnetic stir bar was charged with **TT-22** (176 mg, 0.400 mmol, 1.00 equiv.), $Na_2S_2O_8$ (0.293 g, 1.24 mmol, 3.10 equiv.), and *N*-methyl pyrrole (1.8 mL, 20 mmol, 50 equiv.). Subsequently, DMSO (4.0 mL), and H_2O (0.80 mL) were added, followed by *n*-Bu_3N (0.95 mL, 4.0 mmol, 10 equiv.). The round bottom flask was capped with a ground glass stopper and placed in an oil bath at 80 °C for 12 h. After cooling to 25 °C, H_2O (15 mL) was added to the reaction mixture. The resulting mixture was transferred to a separatory funnel and extracted with EtOAc (2 × 20 mL). The combined organic layers were dried over $MgSO_4$, and concentrated under reduced pressure. The residue was purified by chromatography on silica gel eluting with Et_3N/EtOAc/hexanes (3:0:100–3:5:100 (v/v/v)) to afford 56.6 mg (75%) of **22** as a colorless oil.

R_f = 0.71 (EtOAc/hexanes, 1:1 (v/v)).

NMR Spectroscopy:

^{1}H NMR (500 MHz, CD_3CN, 298 K, δ): 8.19 (dd, *J* = 2.5, 0.8 Hz, 1H), 7.68 (dd, *J* = 8.6, 2.5 Hz, 1H), 6.80 (dd, *J* = 8.6, 0.8 Hz, 1H), 6.75 (dd, *J* = 2.7, 1.8 Hz, 1H), 6.15 (dd, *J* = 3.6, 1.8 Hz, 1H), 6.10 (dd, *J* = 3.6, 2.7 Hz, 1H), 3.91 (s, 3H), 3.59 (s, 3H).

^{13}C NMR (126 MHz, CD_3CN, 298 K, δ): 164.0, 147.1, 140.0, 131.5, 125.0, 123.9, 111.1, 109.5, 108.5, 54.0, 35.3.

HRMS-CI (m/z) calc'd for $C_{11}H_{13}N_2O^+$ $[M+H]^+$, 189.1022; found, 189.1022; deviation: + 0.36 ppm.

1-(1,1'-Dimethyl-1H,1'H-[2,2'-bipyrrol]-5-yl)ethan-1-one (23)

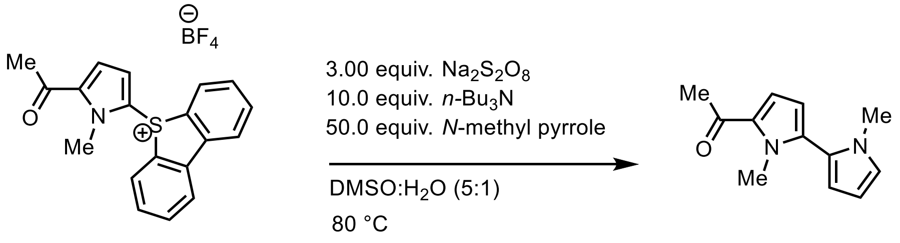

TT-21 **23**, 52%

A 25 mL round bottom flask equipped with a magnetic stir bar was charged with **TT-21** (197 mg, 0.500 mmol, 1.00 equiv.), $Na_2S_2O_8$ (0.36 g, 1.5 mmol, 3.0 equiv.), and *N*-methyl pyrrole (2.20 mL, 25.0 mmol, 50.0 equiv.). Subsequently, DMSO (4.0 mL), and H_2O (0.80 mL) were added followed by *n*-Bu_3N (1.2 mL, 5.0 mmol, 10

equiv.). The round bottom flask was capped with a ground glass stopper and placed in an oil bath at 80 °C for 12 h. After cooling to 25 °C, H_2O (15 mL) was added to the reaction mixture. The resulting mixture was transferred to a separatory funnel and extracted with EtOAc (2 × 20 mL). The combined organic layers were dried over $MgSO_4$, and concentrated under reduced pressure. The residue was purified by chromatography on silica gel eluting with Et_3N/EtOAc/hexanes (3:0:100–3:5:100 (v/v/v)) to afford 53 mg (52%) of **23** as an orange oil.

R_f = 0.23 (EtOAc/hexanes, 20:100 (v/v)). The TLC plate was treated with a solution of 3% Et_3N in EtOAc and subsequently dried at ambient conditions.

NMR Spectroscopy:

^{1}H NMR (500 MHz, CD_3CN, 298 K, δ): 7.09 (d, *J* = 2.0 Hz, 1H), 7.03 (d, *J* = 2.0 Hz, 1H), 6.64 (dd, *J* = 2.7, 1.8 Hz, 1H), 6.09 (dd, *J* = 3.5, 1.8 Hz, 1H), 6.03 (dd, *J* = 3.6, 2.7 Hz, 1H), 3.89 (s, 3H), 3.65 (s, 3H), 2.40 (s, 3H).

^{13}C NMR (126 MHz, CD_3CN, 298 K, δ): 189.4, 131.8, 129.6, 128.8, 123.6, 119.0, 116.7, 108.1, 107.8, 37.8, 35.5, 27.5.

HRMS-CI (m/z) calc'd for $C_{12}H_{15}N_2O^+$ $[M+H]^+$, 203.1177; found, 203.1179; deviation: + 0.82 ppm.

1-Methyl-2-(5-phenylthiophen-2-yl)-1H-pyrrole (24)

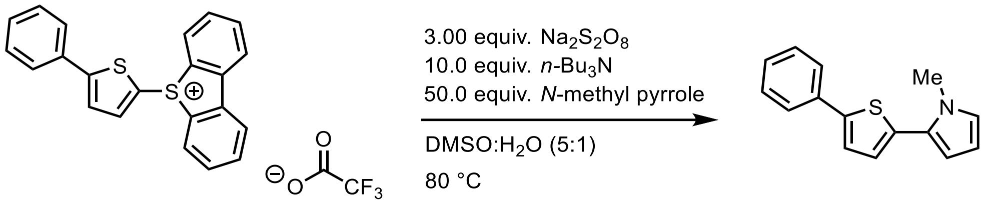

TT-24 **24**, 64%

A 25 mL round bottom flask equipped with a magnetic stir bar was charged with **TT-24** (120 mg, 0.263 mmol, 1.00 equiv.), $Na_2S_2O_8$ (0.19 g, 0.79 mmol, 3.0 equiv.), and *N*-methyl pyrrole (1.20 mL, 13.2 mmol, 50.0 equiv.). Subsequently, DMSO (2.2 mL), and H_2O (0.44 mL) were added, followed by *n*-Bu_3N (0.620 mL, 2.63 mmol, 10.0 equiv.). The round bottom flask was capped with a septum, and placed in an oil bath at 80 °C for 20 h. After cooling to 25 °C, H_2O (15 mL) was added to the reaction mixture. The resulting mixture was transferred to a separatory funnel and extracted with EtOAc (3 × 15 mL). The combined organic layers were dried over $MgSO_4$, and concentrated under reduced pressure. The residue was purified by chromatography on silica gel eluting with Et_3N/MTBE/hexanes (3:0:100–3:5:100 (v/v/v)) to afford 40.3 mg (64%) of **24** as a white solid.

R_f = 0.28 (MTBE/hexanes, 5:100 (v/v)). The TLC plate was treated with a solution of 3% Et_3N in EtOAc and subsequently dried at ambient conditions.

NMR Spectroscopy:

^{1}H NMR (500 MHz, CD_3CN, 298 K, δ): 7.69 – 7.62 (m, 2H), 7.41 (dd, *J* = 8.3, 7.2 Hz, 2H), 7.38 (d, *J* = 3.8

Hz, 1H), 7.33 – 7.28 (m, 1H), 7.07 (d, *J* = 3.8 Hz, 1H), 6.78 – 6.76 (m, 1H), 6.32 (dd, *J* = 3.7, 1.8 Hz, 1H), 6.10 (dd, *J* = 3.7, 2.7 Hz, 1H), 3.75 (s, 3H).

^{13}C NMR (500 MHz, CD_3CN, 298 K, δ): 143.1, 135.7, 135.0, 130.1, 128.5, 127.6, 126.3, 126.2, 125.8, 124.9, 110.7, 108.8, 35.9.

HRMS-EI (m/z) calc'd for $C_{15}H_{13}NS^+$ [M]$^+$, 239.0765; found, 239.0763; deviation: – 0.62 ppm.

Ethyl 3-(5-(1-methyl-1H-pyrrol-2-yl)furan-2-yl)propanoate (25)

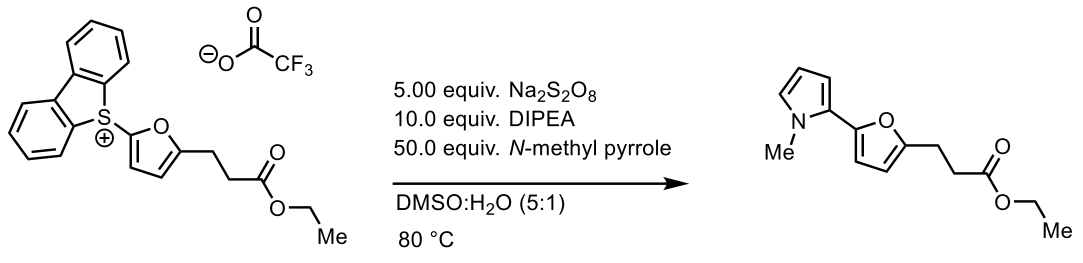

TT-25 **25**, 52%

A 25 mL round bottom flask equipped with a magnetic stir bar was charged with **TT-25** (139 mg, 0.300 mmol, 1.00 equiv.), $Na_2S_2O_8$ (0.36 g, 1.5 mmol, 5.0 equiv.), and *N*-methyl pyrrole (1.30 mL, 15.0 mmol, 50.0 equiv.). Subsequently, DMSO (2.0 mL), and H_2O (1.0 mL) were added, followed by DIPEA (0.52 mL, 3.0 mmol, 10 equiv.). The round bottom flask was capped with a ground glass stopper and placed in an oil bath at 80 °C. After 2 h, the reaction was monitored by TLC and starting material was still left so additional $Na_2S_2O_8$ (0.36 g, 1.5 mmol, 5.0 equiv.) and DIPEA (0.52 mL, 3.0 mmol, 10 equiv.) were added and the reaction was left stirring until full conversion. After cooling to 25 °C, H_2O (15 mL) was added to the reaction mixture. The resulting mixture was transferred to a separatory funnel and extracted with EtOAc (3 × 15 mL). The combined organic layers were dried over $MgSO_4$, and concentrated under reduced pressure. The residue was purified by chromatography on silica gel eluting with Et_3N/EtOAc/hexanes (3:5:100 (v/v/v)) to afford 38.5 mg (52%) of **25** as a yellow liquid.

R_f = 0.40 (EtOAc/hexanes, 10:100 (v/v)). The TLC plate was treated with a solution of 3% Et_3N in EtOAc and subsequently dried at ambient conditions.

NMR Spectroscopy:

^{1}H NMR (500 MHz, CD_3CN, 298 K, δ): 6.68 (dd, *J* = 2.7, 1.8 Hz, 1H), 6.31 – 6.27 (m, 2H), 6.12 (dt, *J* = 3.3, 1.0 Hz, 1H), 6.06 (dd, *J* = 3.7, 2.7 Hz, 1H), 4.10 (q, *J* = 7.1 Hz, 2H), 3.70 (s, 3H), 2.95 (td, *J* = 7.4, 1.0 Hz, 2H), 2.65 (t, *J* = 7.4 Hz, 2H), 1.20 (t, *J* = 7.2 Hz, 3H).

^{13}C NMR (126 MHz, CD_3CN, 298 K, δ): 173.3, 154.2, 147.5, 125.8, 125.1, 108.9, 108.4, 107.8, 106.8, 61.2, 36.1, 33.3, 24.2, 14.6.

HRMS-EI (m/z) calc'd for $C_{14}H_{17}NO_3^+$ [M]$^+$, 247.1207; found, 247.1203; deviation: – 1.68 ppm.

1-Methyl-4-(1-methyl-1H-pyrrol-2-yl)-1H-pyrazole (26)

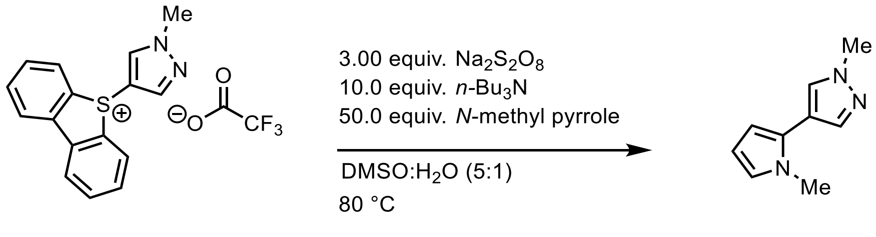

TT-26 **26**, 67%

A 25 mL round bottom flask equipped with a magnetic stir bar was charged with **TT-26** (152 mg, 0.400 mmol, 1.00 equiv.), $Na_2S_2O_8$ (0.29 g, 1.2 mmol, 3.0 equiv.), and *N*-methyl pyrrole (1.80 mL, 20.0 mmol, 50.0 equiv.). Subsequently, DMSO (3.35 mL), and H_2O (0.67 mL) were added, followed by *n*-Bu_3N (0.95 mL, 4.0 mmol, 10 equiv.). The round bottom flask was capped with a ground glass stopper and placed in an oil bath at 80 °C. After 2 h, the reaction was monitored by TLC and starting material was still left so additional $Na_2S_2O_8$ (0.29 g, 1.2 mmol, 3.0 equiv.) and *n*-Bu_3N (0.95 mL, 4.0 mmol, 10 equiv.) were added and the reaction was left stirring until full conversion. After cooling to 25 °C, H_2O (15 mL) was added to the reaction mixture. The resulting mixture was transferred to a separatory funnel and extracted with EtOAc (3 × 15 mL). The combined organic layers were dried over $MgSO_4$, and concentrated under reduced pressure. The residue was purified by chromatography on silica gel eluting with Et_3N/EtOAc/hexanes (3:10:100–3:30:100 (v/v/v)) to afford 43.1 mg (67%) of **26** as an orange liquid.

R_f = 0.11 (EtOAc/hexanes, 30:100 (v/v)). The TLC plate was treated with a solution of 3% Et_3N in EtOAc and subsequently dried at ambient conditions.

NMR Spectroscopy:

^{1}H NMR (500 MHz, CD_3CN, 298 K, δ): 7.59 (d, *J* = 0.8 Hz, 1H), 7.51 (d, *J* = 0.9 Hz, 1H), 6.65 (dd, *J* = 2.7, 1.8 Hz, 1H), 6.09 (dd, *J* = 3.5, 1.8 Hz, 1H), 6.03 (dd, *J* = 3.5, 2.7 Hz, 1H), 3.86 (s, 3H), 3.62 (s, 3H).

^{13}C NMR (75 MHz, CD_3CN, 298 K, δ): 138.3, 128.8, 126.8, 123.6, 115.4, 108.2, 107.9, 39.3, 35.4.

HRMS-EI (m/z) calc'd for $C_9H_{11}N_3^+$ $[M+Na]^+$, 161.0948; found, 161.0948; deviation: – 0.09 ppm.

Gram-Scale Synthesis

1-Bromo-4-phenoxybenzene imidazole derivative 27a, 27b

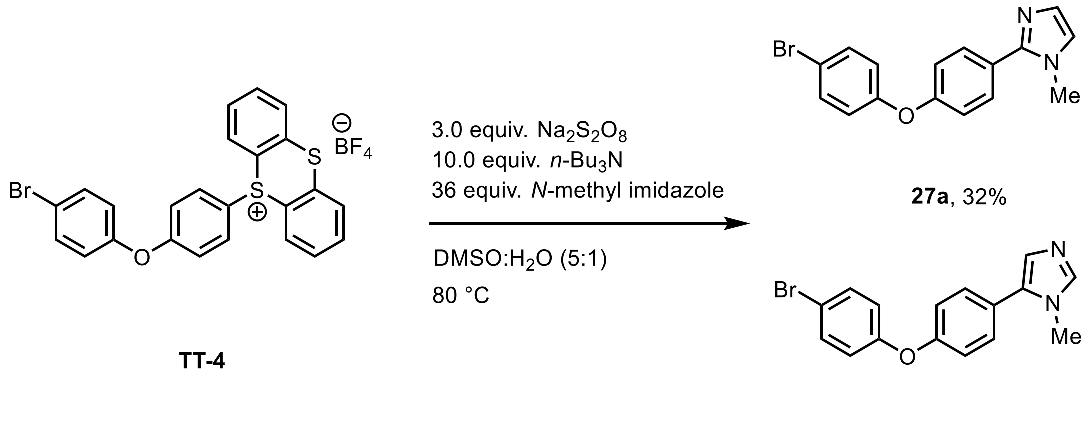

A 1000 mL round bottom flask equipped with a magnetic stir bar was charged with **TT-4** (16.5 g, 30.0 mmol, 1.00 equiv.), $Na_2S_2O_8$ (21.4 g, 90.0 mmol, 3.00 equiv.), and *N*-methyl imidazole (87.0 mL, 1.08 mol, 36.0 equiv.). Subsequently, DMSO (250 mL), and H_2O (50 mL) were added, followed by *n*-Bu_3N (71.4 mL, 300 mmol, 10.0 equiv.). The round bottom flask was capped with a ground glass stopper and placed in an oil bath at 80 °C for 16 h. After cooling to 25 °C, H_2O (150 mL) was added to the reaction mixture. The resulting mixture was transferred to a separatory funnel and extracted with EtOAc (2 × 200 mL).The combined organic layers were dried over $MgSO_4$, and concentrated under reduced pressure at 70°C. The residue was purified by chromatography on silica gel eluting with *i*-PrOH/EtOAc/hexanes (0:1:1–3:20:0 (v/v/v)). The product-containing fractions were collected and concentrated under reduced pressure. The residue was further dried in vacuo to afford 3.20 g (32%) of **27a** as a light orange solid and 2.21 g (22%) of **27b** as an orange solid.

Data for 27a:

R_f = 0.57 (MeOH/EtOAc, 1:10 (v/v)).

NMR Spectroscopy:

^{1}H NMR (500 MHz, $CDCl_3$, 298 K, δ): 7.59 (d, *J* = 8.7 Hz, 2H), 7.44 (d, *J* = 8.9 Hz, 2H), 7.09 (d, *J* = 1.3 Hz, 1H), 7.05 (d, *J* = 8.7 Hz, 2H), 6.95 (d, *J* = 1.2 Hz, 1H), 6.92 (d, *J* = 8.8 Hz, 2H), 3.73 (s, 3H).

^{13}C NMR (126 MHz, $CDCl_3$, 298 K, δ): 157.4, 156.0, 147.3, 132.9, 130.4, 128.4, 126.0, 122.4, 121.0, 118.7, 116.3, 34.6.

HRMS-ESI (m/z) calc'd for $C_{16}H_{14}BrN_2O^+$ [M+H]$^+$, 329.0282; found, 329.0284; deviation: + 0.65 ppm.

Data for 27b:

R_f = 0.44 (MeOH/EtOAc, 1:10 (v/v)).

NMR Spectroscopy:

^{1}H NMR (500 MHz, $CDCl_3$, 298 K, δ): 7.64 (d, *J* = 1.1 Hz, 1H), 7.47 (d, *J* = 9.0 Hz, 2H), 7.35 (d, *J* = 8.9

Hz, 2H), 7.09 (d, J = 1.2 Hz, 1H), 7.05 (d, J = 8.7 Hz, 2H), 6.94 (d, J = 9.0 Hz, 2H), 3.68 (s, 3H).

^{13}C NMR (126 MHz, $CDCl_3$, 298 K, δ): 156.9, 156.0, 139.0, 132.9, 132.9, 130.2, 127.9, 125.1, 121.0, 119.0, 116.3, 32.6.

HRMS-ESI (m/z) calc'd for $C_{16}H_{14}BrN_2O^+$ [M+H]$^+$, 329.0280; found, 329.0284; deviation: + 1.16 ppm.

Diversification

2-Fluoro-6-(4-(4,4,5,5-tetramethyl-1,3,2-dioxaborolan-2-yl)phenoxy)benzonitrile (28)

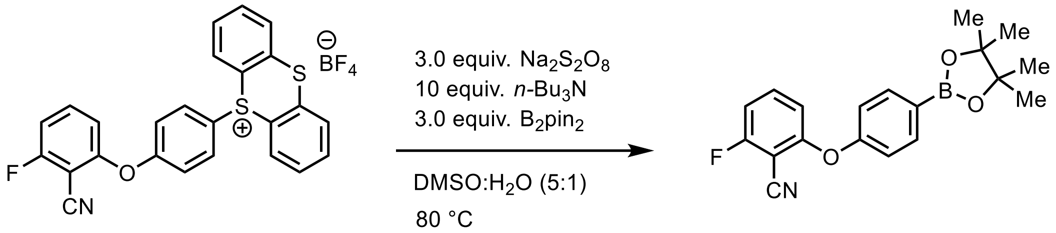

TT-12 **28**, 46%

A 25 mL round bottom flask equipped with a magnetic stir bar was charged with **TT-12** (206 mg, 0.400 mmol, 1.00 equiv.), $Na_2S_2O_8$ (0.286 g, 1.20 mmol, 3.00 equiv.), B_2pin_2 (0.305 g, 1.20 mmol, 3.00 equiv.). Subsequently, DMSO (3.0 mL), and H_2O (0.6 mL) were added, followed by *n*-Bu_3N (0.95 mL, 4.0 mmol, 10 equiv.). The round bottom flask was capped with a ground glass stopper and placed in an oil bath at 80 °C for 12 h. After cooling to 25 °C, H_2O (20 mL) was added to the reaction mixture. The resulting mixture was transferred to a separatory funnel and extracted with EtOAc (2 × 20 mL).The combined organic layers were dried over $MgSO_4$, and concentrated under reduced pressure. The residue was purified by chromatography on silica gel eluting with EtOAc/hexanes (0:100–10:100 (v/v)) to afford 61 mg (46%) of **28** as a colorless solid.

R_f = 0.83 (EtOAc/hexanes, 1:1 (v/v)).

NMR Spectroscopy:

^{1}H NMR (500 MHz, $CDCl_3$, 298 K, δ): 7.87 (d, J = 8.5 Hz, 2H), 7.41 (td, J = 8.5, 6.4 Hz, 1H), 7.09 (d, J = 8.5 Hz, 2H), 6.89 (td, J = 8.4, 0.8 Hz, 1H), 6.62 (dt, J = 8.5, 0.9 Hz, 1H), 1.35 (s, 12H).

^{13}C NMR (126 MHz, $CDCl_3$, 298 K, δ): 164.2 (d, J = 259.8 Hz), 160.8, 157.2, 137.2, 135.0 (d, J = 10.4 Hz), 119.6, 112.4 (d, J = 3.2 Hz), 111.2, 110.0 (d, J = 19.5 Hz), 94.1 (d, J = 17.7 Hz), 84.2, 25.0. The carbon directly attached to the boron atom was not detected, likely due to quadrupolar relaxation.

^{19}F NMR (470 MHz, $CDCl_3$, 298 K, δ): –104.6 (s).

HRMS-GC-EI (m/z) calc'd for $C_{19}H_{19}NO_3BF^+$ [M]$^+$, 339.1436; found, 339.1437; deviation: + 0.27 ppm.

2-(4-Allylphenyl)ethan-1-ol (29)

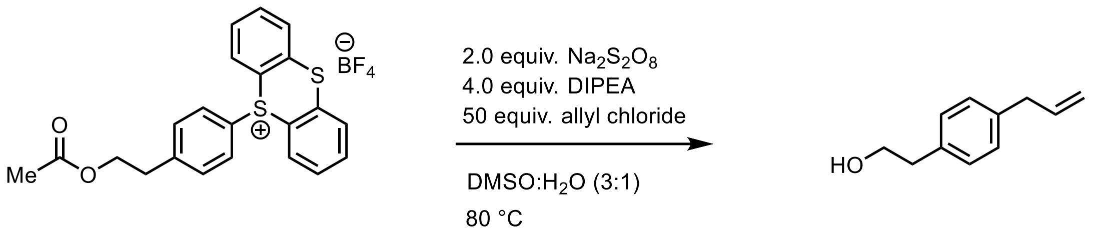

TT-29 **29**, 55%

A 25 mL round bottom flask equipped with a magnetic stir bar was charged with **TT-29** (233 mg, 0.500 mmol, 1.00 equiv.), $Na_2S_2O_8$ (0.24 g, 1.0 mmol, 2.0 equiv.), and allyl chloride (2.0 mL, 25 mmol, 50 equiv.). Subsequently, DMSO (3.6 mL), and H_2O (1.2 mL) were added, followed by DIPEA (0.35 mL, 2.0 mmol, 4.0 equiv.). The round bottom flask was capped with a ground glass stopper and placed in an oil bath at 80 °C for 12 h. After cooling to 25 °C, H_2O (20 mL) was added to the reaction mixture. The resulting mixture was transferred to a separatory funnel and extracted with EtOAc (2 × 20 mL).The combined organic layers were dried over $MgSO_4$, and concentrated under reduced pressure. The residue was purified by chromatography on silica gel eluting with EtOAc/hexanes (0:100–10:100 (v/v)) to afford 45 mg (55%) of **29** as a colorless liquid.

R_f = 0.56 (EtOAc/hexanes, 1:1 (v/v)).

NMR Spectroscopy:

^{1}H NMR (500 MHz, $CDCl_3$, 298 K, δ): 7.21 – 7.09 (m, 4H), 5.96 (ddt, *J* = 16.8, 10.1, 6.7 Hz, 1H), 5.11 – 5.04 (m, 2H), 3.85 (t, *J* = 6.6 Hz, 2H), 3.37 (d, *J* = 6.7 Hz, 2H), 2.85 (t, *J* = 6.6 Hz, 2H).

^{13}C NMR (126 MHz, $CDCl_3$, 298 K, δ): 138.4, 137.6, 136.2, 129.2, 129.0, 115.9, 63.9, 40.0, 38.9.

HRMS-ESI (m/z) calc'd for $C_{11}H_{14}ONa^+$ $[M+Na]^+$, 185.0940; found, 185.0937; deviation: – 1.49 ppm.

Methyl 5-iodo-2-methoxybenzoate (30)

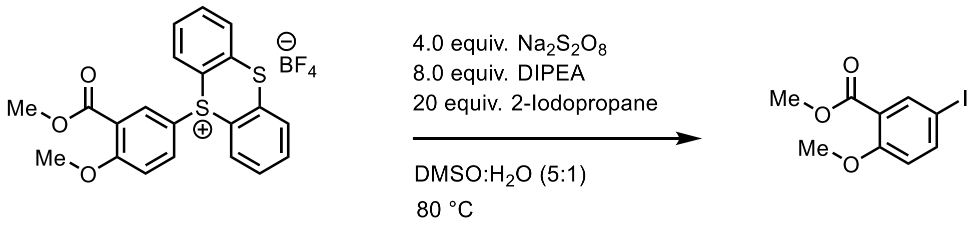

TT-30 **30**, 52%

A 25 mL round bottom flask equipped with a magnetic stir bar was charged with **TT-30** (187 mg, 0.400 mmol, 1.00 equiv.), $Na_2S_2O_8$ (0.380 g, 1.20 mmol, 4.00 equiv.), and 2-iodopropane (0.80 mL, 8.0 mmol, 20 equiv.). Subsequently, DMSO (2.0 mL), and H_2O (2.0 mL) were added, followed by DIPEA (0.56 mL, 3.2 mmol, 8.0 equiv.). The round bottom flask was capped with a ground glass stopper and placed in an oil bath at 80 °C for 12 h. After cooling to 25 °C, H_2O (20 mL) was added to the reaction mixture. The resulting mixture was transferred to a separatory funnel and extracted with EtOAc (2 × 20 mL).The combined organic layers were

dried over $MgSO_4$ and concentrated under reduced pressure. The residue was purified by chromatography on silica gel eluting with EtOAc/hexanes (0:100–5:100 (v/v)) to afford 61 mg (52%) of **30** as a colorless solid.

R_f = 0.50 (DCM).

NMR Spectroscopy:

^{1}H NMR (500 MHz, $CDCl_3$, 298 K, δ): 8.05 (d, *J* = 2.3 Hz, 1H), 7.71 (dd, *J* = 8.9, 2.4 Hz, 1H), 6.74 (d, *J* = 8.9 Hz, 1H), 3.88 – 3.86 (m, 6H).

^{13}C NMR (126 MHz, $CDCl_3$, 298 K, δ): 165.2, 159.1, 142.1, 140.1, 122.2, 114.5, 81.8, 56.2, 52.3.

HRMS-GC-MS (m/z) calc'd for $C_9H_9O_3I^+$ [M]$^+$, 291.9597; found, 291.9591; deviation: – 2.08 ppm.

SPECTROSCOPIC DATA

^{1}H NMR of 1-(4-methoxyphenyl)ethan-1-one-derived thianthrenium tetrafluoroborate (TT-7)

DMSO-d_6, 500 MHz, 298 K

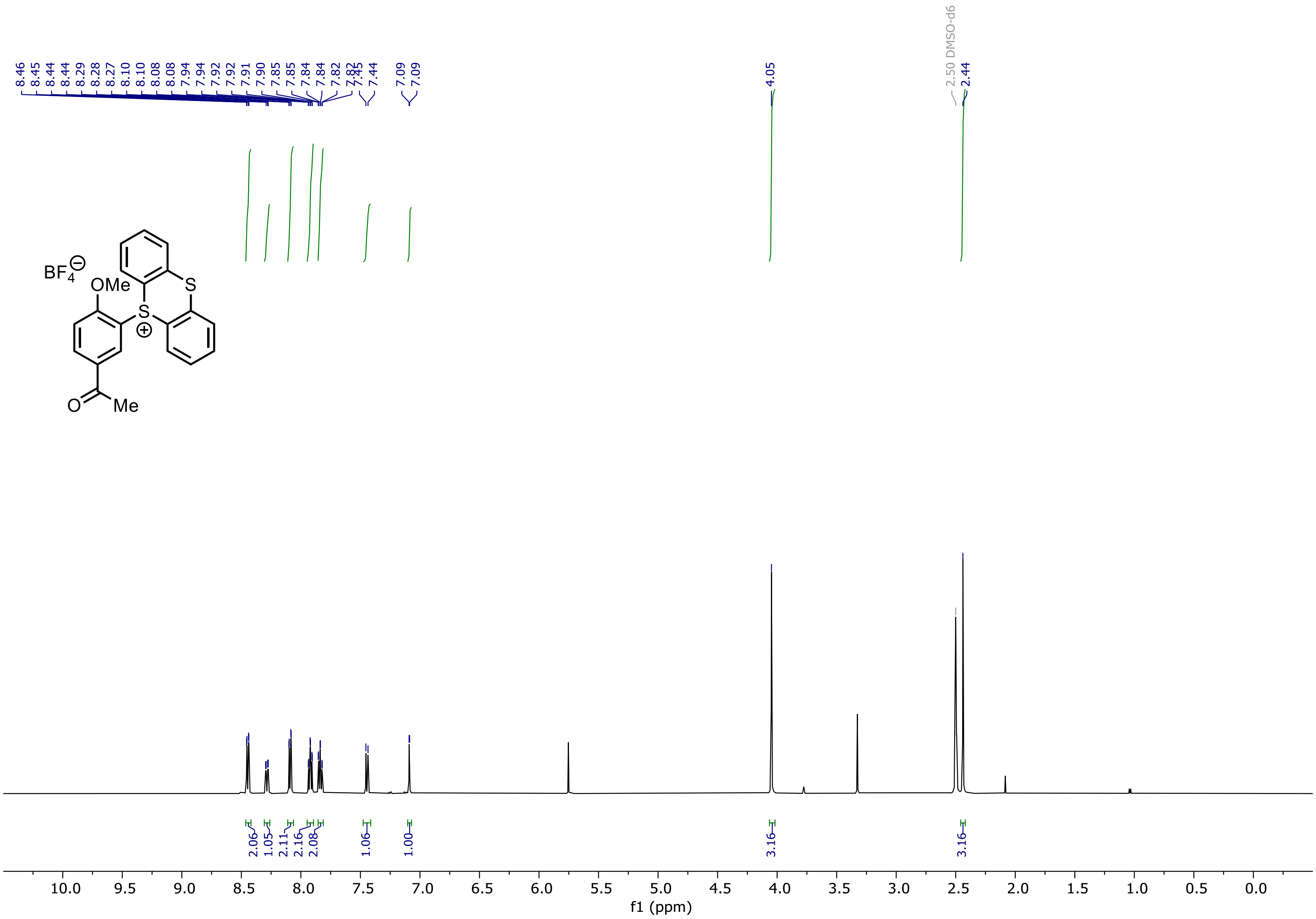

^{13}C NMR of 1-(4-methoxyphenyl)ethan-1-one-derived thianthrenium tetrafluoroborate (TT-7)

DMSO-d_6, 126 MHz, 298 K

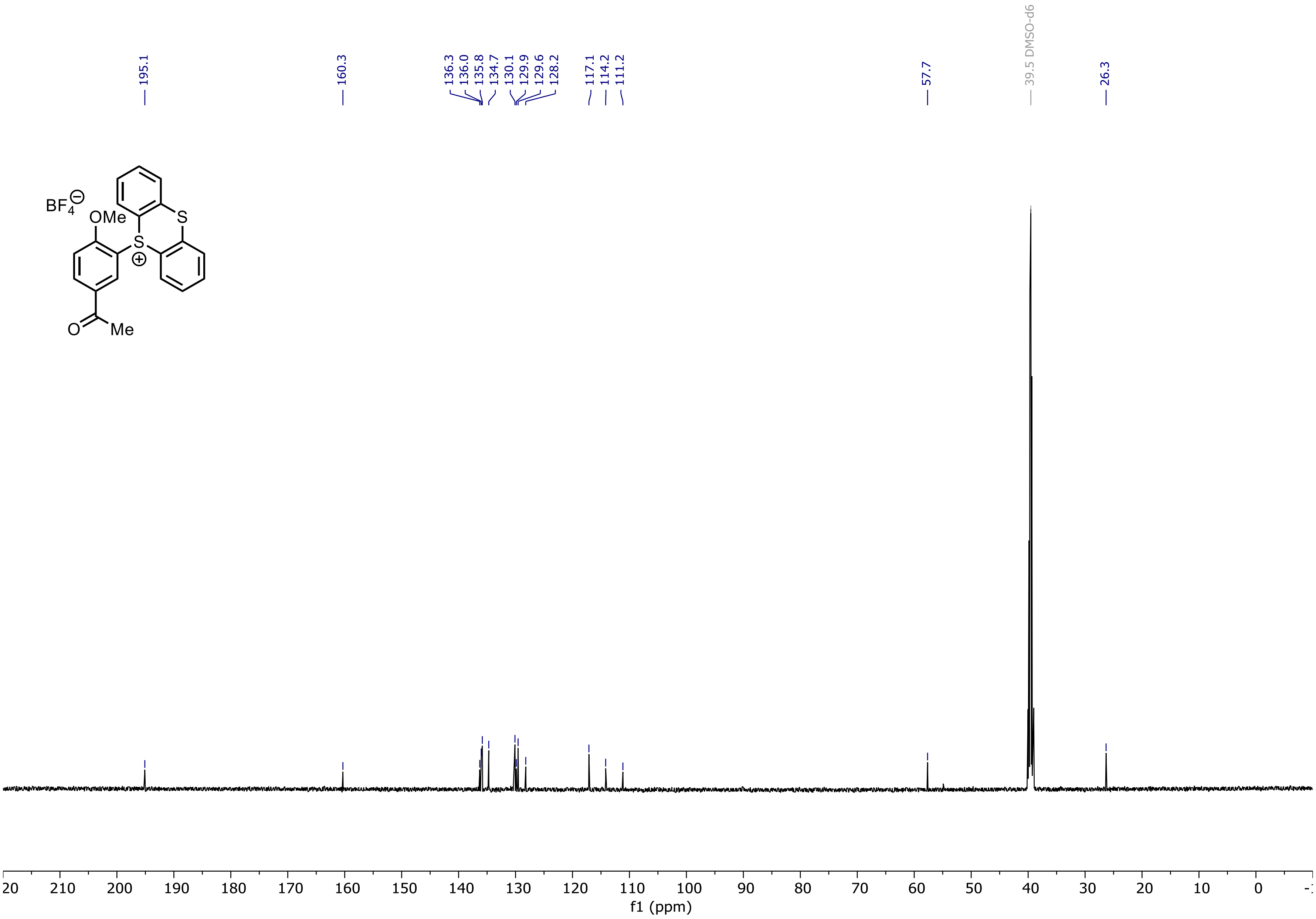

^{19}F NMR of 1-(4-methoxyphenyl)ethan-1-one-derived thianthrenium tetrafluoroborate (TT-7)

DMSO-d_6, 471 MHz, 298 K

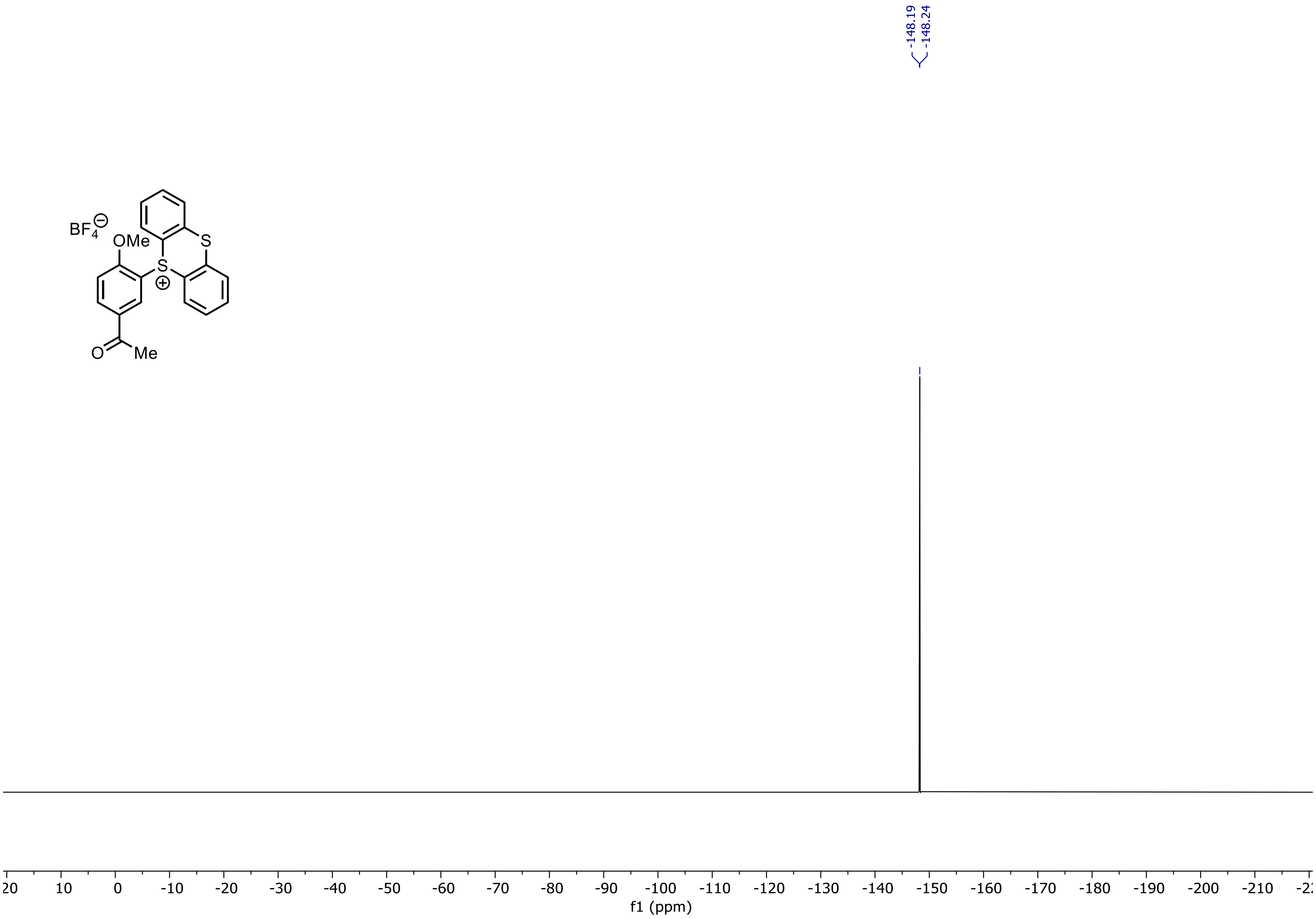

^{1}H NMR of 5-methyl-2-nitroanisole-derived thianthrenium tetrafluoroborate (TT-16)

DMSO-d_6, 500 MHz, 298 K

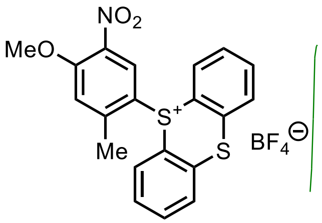

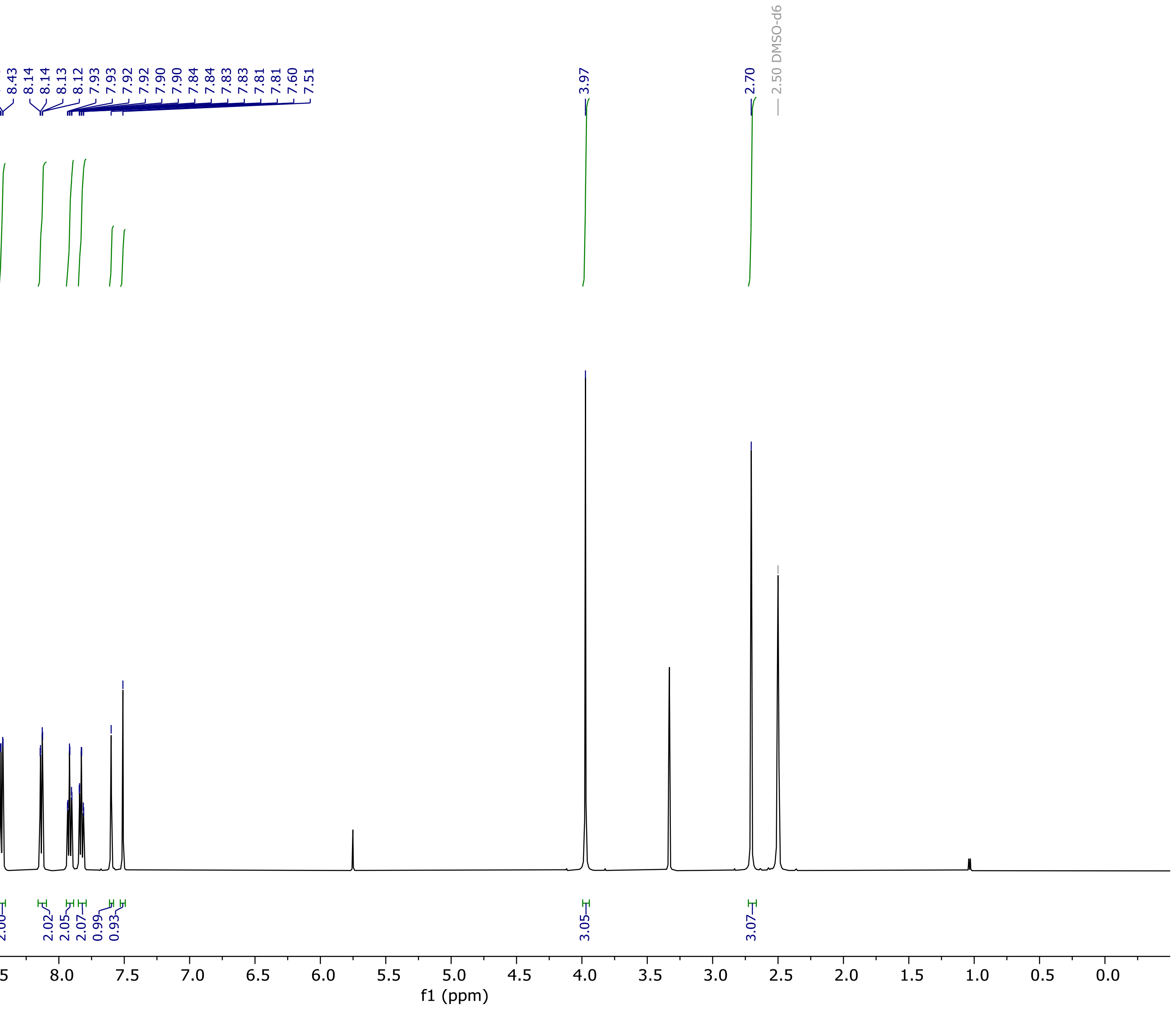

^{13}C NMR of 5-methyl-2-nitroanisole-derived thianthrenium tetrafluoroborate (TT-16)

DMSO-d_6, 126 MHz, 298 K

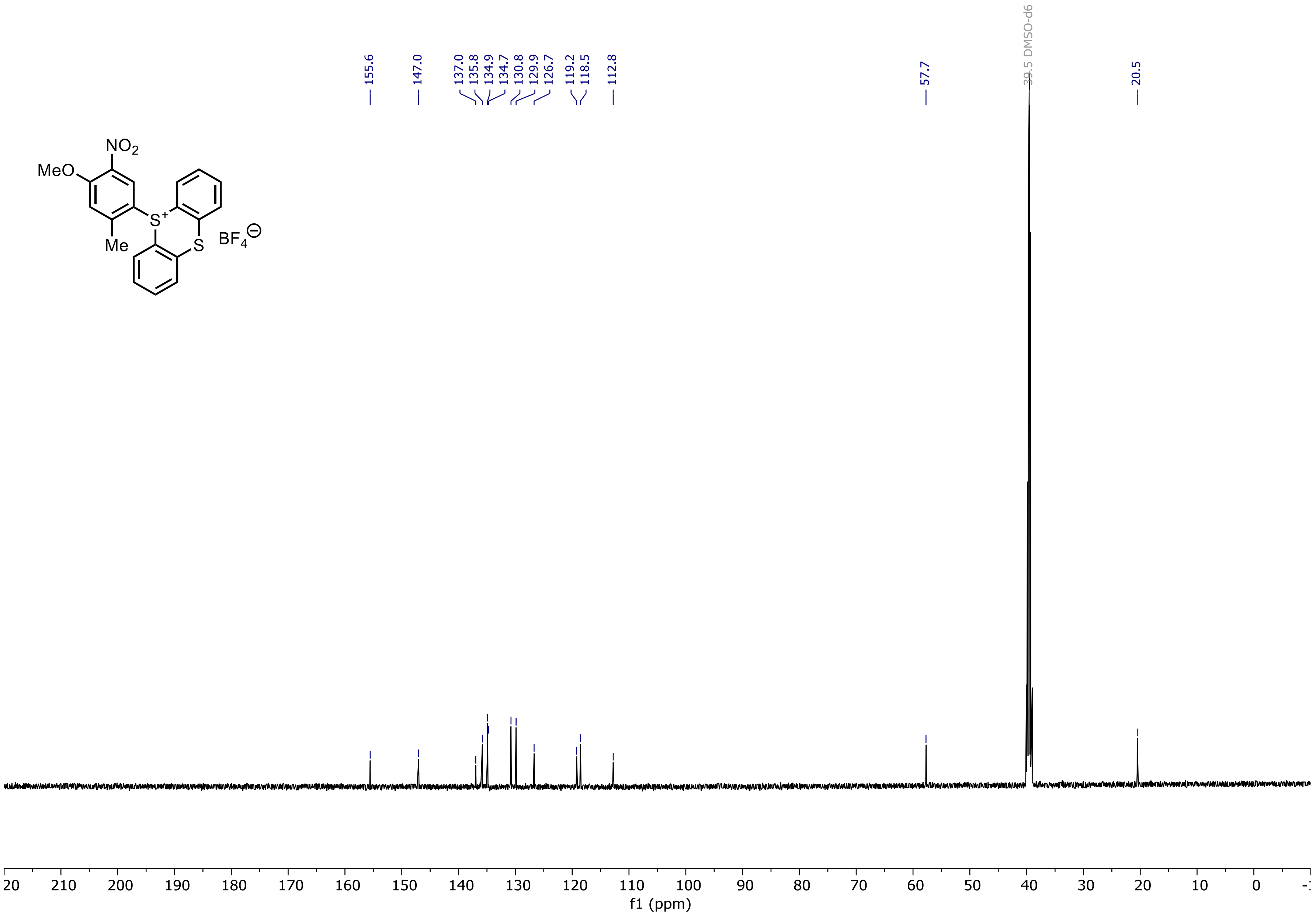

^{19}F NMR of 5-methyl-2-nitroanisole-derived thianthrenium tetrafluoroborate (TT-16)

DMSO-d_6, 471 MHz, 298 K

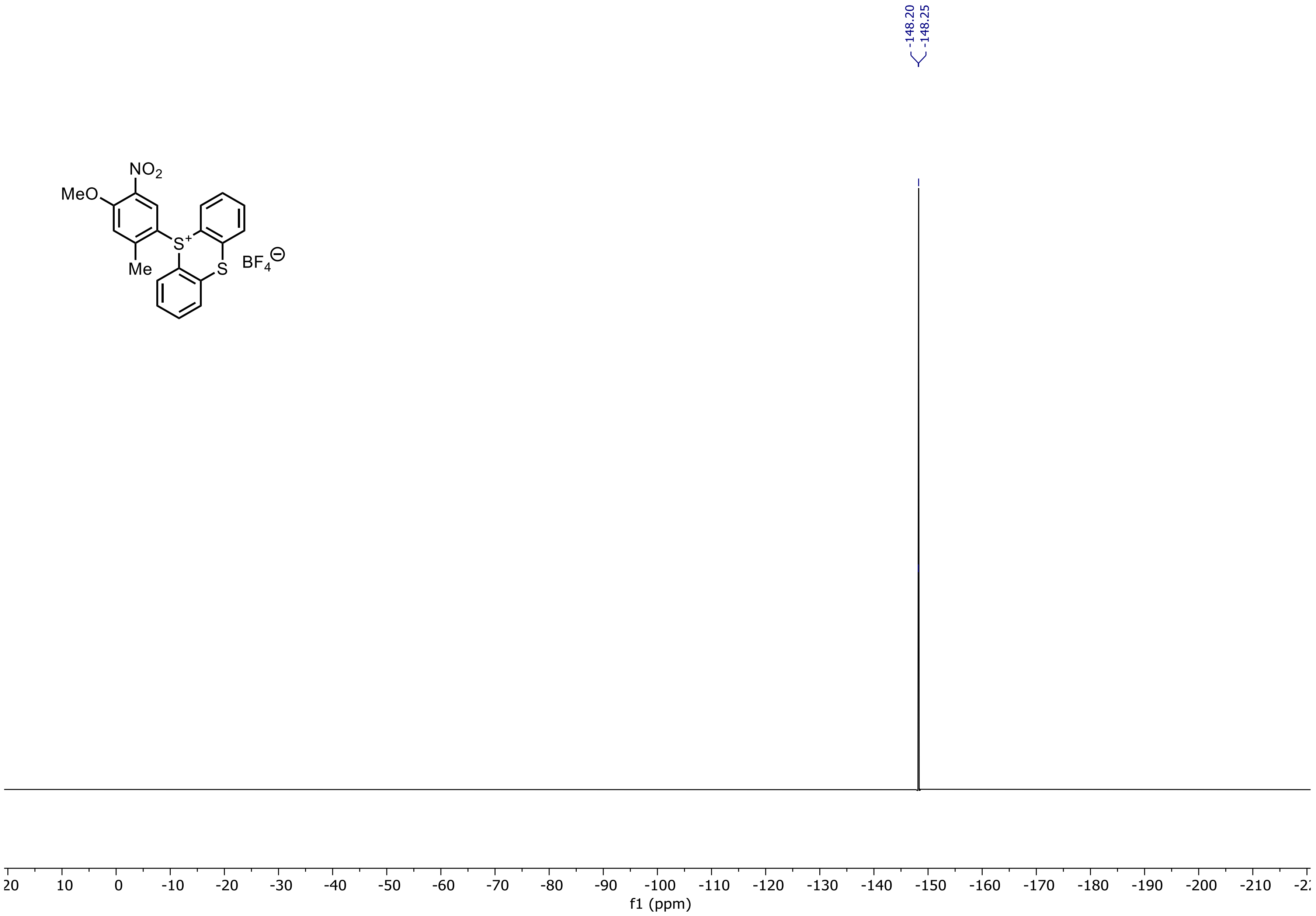

HSQC of 5-methyl-2-nitroanisole-derived thianthrenium tetrafluoroborate (TT-16)

$CDCl_3$, 600 MHz, 298 K

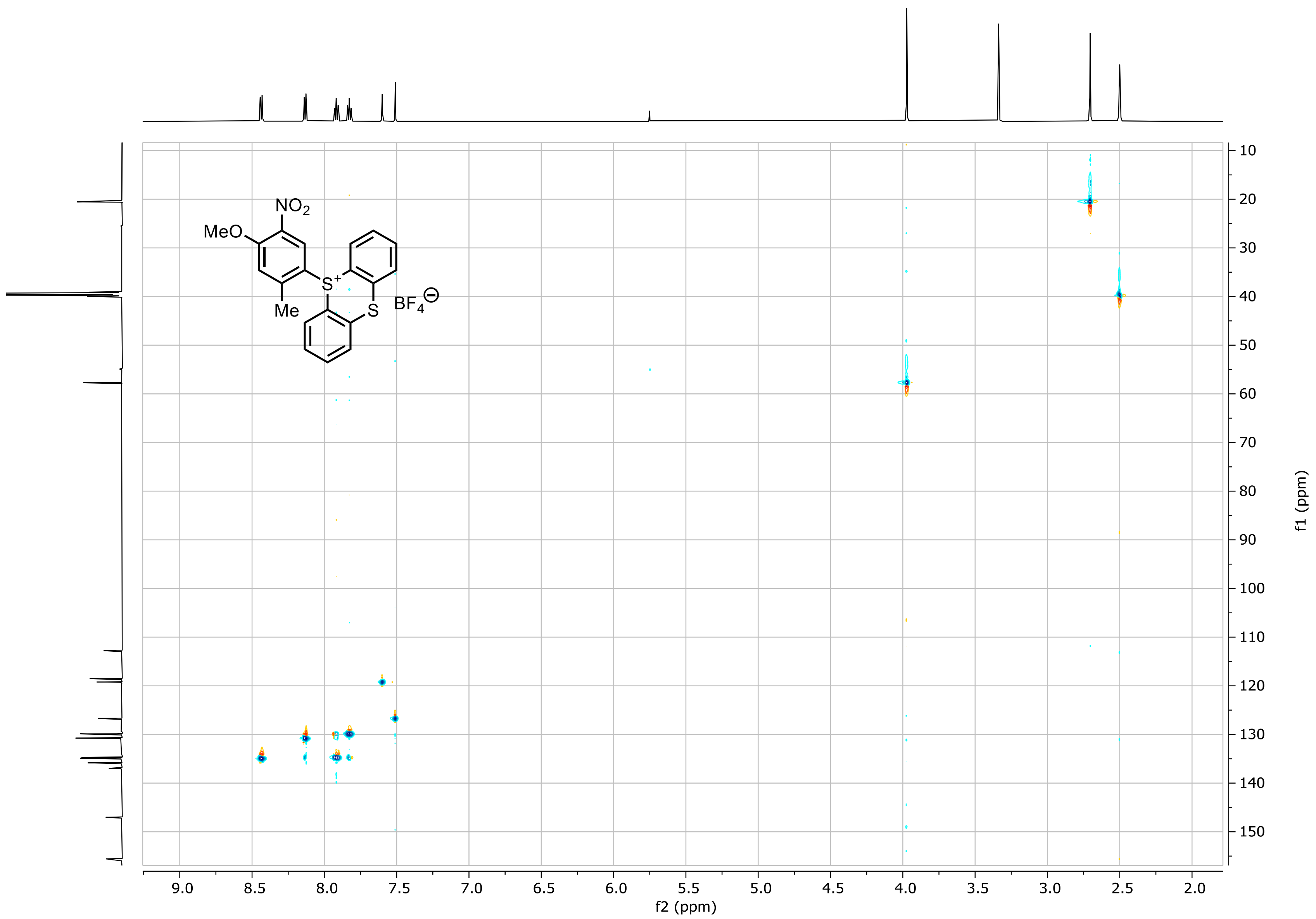

HMBC of 5-methyl-2-nitroanisole-derived thianthrenium tetrafluoroborate (TT-16)

CDCl$_3$, 600 MHz, 298 K

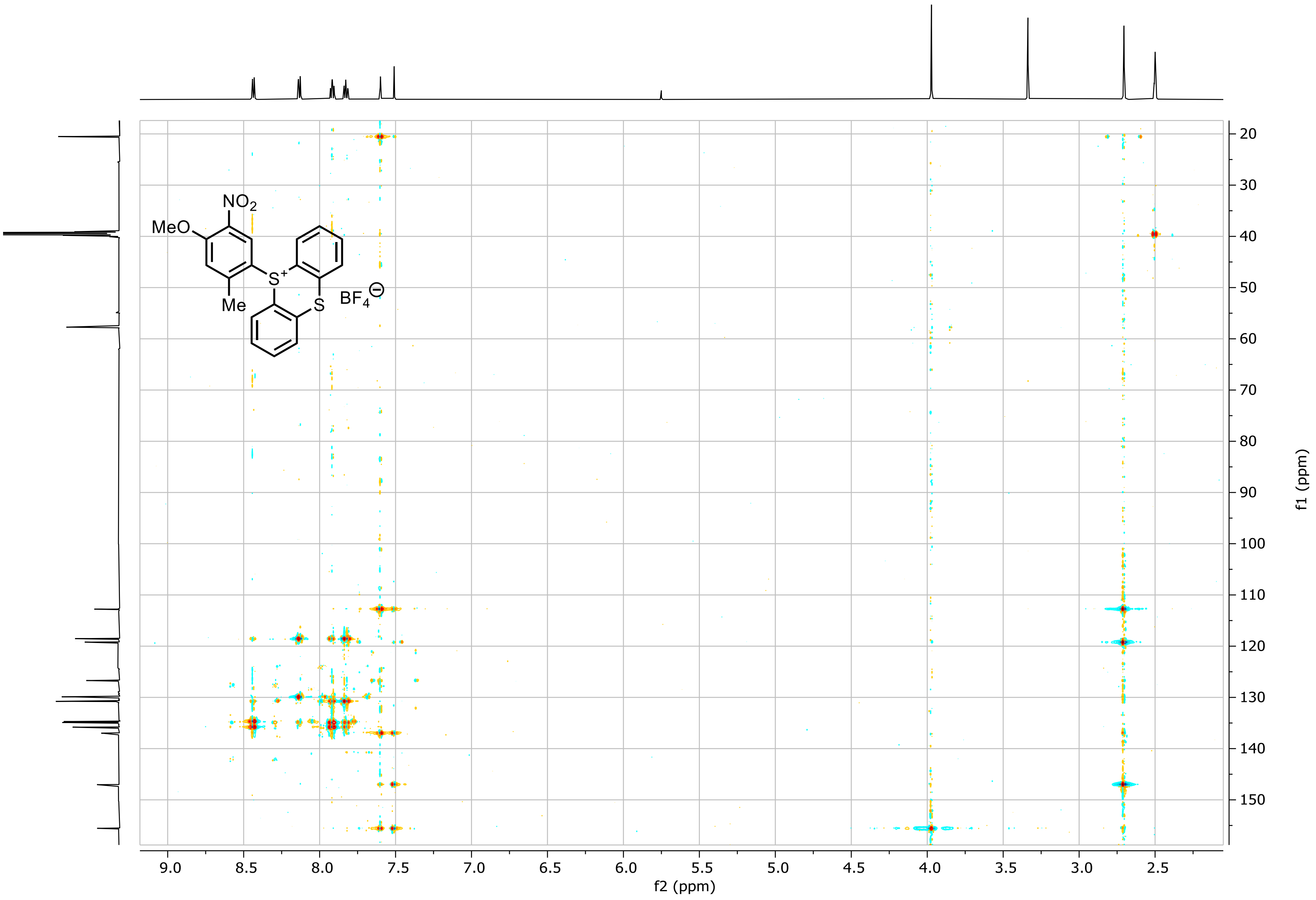

^{1}H NMR of 2-acetyl-1-methylpyrrole dibenzothiophenium tetrafluoroborate (TT-21)

CD_3CN, 500 MHz, 298 K

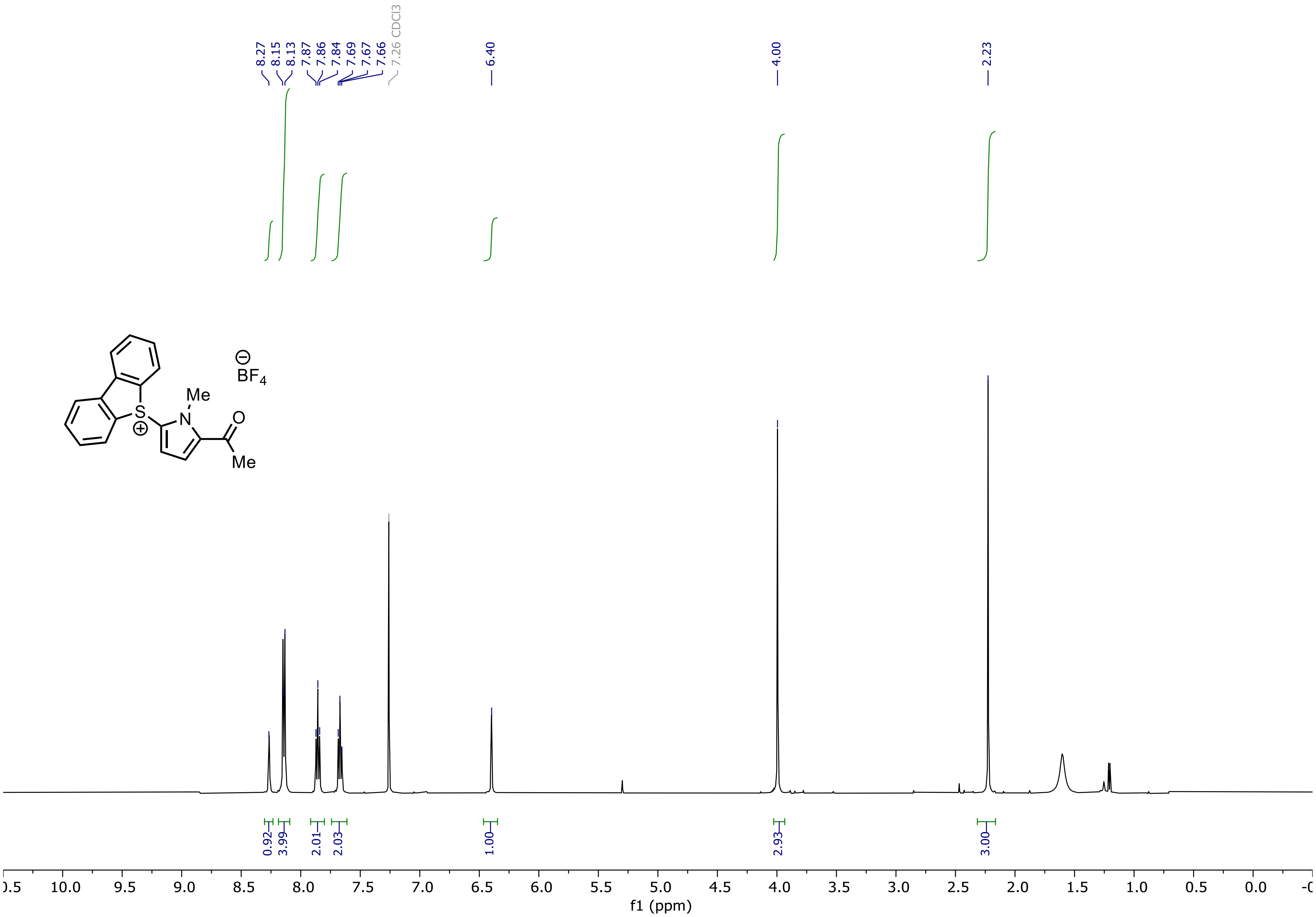

^{13}C NMR of 2-acetyl-1-methylpyrrole dibenzothiophenium tetrafluoroborate (TT-21)

CD_3CN, 126 MHz, 298 K

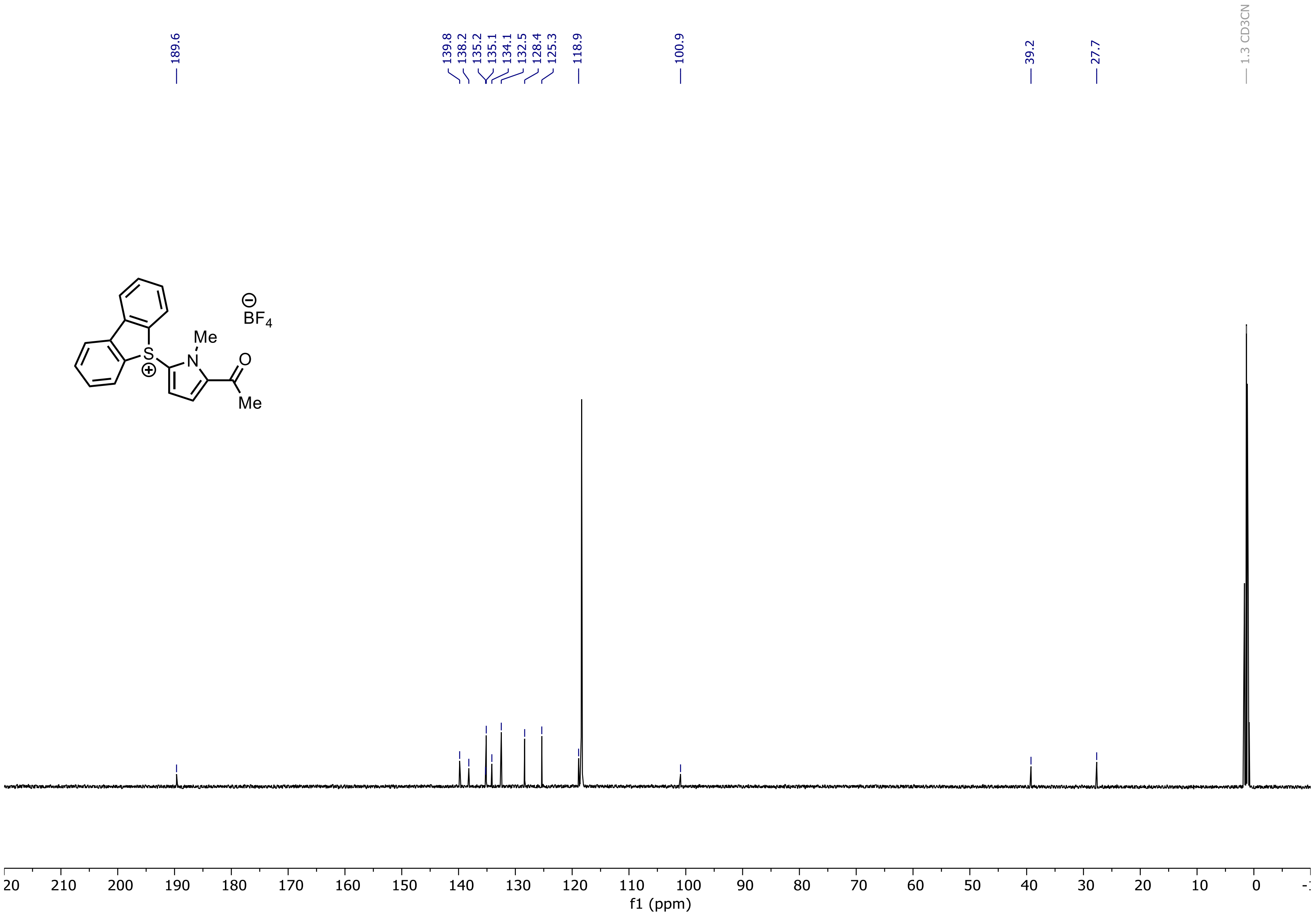

^{19}F NMR of 2-acetyl-1-methylpyrrole dibenzothiophenium tetrafluoroborate (TT-21)

$CDCl_3$, 471 MHz, 298 K

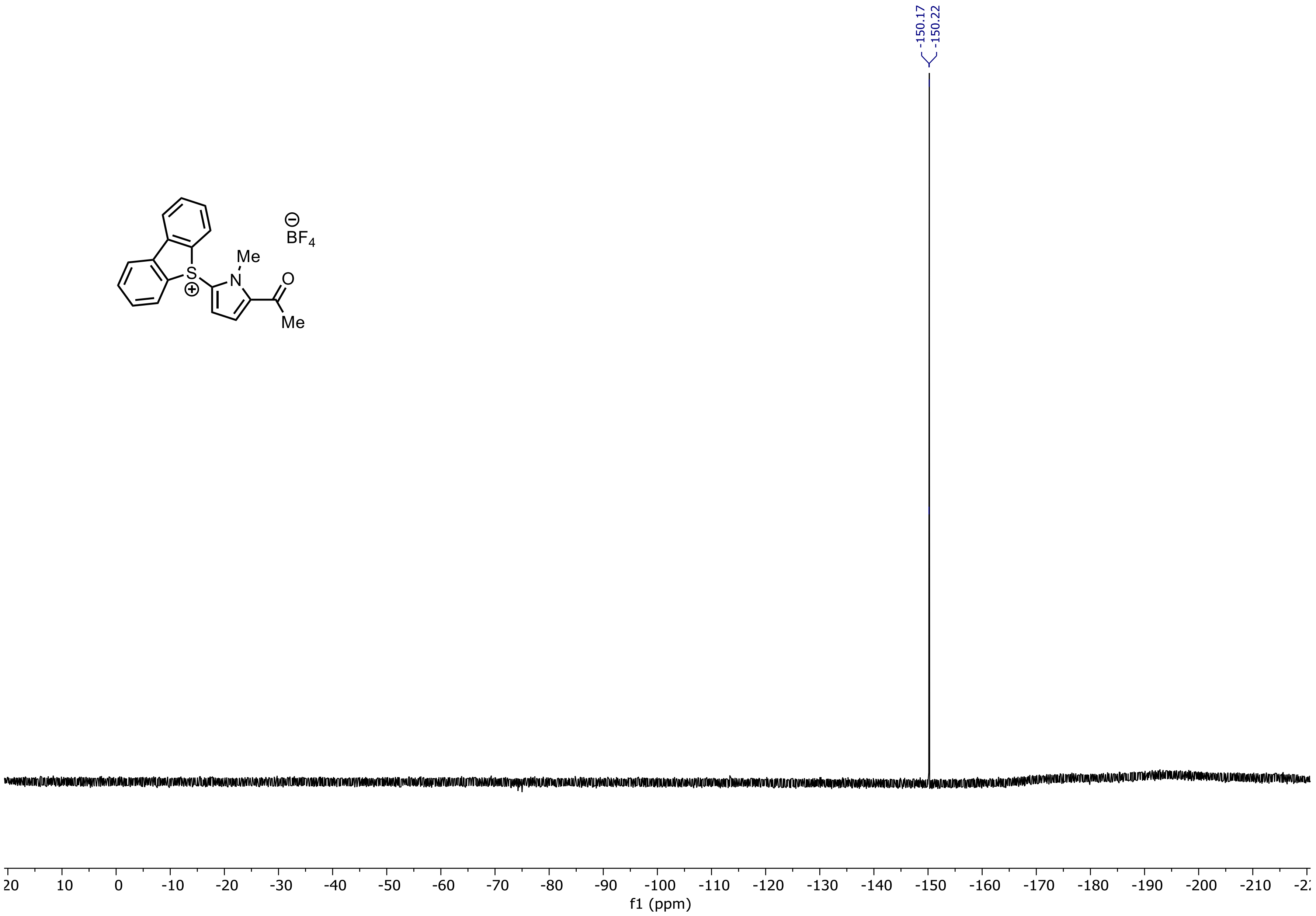

^{1}H NMR of ethyl 3-(furan-2-yl)propanoate dibenzothiophenium trifluoroacetate (TT-25)

$CDCl_3$, 500 MHz, 298 K

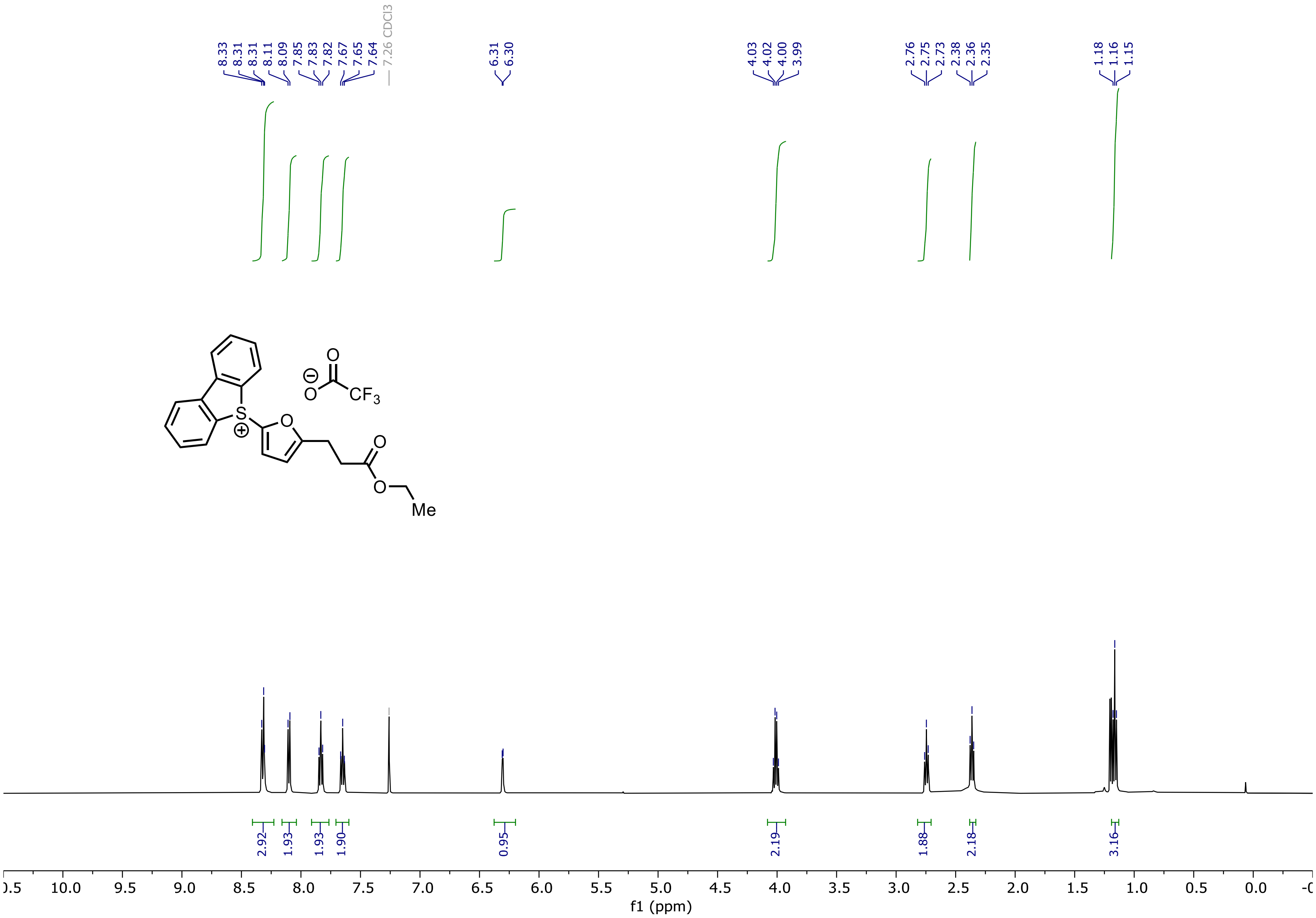

^{13}C NMR of ethyl 3-(furan-2-yl)propanoate dibenzothiophenium trifluoroacetate (TT-25)

CD_3CN, 126 MHz, 298 K

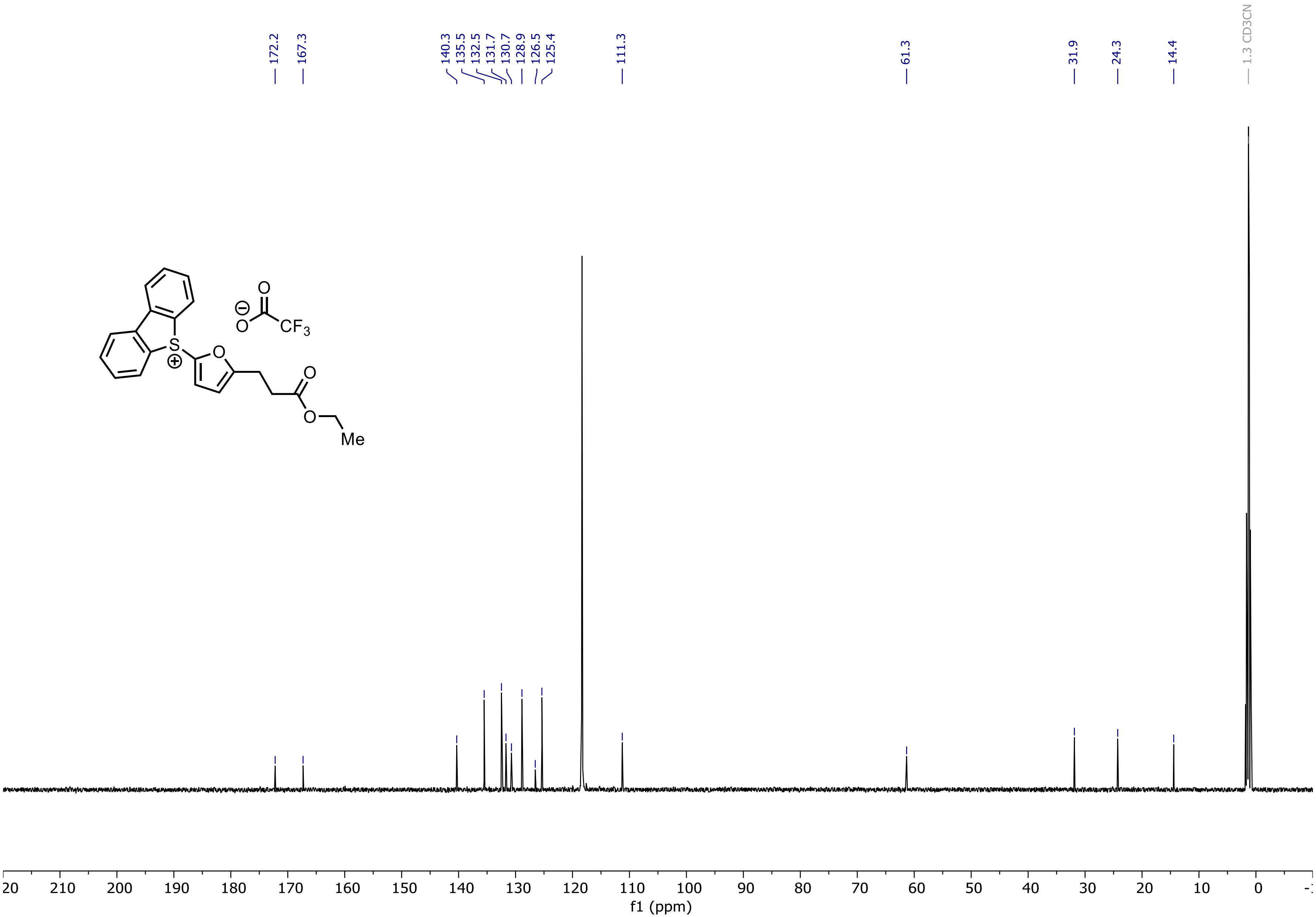

^{19}F NMR of ethyl 3-(furan-2-yl)propanoate dibenzothiophenium trifluoroacetate (TT-25)

$CDCl_3$, 471 MHz, 298 K

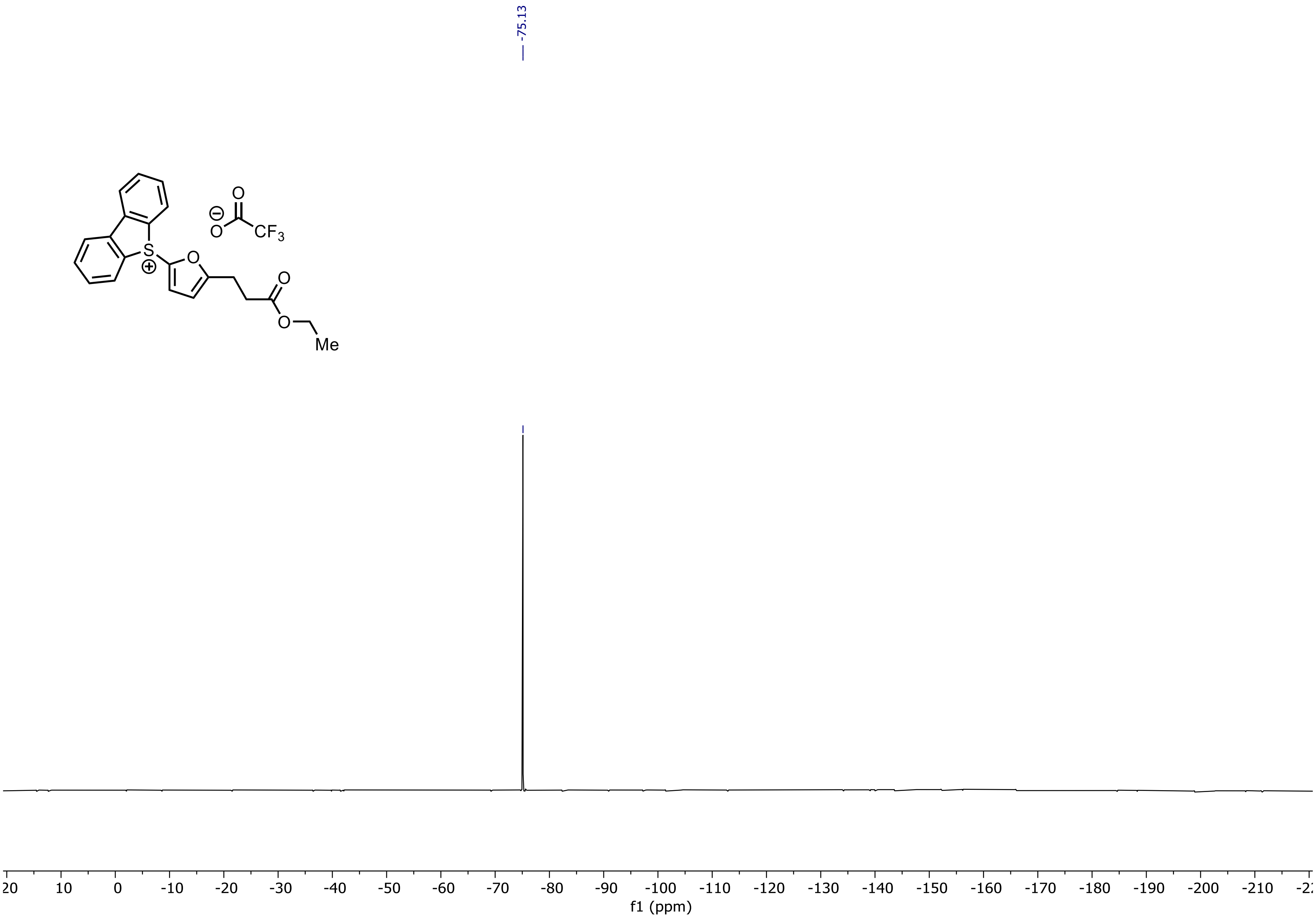

^{1}H NMR of 1-methyl pyrazole-derived dibenzothiophenium salt (TT-26)

$CDCl_3$, 500 MHz, 298 K

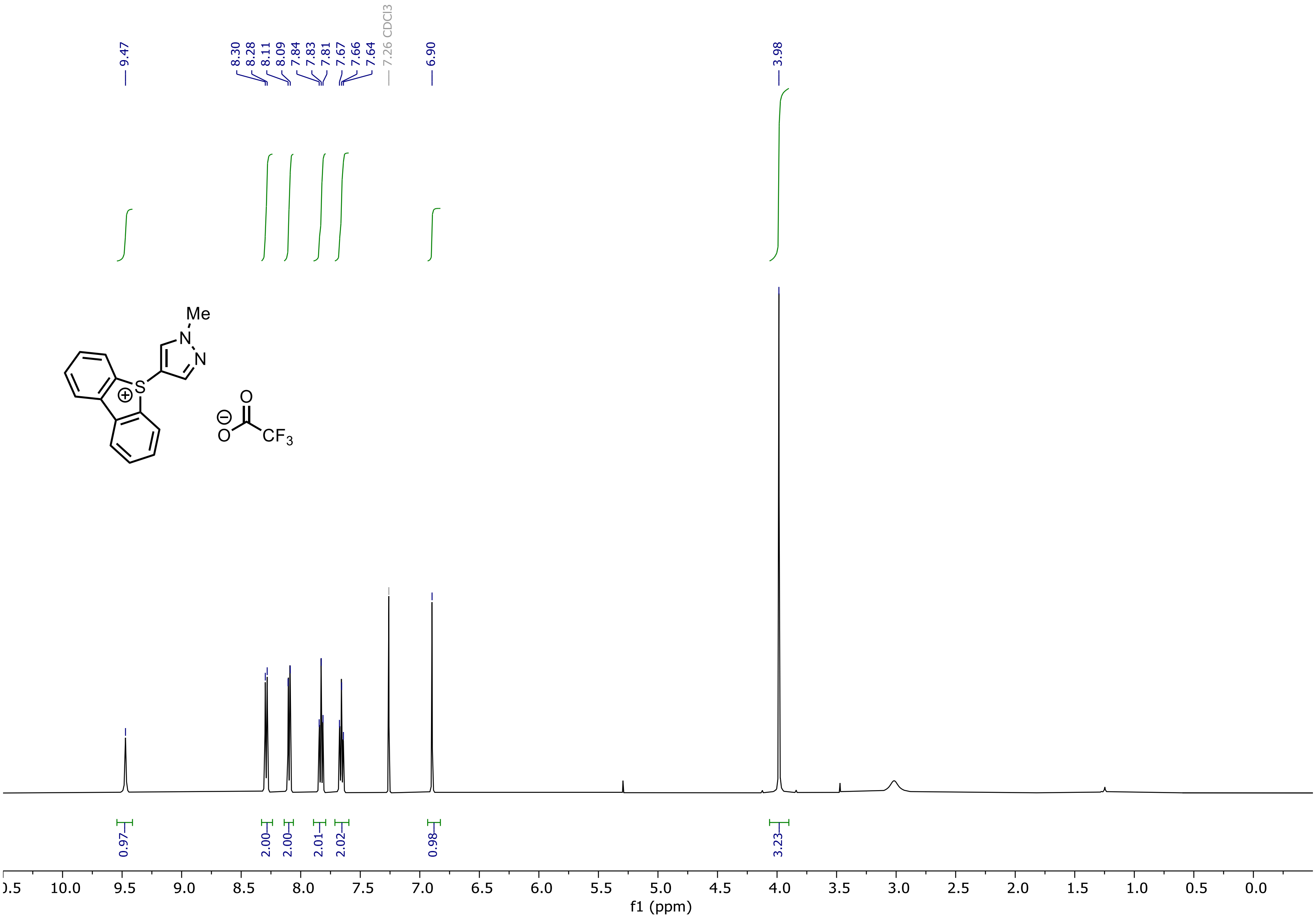

^{13}C NMR of 1-methyl pyrazole-derived dibenzothiophenium salt (TT-26)

$CDCl_3$, 126 MHz, 298 K

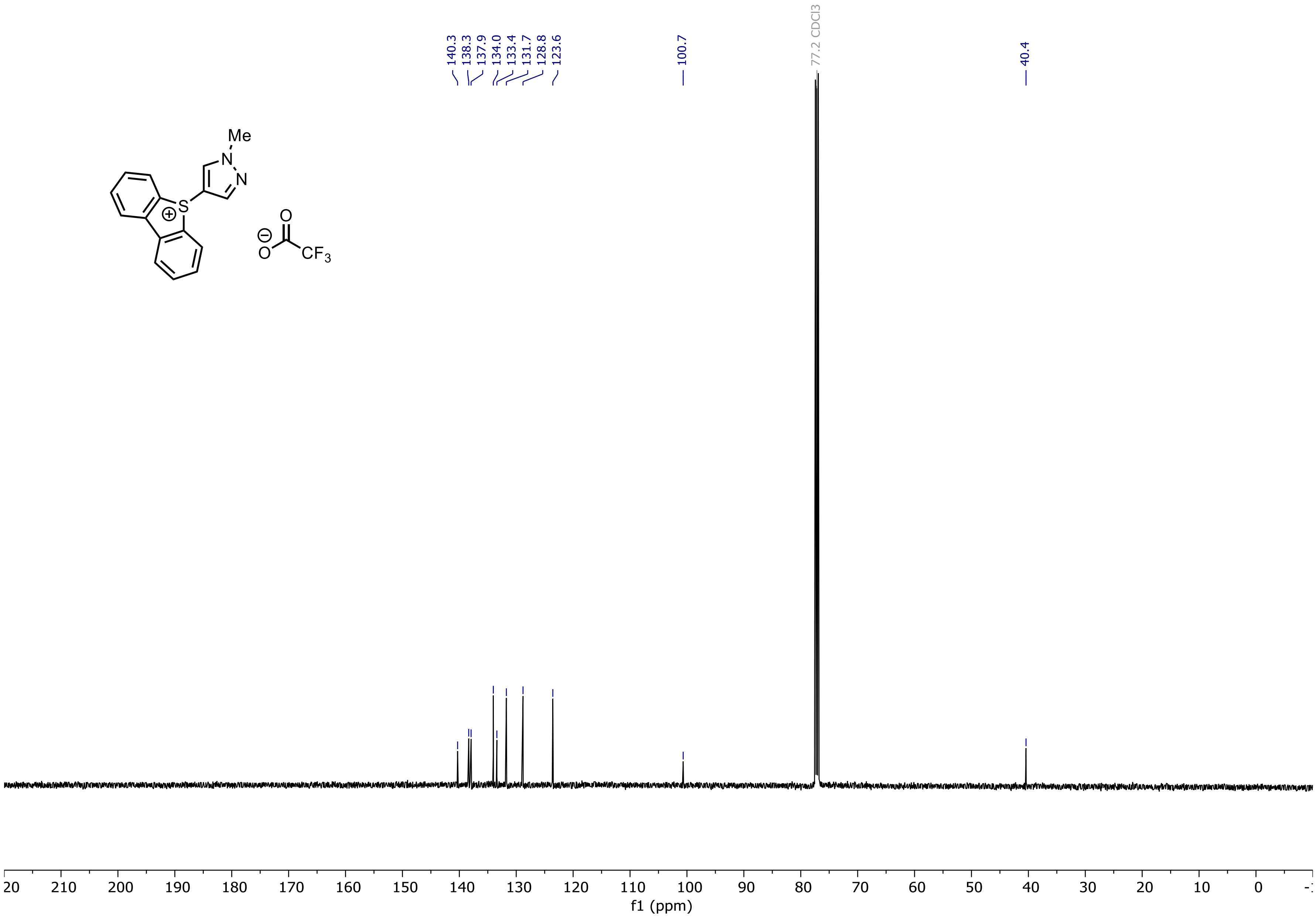

^{19}F NMR of 1-methyl pyrazole-derived dibenzothiophenium salt (TT-26)

$CDCl_3$, 471 MHz, 298 K

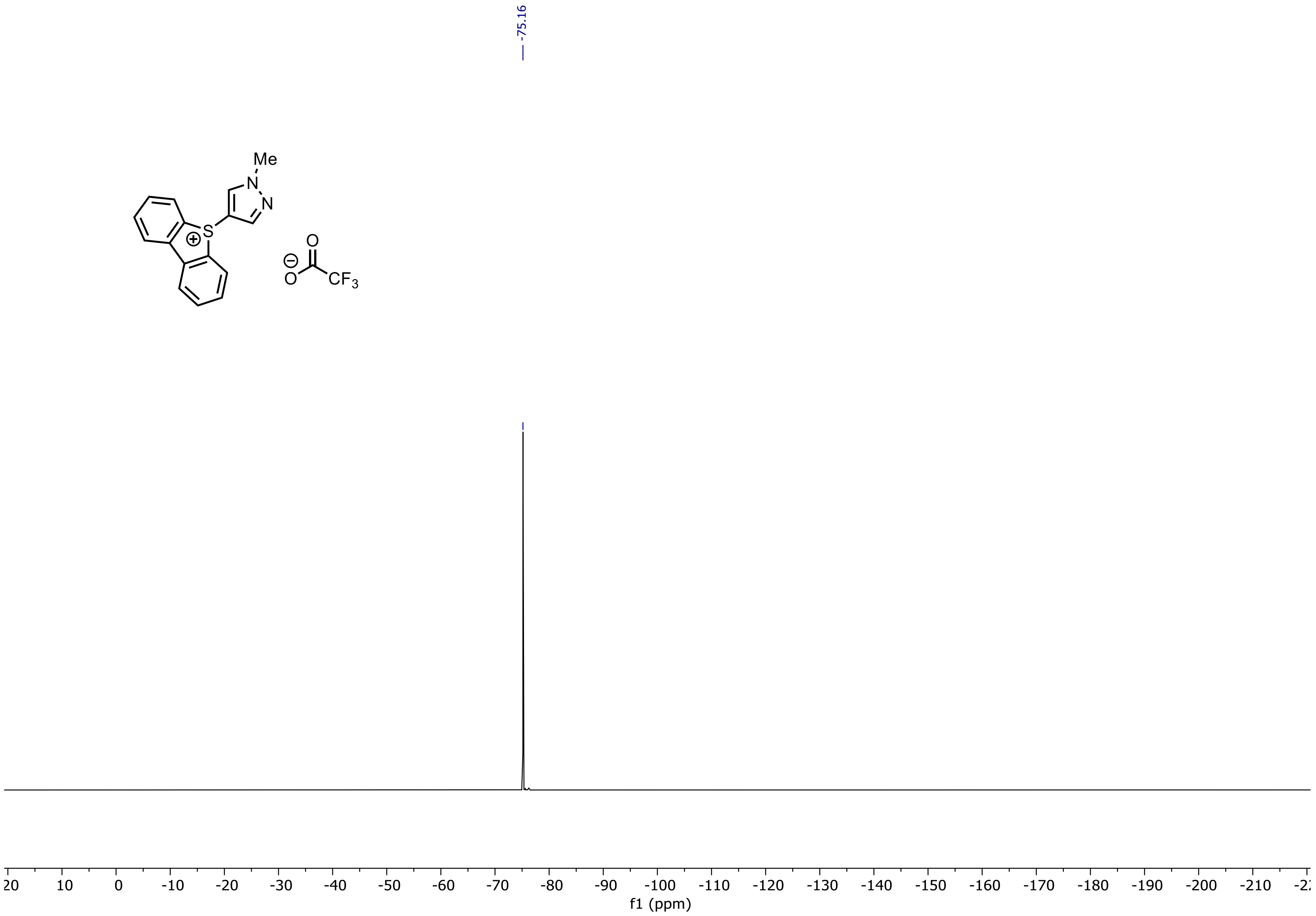

^{1}H NMR of salicin pentaacetate *N*-methyl imidazole derivative (1a)

CDCl$_3$, 500 MHz, 298 K

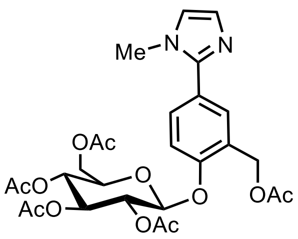

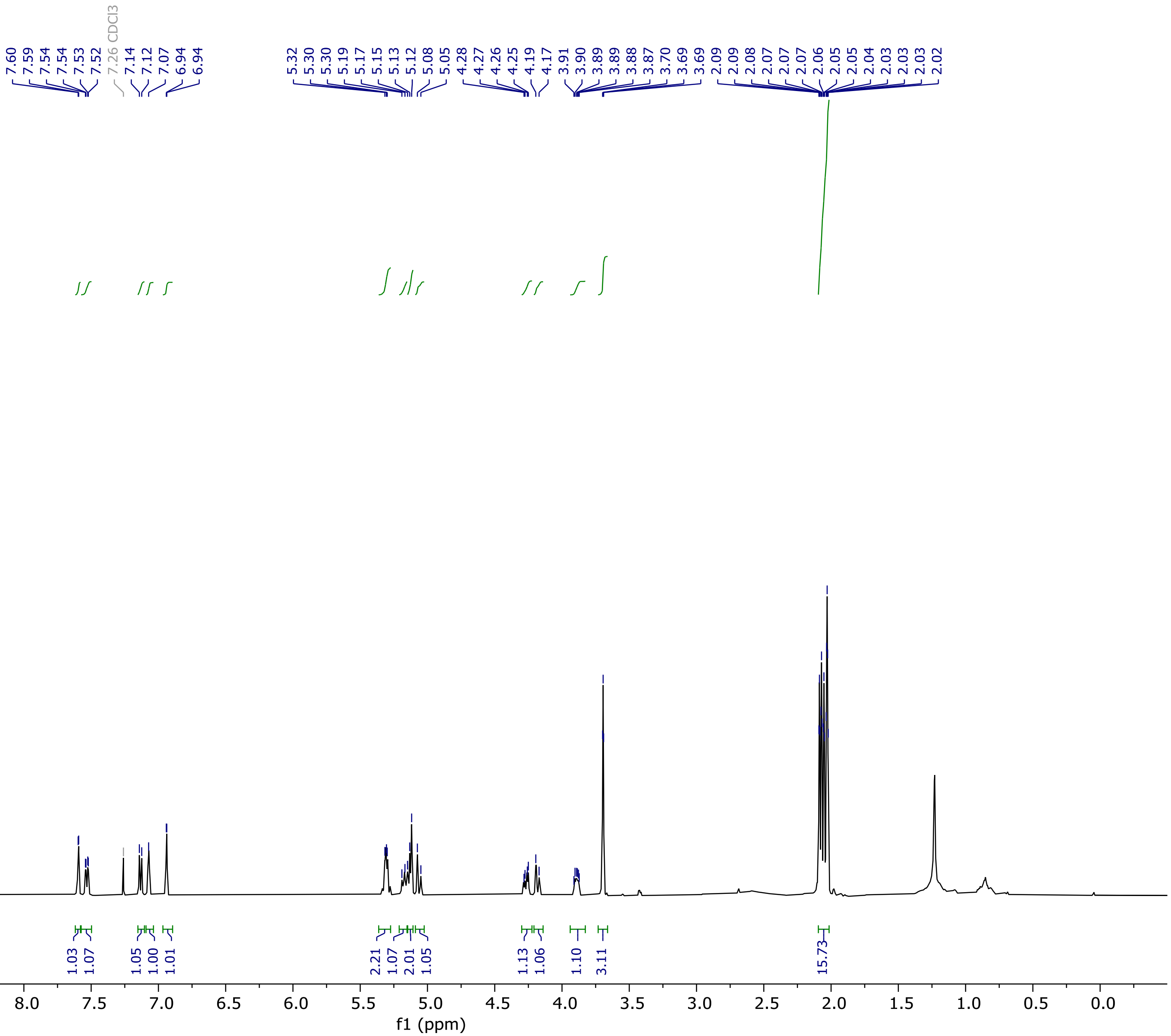

^{13}C NMR of salicin pentaacetate *N*-methyl imidazole derivative (1a)

$CDCl_3$, 126 MHz, 298 K

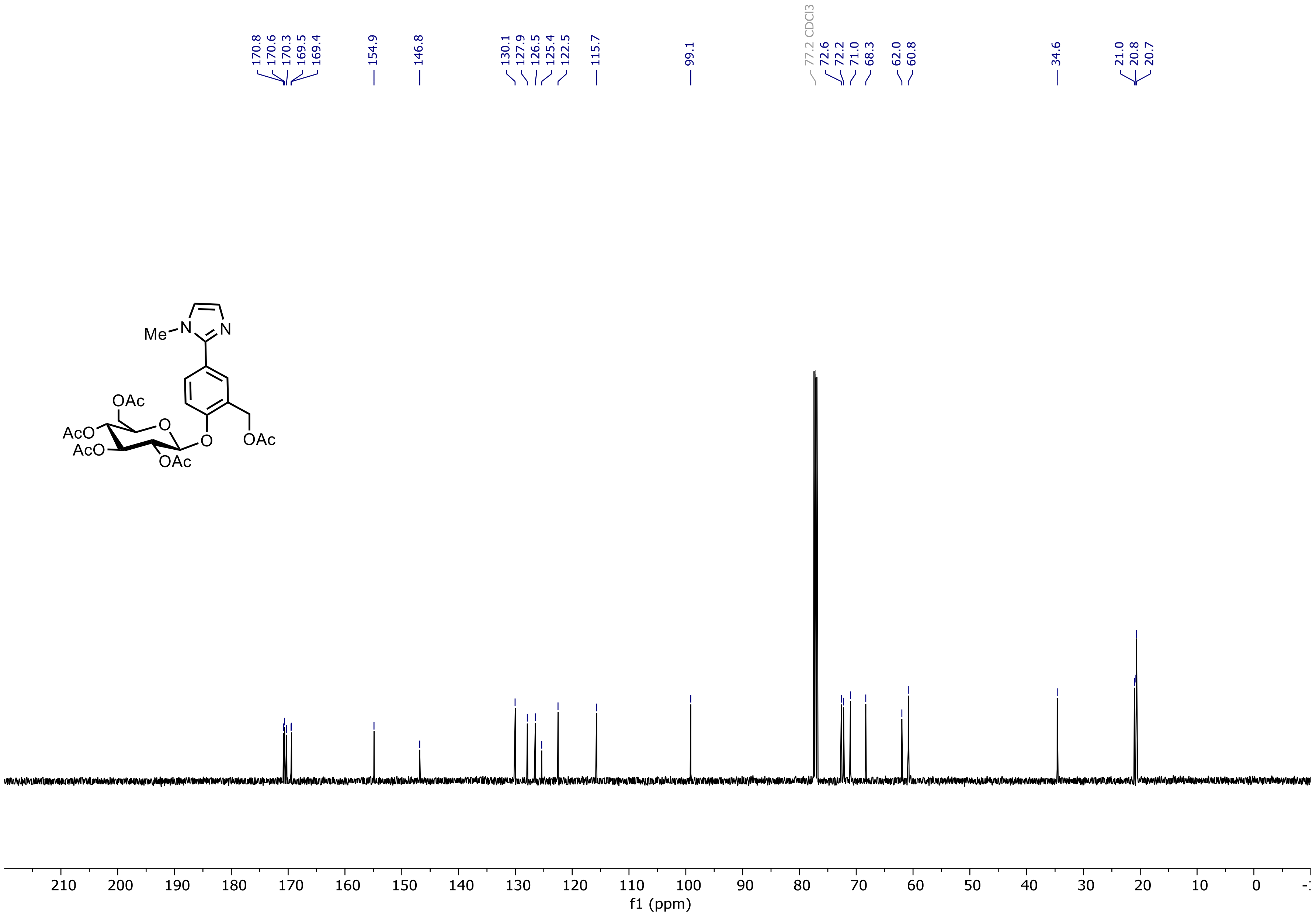

^{1}H NMR of salicin pentaacetate *N*-methyl imidazole derivative (1b)

CDCl3, 500 MHz, 298 K

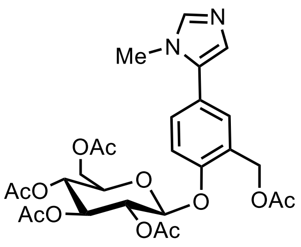

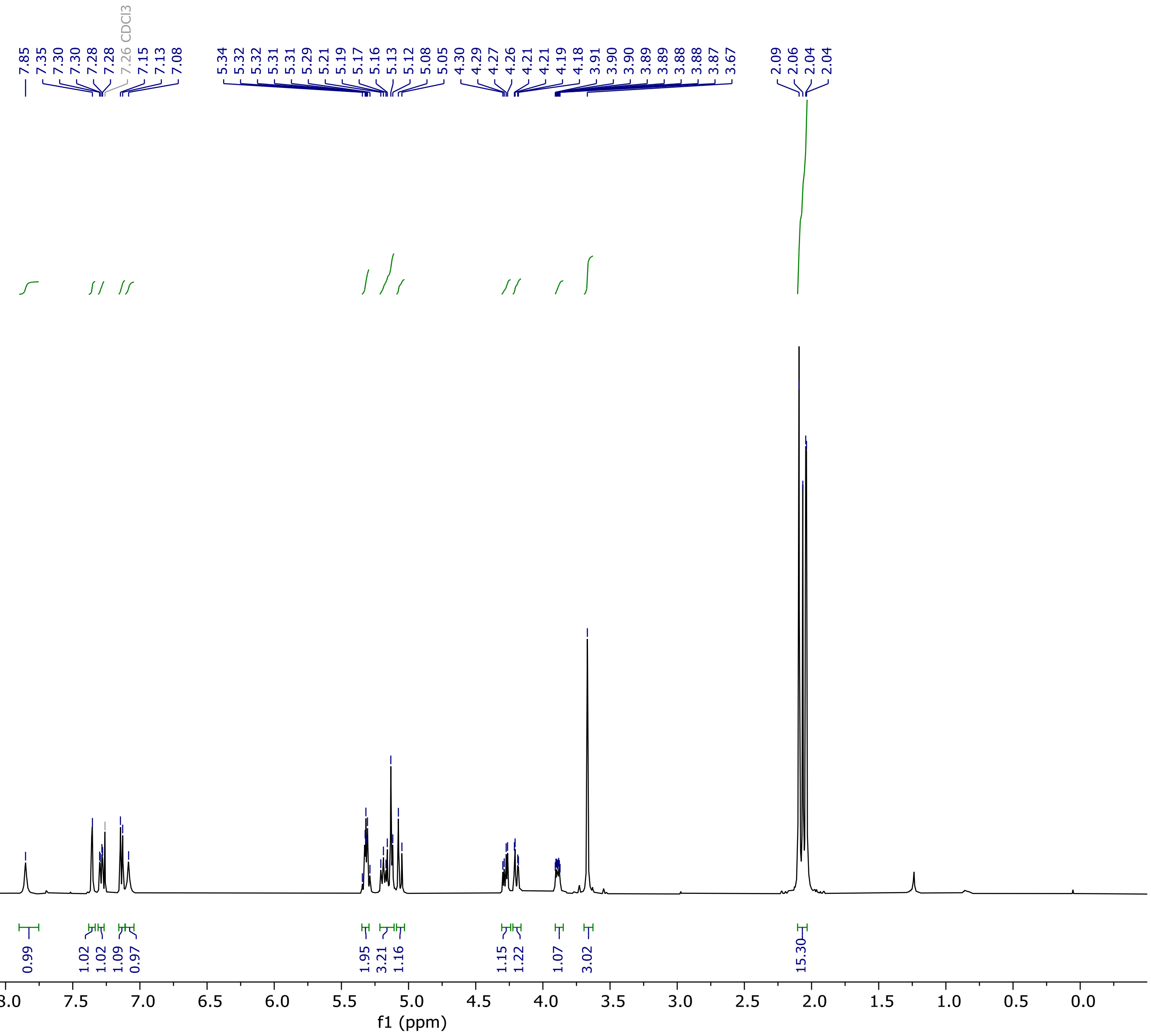

^{13}C NMR of salicin pentaacetate *N*-methyl imidazole derivative (1b)

$CDCl_3$, 126 MHz, 298 K

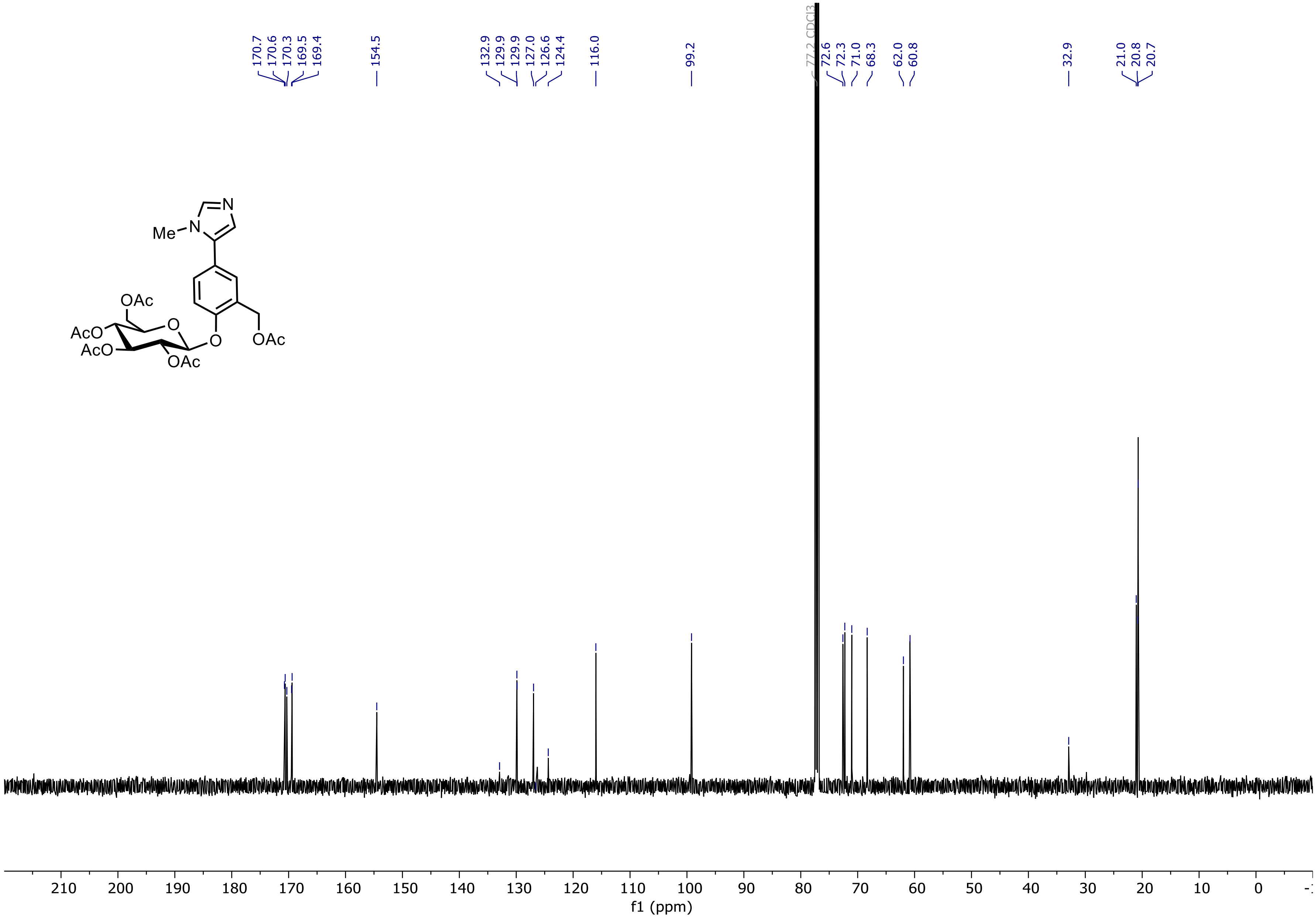

^{1}H NMR of 8-butoxy-5-(pyrazin-2-yl)quinoline (2)

$CDCl_3$, 500 MHz, 298 K

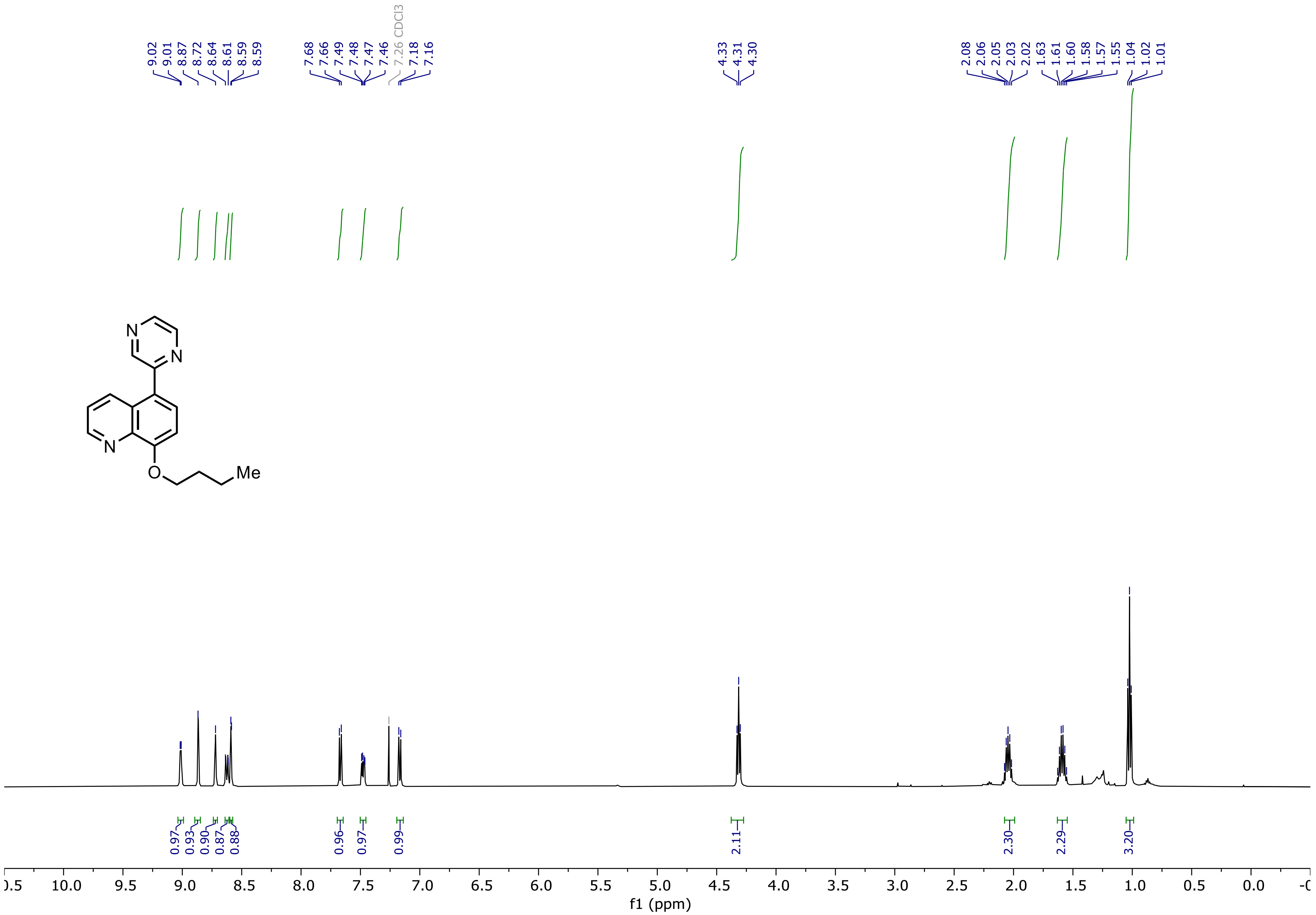

^{13}C NMR of 8-butoxy-5-(pyrazin-2-yl)quinoline (2)

$CDCl_3$, 126 MHz, 298 K

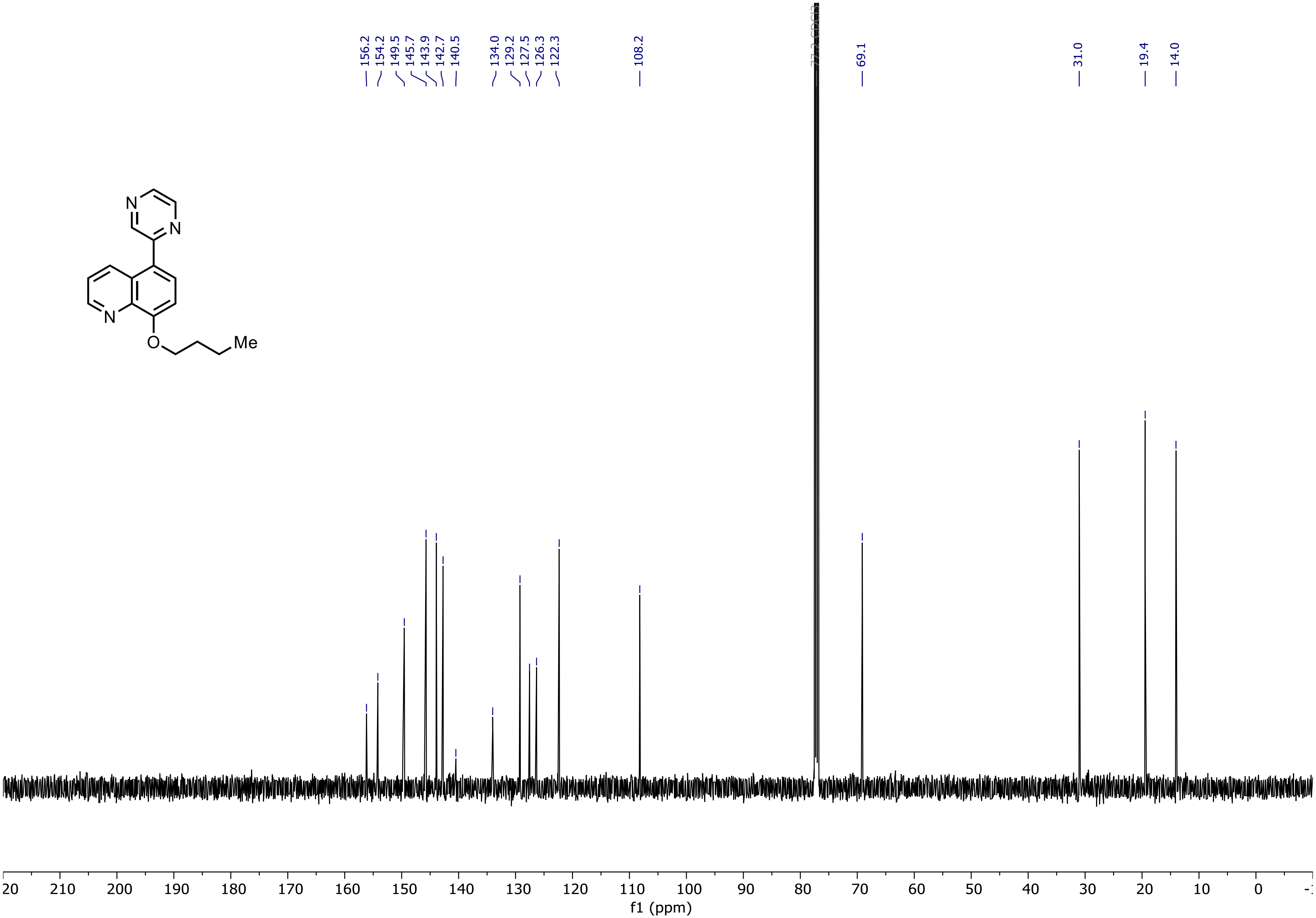

^{1}H NMR of 2-([1,1'-biphenyl]-4-yl)pyrazine (3)

$CDCl_3$, 500 MHz, 298 K

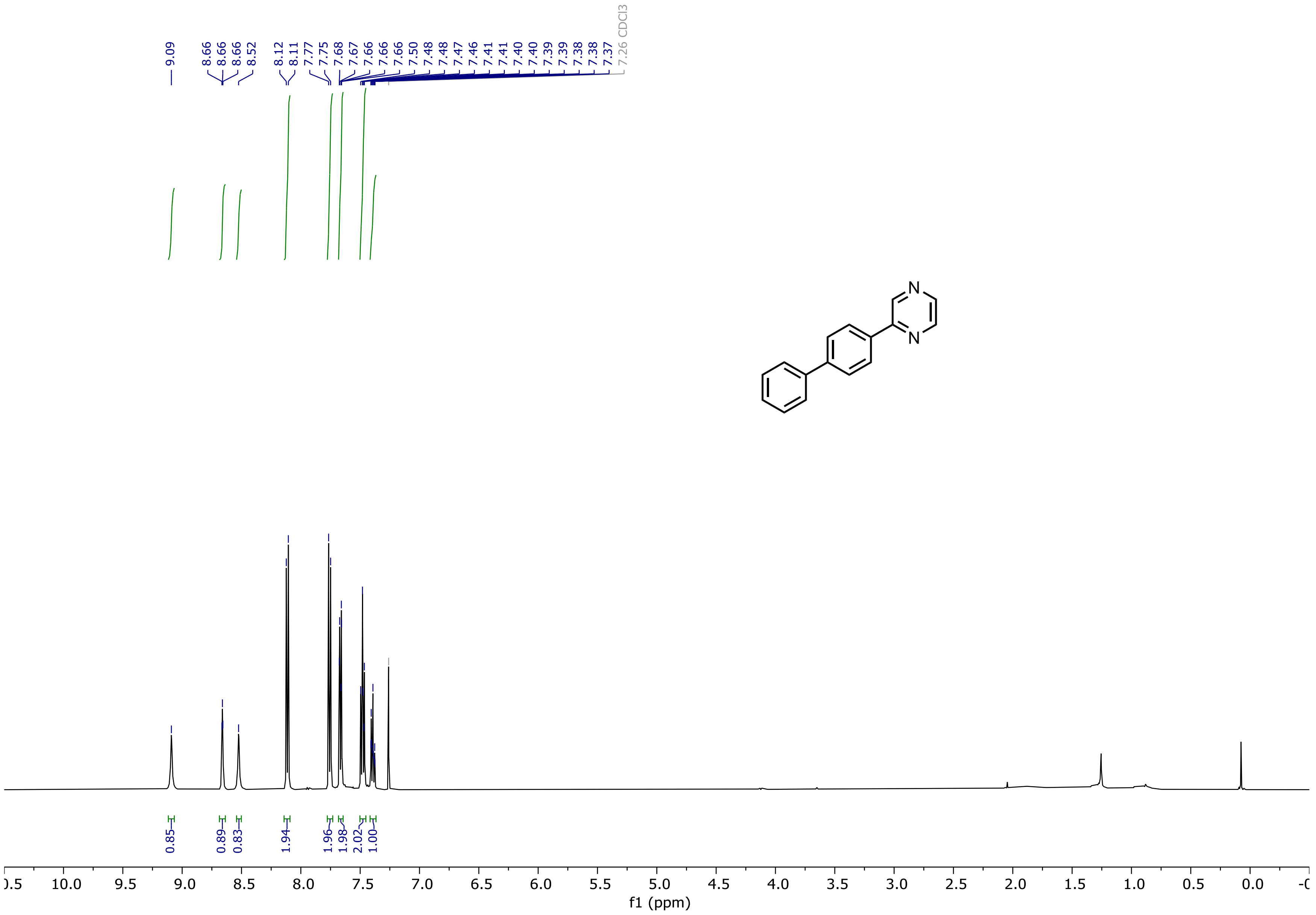

^{13}C NMR of 2-([1,1'-biphenyl]-4-yl)pyrazine (3)

$CDCl_3$, 126 MHz, 298 K

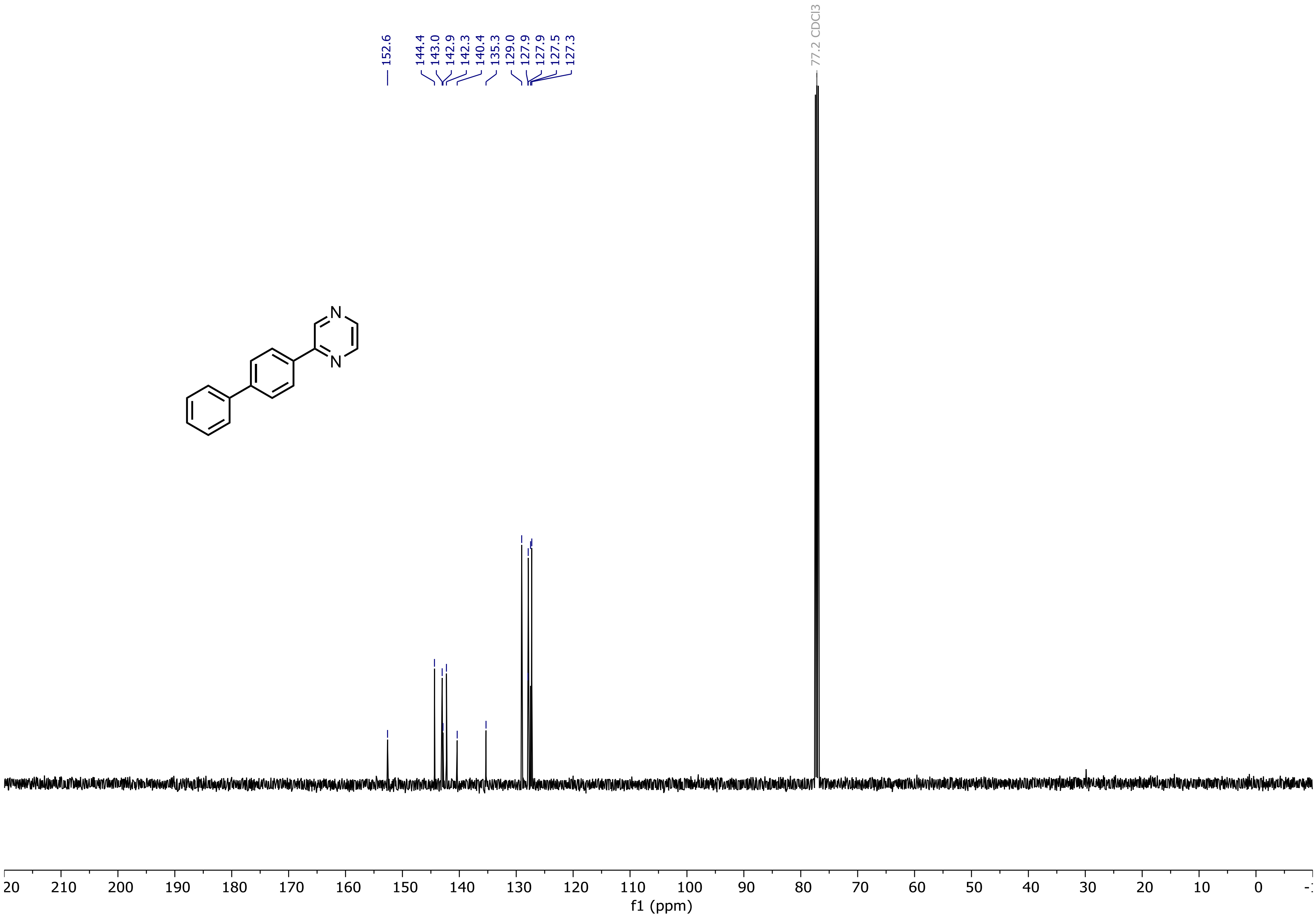

^{1}H NMR of 2-(4-(4-bromophenoxy)phenyl)pyrimidine (4a)

CDCl₃, 500 MHz, 298 K

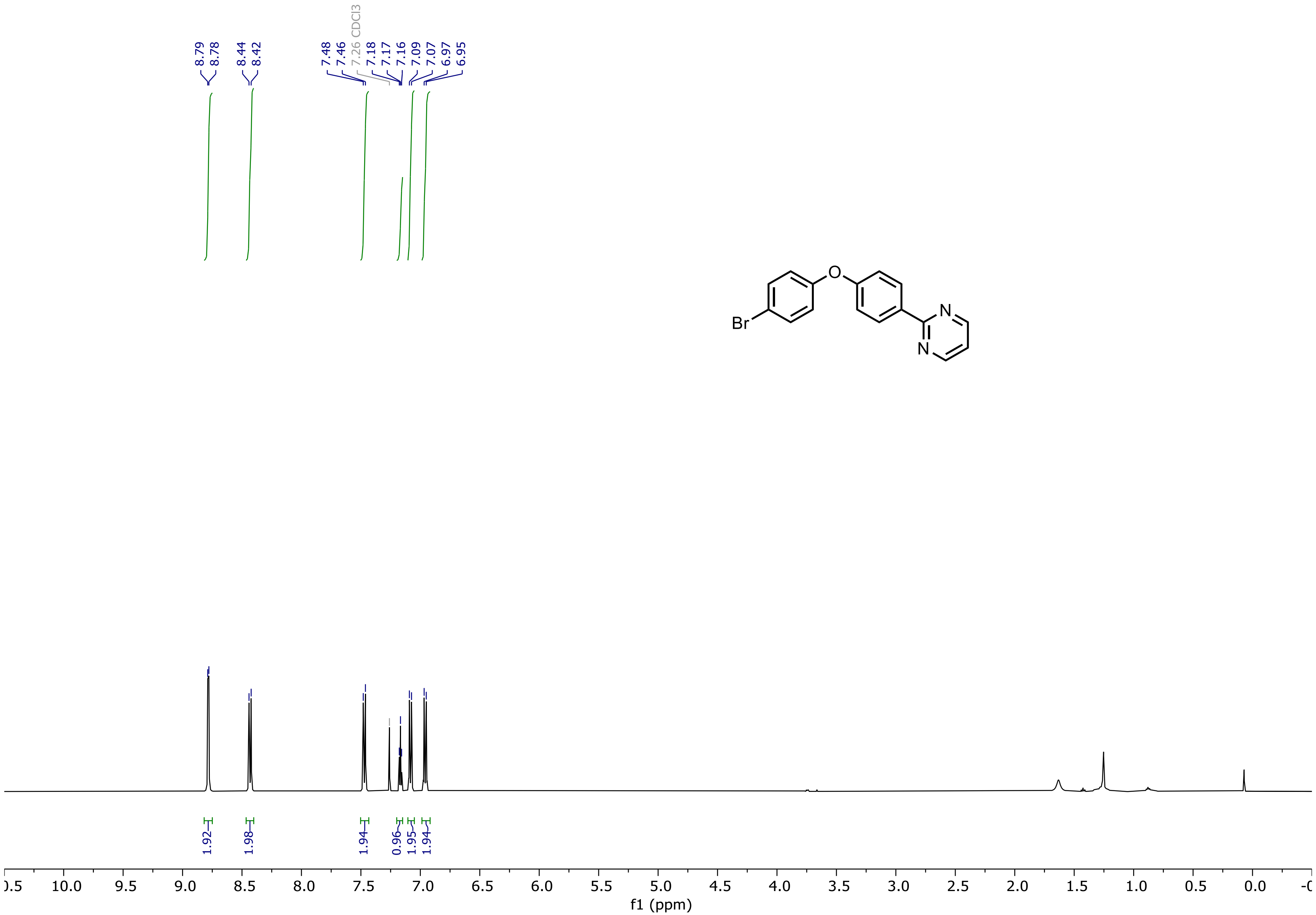

^{13}C NMR of 2-(4-(4-bromophenoxy)phenyl)pyrimidine (4a)

$CDCl_3$, 151 MHz, 298 K

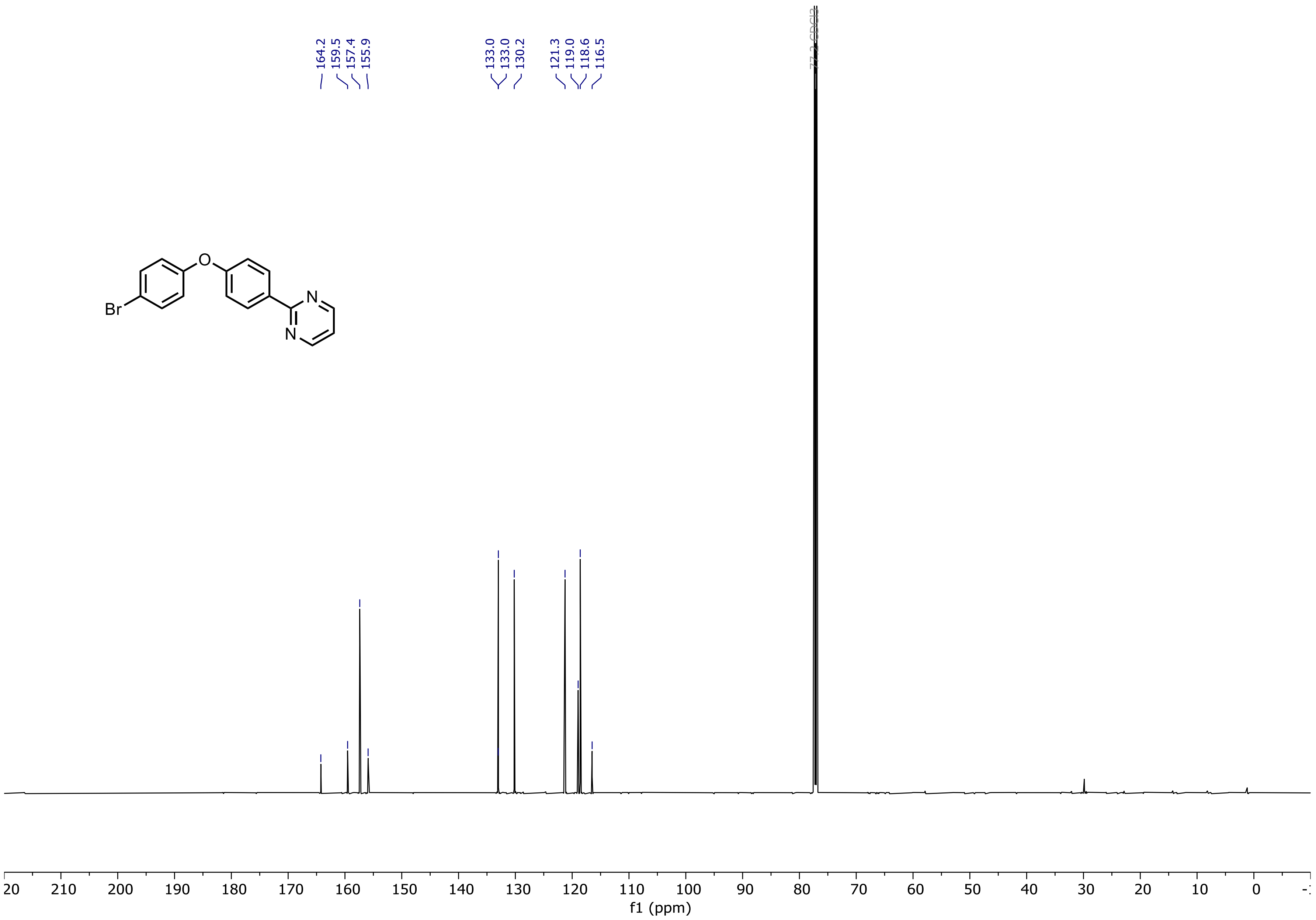

^{1}H NMR of 5-(4-(4-bromophenoxy)phenyl)pyrimidine (4b)

$CDCl_3$, 500 MHz, 298 K

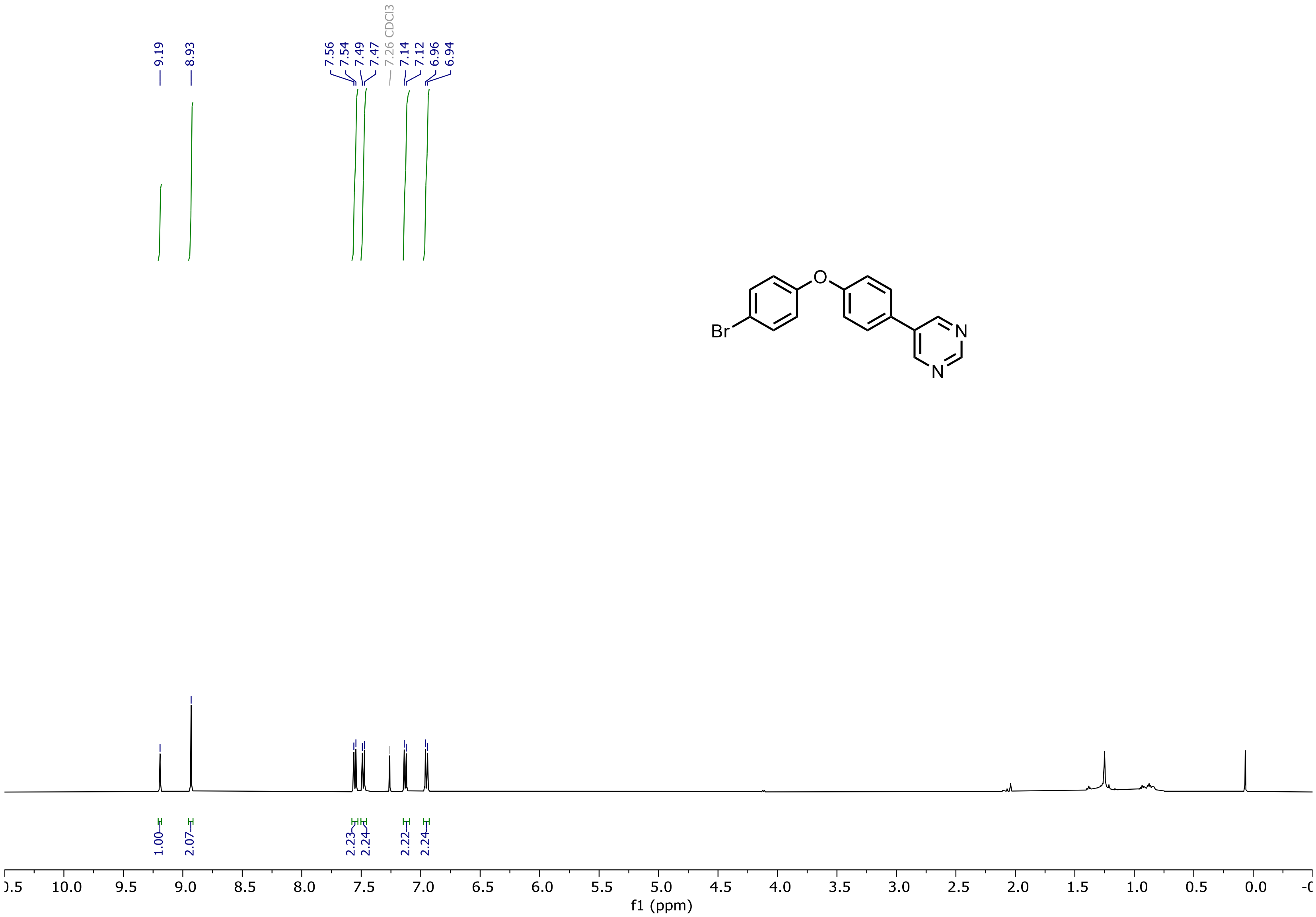

^{13}C NMR of 5-(4-(4-bromophenoxy)phenyl)pyrimidine (4b)

$CDCl_3$, 151 MHz, 298 K

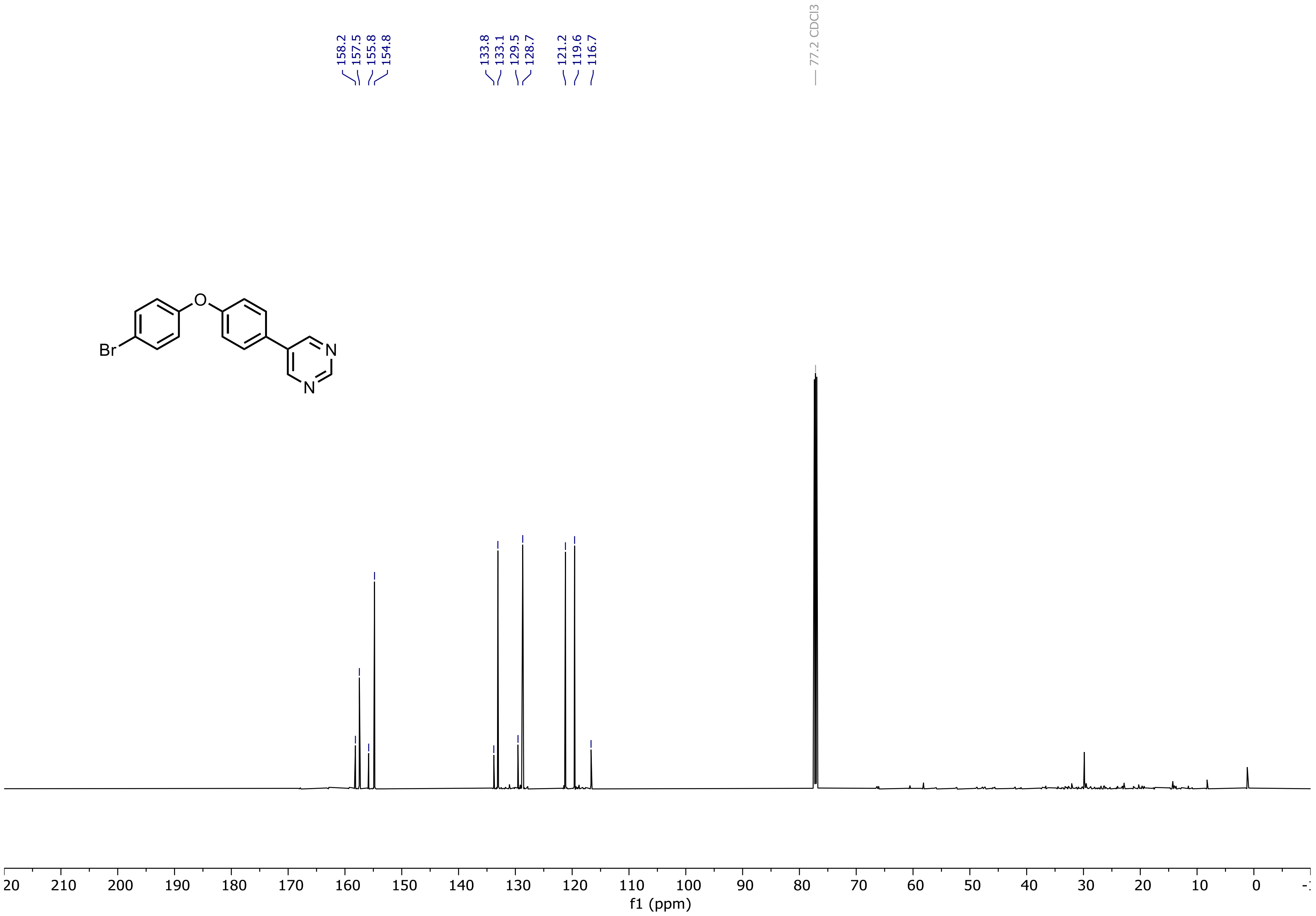

^{1}H NMR of 4-(4-(4-bromophenoxy)phenyl)pyrimidine (4c)

$CDCl_3$, 500 MHz, 298 K

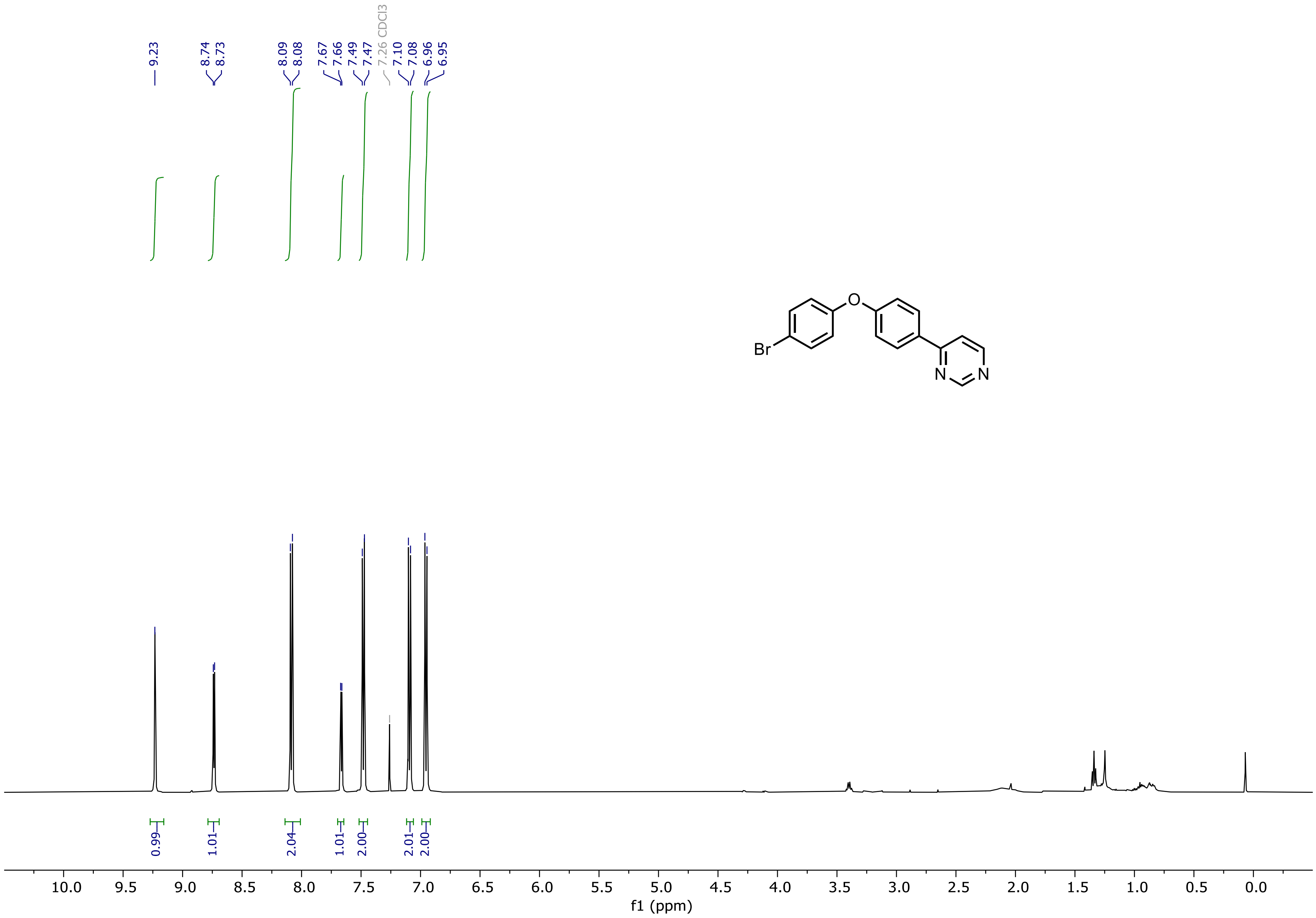

^{13}C NMR of 4-(4-(4-bromophenoxy)phenyl)pyrimidine (4c)

$CDCl_3$, 126 MHz, 298 K

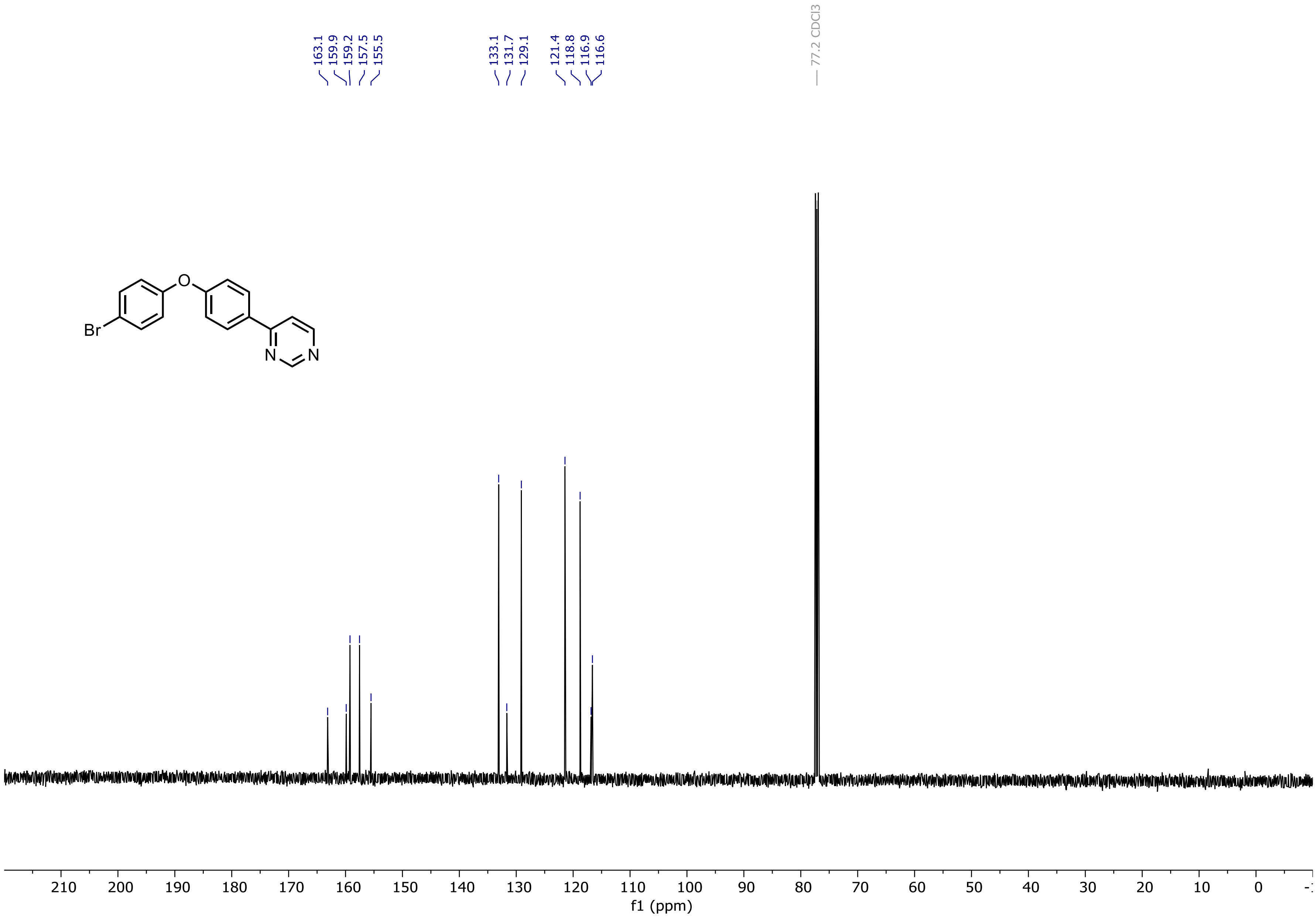

^{1}H NMR of boscalid pyrazine derivative (5)

$CDCl_3$, 500 MHz, 298 K

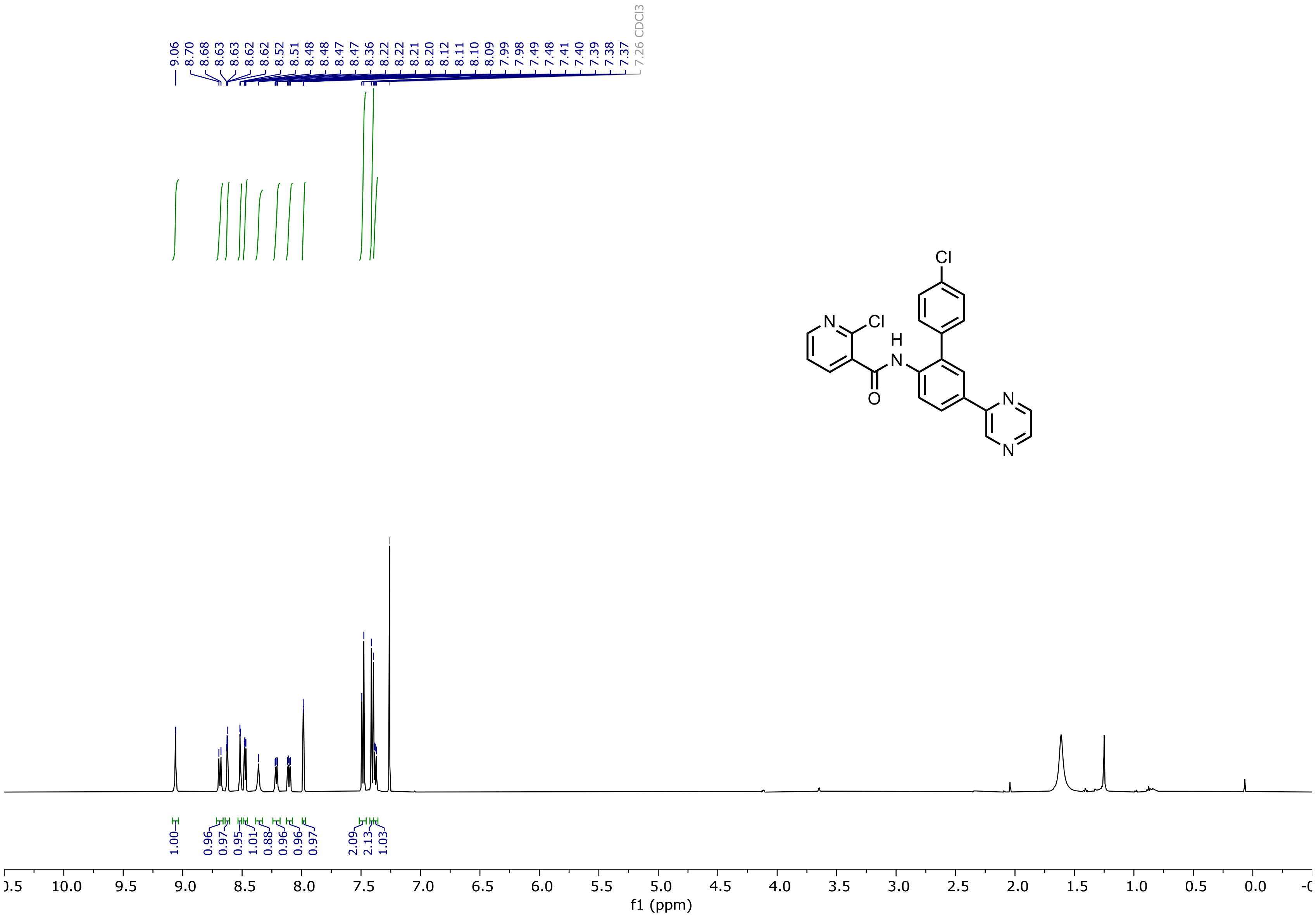

^{13}C NMR of boscalid pyrazine derivative (5)

$CDCl_3$, 151 MHz, 298 K

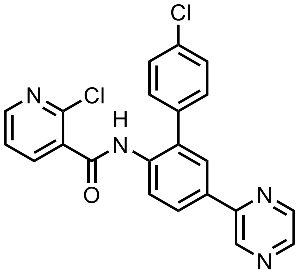

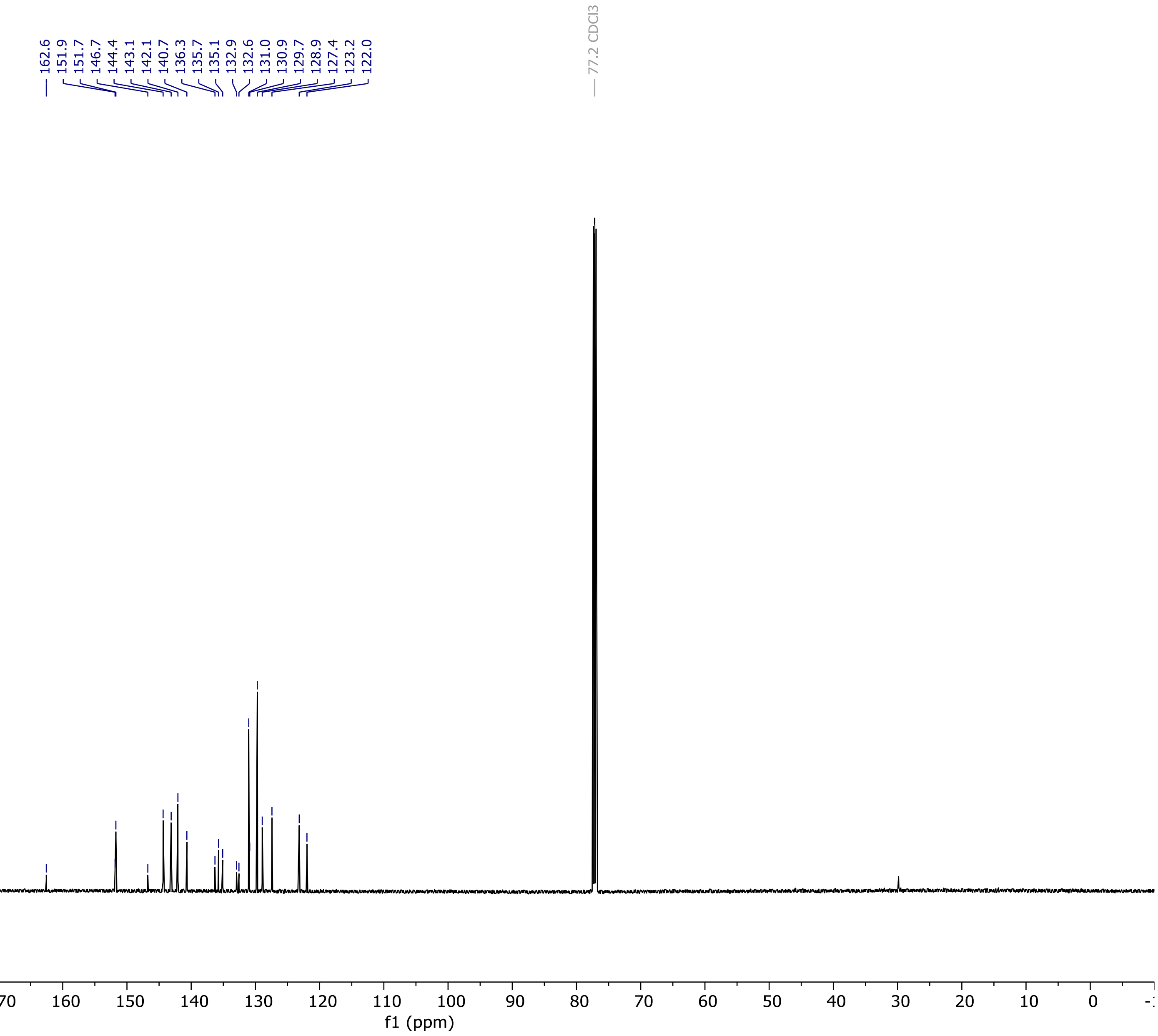

^{1}H NMR of 2-(5-bromo-6-butoxynaphthalen-2-yl)pyridine (6a)

$CDCl_3$, 300 MHz, 298 K

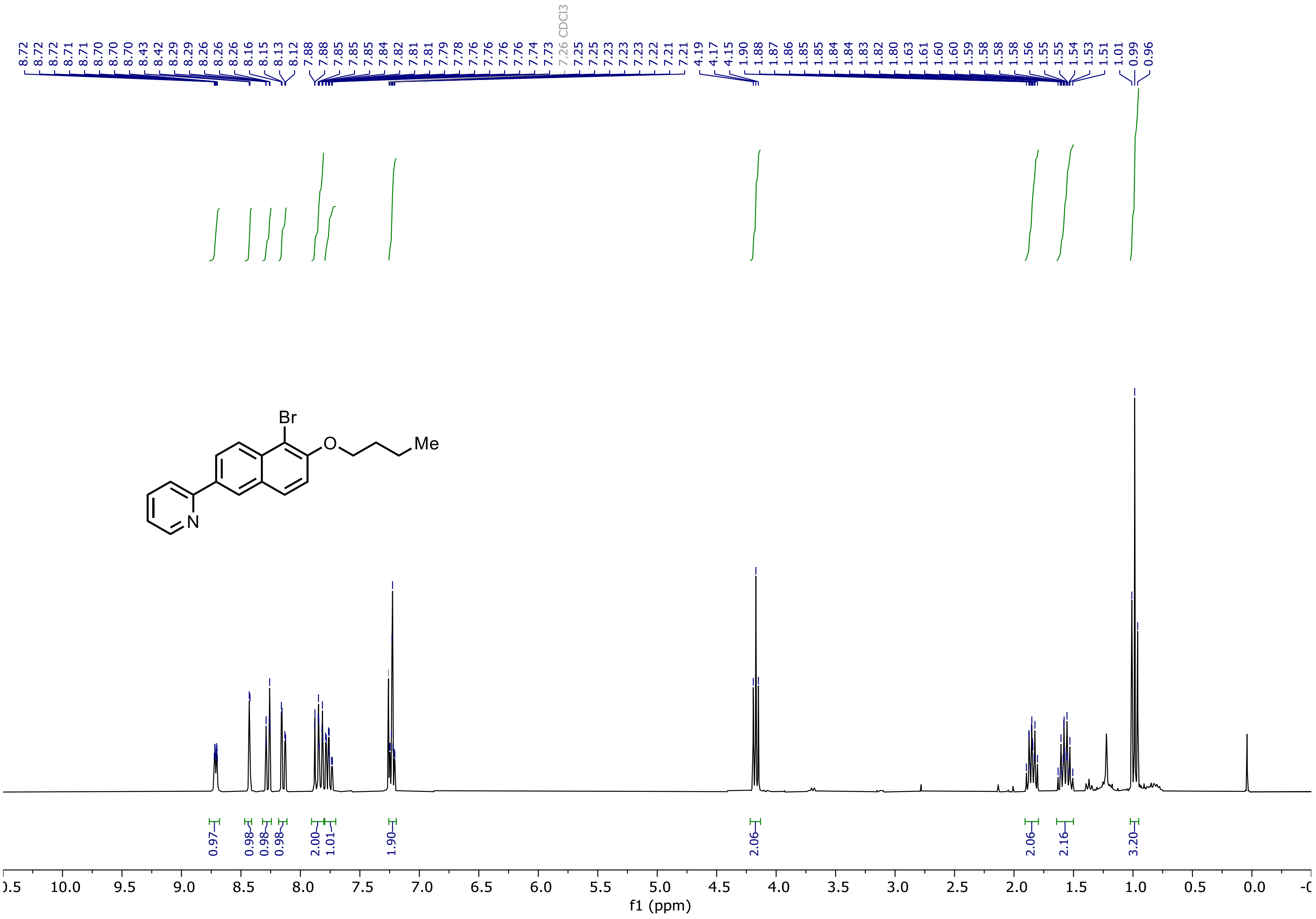

^{13}C NMR of 2-(5-bromo-6-butoxynaphthalen-2-yl)pyridine (6a)

$CDCl_3$, 126 MHz, 298 K

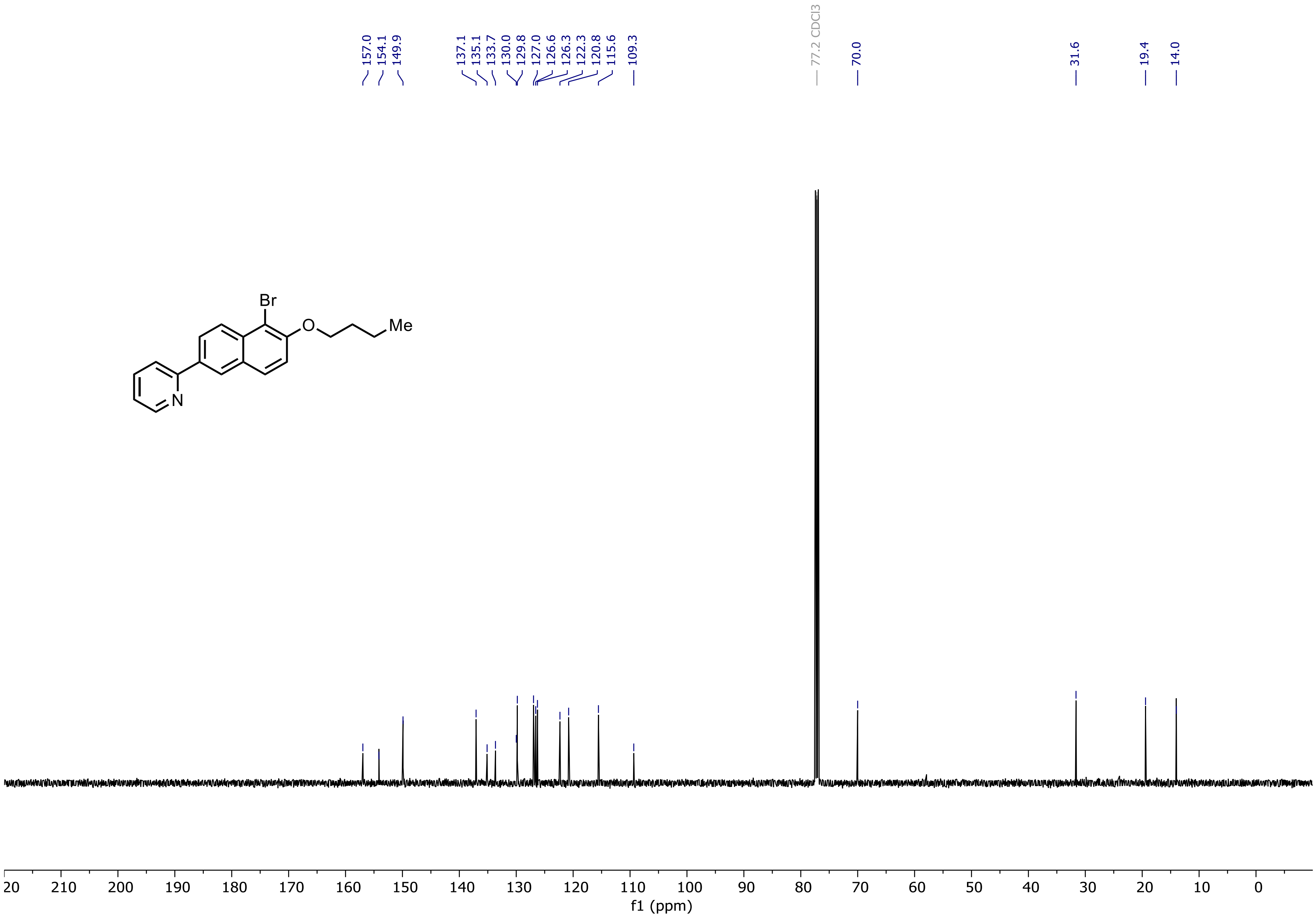

^{1}H NMR of 3-(5-bromo-6-butoxynaphthalen-2-yl)pyridine (6b)

$CDCl_3$, 300 MHz, 298 K

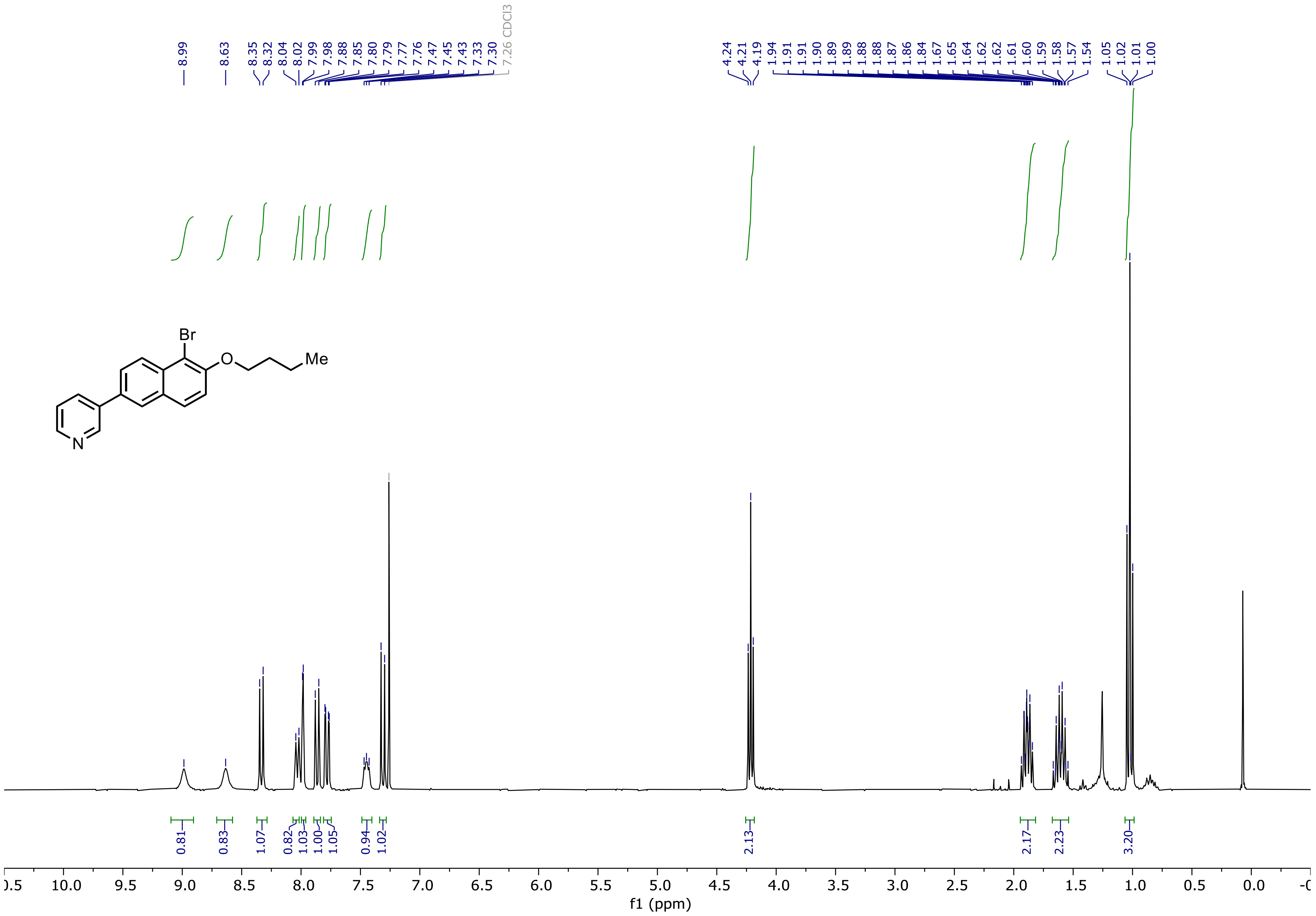

^{13}C NMR of 3-(5-bromo-6-butoxynaphthalen-2-yl)pyridine (6b)

$CDCl_3$, 151 MHz, 298 K

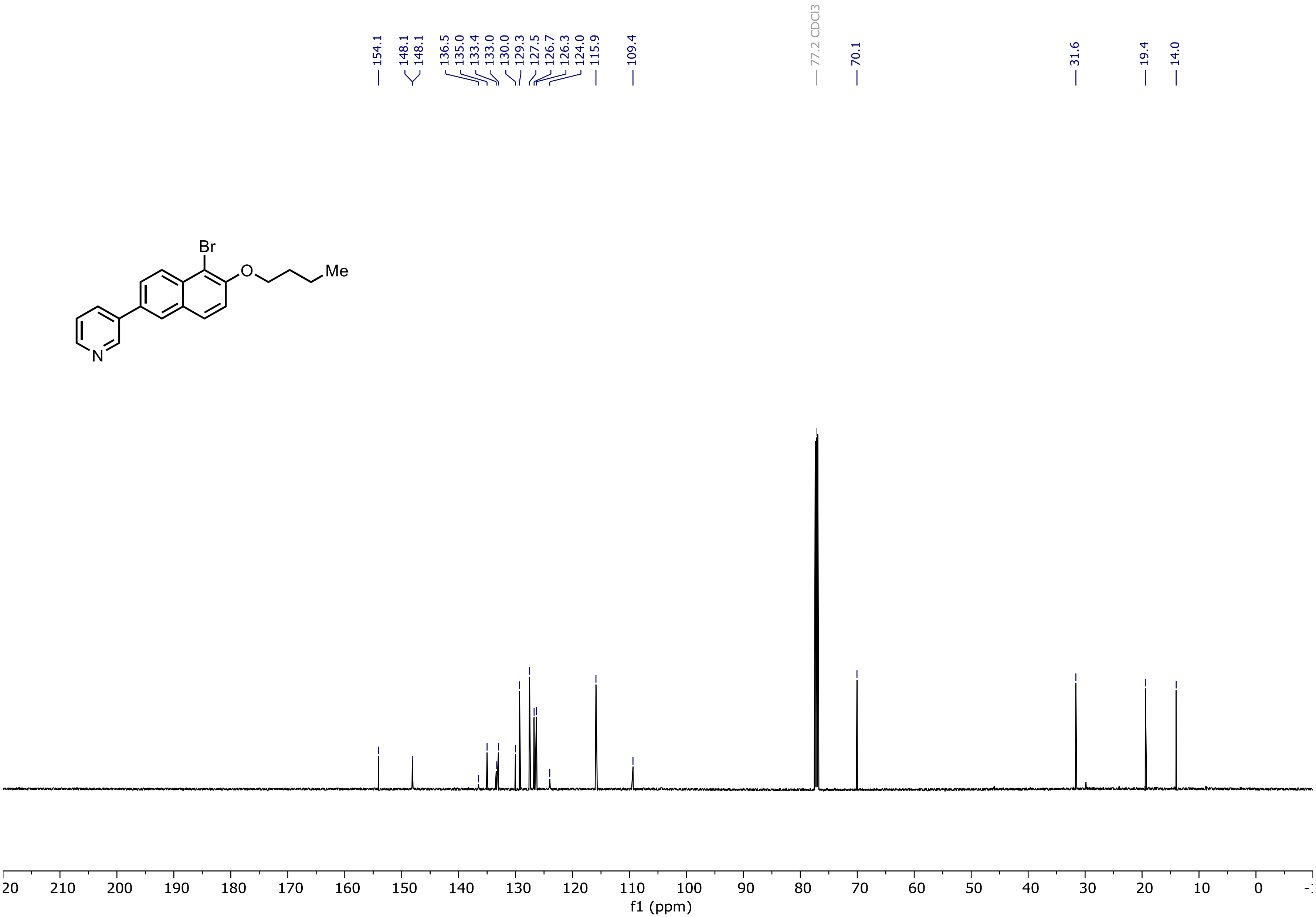

^{1}H NMR of 4-(5-bromo-6-butoxynaphthalen-2-yl)pyridine (6c)

$CDCl_3$, 600 MHz, 298 K

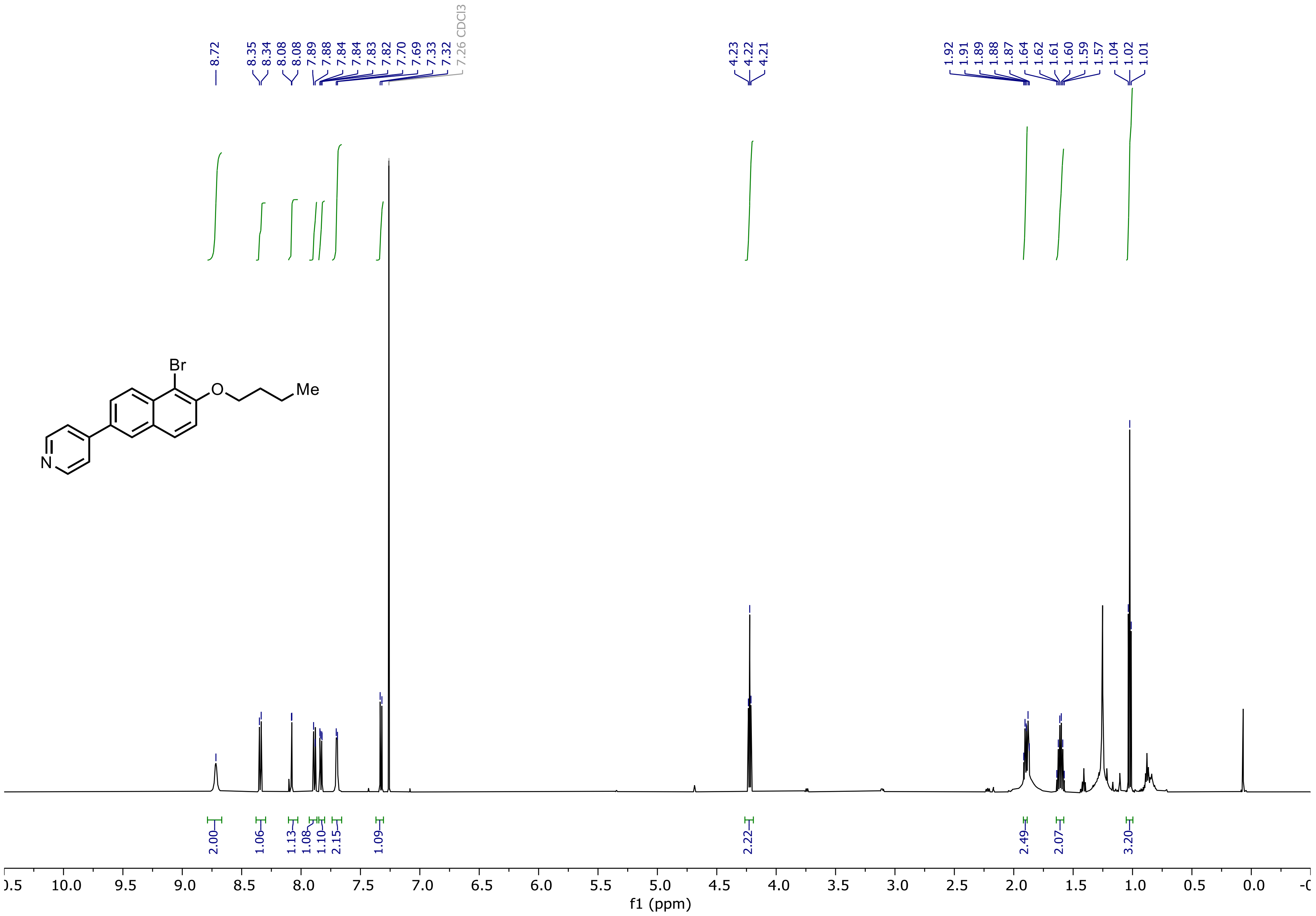

^{13}C NMR of 4-(5-bromo-6-butoxynaphthalen-2-yl)pyridine (6c)

$CDCl_3$, 151 MHz, 298 K

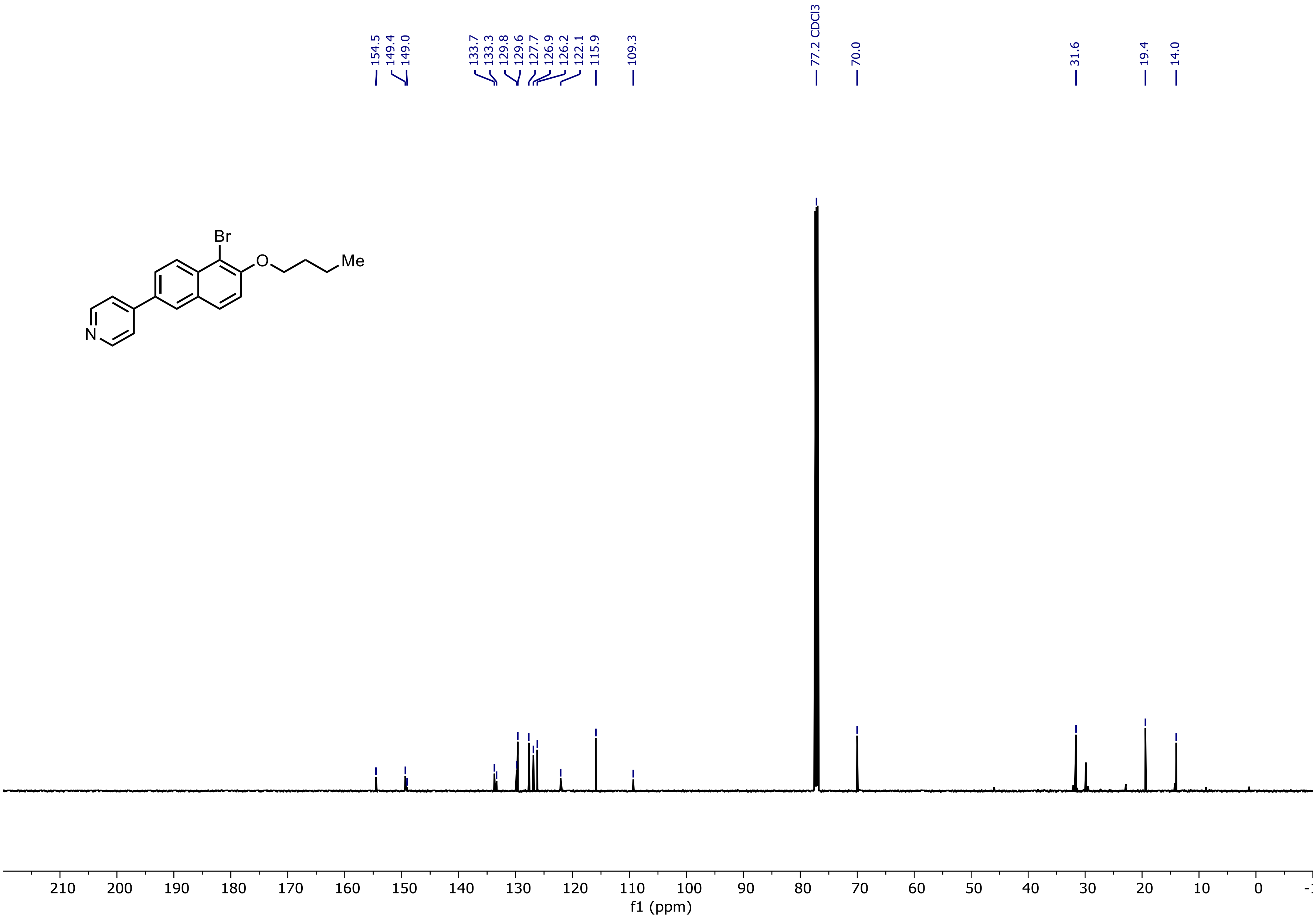

^{1}H NMR of 1-(4-methoxy-3-(pyrazin-2-yl)phenyl)ethan-1-one (7)

$CDCl_3$, 500 MHz, 298 K

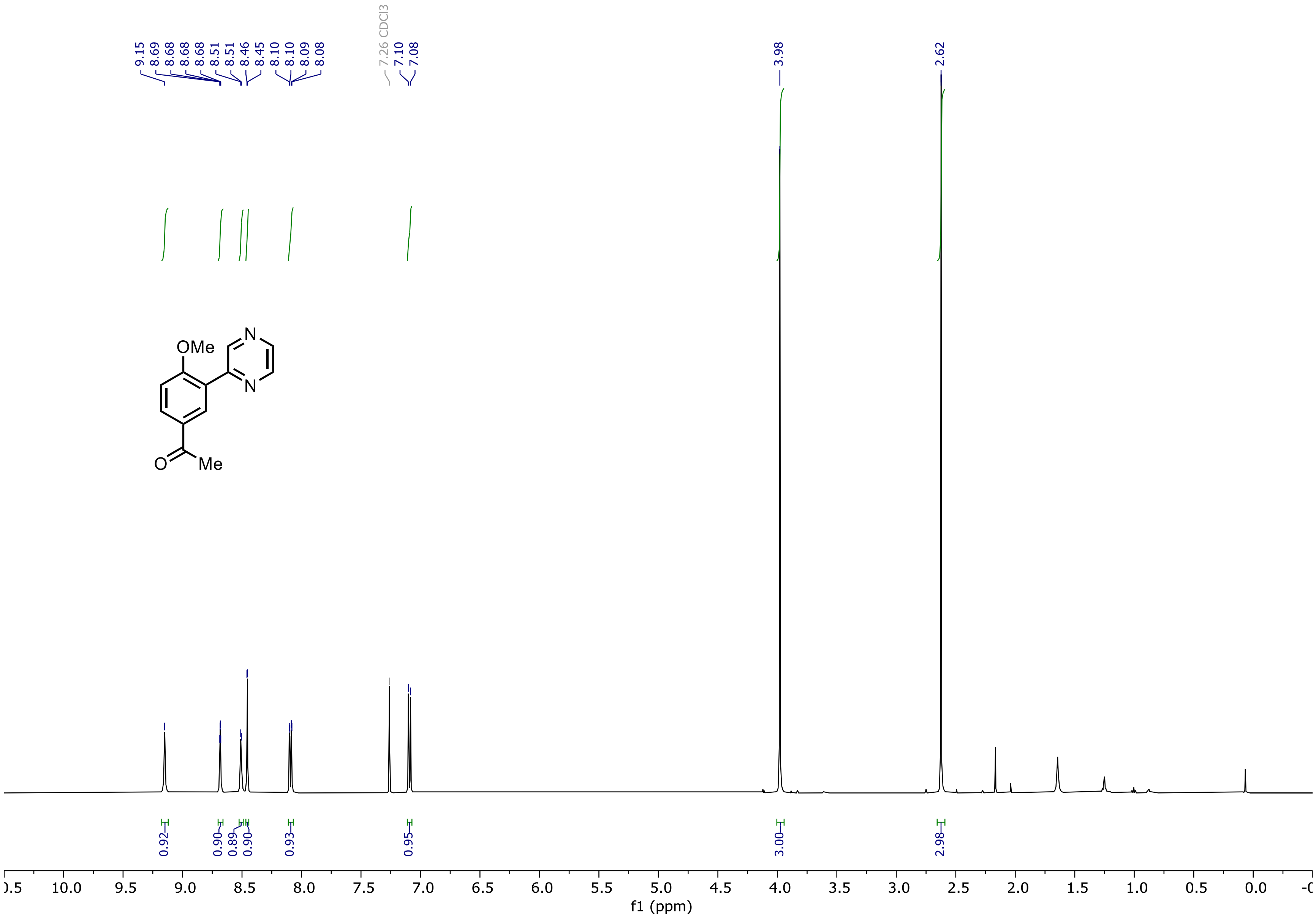

^{13}C NMR of 1-(4-methoxy-3-(pyrazin-2-yl)phenyl)ethan-1-one (7)

$CDCl_3$, 75 MHz, 298

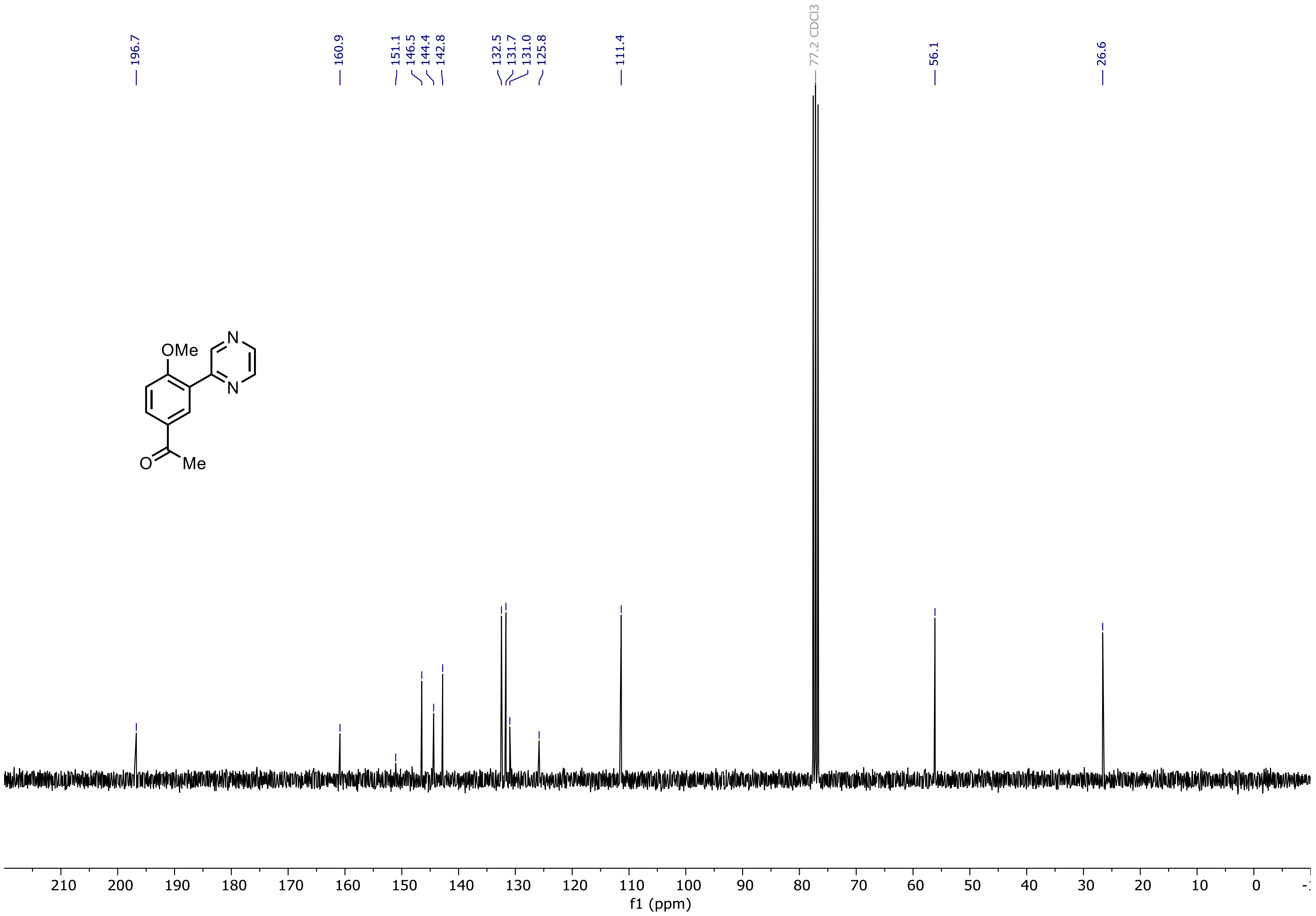

^{1}H NMR of pyriproxyfen pyrazine derivative (8)

$CDCl_3$, 500 MHz, 298 K

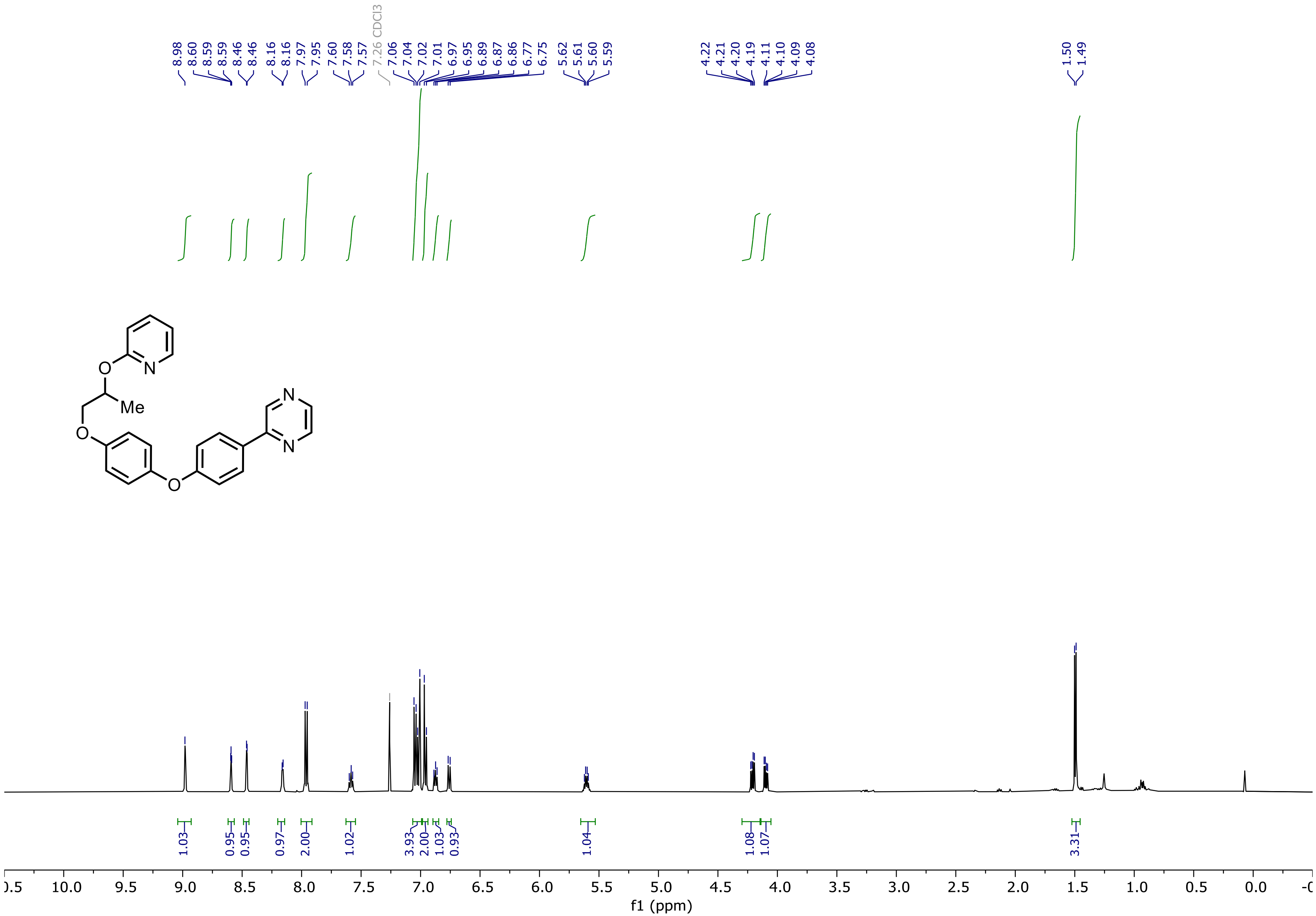

^{13}C NMR of pyriproxyfen pyrazine derivative (8)

$CDCl_3$, 126 MHz, 298 K

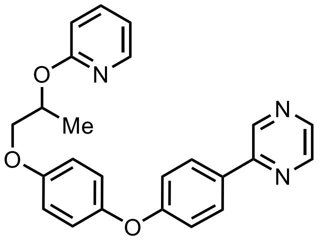

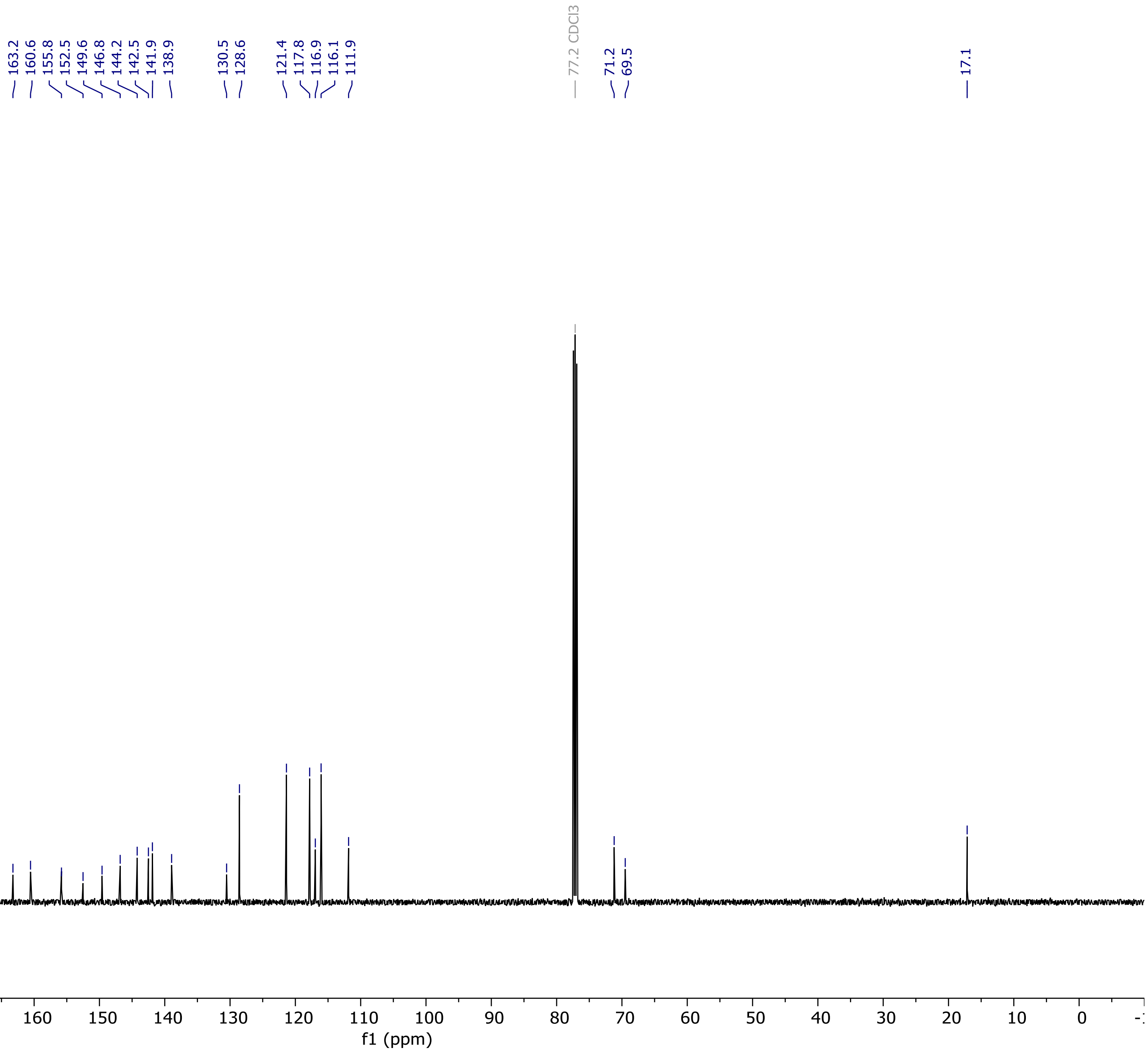

^{1}H NMR of boscalid pyrimidine derivative (9a)

$CDCl_3$, 500 MHz, 298 K

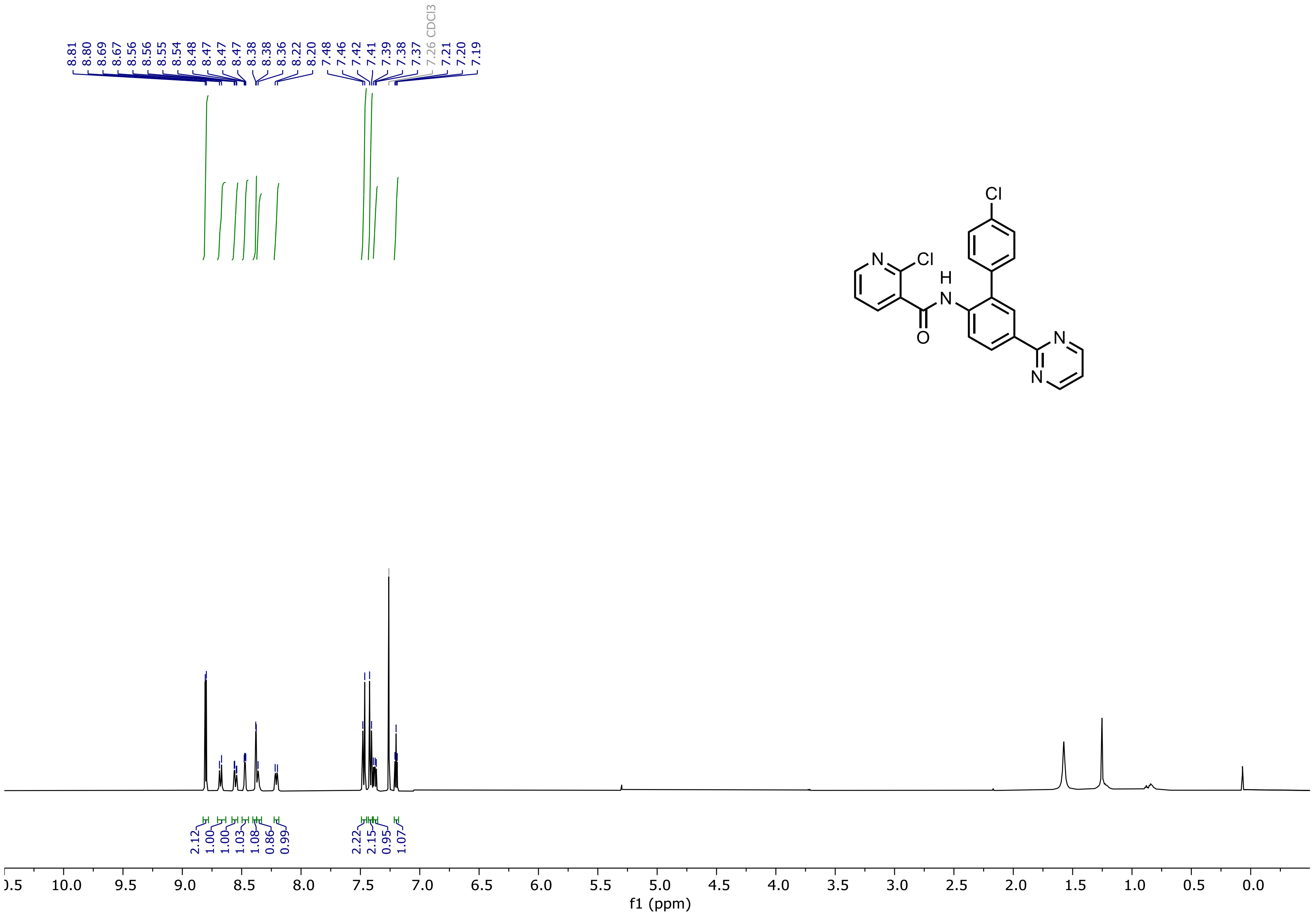

^{13}C NMR of boscalid pyrimidine derivative (9a)

$CDCl_3$, 151 MHz, 298 K

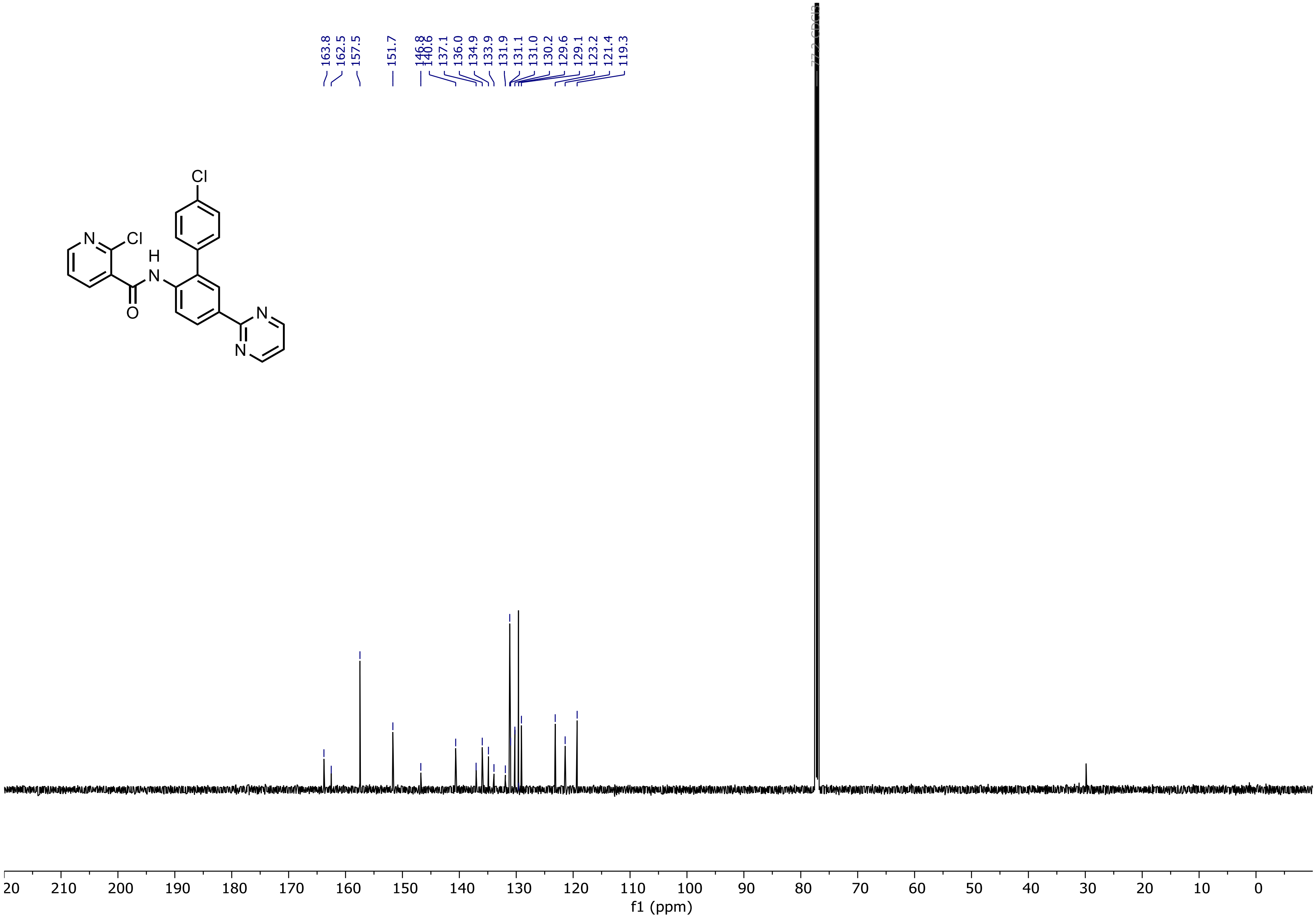

^{1}H NMR of boscalid pyrimidine derivative (9b)

$CDCl_3$, 500 MHz, 298 K

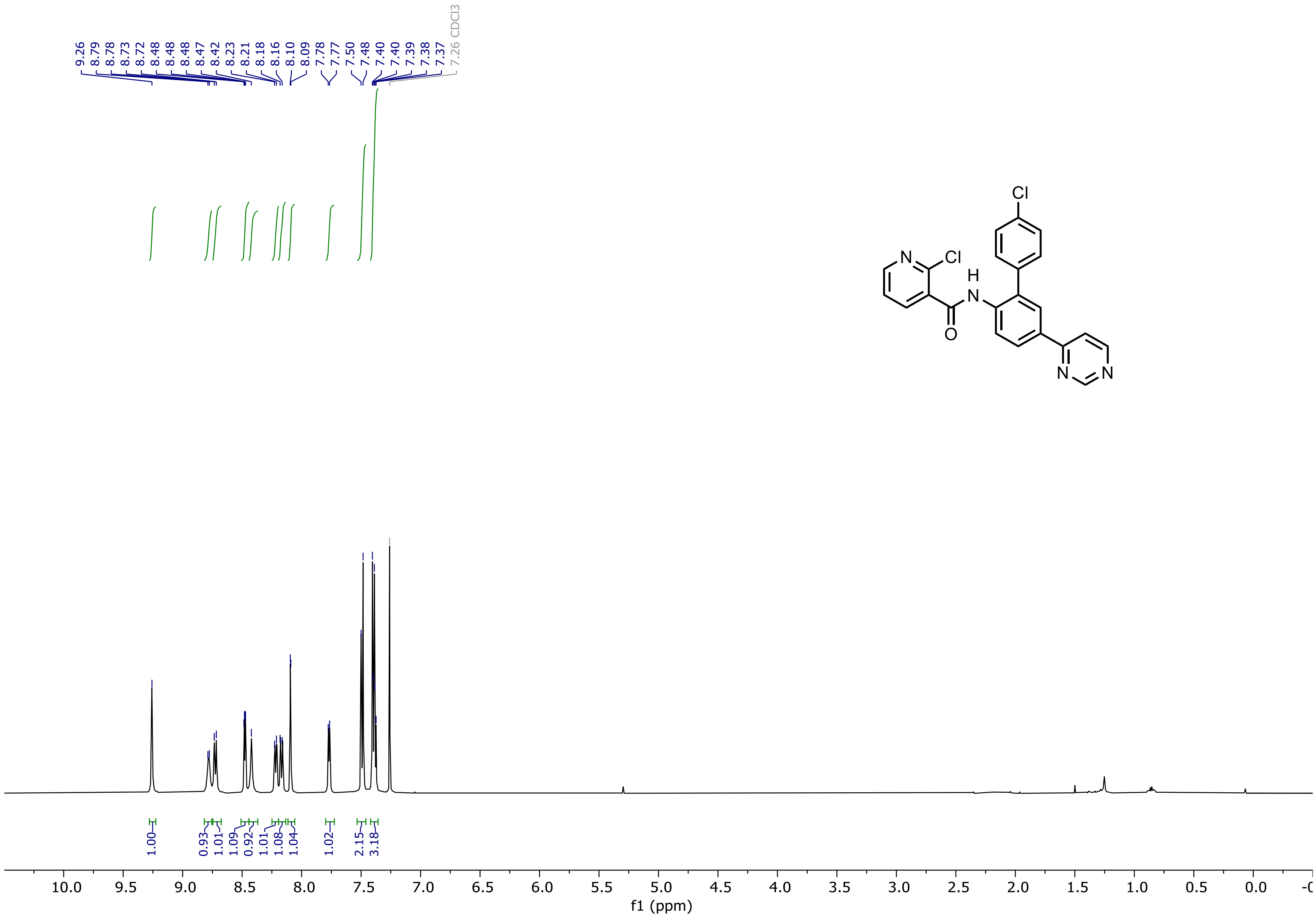

^{13}C NMR of of boscalid pyrimidine derivative (9b)

$CDCl_3$, 151 MHz, 298 K

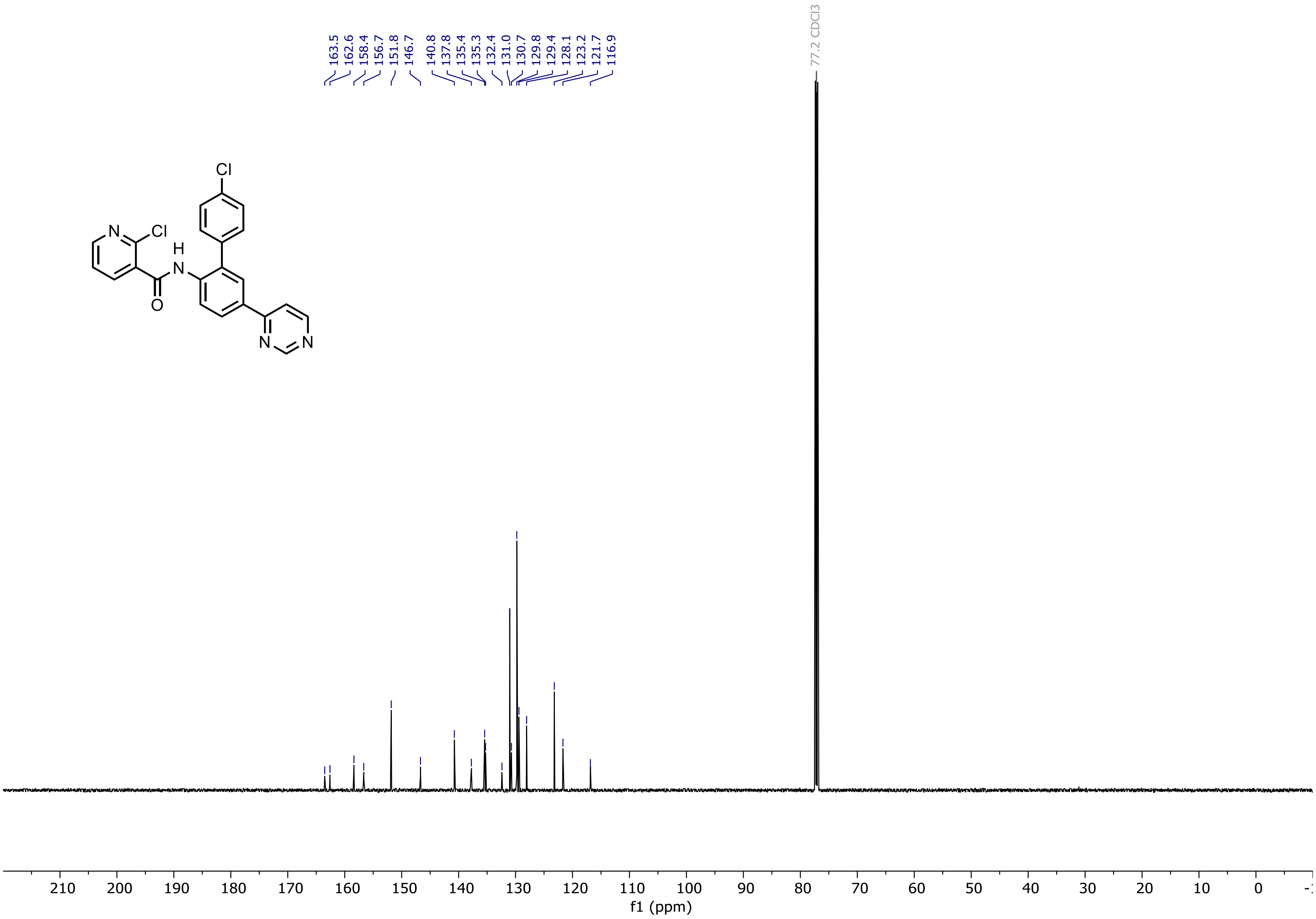

^{1}H NMR of bifonazole pyrazine derivative (10)

$CDCl_3$, 500 MHz, 298 K

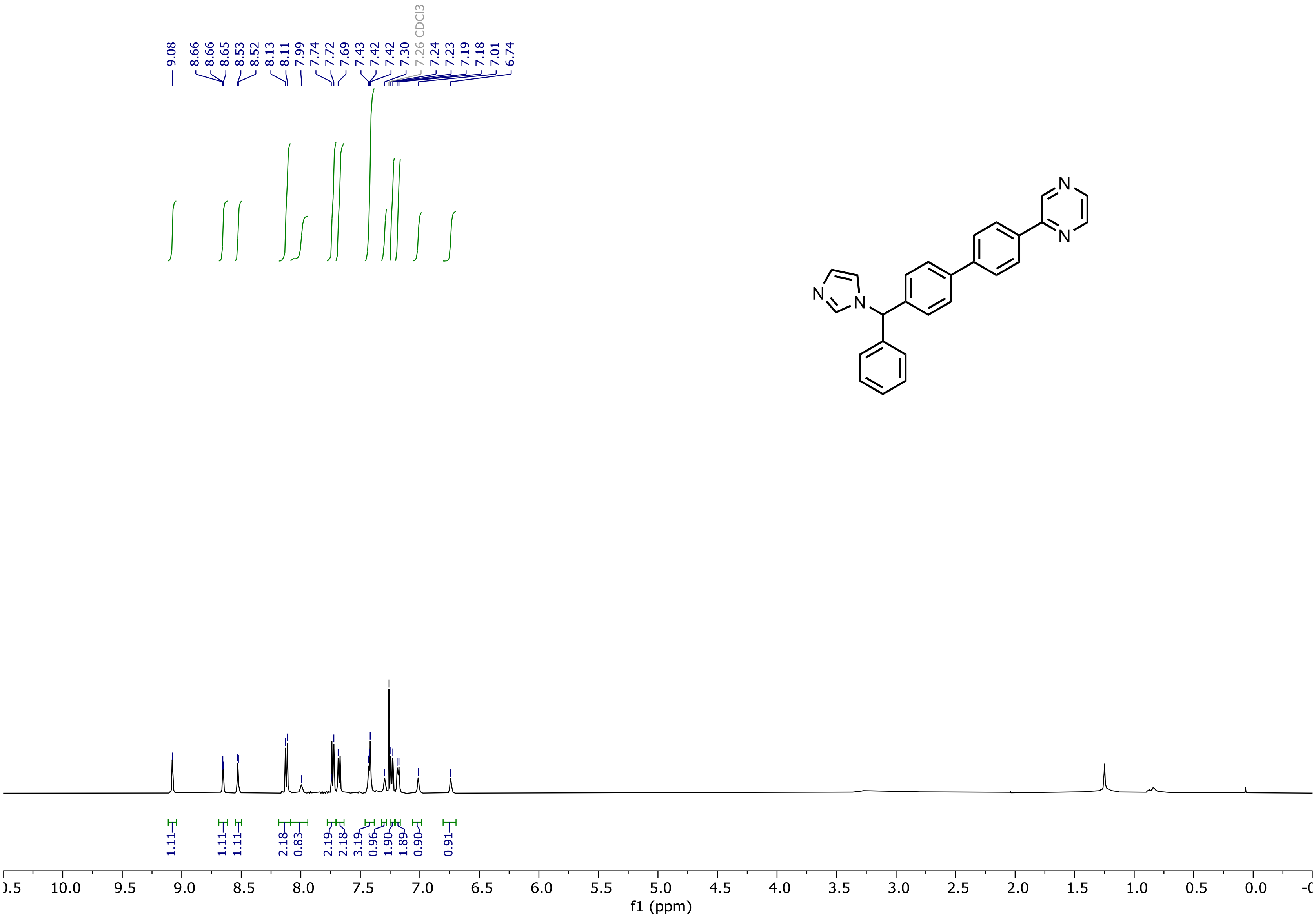

^{13}C NMR of bifonazole pyrazine derivative (10)

$CDCl_3$, 126 MHz, 298 K

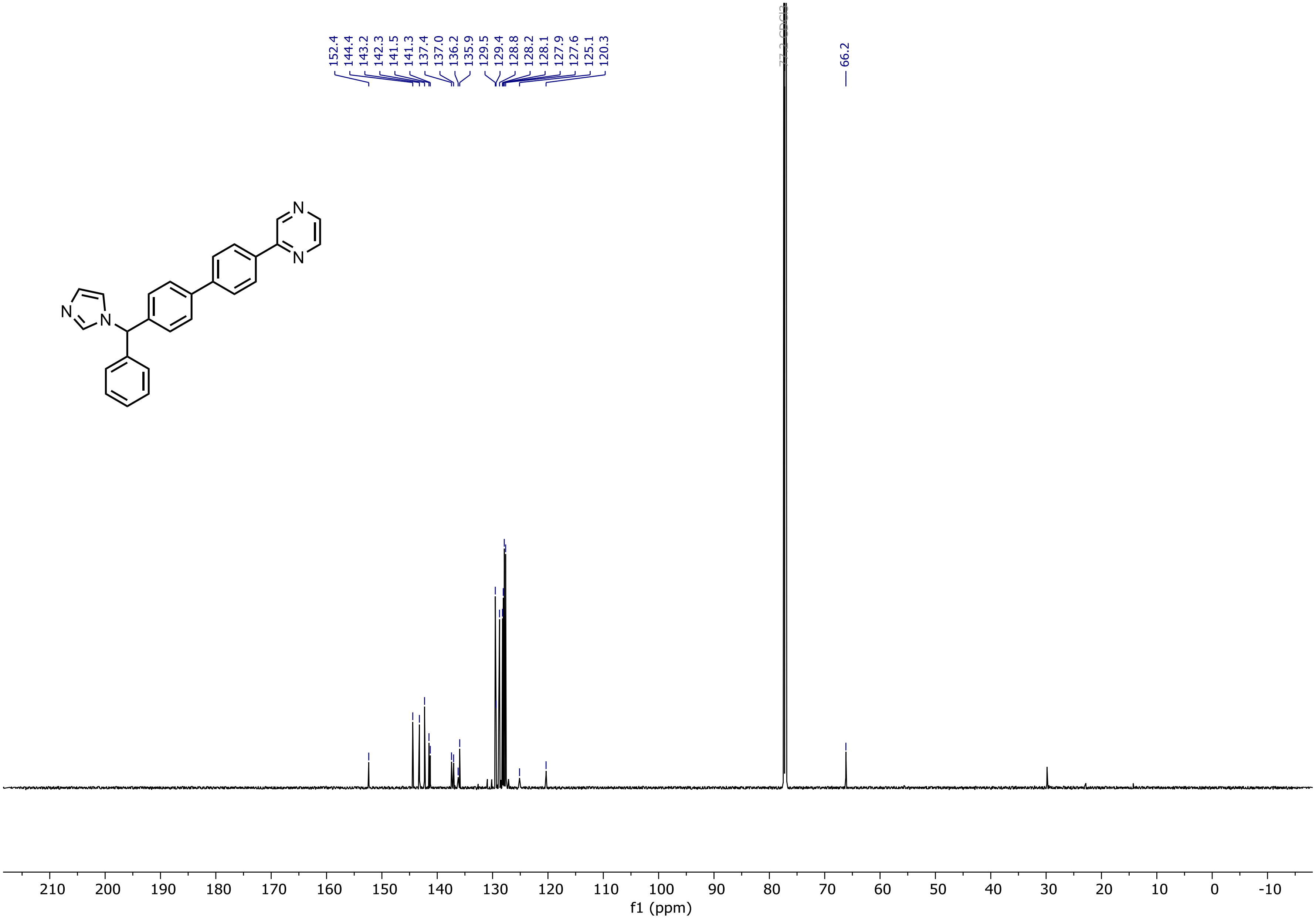

^{1}H NMR of 1-(4-methoxy-3-(1-methyl-1H-pyrrol-2-yl)phenyl)ethan-1-one (11)

$CDCl_3$, 500 MHz, 298 K

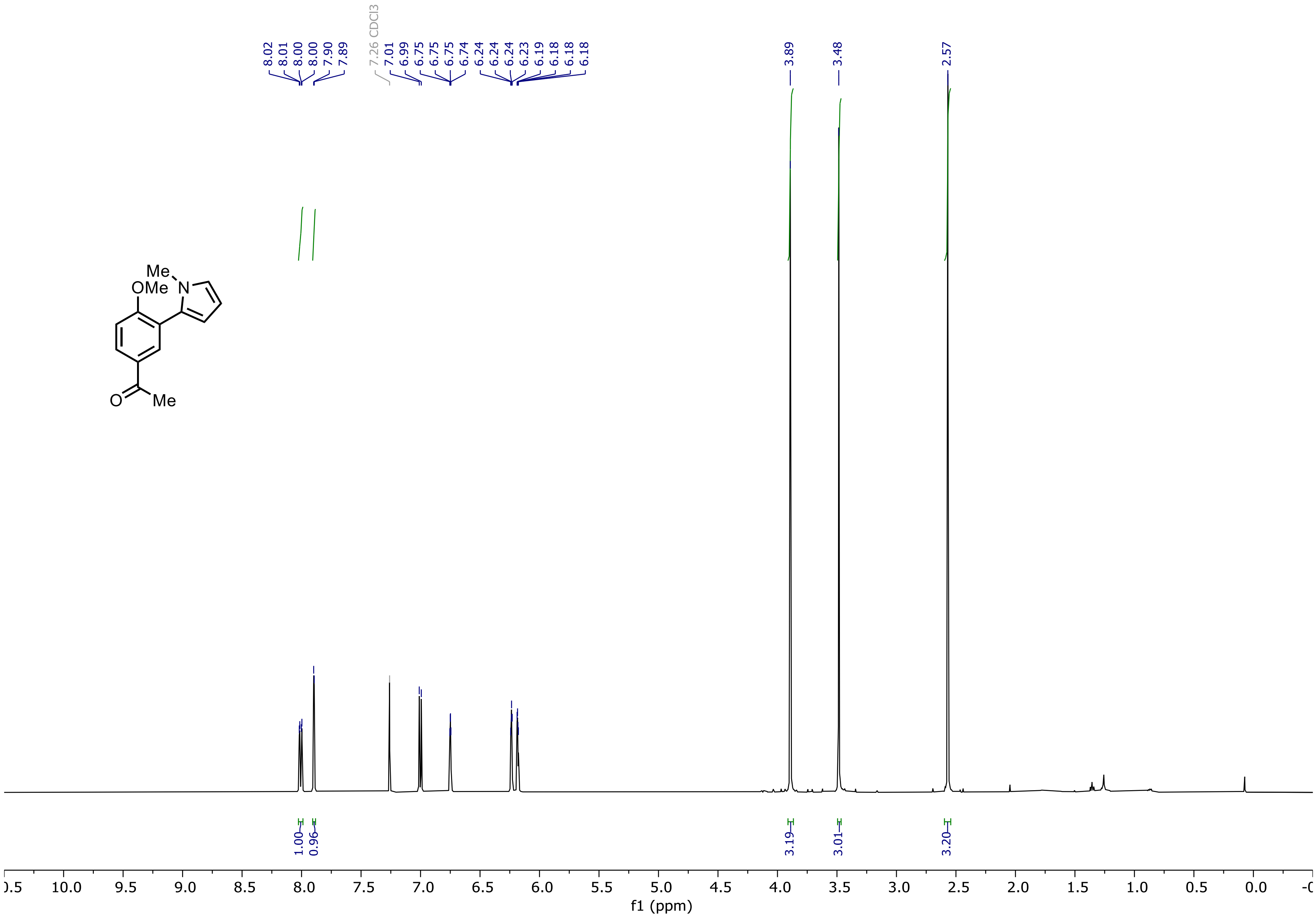

^{13}C NMR of 1-(4-methoxy-3-(1-methyl-1H-pyrrol-2-yl)phenyl)ethan-1-one (11)

$CDCl_3$, 75 MHz, 298 K

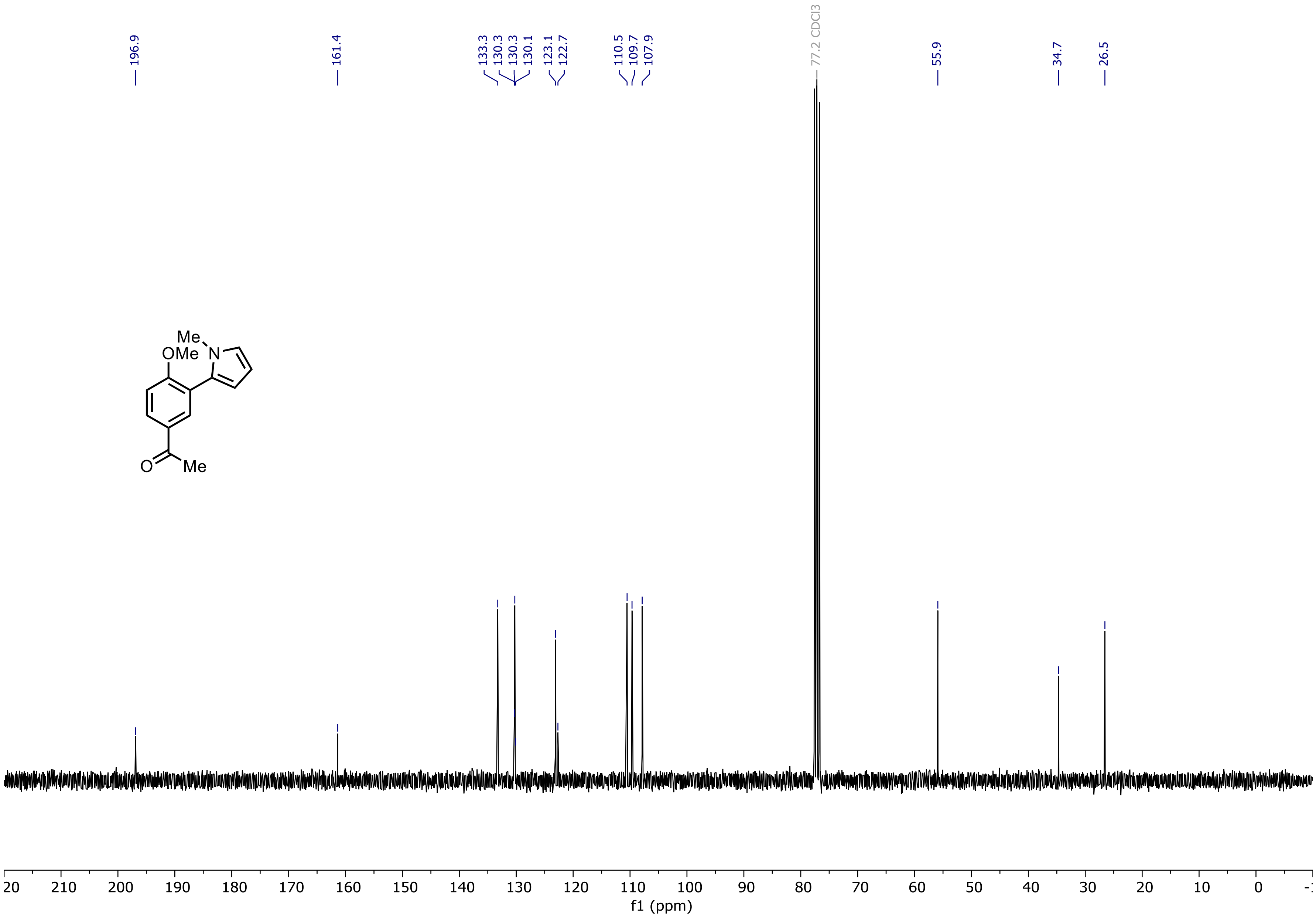

^{1}H NMR of 5-(4-(5-acetyl-1-methyl-1H-pyrrol-2-yl)phenoxy)-2-fluorobenzonitrile (12)

$CDCl_3$, 500 MHz, 298 K

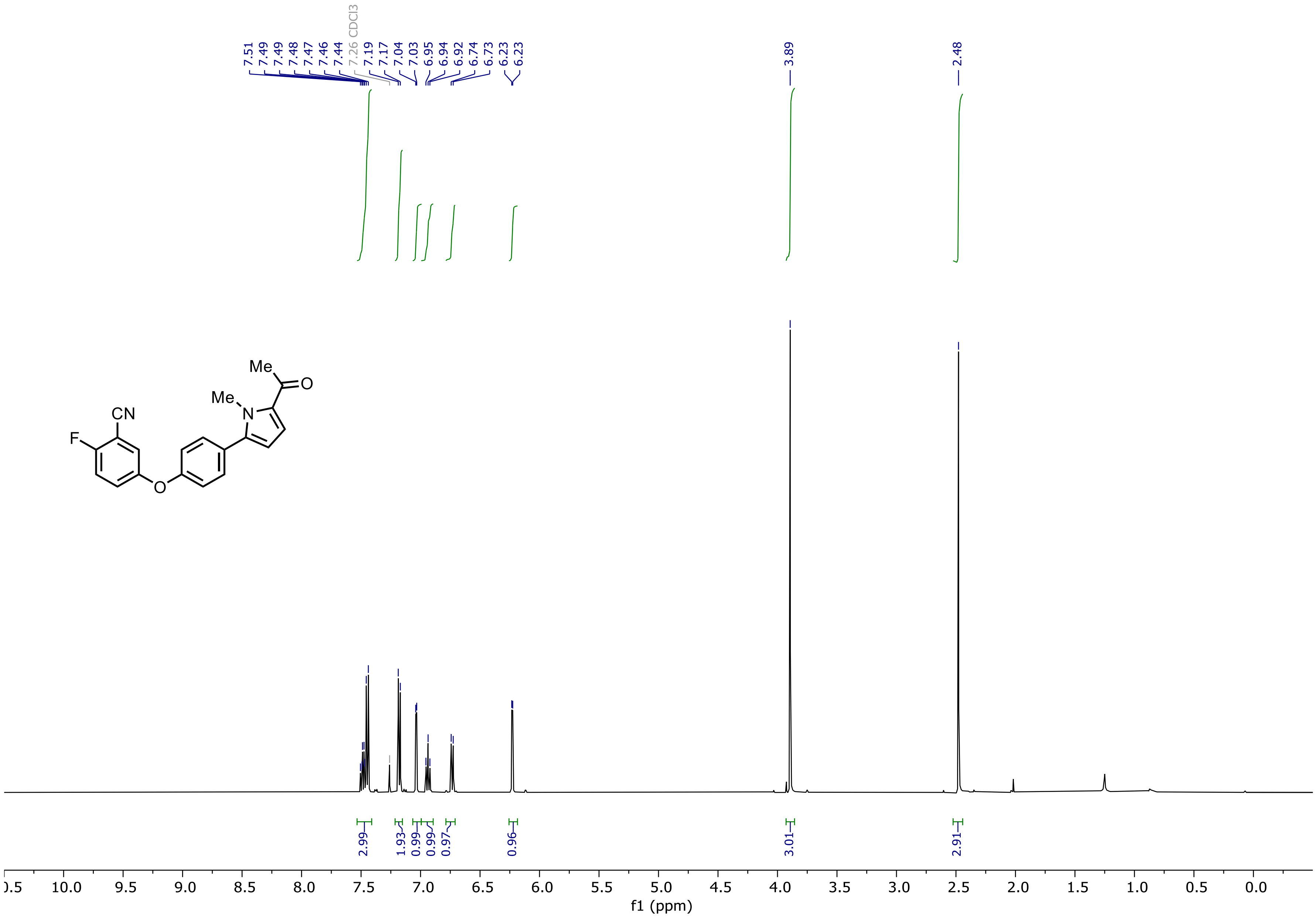

^{13}C NMR of 5-(4-(5-acetyl-1-methyl-1H-pyrrol-2-yl)phenoxy)-2-fluorobenzonitrile (12)

$CDCl_3$, 75 MHz, 298 K

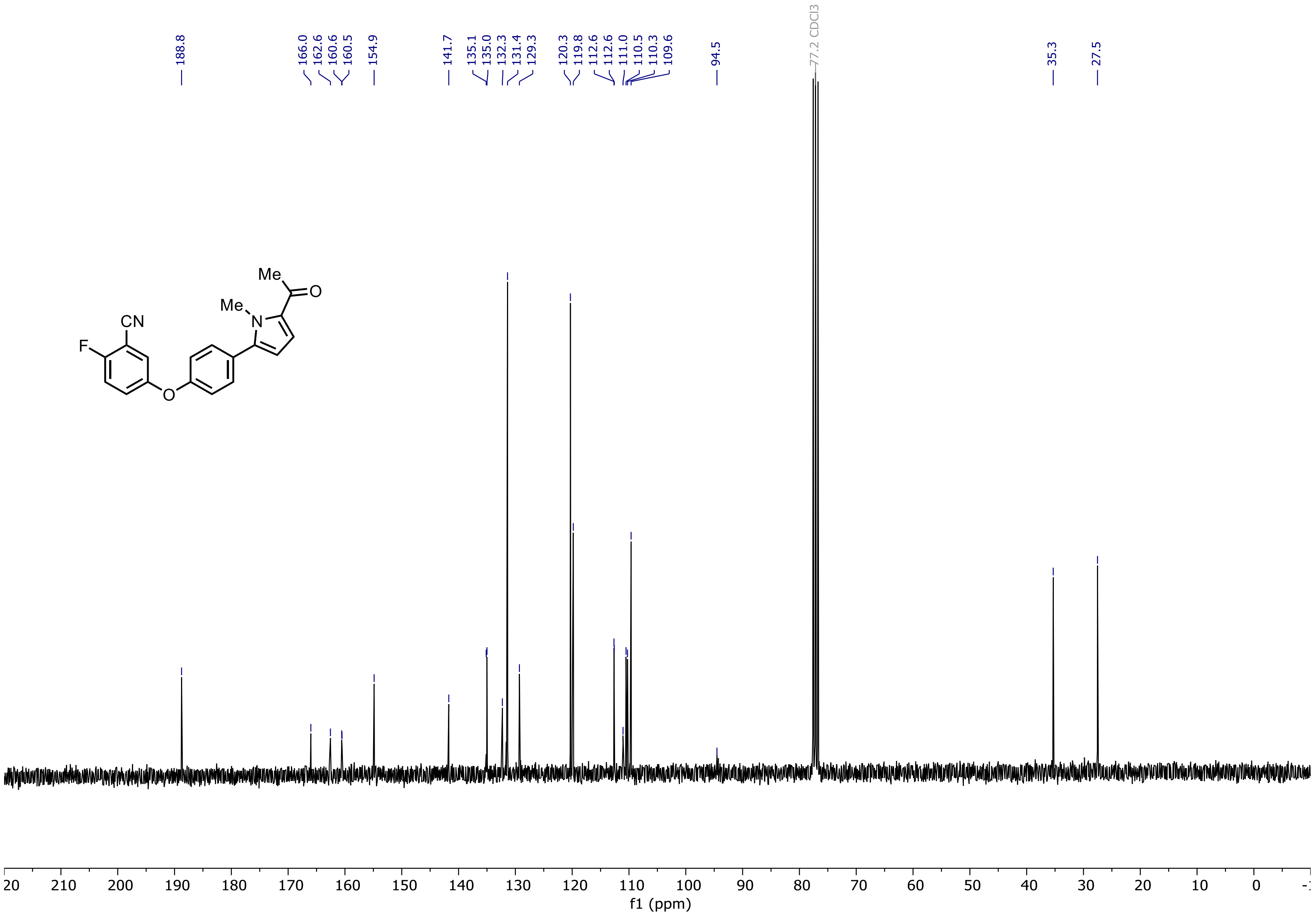

^{19}F NMR of 5-(4-(5-acetyl-1-methyl-1H-pyrrol-2-yl)phenoxy)-2-fluorobenzonitrile (12)

CD_3CN, 471 MHz, 298 K

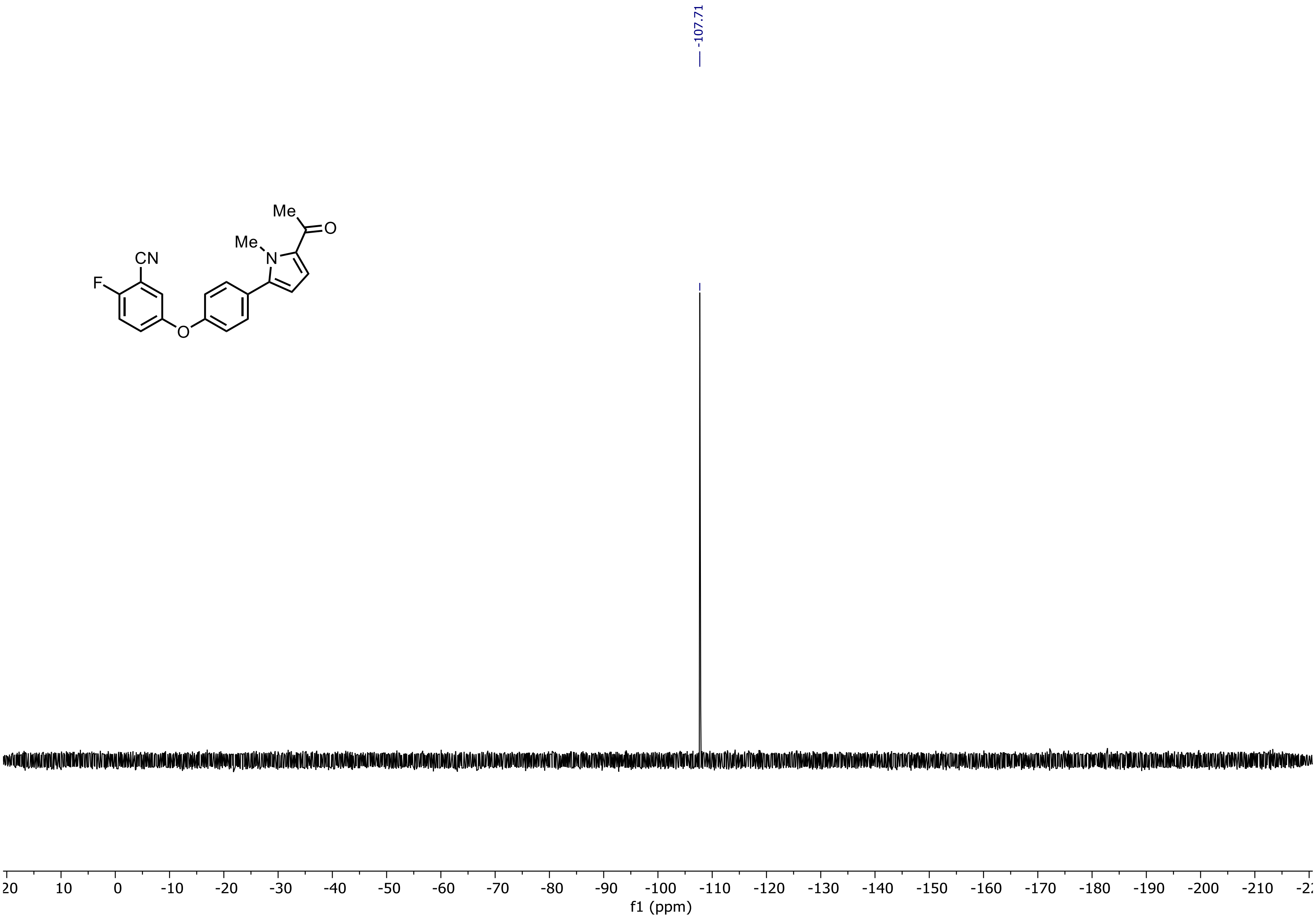

^{1}H NMR of 5-([1,1'-biphenyl]-4-yl)-1-methyl-1H-imidazole (13a)

$CDCl_3$, 500 MHz, 298 K

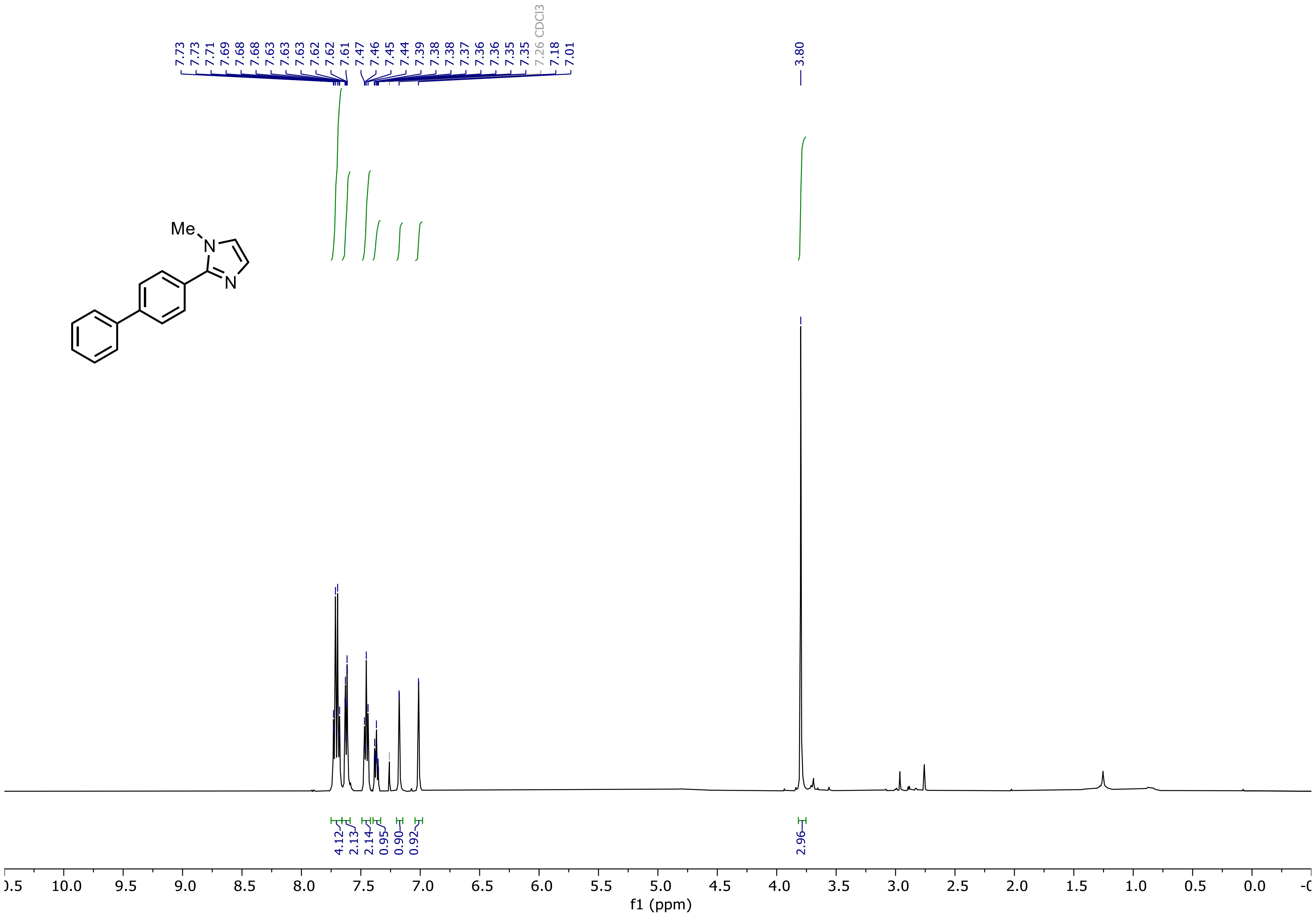

^{13}C NMR of 5-([1,1'-biphenyl]-4-yl)-1-methyl-1H-imidazole (13a)

$CDCl_3$, 126 MHz, 298 K

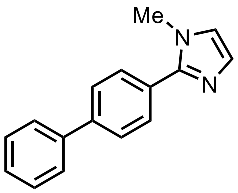

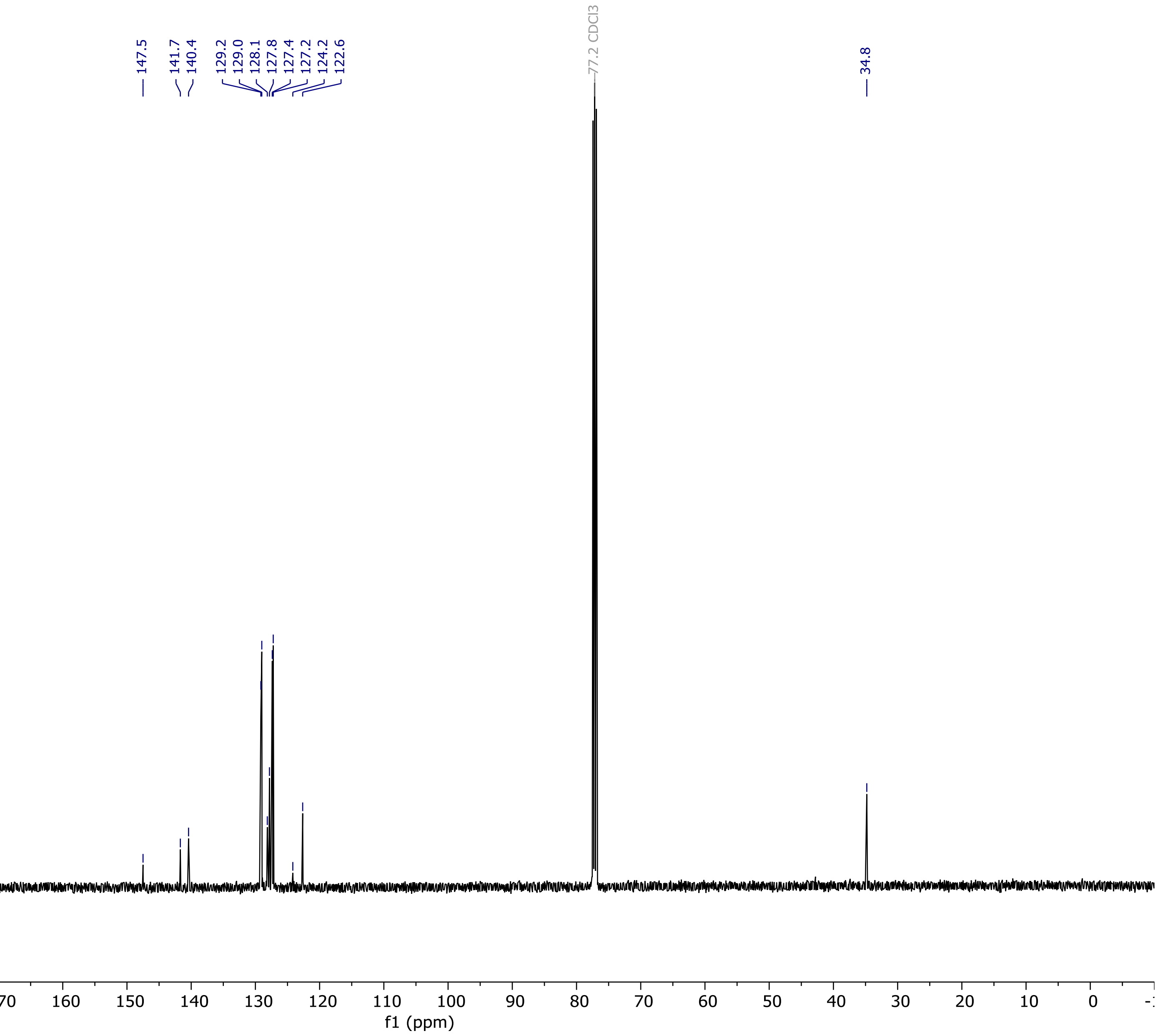

^{1}H NMR of 5-([1,1'-biphenyl]-4-yl)-1-methyl-1H-imidazole (13b)

$CDCl_3$, 600 MHz, 298 K

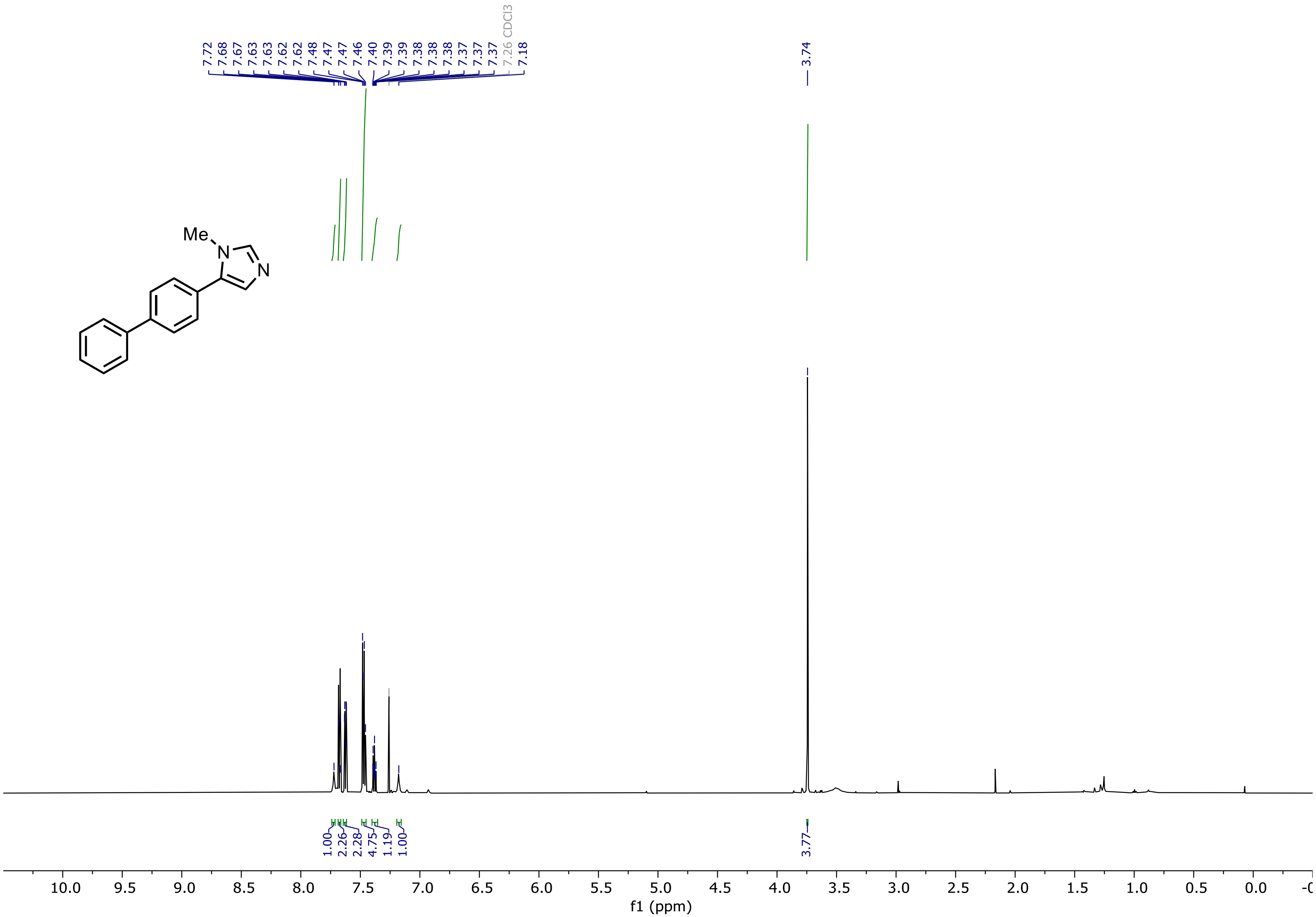

^{13}C NMR of 5-([1,1'-biphenyl]-4-yl)-1-methyl-1H-imidazole (13b)

$CDCl_3$, 151 MHz, 298 K

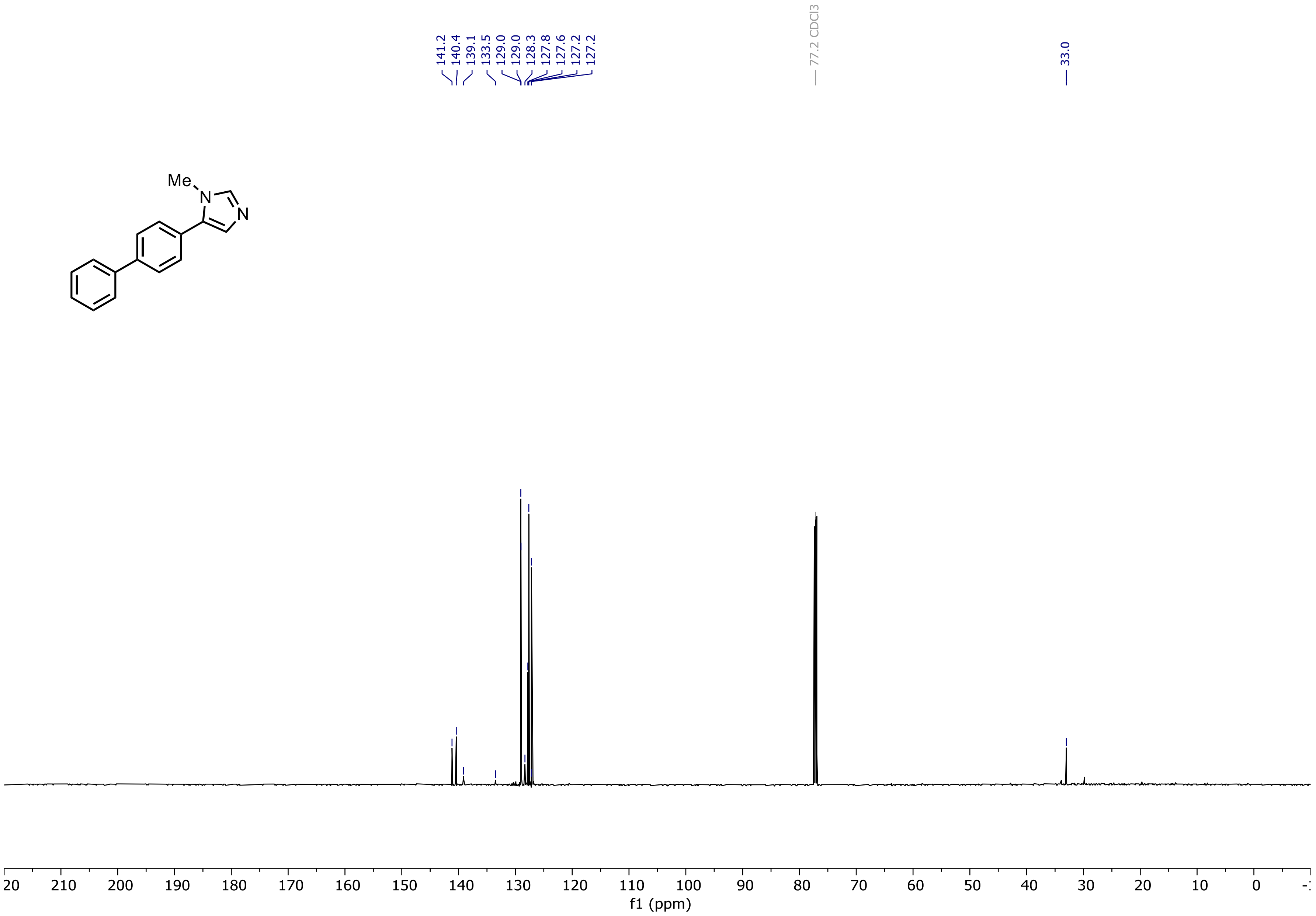

^{1}H NMR of indometacin-*N*-methyl pyrrole derivative (14)

$CDCl_3$, 500 MHz, 298 K

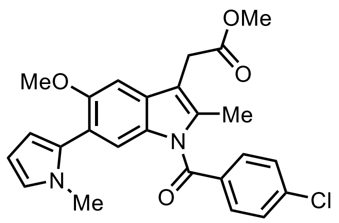

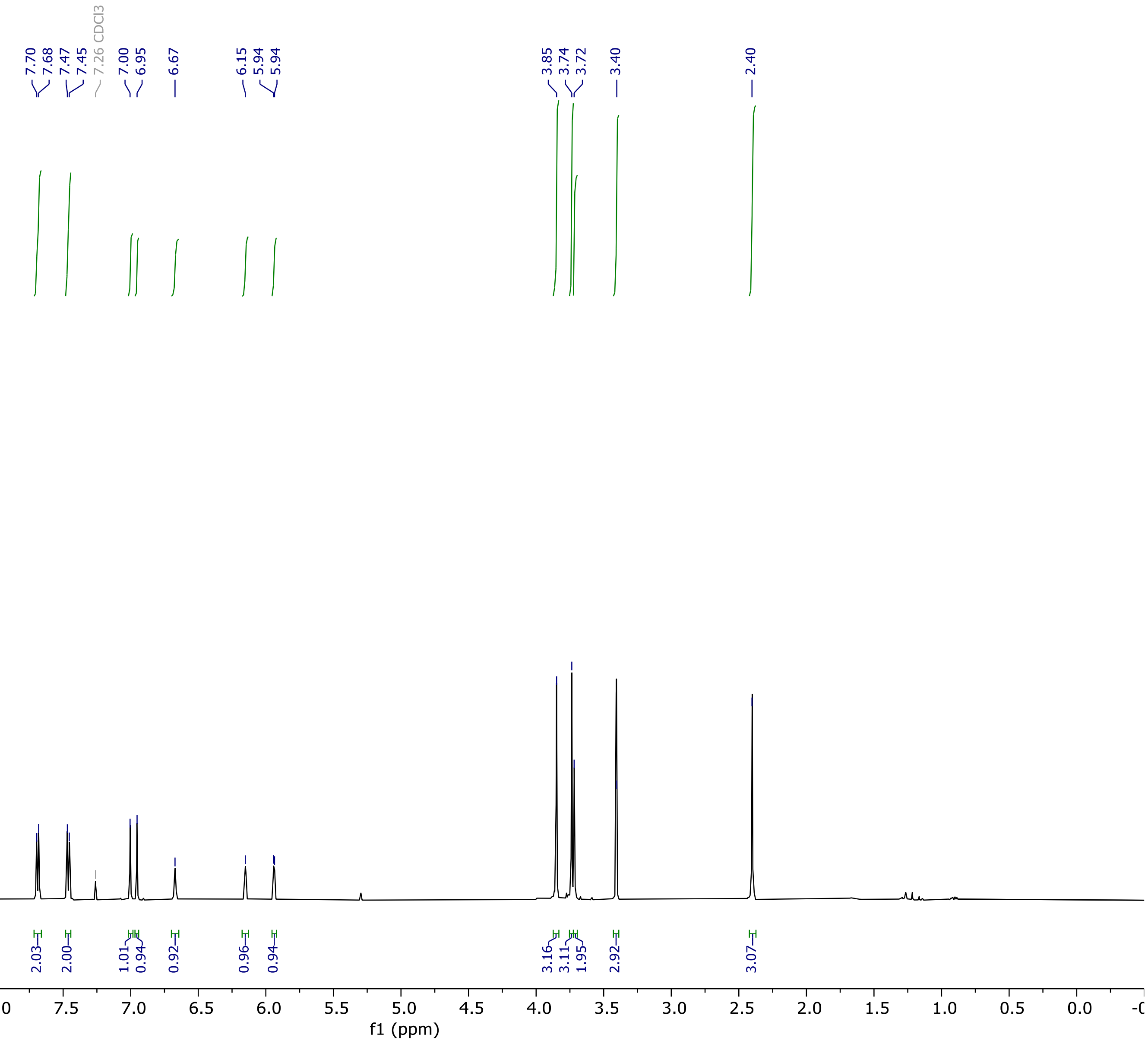

^{13}C NMR of indometacin-N-methyl pyrrole derivative (14)

$CDCl_3$, 126 MHz, 298 K

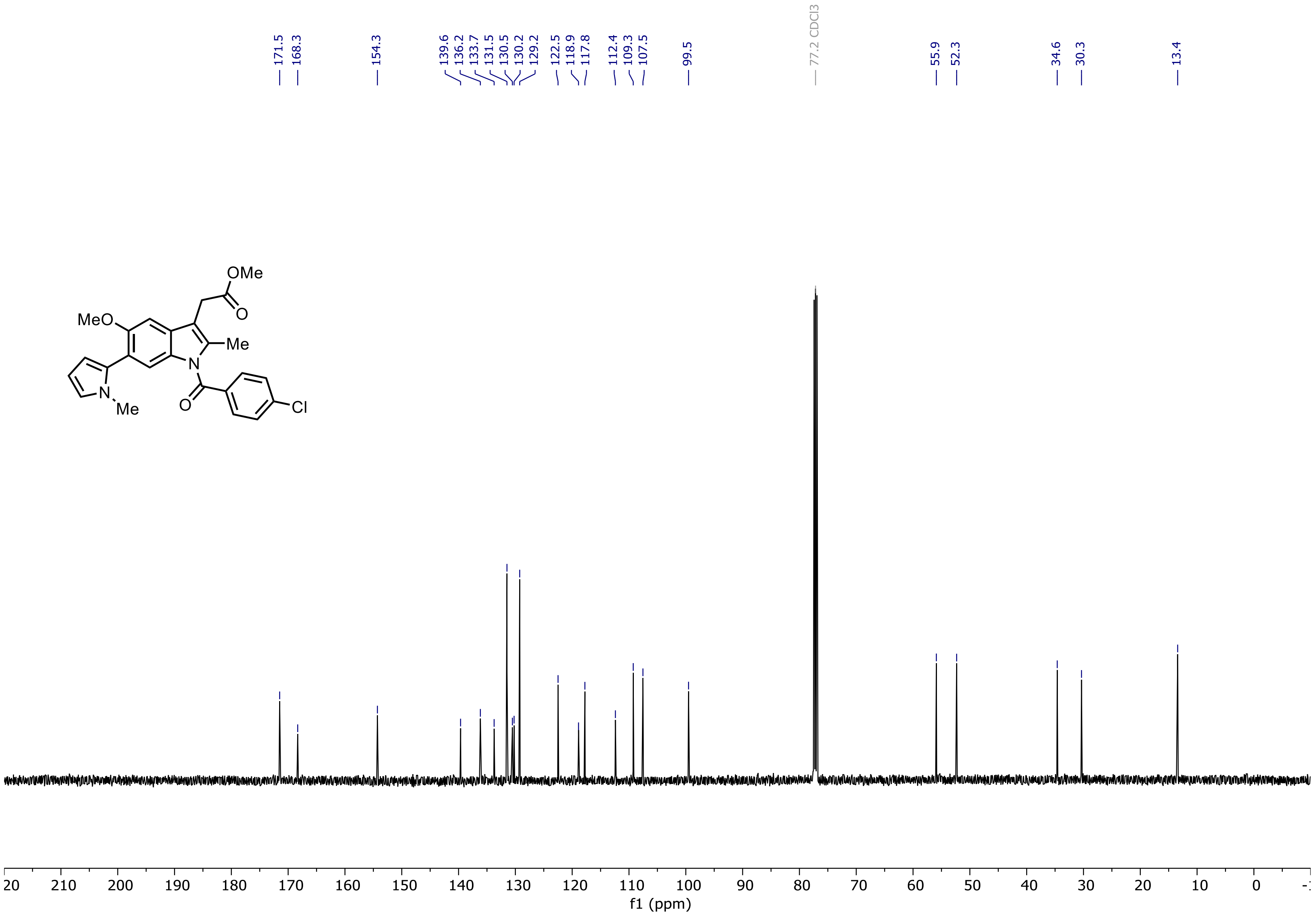

^{1}H NMR of 2-([1,1'-biphenyl]-4-yl)-1-methyl-1H-pyrrole (15)

$CDCl_3$, 500 MHz, 298 K

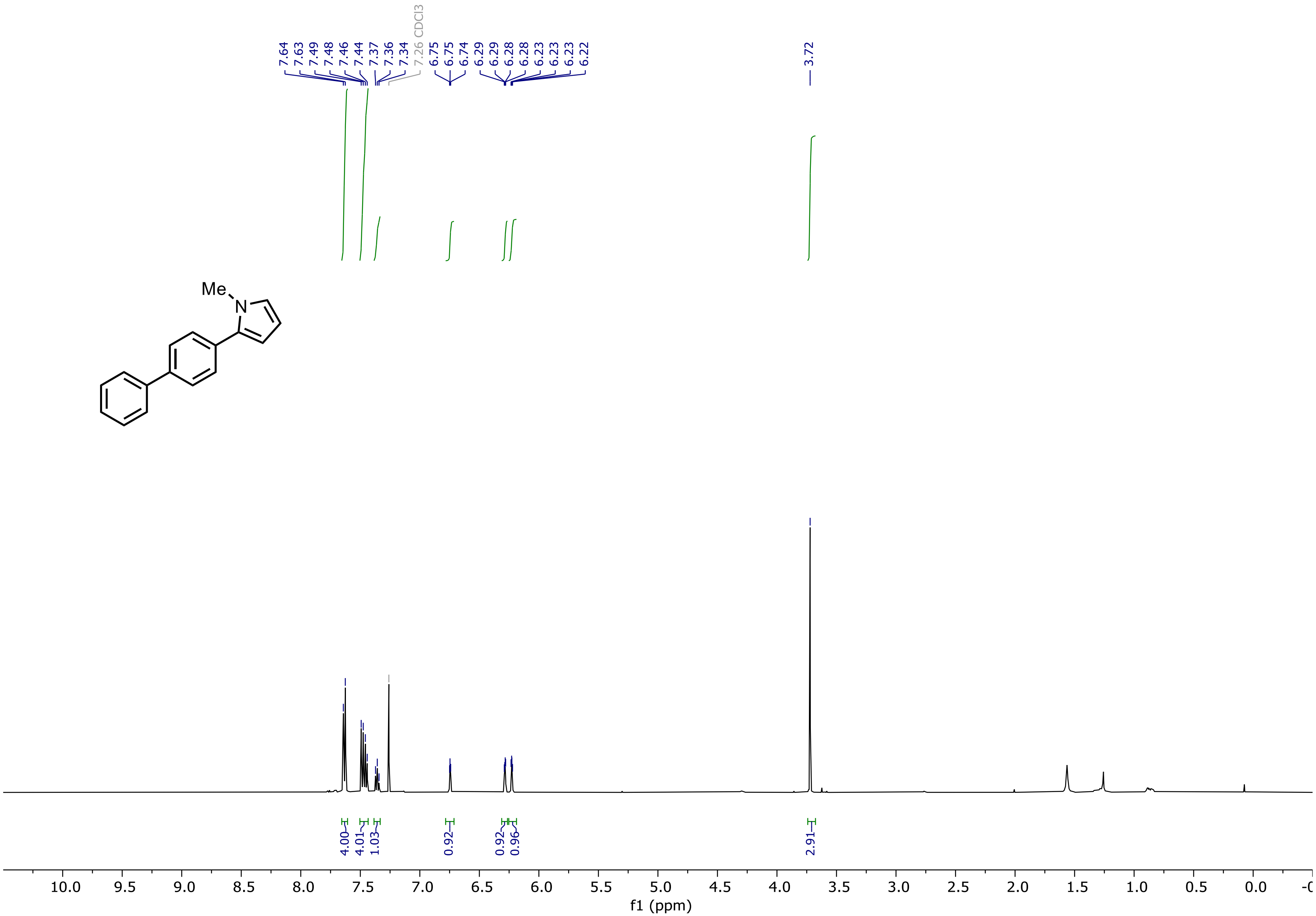

^{13}C NMR of 2-([1,1'-biphenyl]-4-yl)-1-methyl-1H-pyrrole (15)

$CDCl_3$, 126 MHz, 298 K

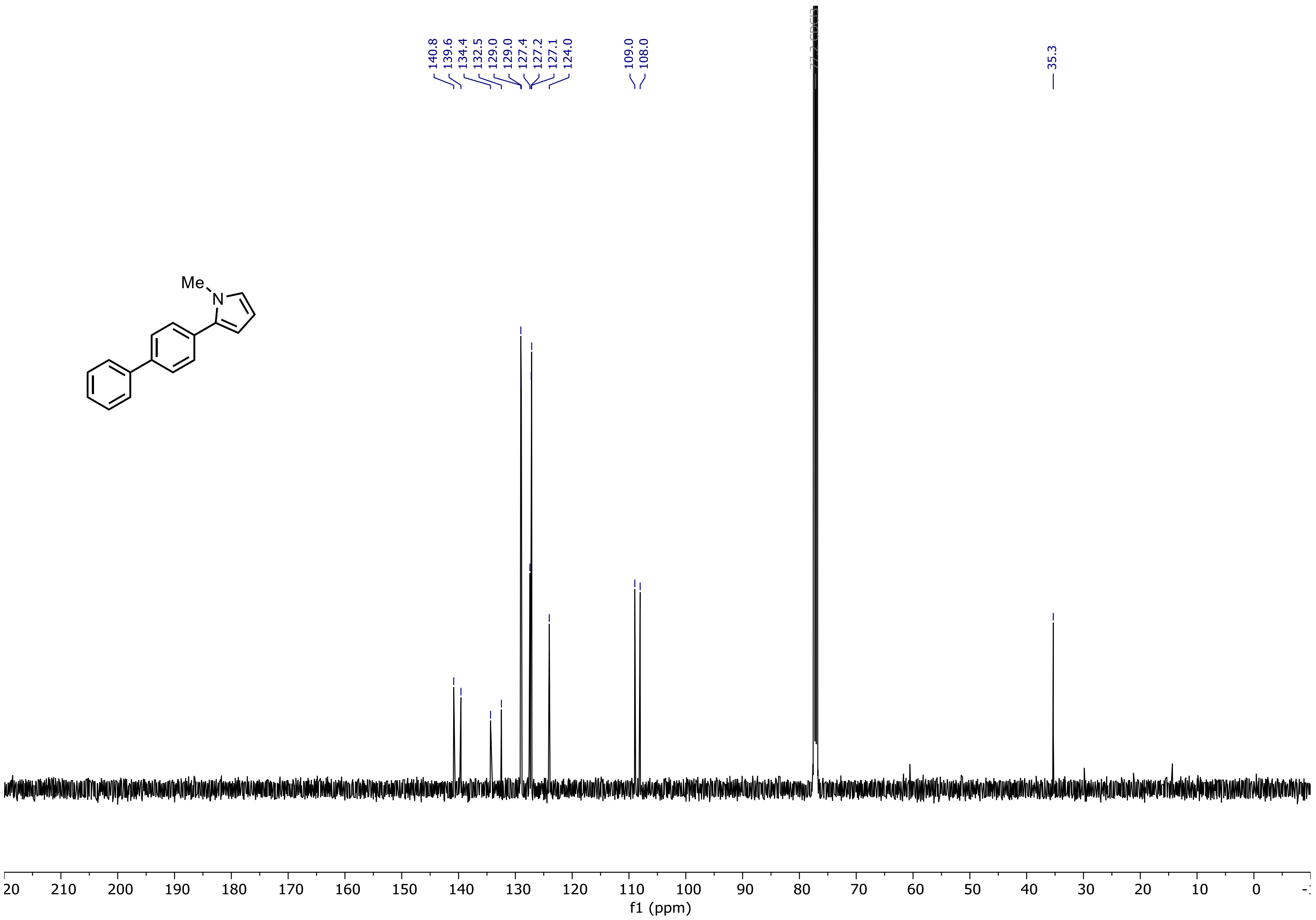

^{1}H NMR 2-(4-methoxy-2-methyl-5-nitrophenyl)-1-methyl-1H-pyrrole (16)

$CDCl_3$, 500 MHz, 298 K

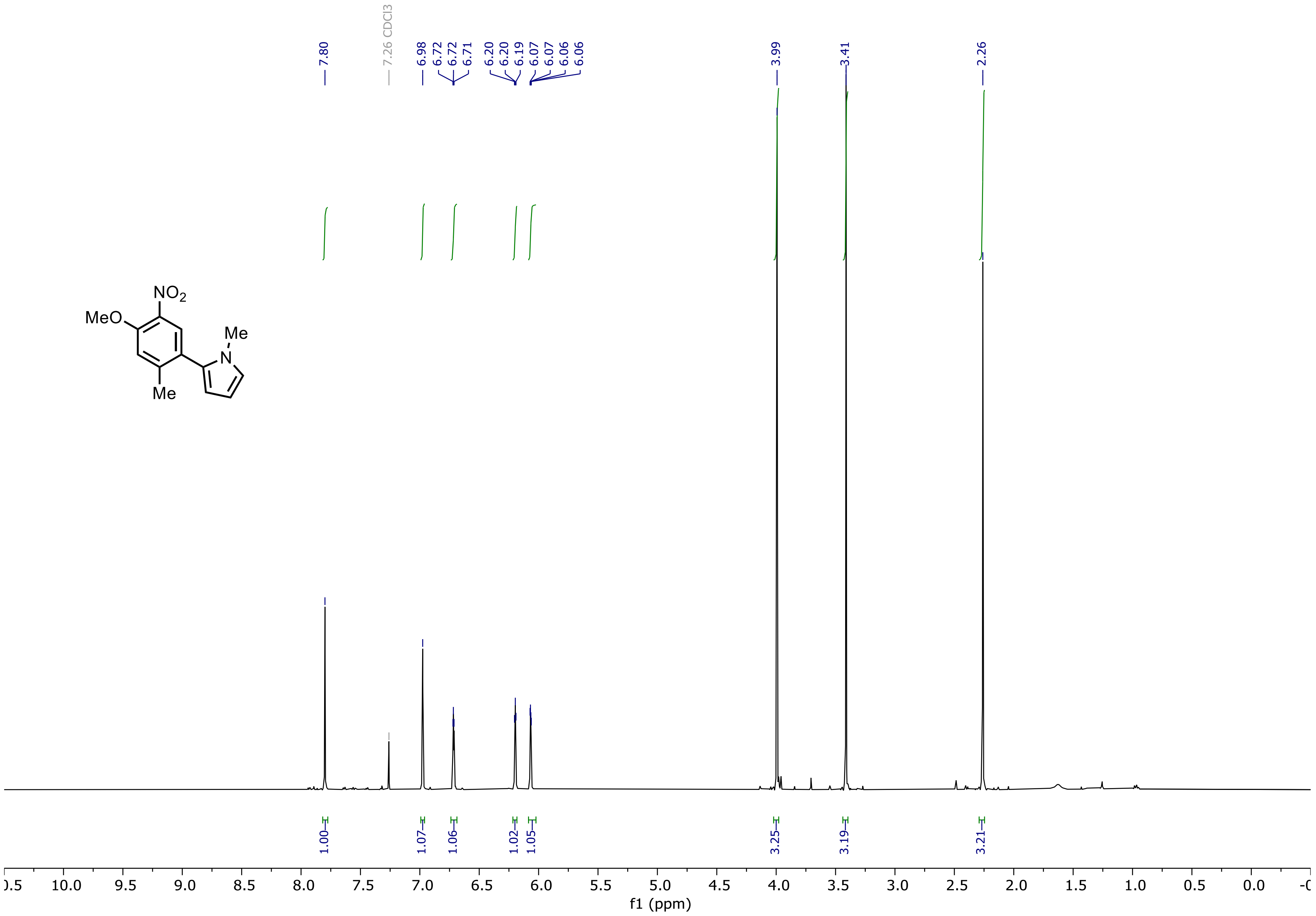

^{13}C NMR of 2-(4-methoxy-2-methyl-5-nitrophenyl)-1-methyl-1H-pyrrole (16)

$CDCl_3$, 126 MHz, 298 K

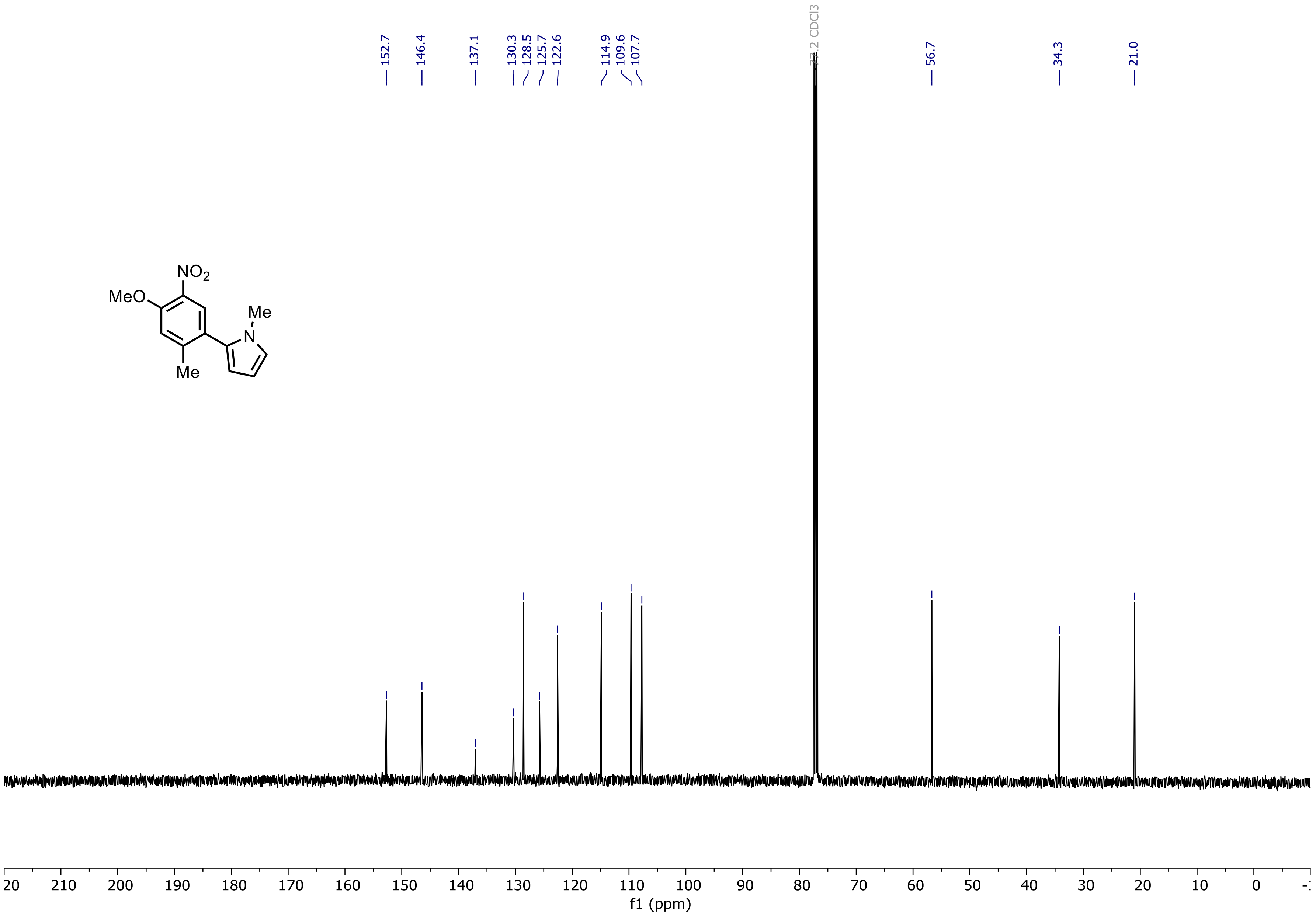

HSQC of 2-(4-methoxy-2-methyl-5-nitrophenyl)-1-methyl-1H-pyrrole (16)

$CDCl_3$, 600 MHz, 298 K

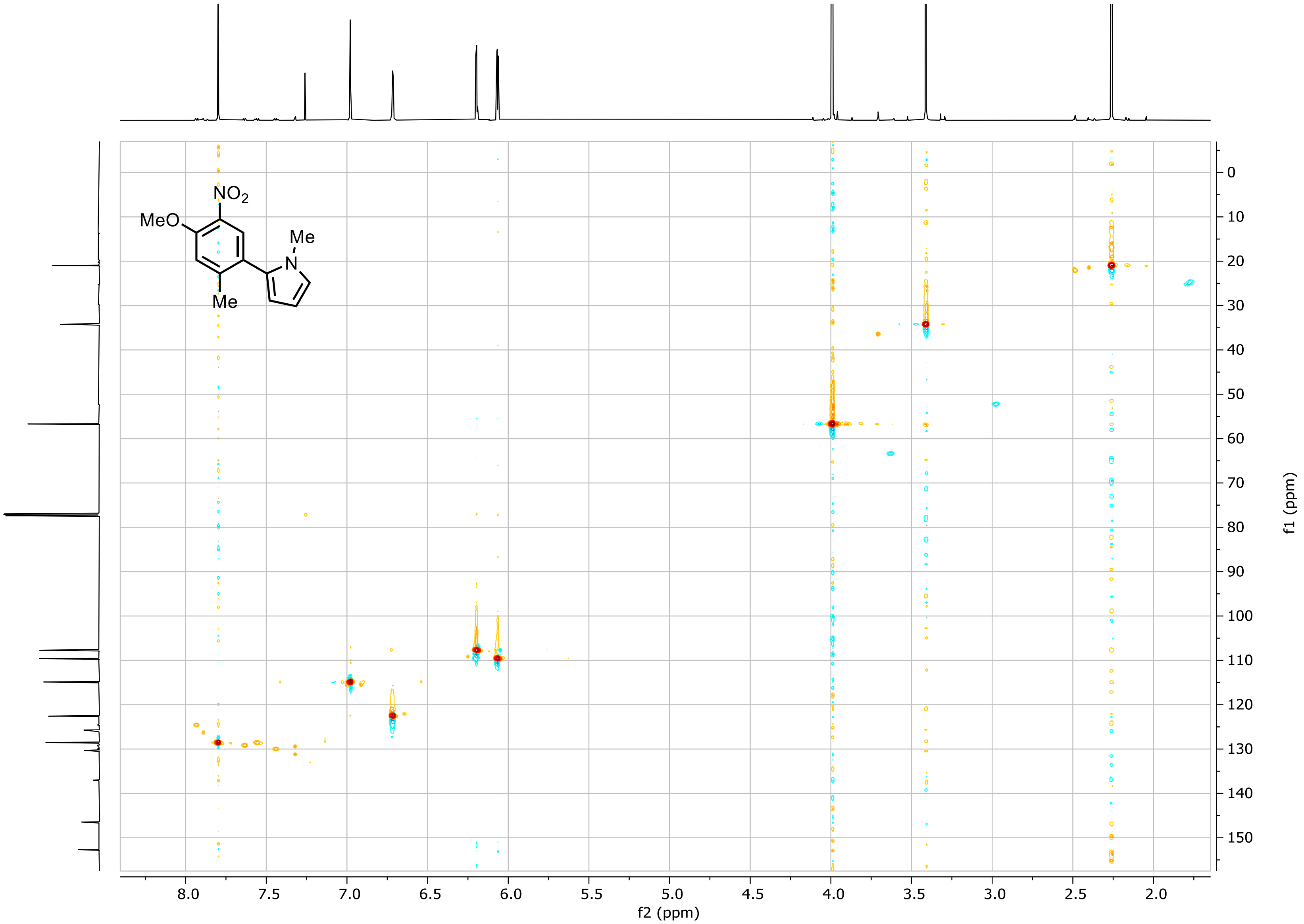

HMBC of 2-(4-methoxy-2-methyl-5-nitrophenyl)-1-methyl-1H-pyrrole (16)

$CDCl_3$, 600 MHz, 298 K

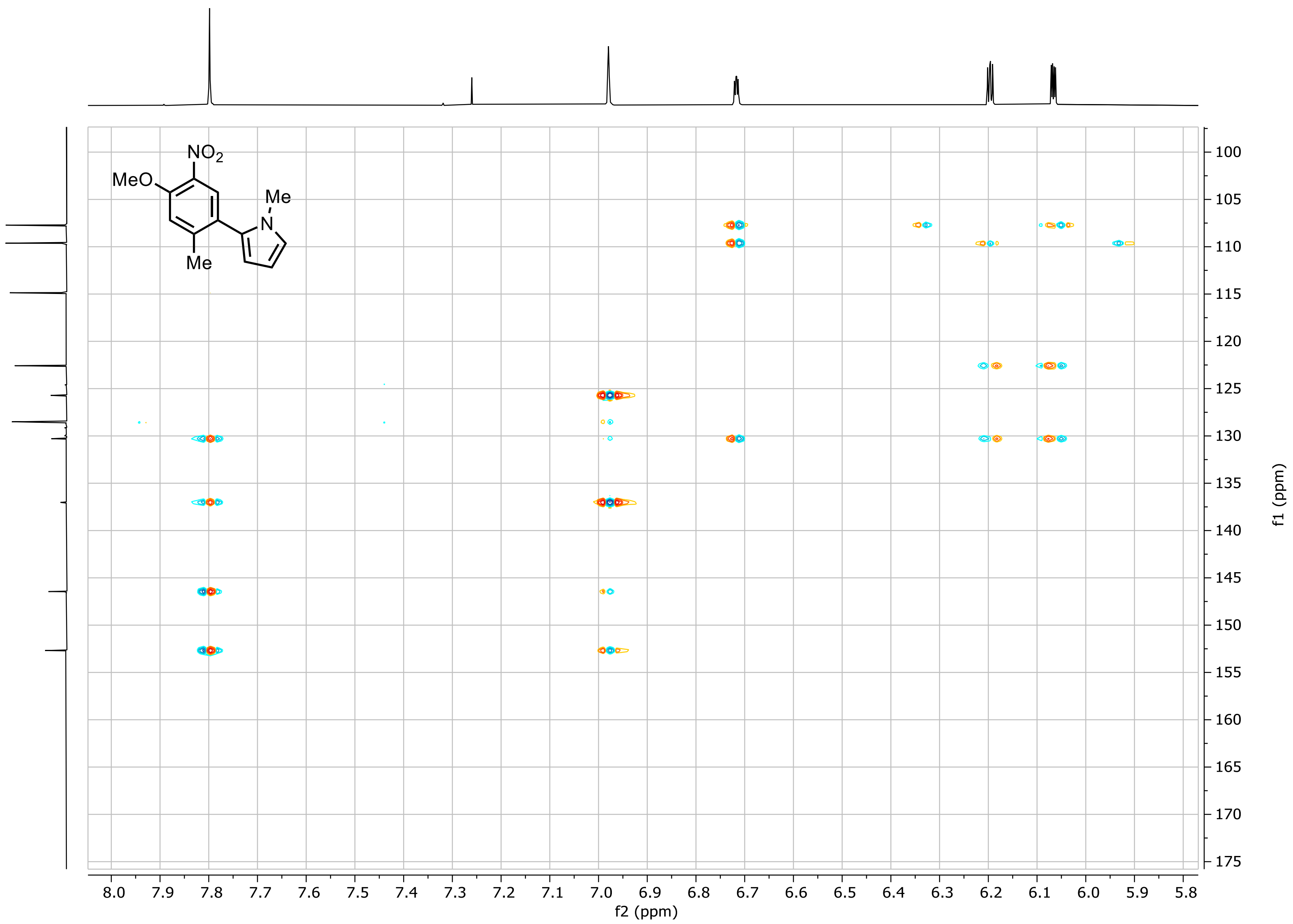

^{1}H NMR of 5-([1,1'-biphenyl]-4-yl)-1-methyl-1H-pyrazole (17)

$CDCl_3$, 500 MHz, 298 K

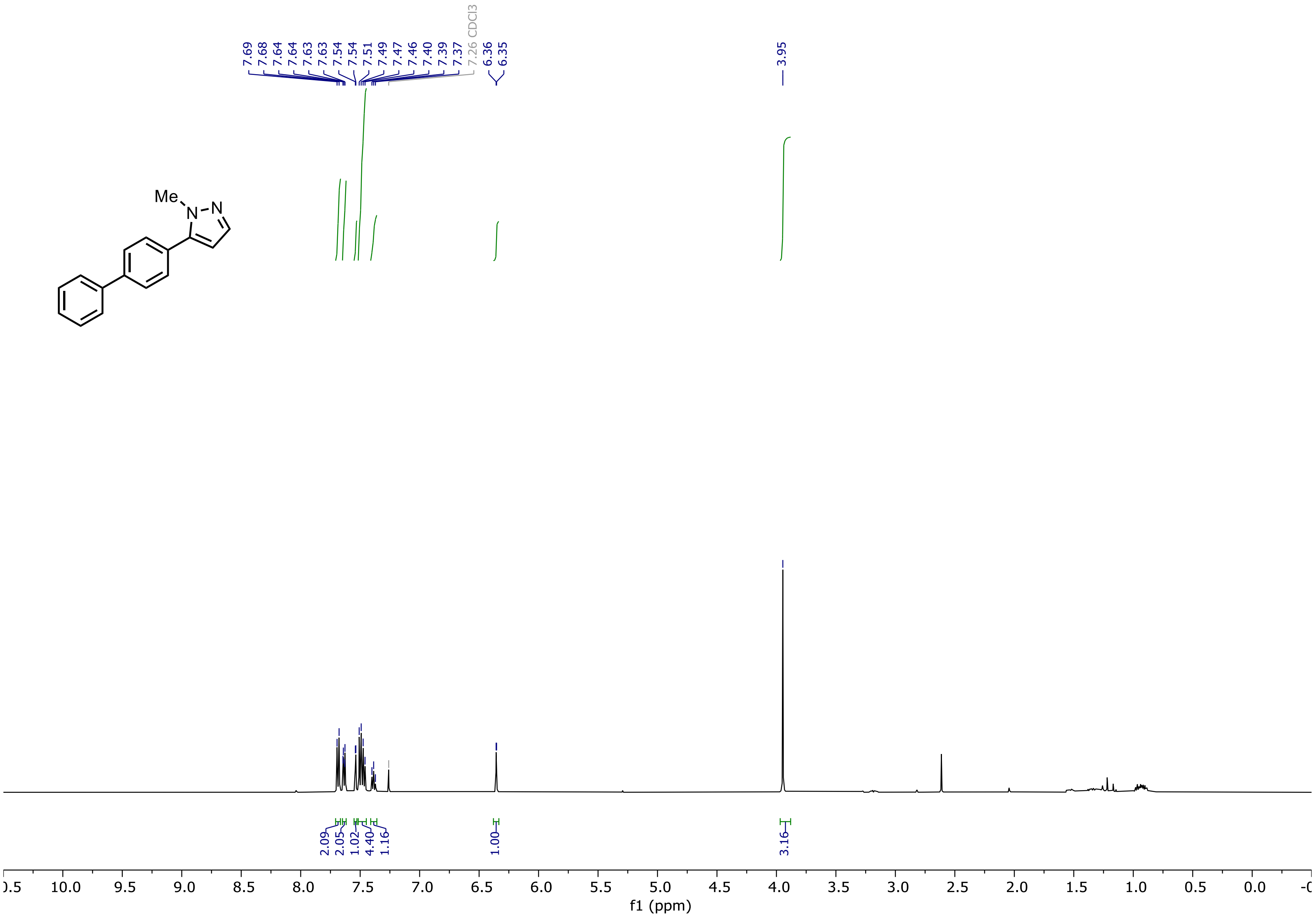

^{13}C NMR of 5-([1,1'-biphenyl]-4-yl)-1-methyl-1H-pyrazole (17)

$CDCl_3$, 126 MHz, 298 K

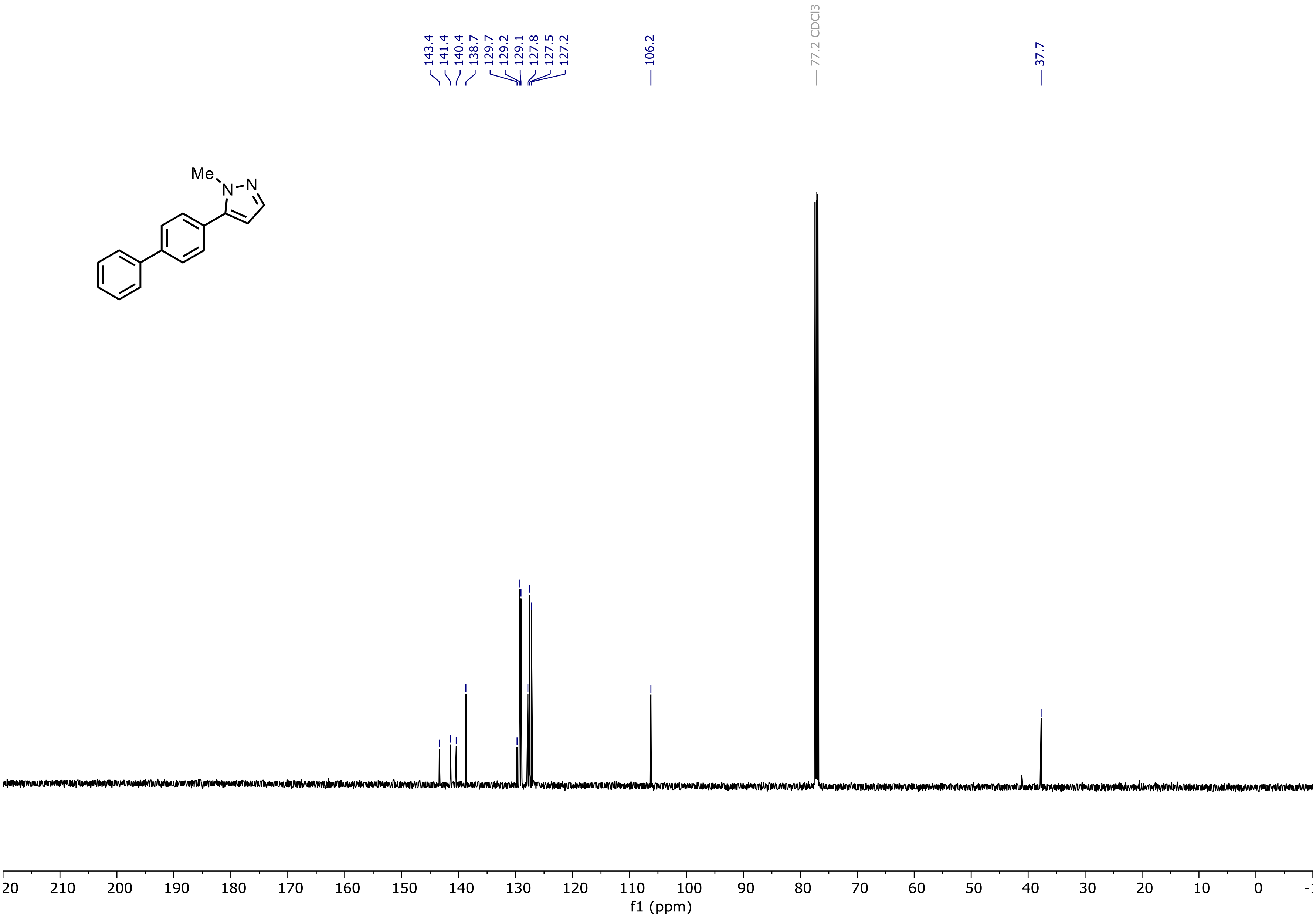

^{1}H NMR of famoxadone *N*-methyl pyrazole derivative (18)

$CDCl_3$, 500 MHz, 298 K

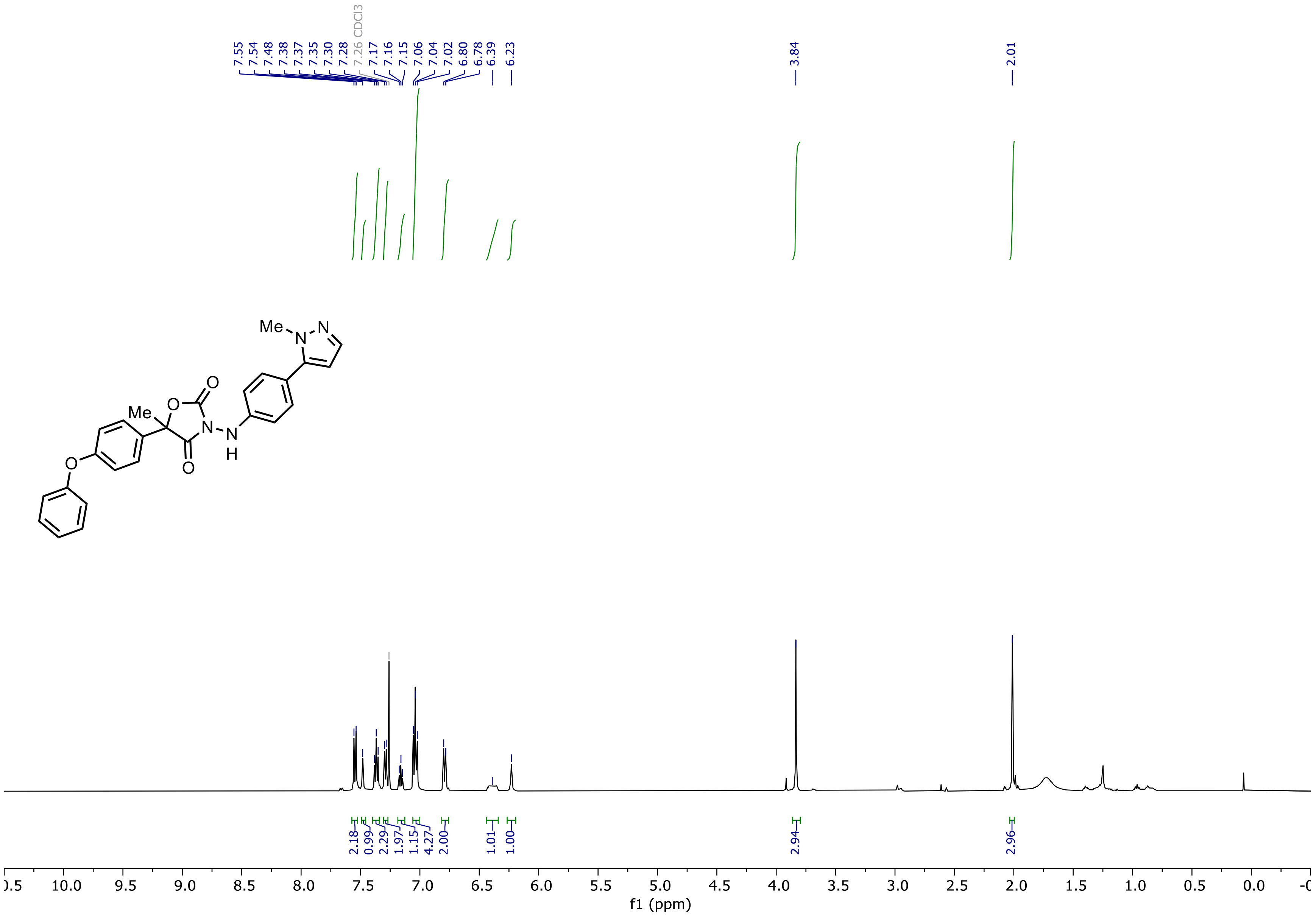

^{13}C NMR of famoxadone N-methyl pyrazole derivative (18)

$CDCl_3$, 126 MHz, 298 K

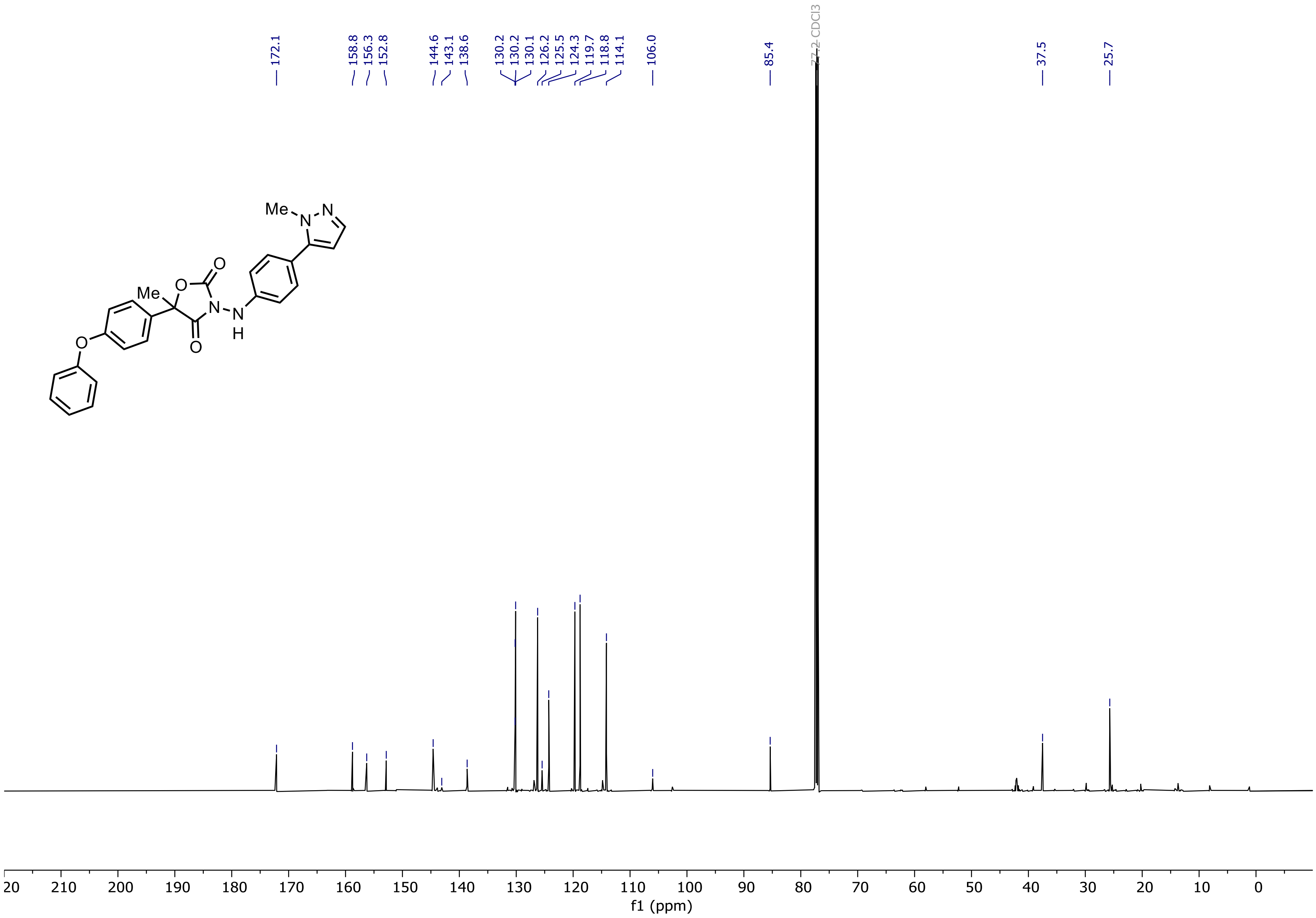

^{1}H NMR of 8-(4-(4-bromophenoxy)phenyl)-1,3,7-trimethyl-3,7-dihydro-1H-purine-2,6-dione (19)

$CDCl_3$, 500 MHz, 298 K

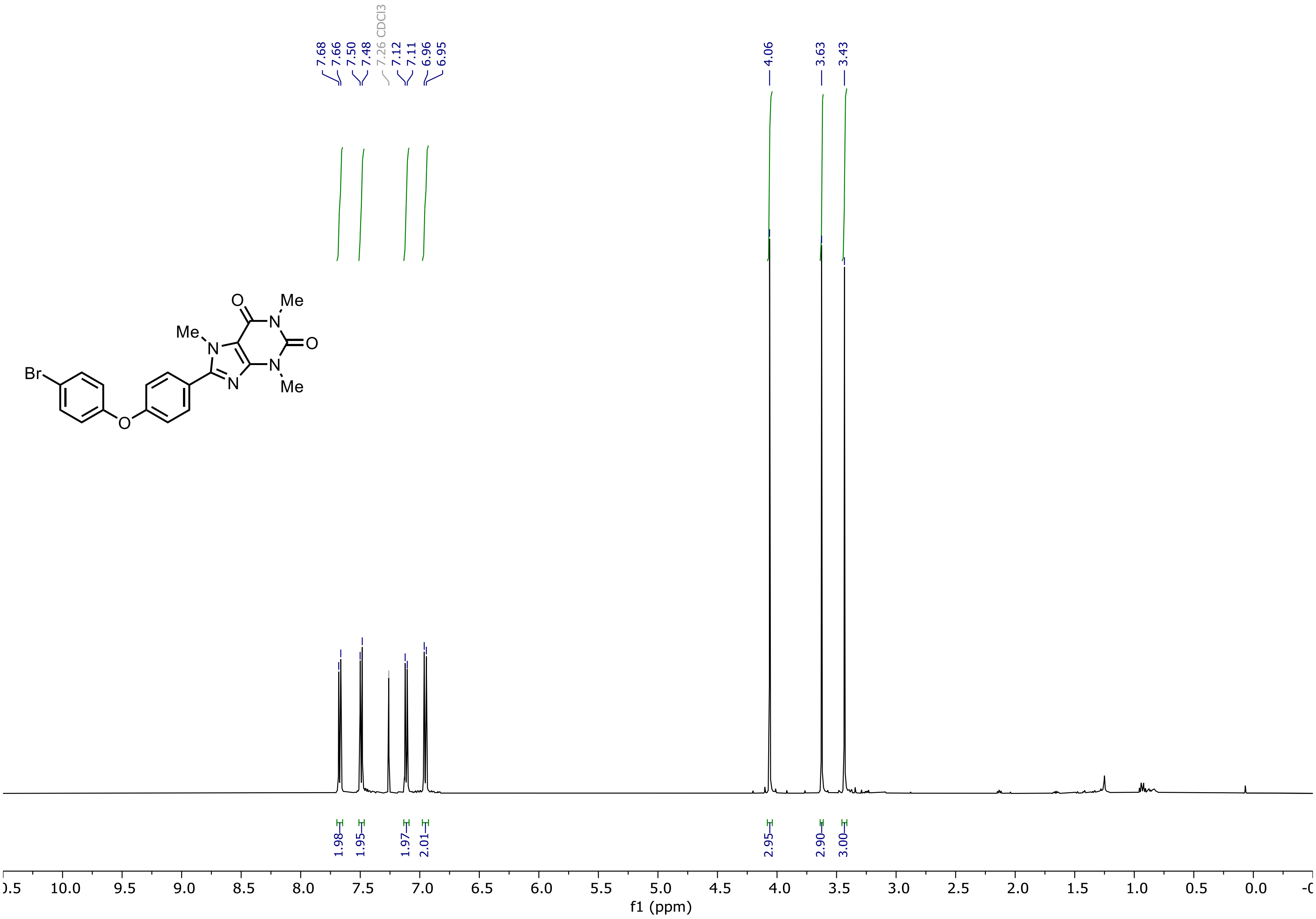

^{13}C NMR of 8-(4-(4-bromophenoxy)phenyl)-1,3,7-trimethyl-3,7-dihydro-1H-purine-2,6-dione (19)

$CDCl_3$, 126 MHz, 298 K

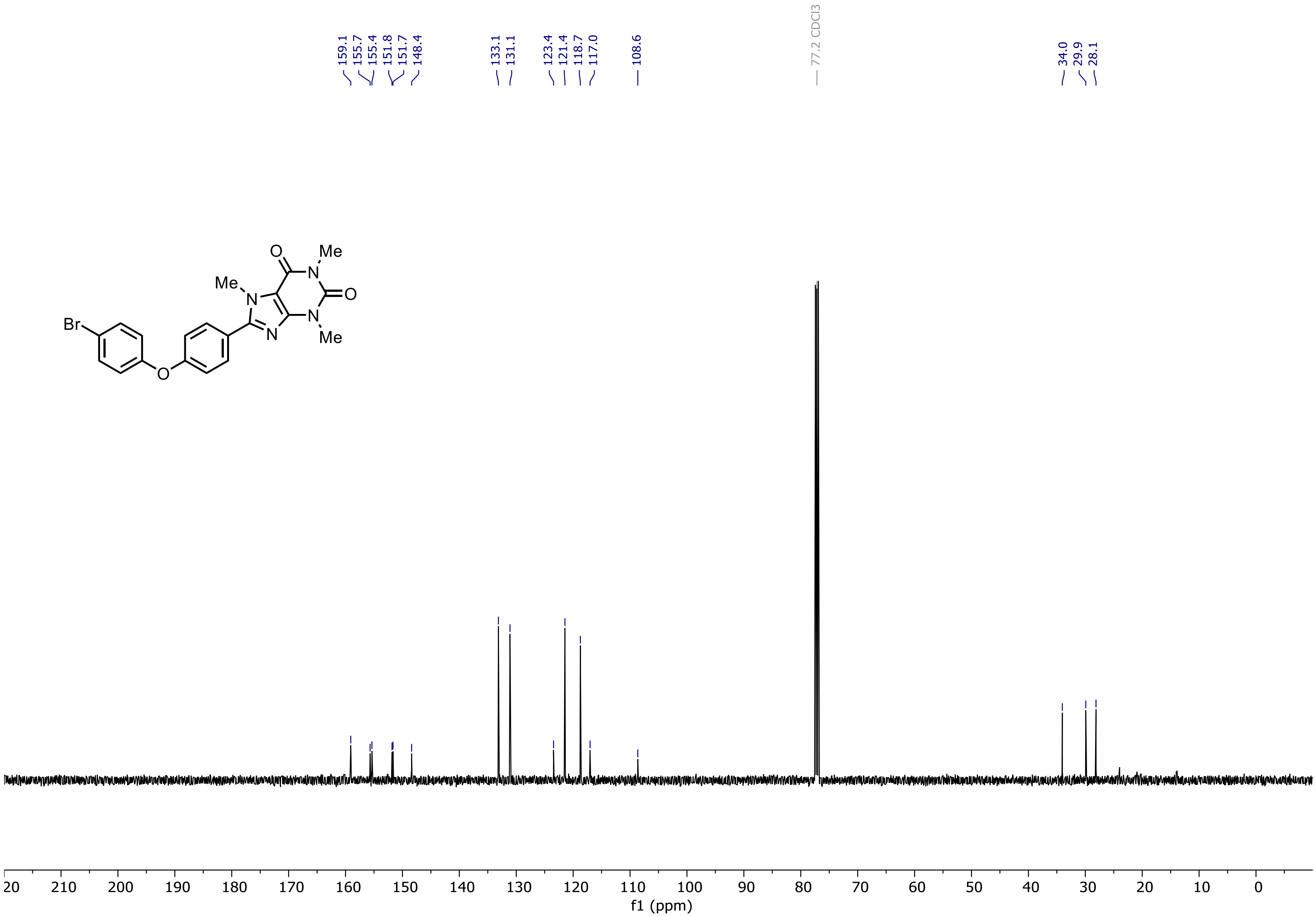

^{1}H NMR of 1-methyl-2-(thieno[3,2-b]thiophen-2-yl)-1H-pyrrole (20)

CD_3CN, 300 MHz, 298 K

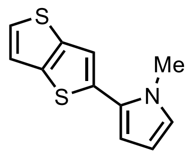

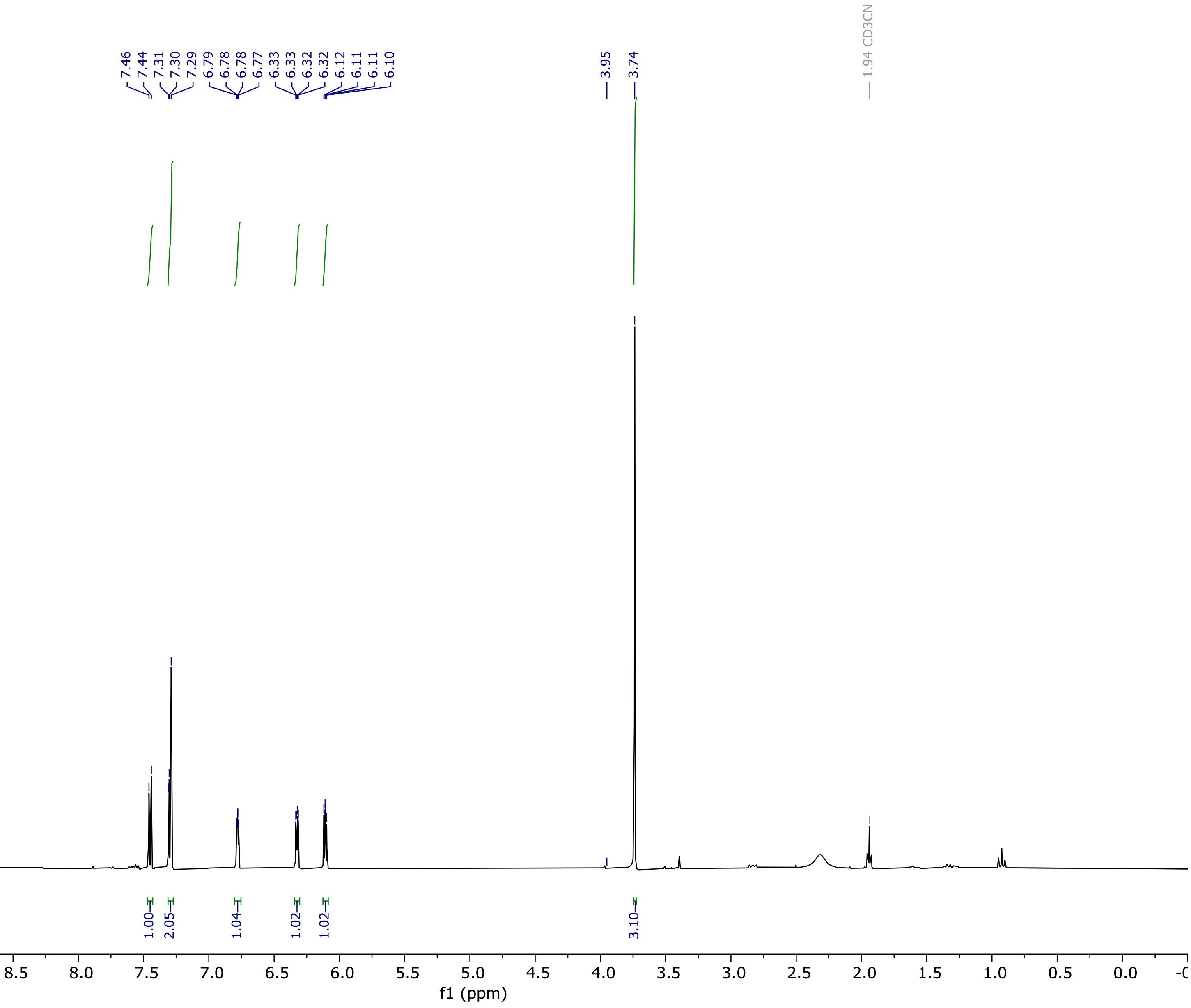

^{13}C NMR of 1-methyl-2-(thieno[3,2-b]thiophen-2-yl)-1H-pyrrole (20)

CD_3CN, 75 MHz, 298 K

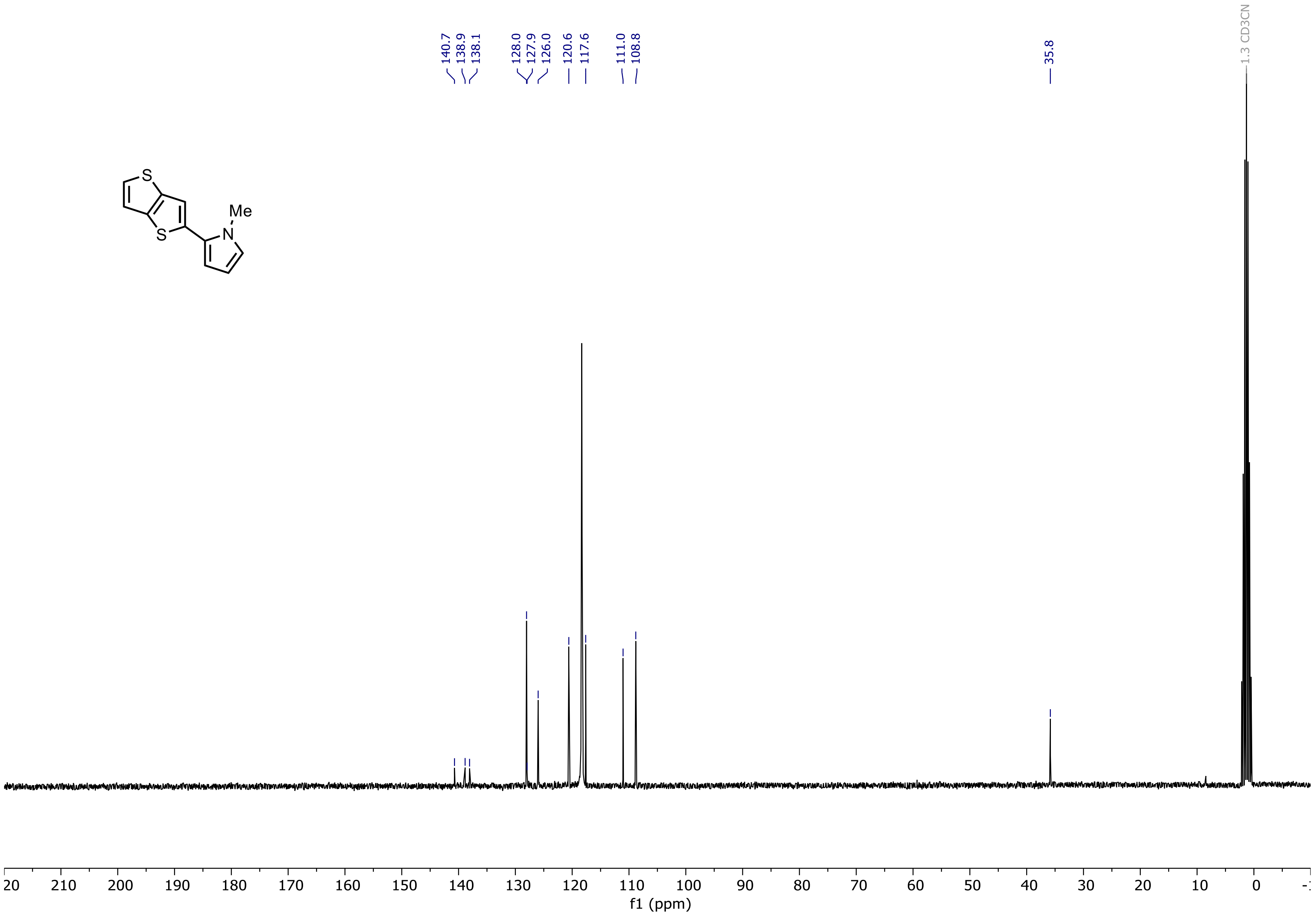

^{1}H NMR of 1-(5-(2,6-dimethoxypyridin-3-yl)-1-methyl-1H-pyrrol-2-yl)ethan-1-one (21)

CD_3CN, 300 MHz, 298 K

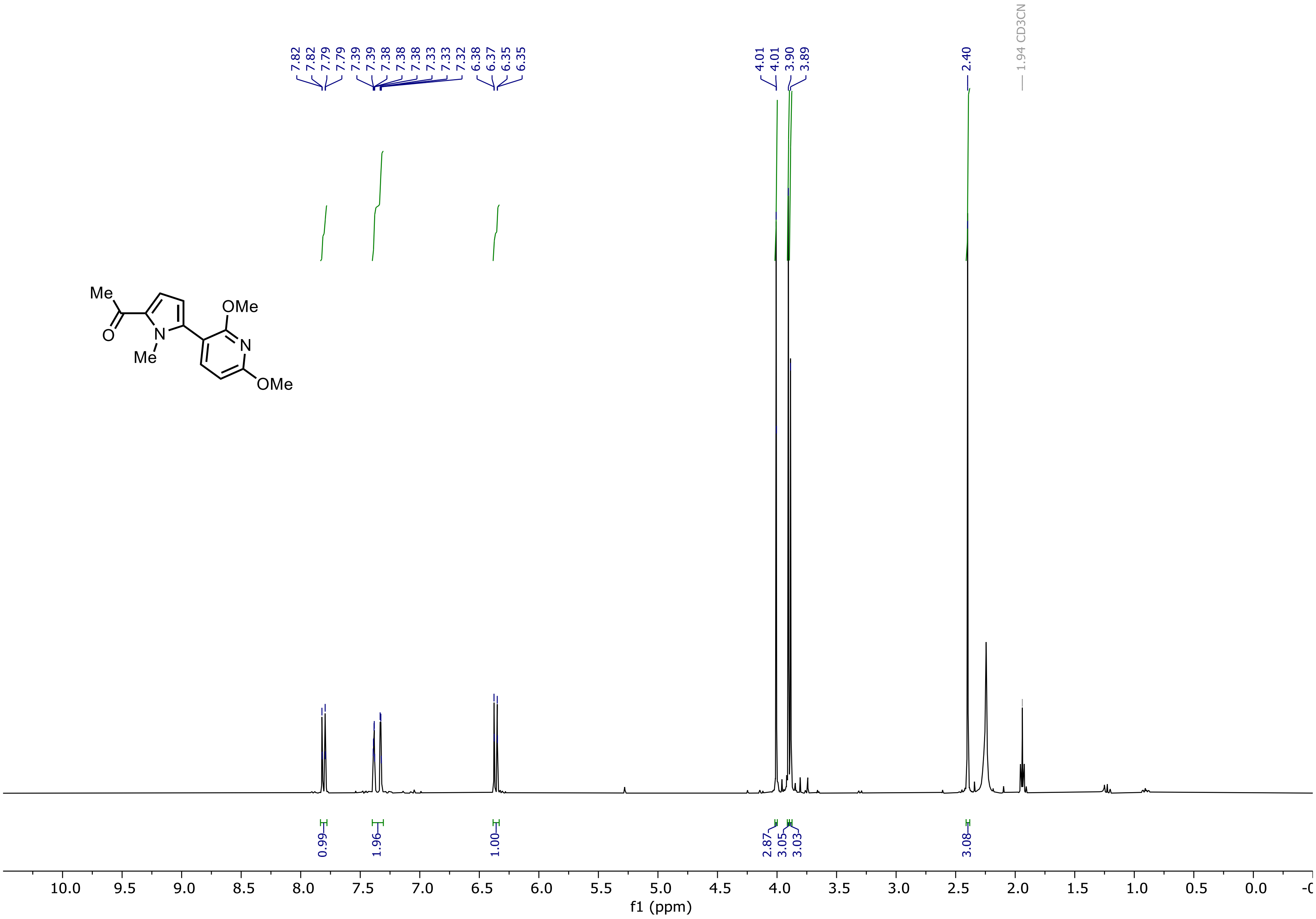

^{13}C NMR of 1-(5-(2,6-dimethoxypyridin-3-yl)-1-methyl-1H-pyrrol-2-yl)ethan-1-one (21)

CD_3CN, 126 MHz, 298 K

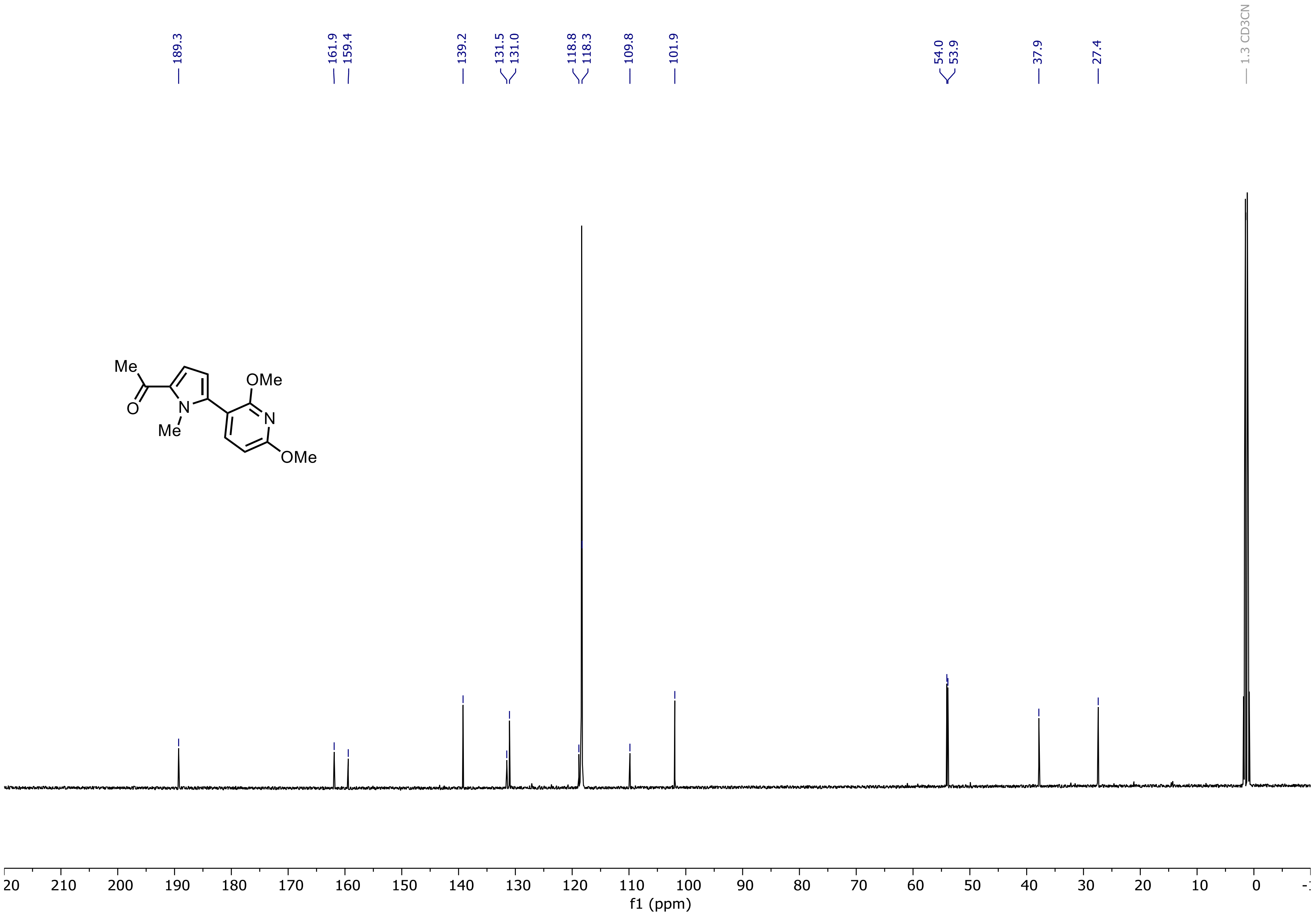

^{1}H NMR of 2-methoxy-5-(1-methyl-1H-pyrrol-2-yl)pyridine (22)

CD_3CN, 500 MHz, 298 K

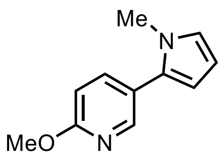

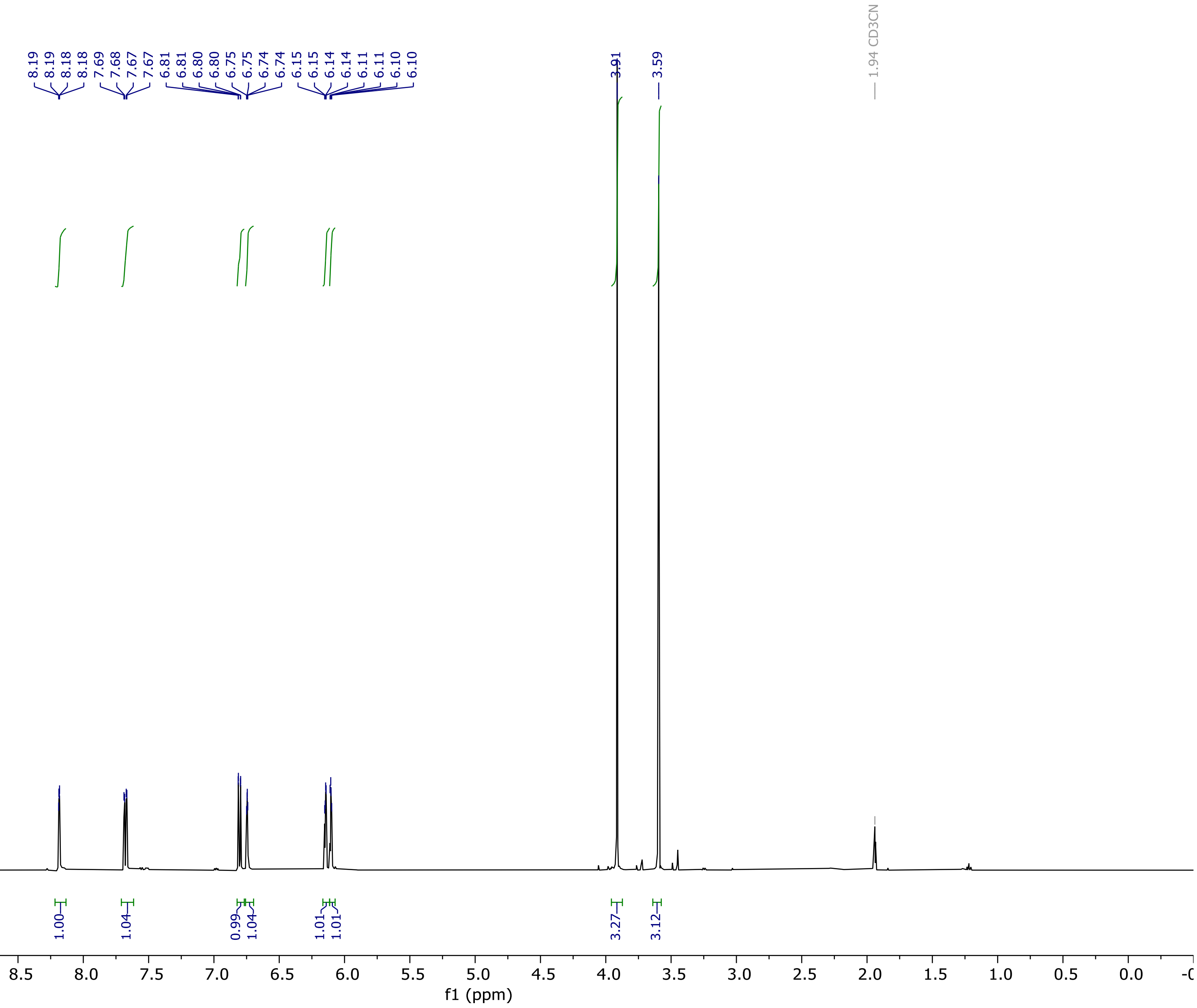

^{13}C NMR of 2-methoxy-5-(1-methyl-1H-pyrrol-2-yl)pyridine (22)

CD_3CN, 126 MHz, 298 K

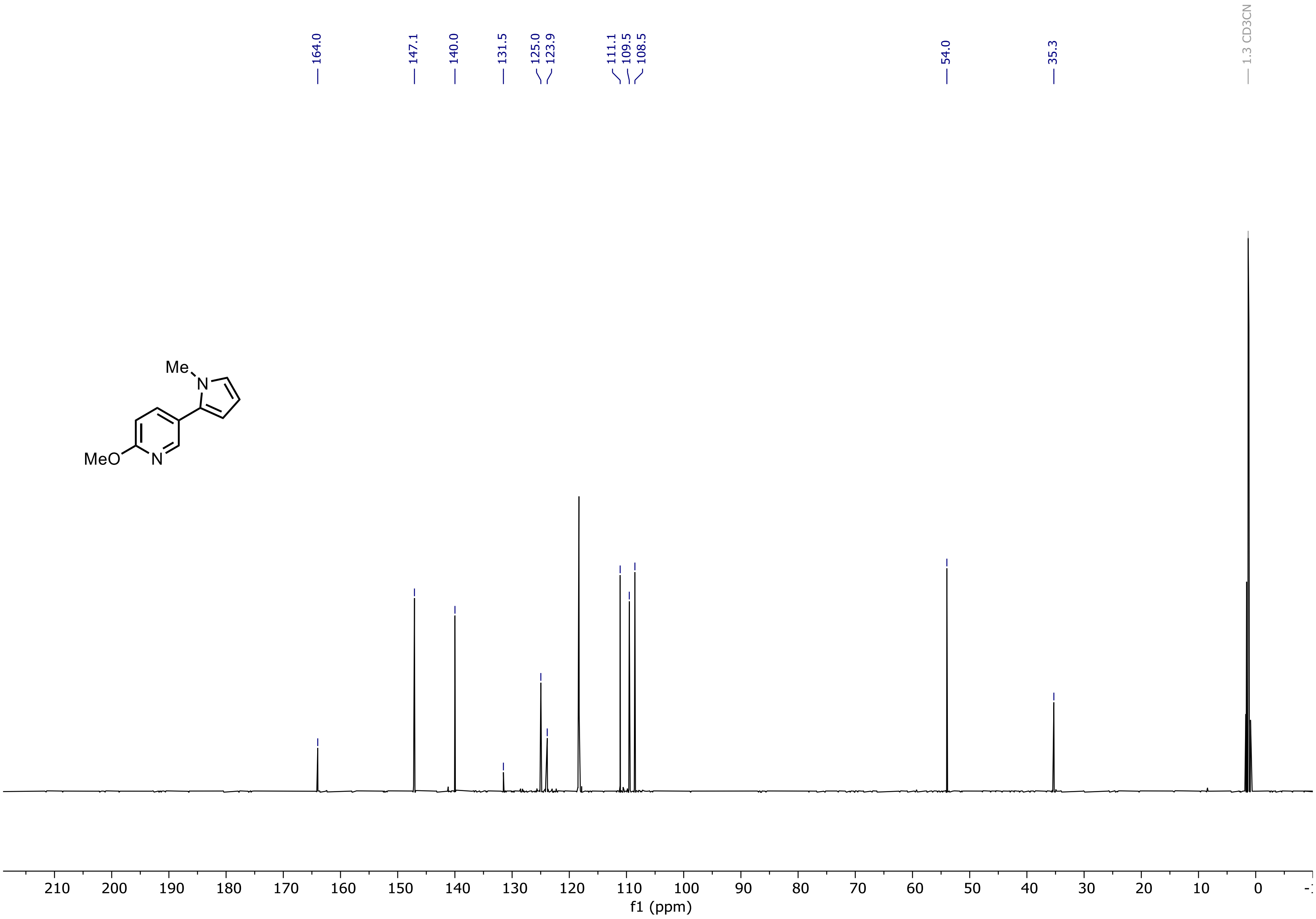

^{1}H NMR of 1-(1,1'-dimethyl-1H,1'H-[2,2'-bipyrrol]-5-yl)ethan-1-one (23)

CD$_3$CN, 500 MHz, 298 K

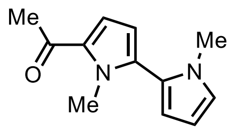

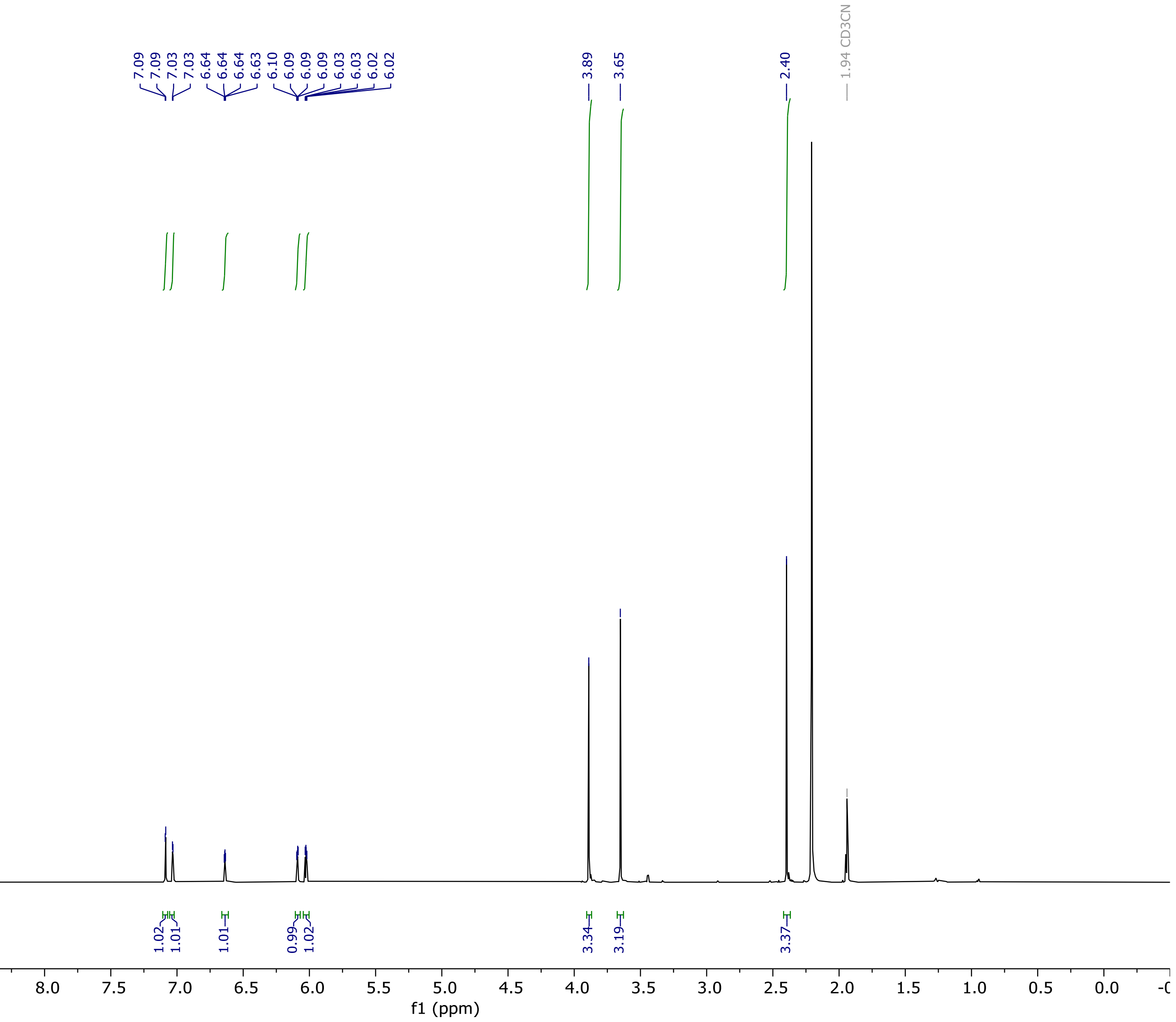

^{13}C NMR of 1-(1,1'-dimethyl-1H,1'H-[2,2'-bipyrrol]-5-yl)ethan-1-one (23)

CD_3CN, 126 MHz, 298 K

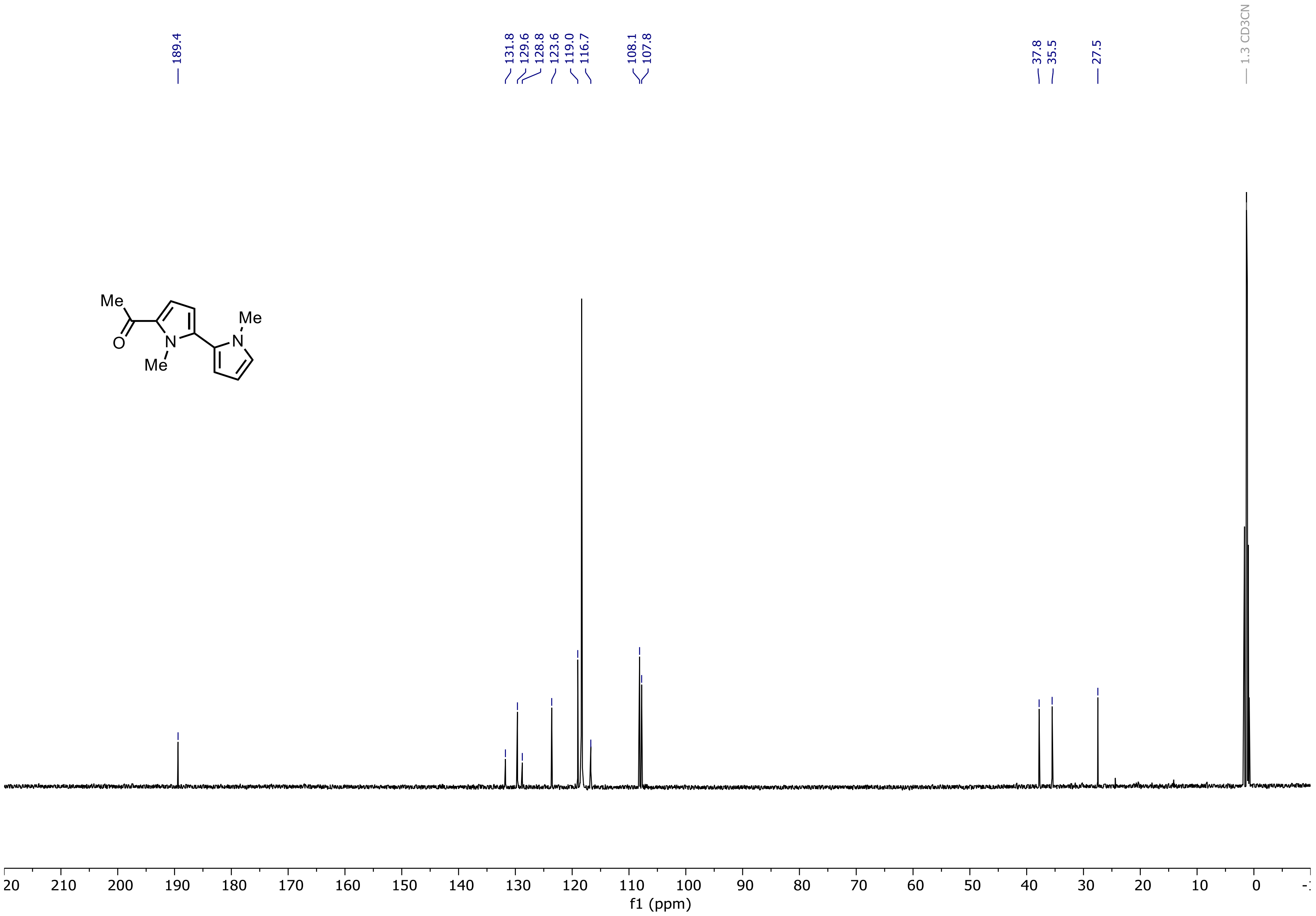

^{1}H NMR of 1-methyl-2-(5-phenylthiophen-2-yl)-1H-pyrrole (24)

CD_3CN, 500 MHz, 298 K

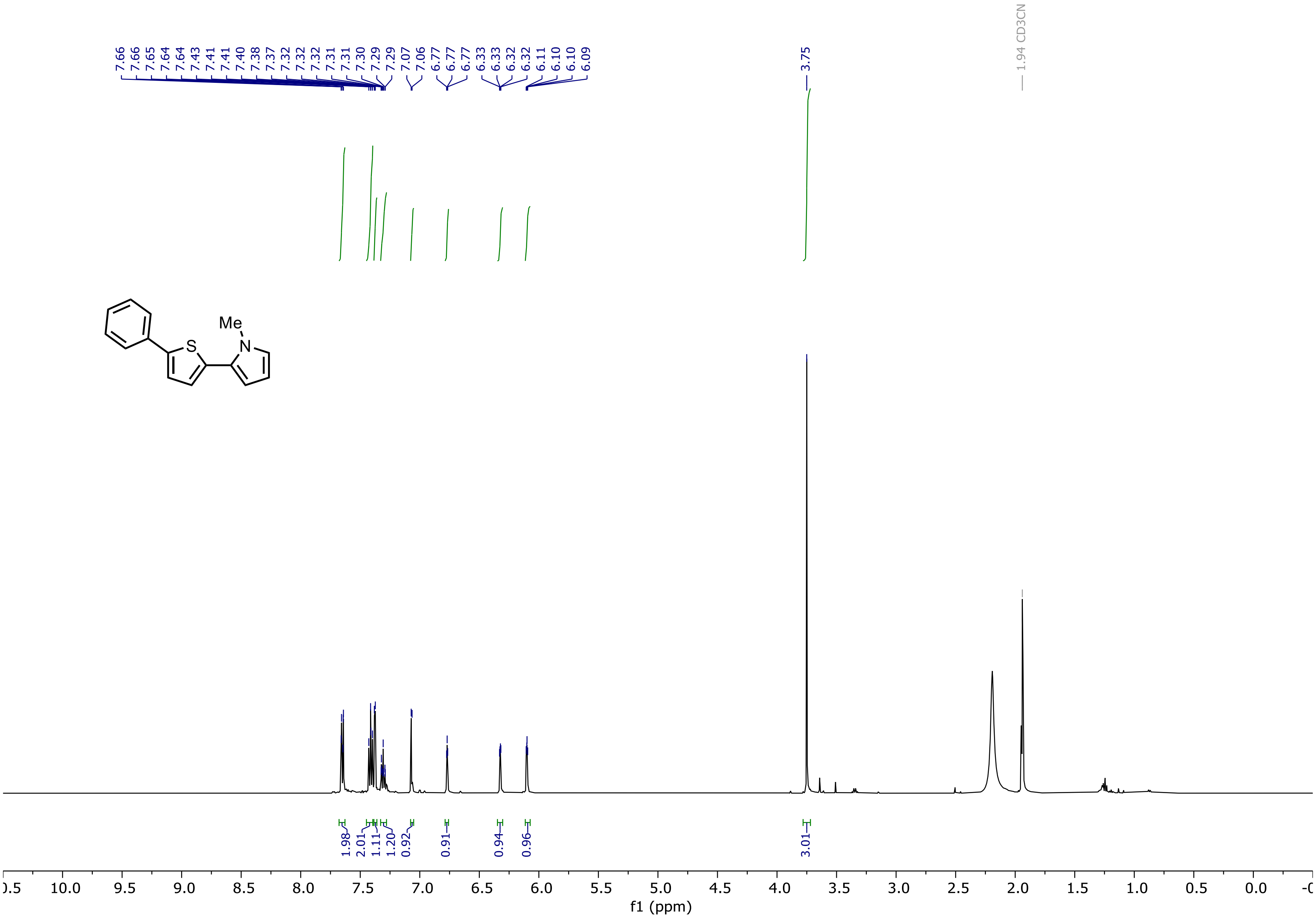

^{13}C NMR of 1-methyl-2-(5-phenylthiophen-2-yl)-1H-pyrrole (24)

CD_3CN, 126 MHz, 298 K

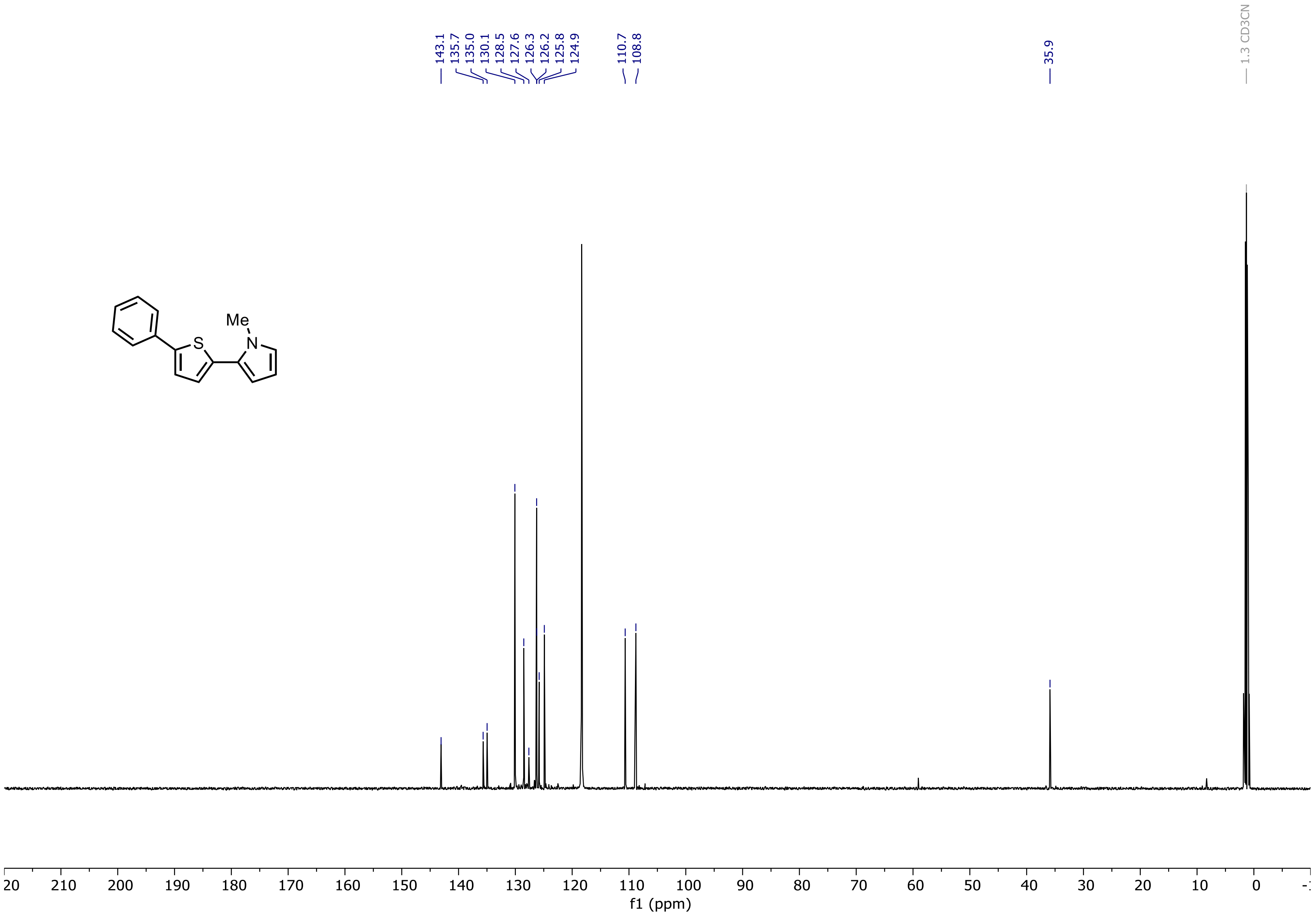

^{1}H NMR of ethyl 3-(5-(1-methyl-1H-pyrrol-2-yl)furan-2-yl)propanoate (25)

CD_3CN, 500 MHz, 298 K

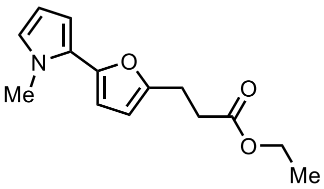

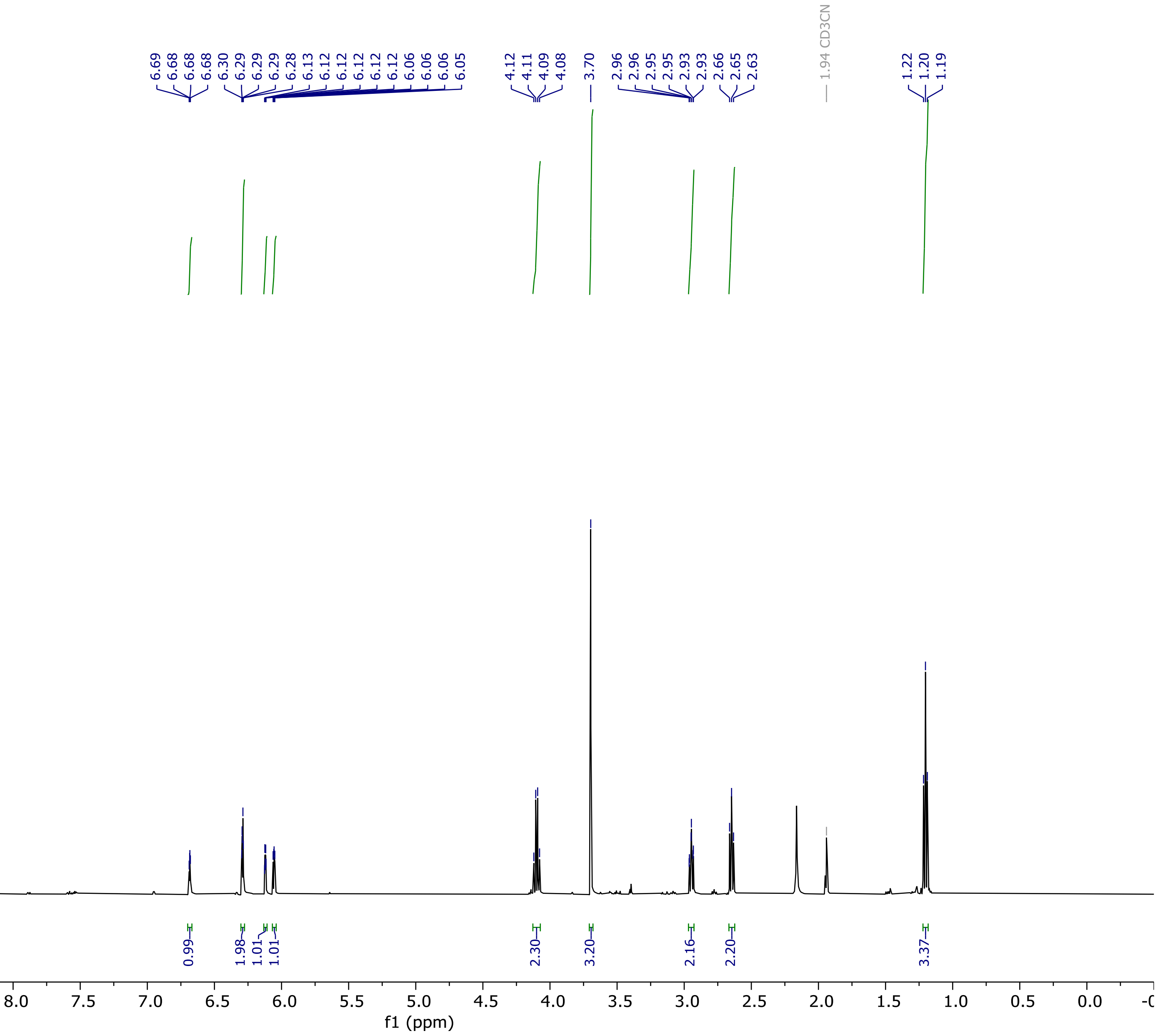

^{13}C NMR of ethyl 3-(5-(1-methyl-1H-pyrrol-2-yl)furan-2-yl)propanoate (25)

$CDCl_3$, 75 MHz, 298 K

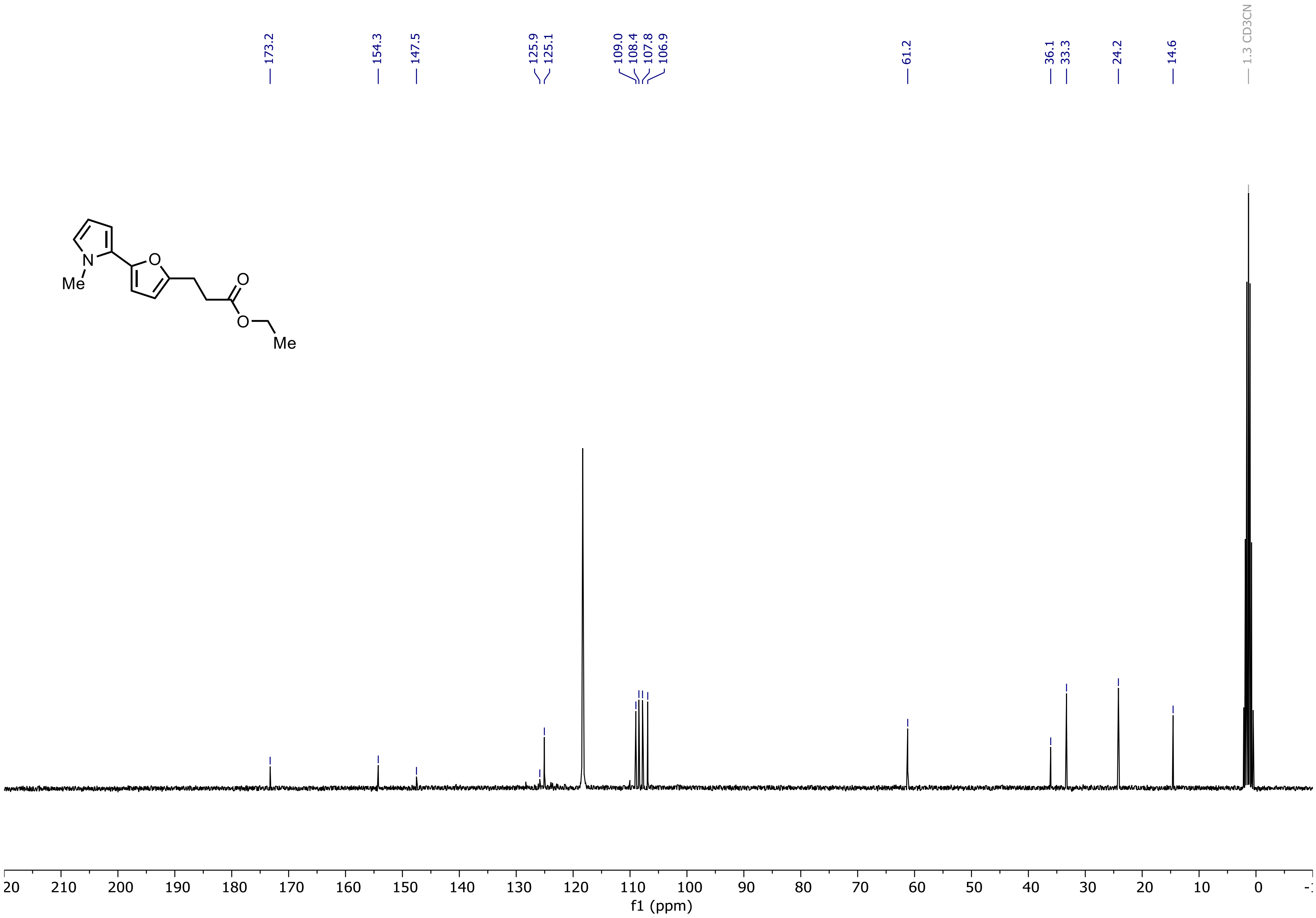

^{1}H NMR of 1-methyl-4-(1-methyl-1H-pyrrol-2-yl)-1H-pyrazole (26)

CD_3CN, 500 MHz, 298 K

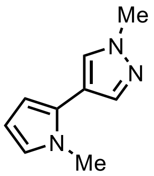

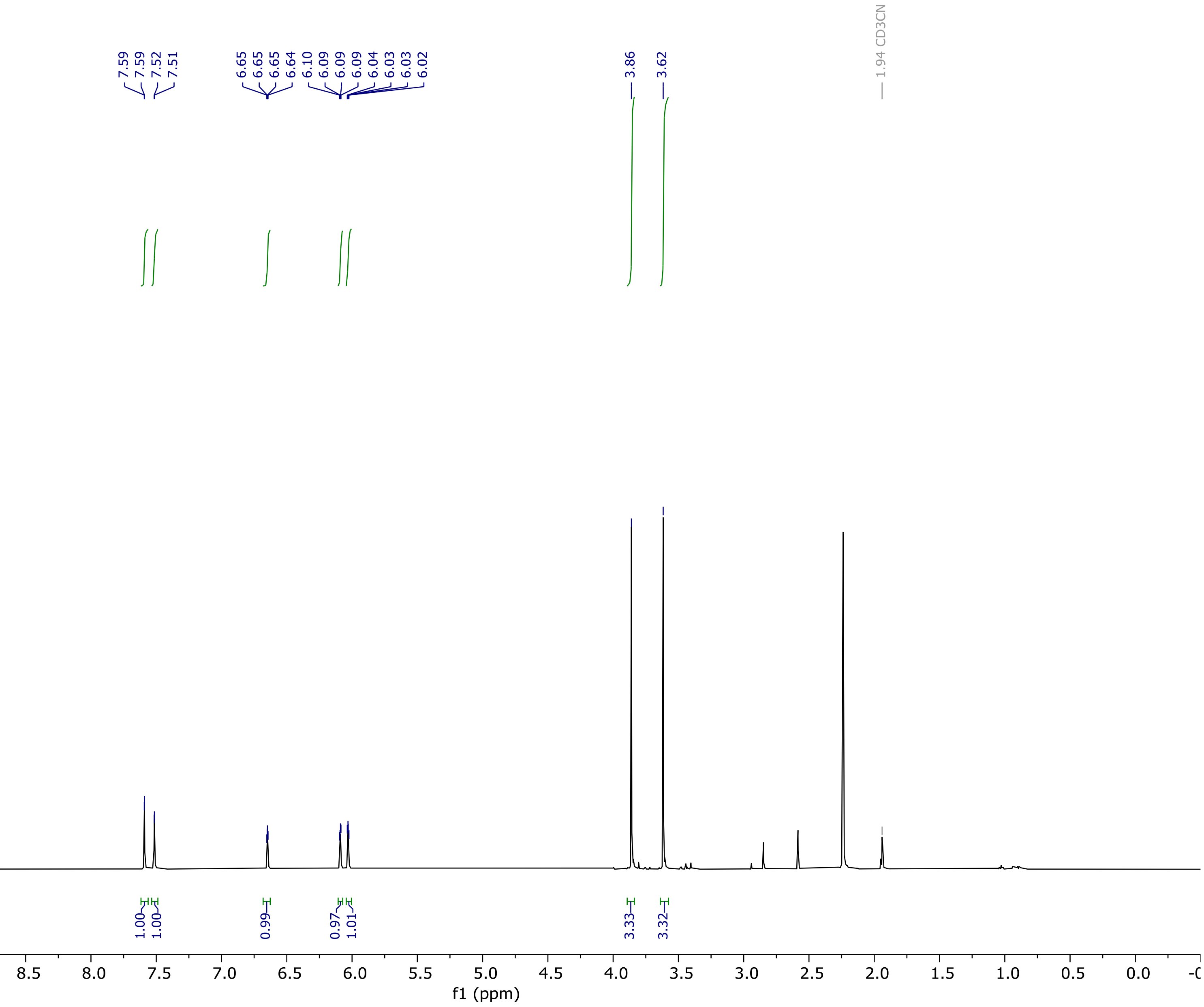

^{13}C NMR of 1-methyl-4-(1-methyl-1H-pyrrol-2-yl)-1H-pyrazole (26)

$CDCl_3$, 75 MHz, 298 K

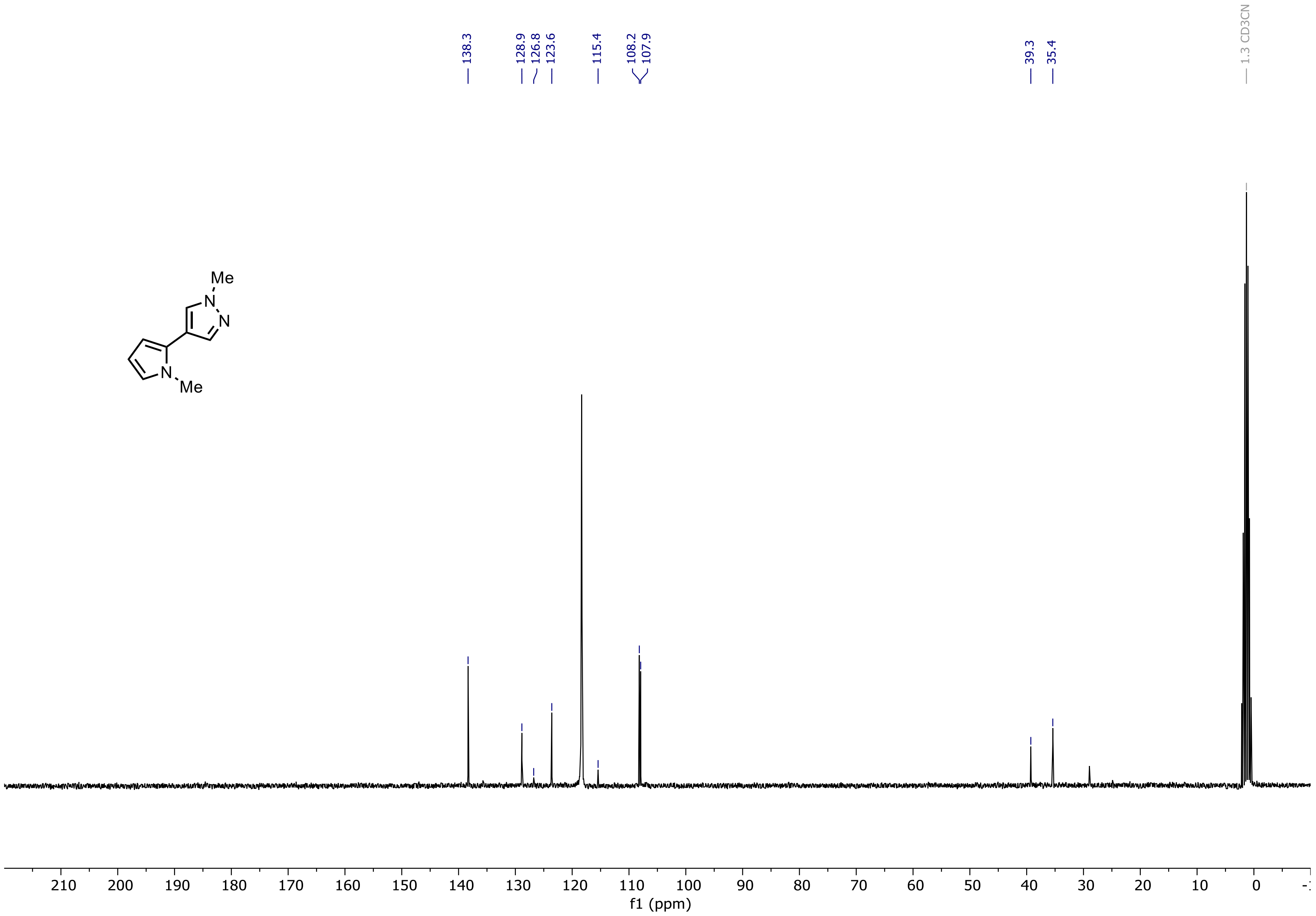

^{1}H NMR of 2-(4-(4-bromophenoxy)phenyl)-1-methyl-1H-imidazole (27a)

$CDCl_3$, 500 MHz, 298 K

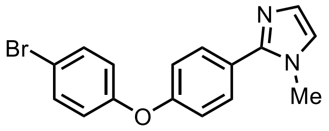

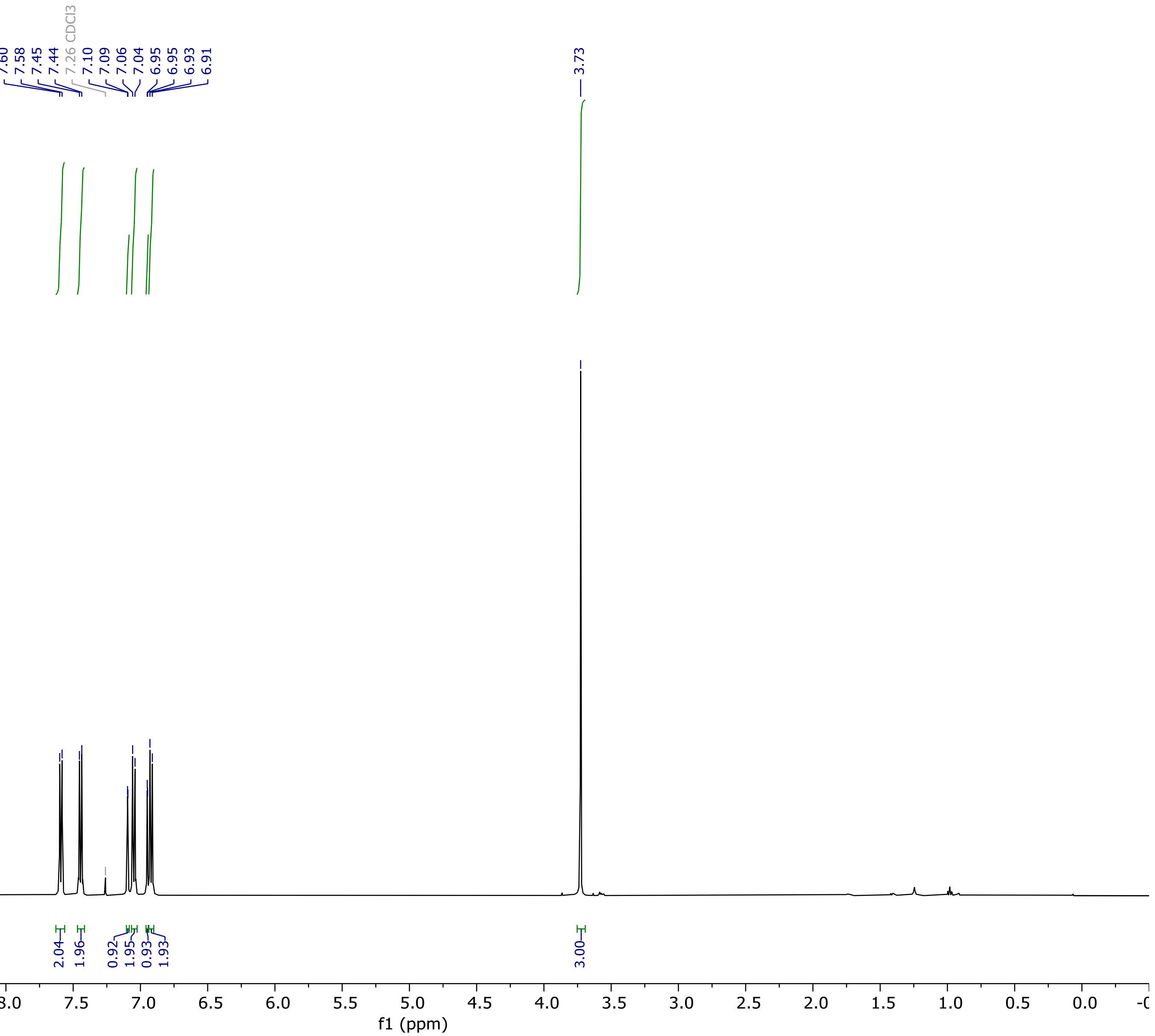

^{13}C NMR of 2-(4-(4-bromophenoxy)phenyl)-1-methyl-1H-imidazole (27a)

$CDCl_3$, 126 MHz, 298 K

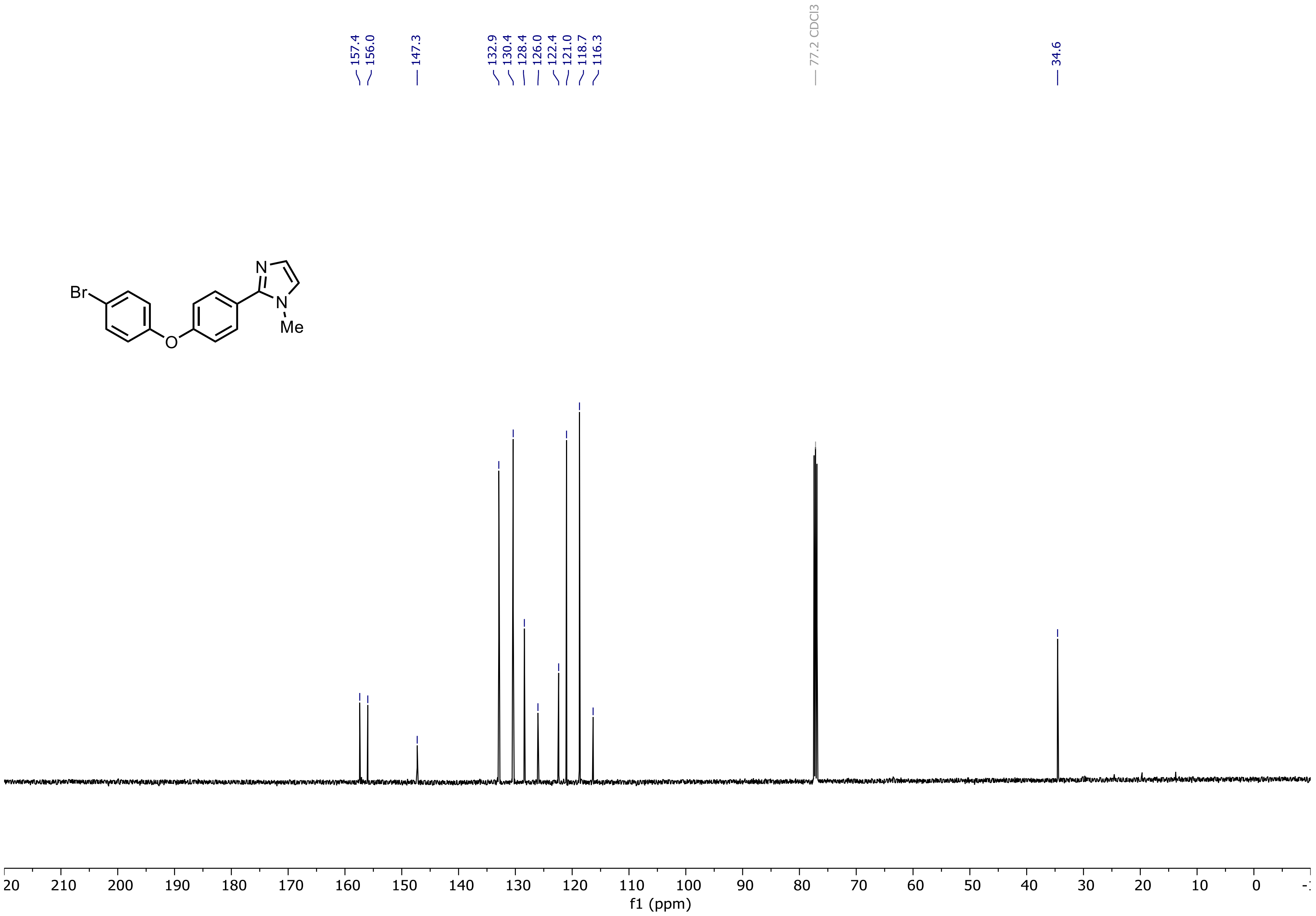

^{1}H NMR of 5-(4-(4-bromophenoxy)phenyl)-1-methyl-1H-imidazole (27b)

$CDCl_3$, 500 MHz, 298 K

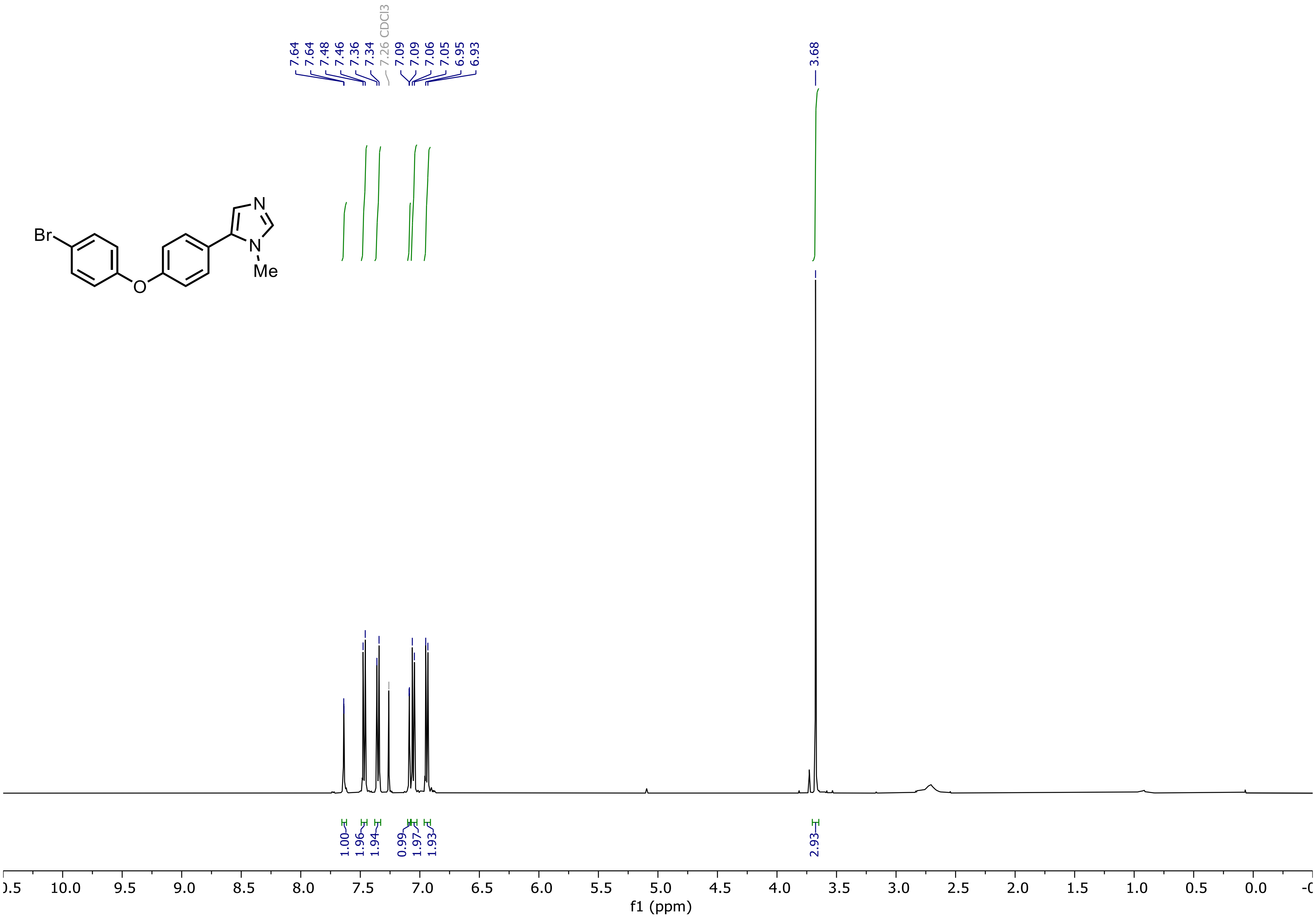

^{13}C NMR of 5-(4-(4-bromophenoxy)phenyl)-1-methyl-1H-imidazole (27b)

$CDCl_3$, 126 MHz, 298 K

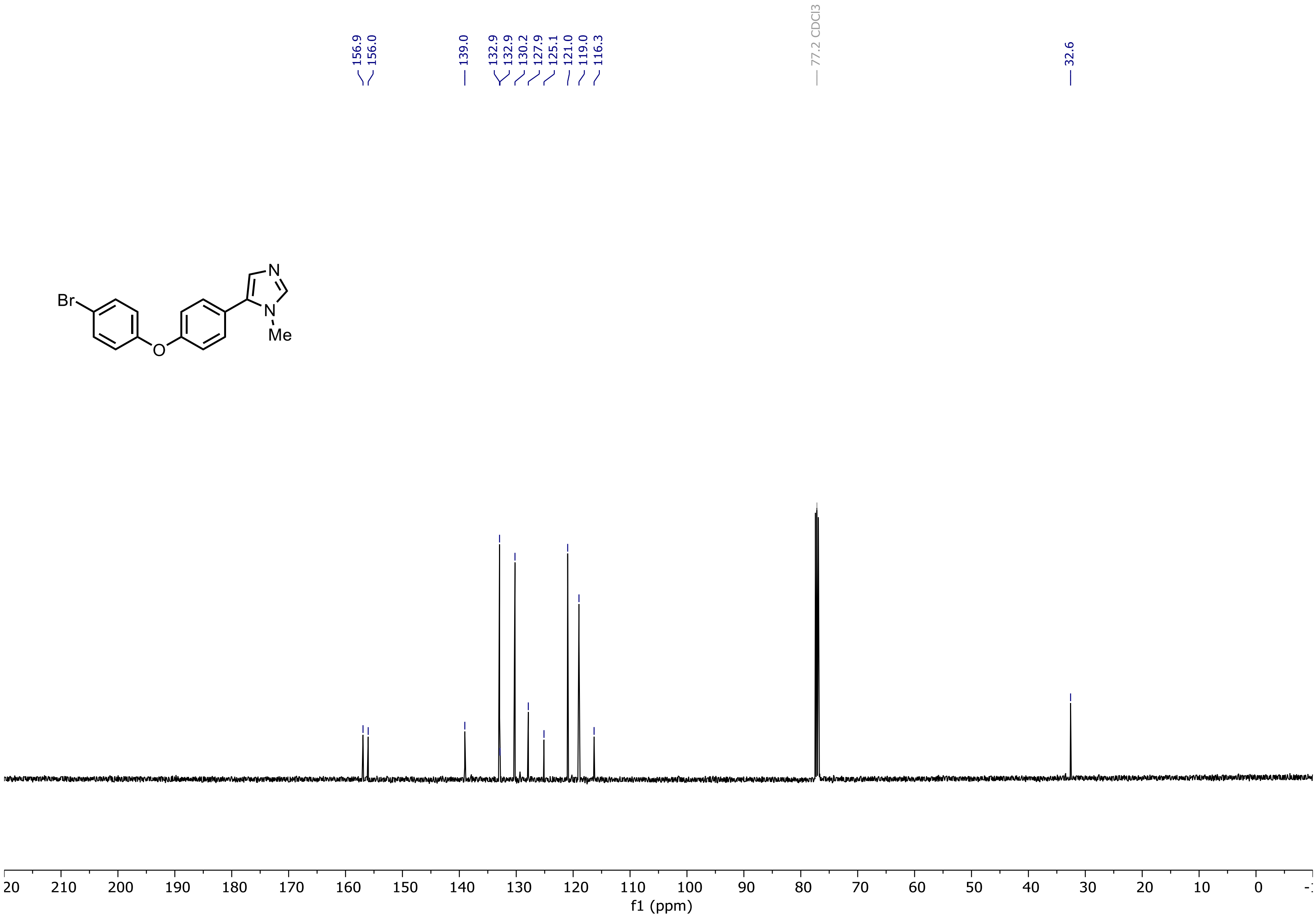

^{1}H NMR of 2-fluoro-6-(4-(4,4,5,5-tetramethyl-1,3,2-dioxaborolan-2-yl)phenoxy)benzonitrile (28)

$CDCl_3$, 500 MHz, 298 K

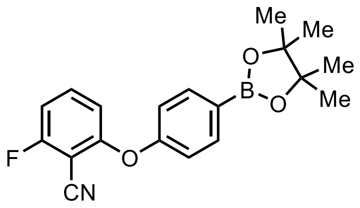

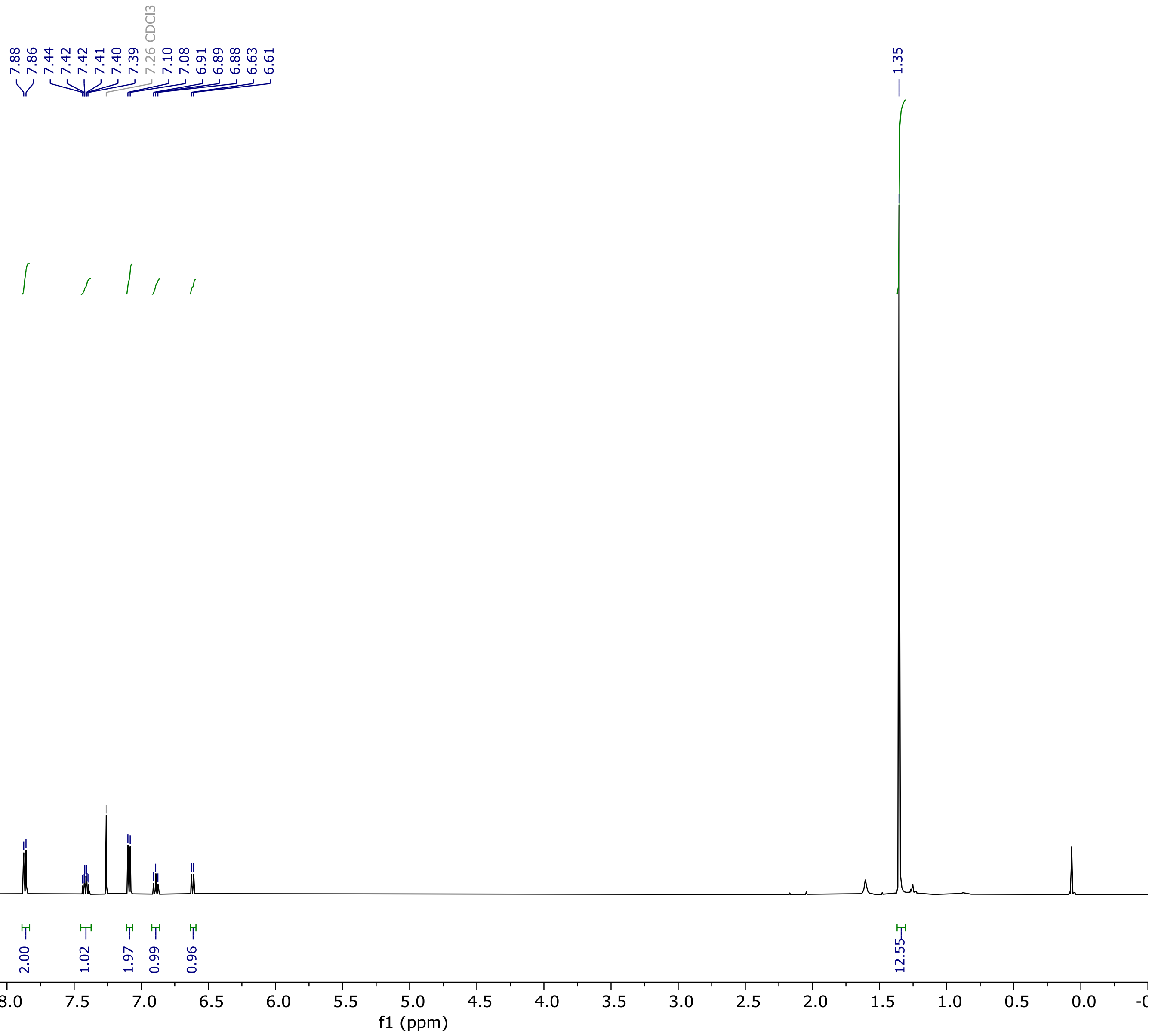

^{13}C NMR of 2-fluoro-6-(4-(4,4,5,5-tetramethyl-1,3,2-dioxaborolan-2-yl)phenoxy)benzonitrile (28)

$CDCl_3$, 126 MHz, 298 K

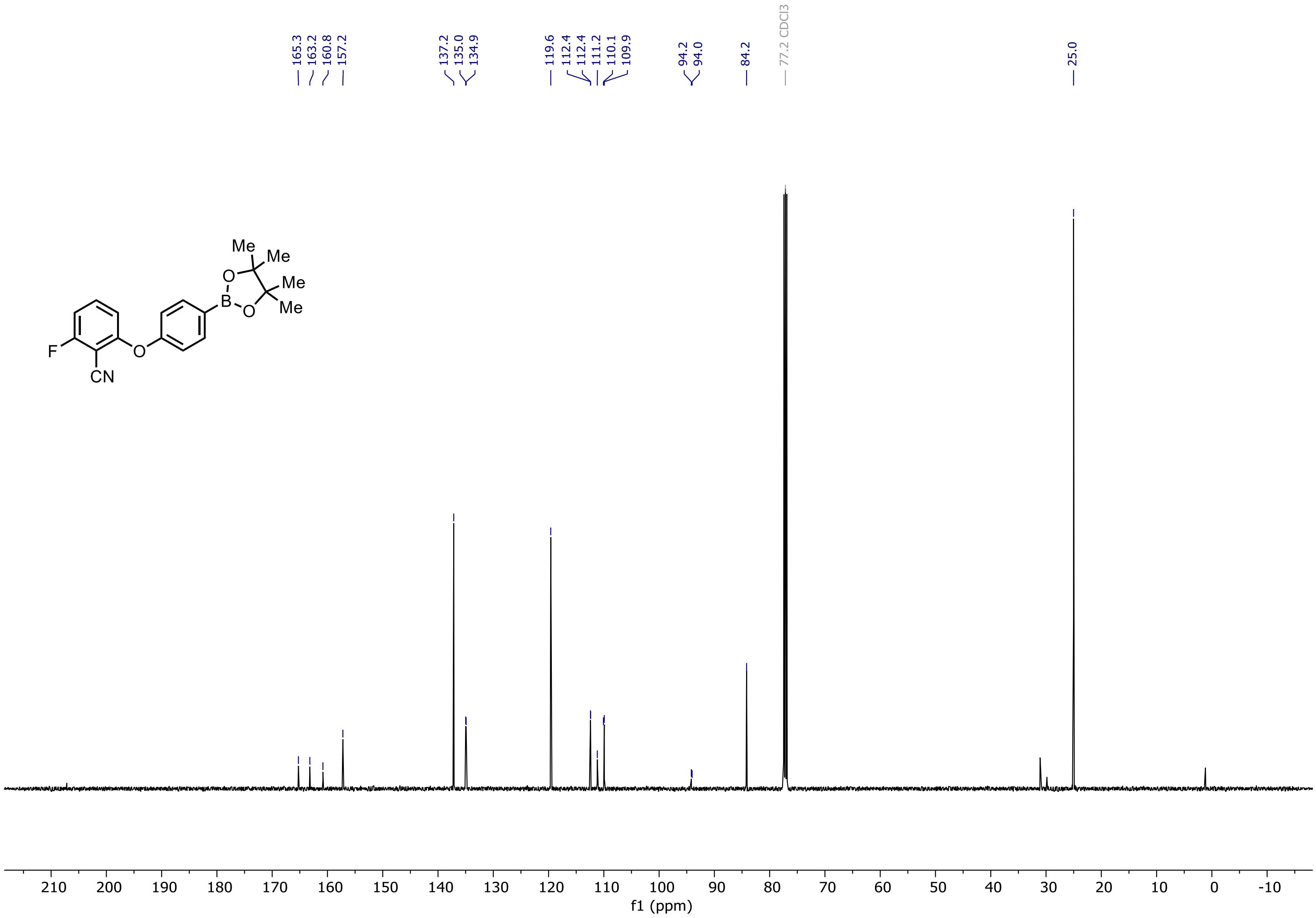

^{19}F NMR of 2-fluoro-6-(4-(4,4,5,5-tetramethyl-1,3,2-dioxaborolan-2-yl)phenoxy)benzonitrile (28)

$CDCl_3$, 470 MHz, 298 K

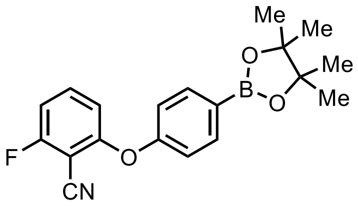

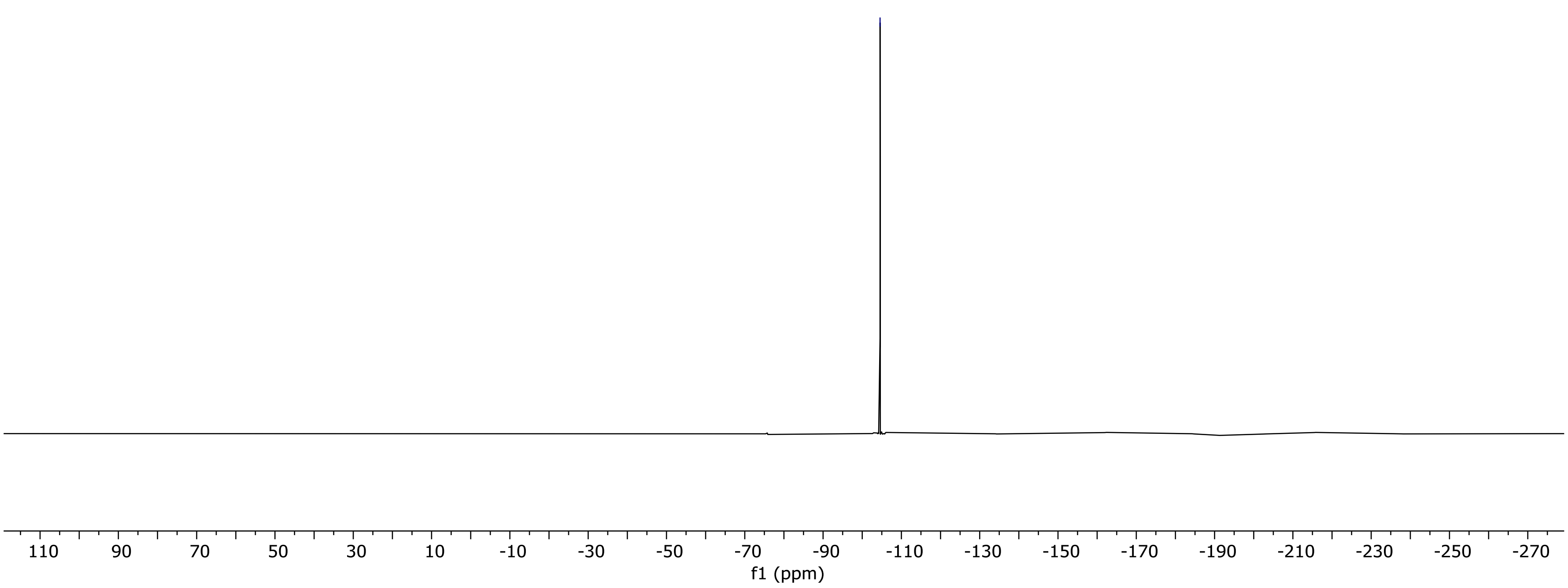

^{1}H NMR of 2-(4-allylphenyl)ethan-1-ol (29)

CDCl$_3$, 500 MHz, 298 K

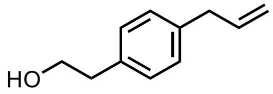

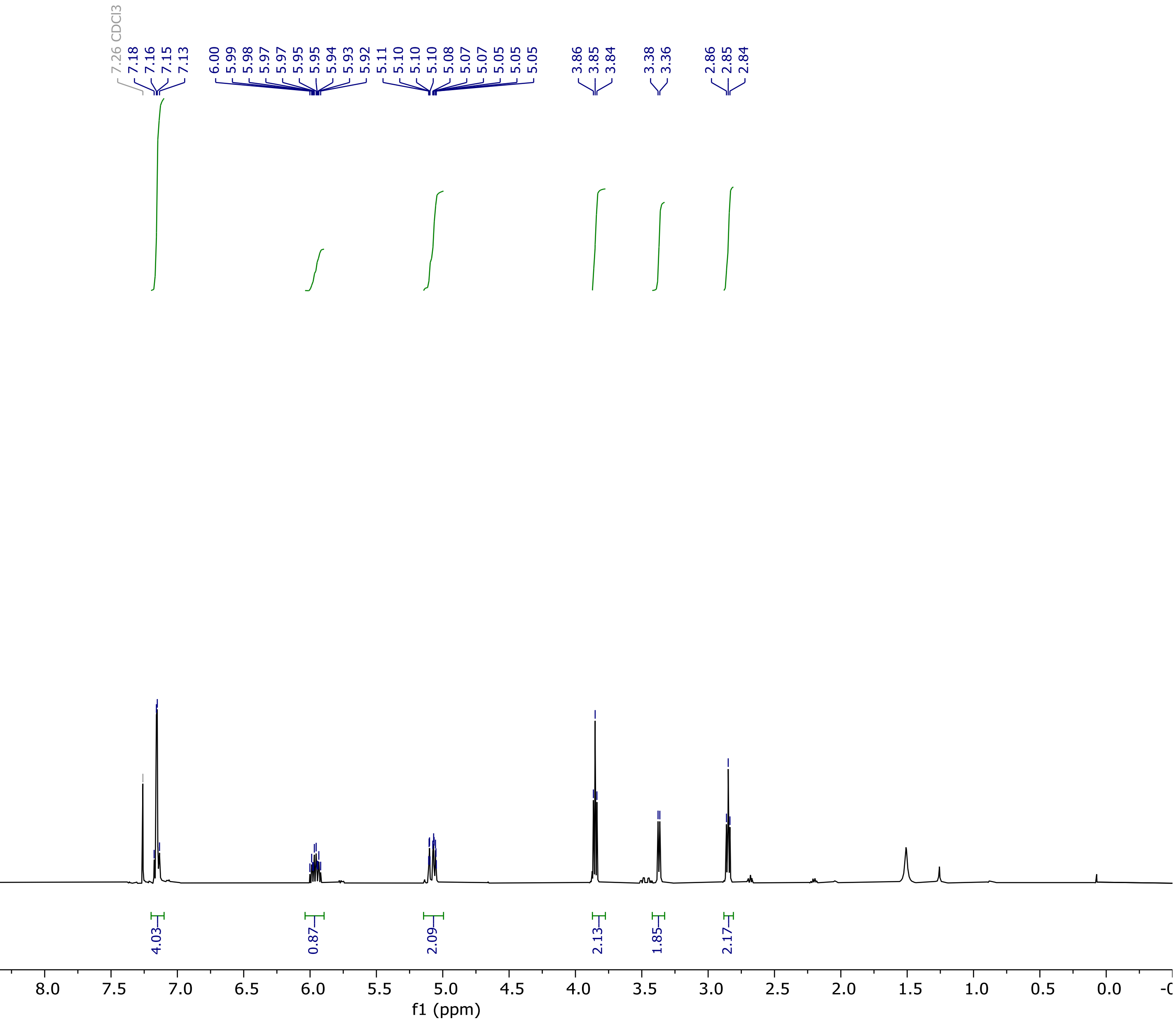

^{13}C NMR of 2-(4-allylphenyl)ethan-1-ol (29)

$CDCl_3$, 126 MHz, 298 K

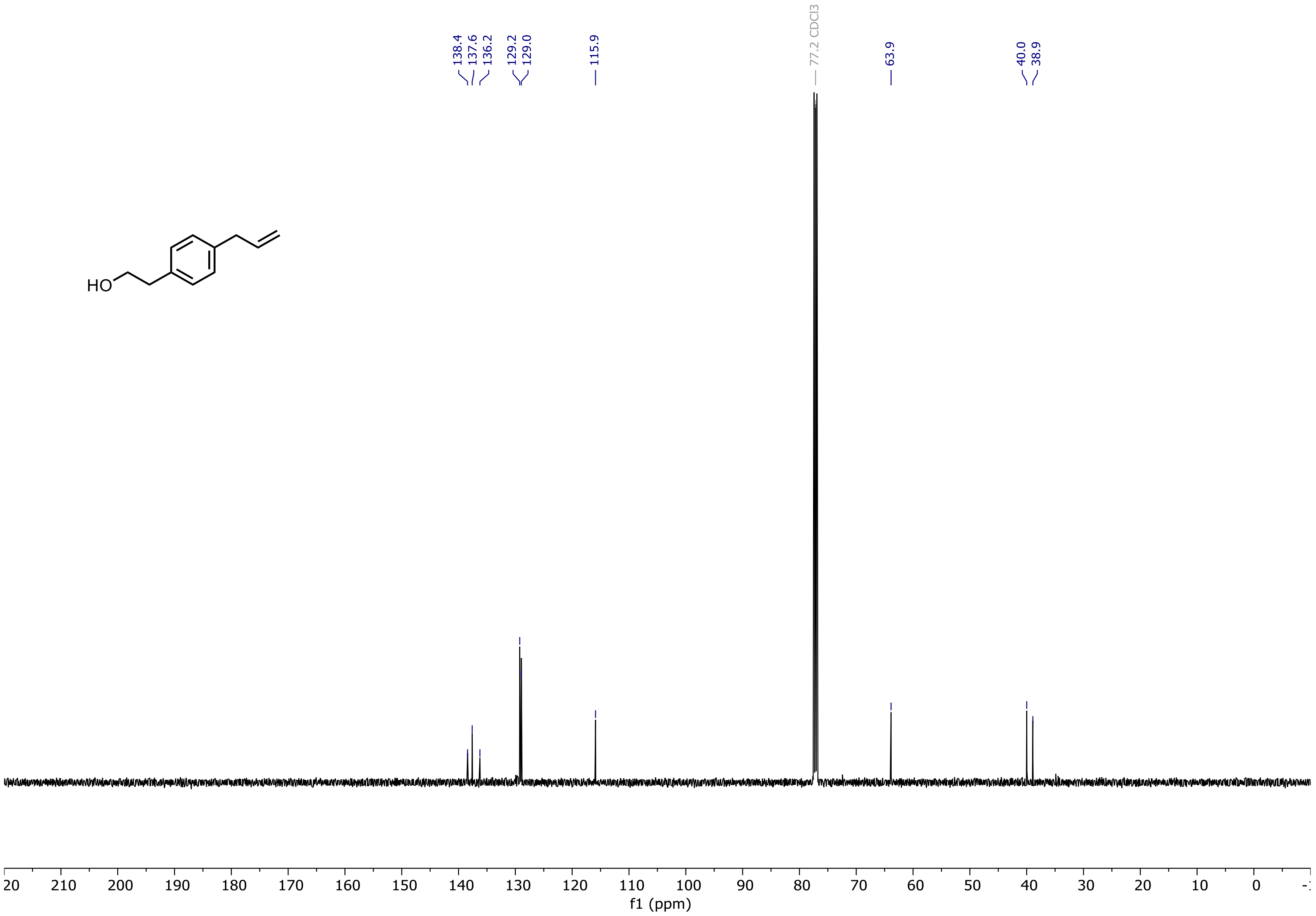

^{1}H NMR of methyl 5-iodo-2-methoxybenzoate (30)

$CDCl_3$, 500 MHz, 298 K

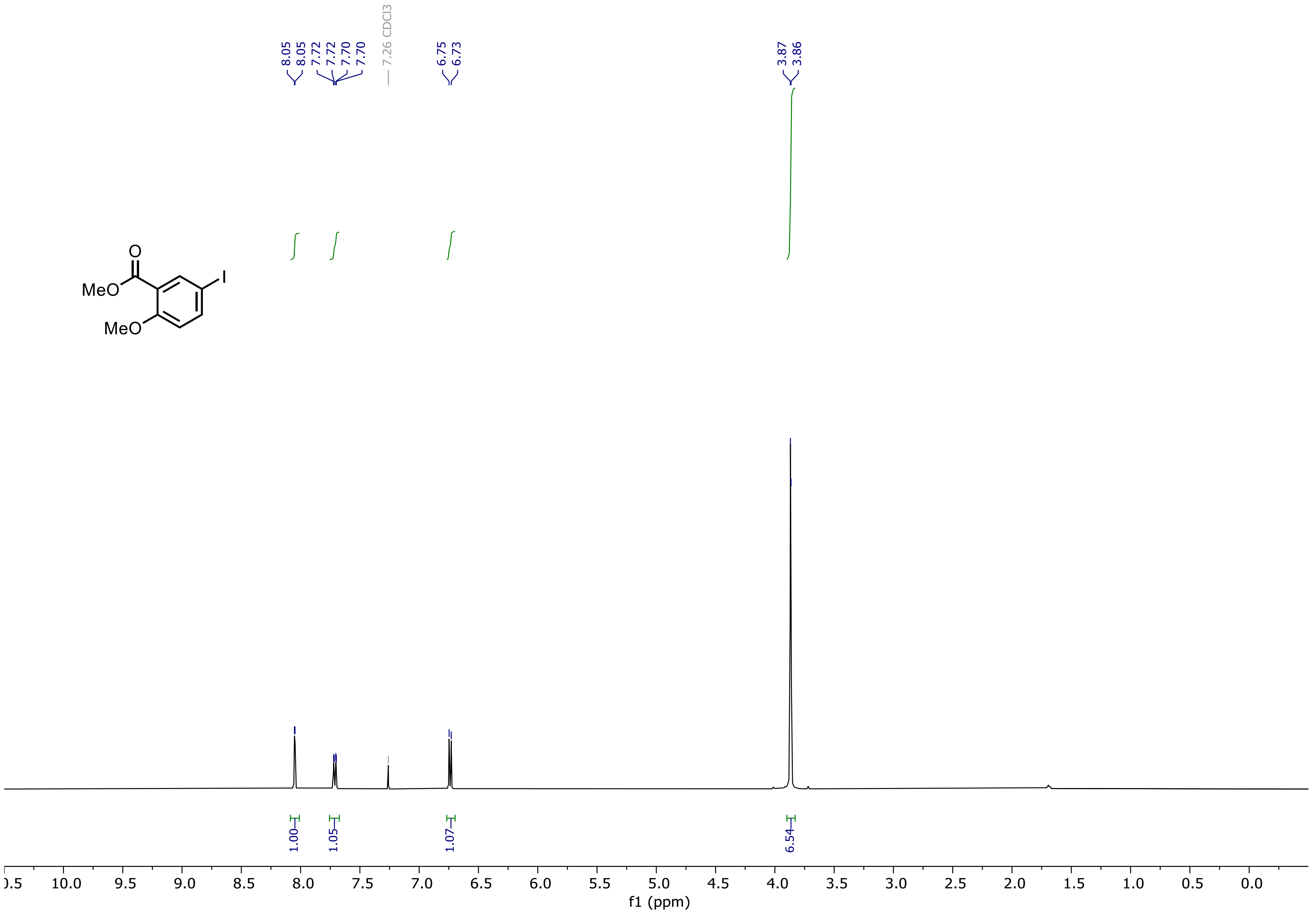

^{13}C NMR of methyl 5-iodo-2-methoxybenzoate (30)

$CDCl_3$, 126 MHz, 298 K

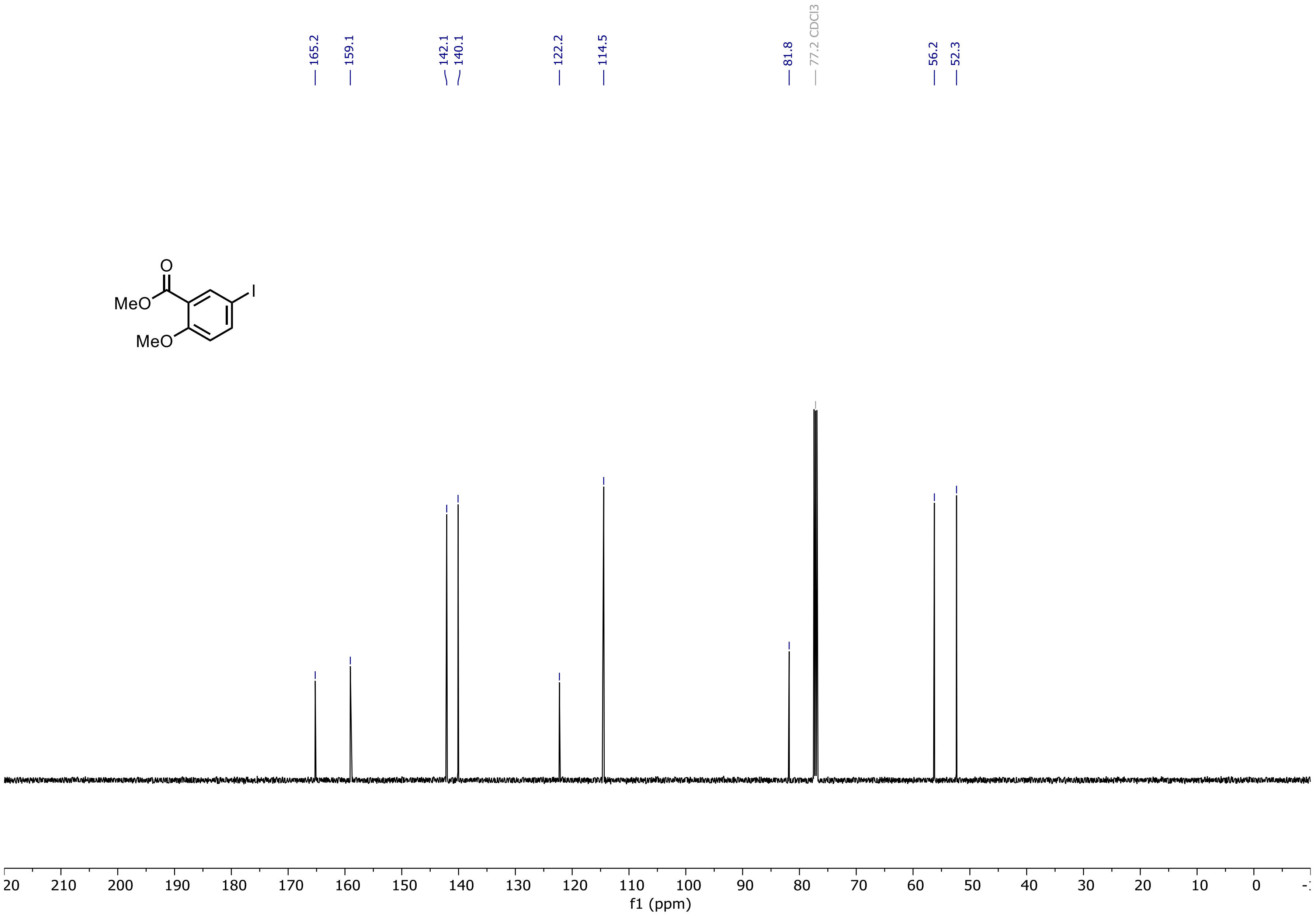

REFERENCES

1. G. R. Fulmer, A. J. M. Miller, N. H. Sherden, H. E. Gottlieb, A. Nudelman, B. M. Stoltz, J. E. Bercaw, K. I. Goldberg, *Organometallics*, **2010**, *29*, 2176–2179.
2. F. Berger, M. B. Plutschack, J. Riegger, W. Yu, S. Speicher, M. Ho, N. Frank, T. Ritter, *Nature*, **2019**, *567*, 223–228.
3. P. S. Engl, A. P. Häring, F. Berger, G. Berger, A. Peréz-Bitrián, T. Ritter, *J. Am. Chem. Soc.* **2019**, *141,* 13346–13351.
4. F. Ye, F. Berger, H. Jia, J. Ford, A. Wortman, J. Borgel, C. Genicot, T. Ritter, *Angew. Chem. Int. Ed.* **2019**, *58,* 14615–14619.
5. R. Sang, S. Korkis, W. Su, F. Ye, P. Engl, F. Berger, T. Ritter, *Angew. Chem. Int. Ed.* **2019**, *58,* 16161–16166.
6. J. Li, J. Chen, R. Sang, W-S. Ham, M. B. Plutschack, F. Berger, S. Chabbra, A. Schnegg, C. Genicot, T. Ritter, *Nat. Chem.* **2020**, *12,* 56–62.
7. F. Berger, E. M. Alvarez, N. Frank, K. Bohdan, M. Kondratiuk, L. Torkowski, P. S. Engl, J. Barletta, T. Ritter, *Org. Lett.* **2020,** *22*, 5671–5674.
8. E. M. Alvarez, M.B. Plutschack, F. Berger, T. Ritter, *Org. Lett.* **2020,** *22*, 4593–4596.
